# Directed evolution of an enantiocomplementary S_N_Arase reveals divergent catalytic features

**DOI:** 10.1038/s41467-026-76922-5

**Published:** 2026-08-21

**Authors:** Thomas M. Lister, George W. Roberts, Cristina Duran, Guillem Casadevall, Fei Zhao, Alexander A. V. Millman, Igor Larrosa, Sílvia Osuna, Anthony P. Green

**Affiliations:** 1 Manchester Institute of Biotechnology, The University of Manchester, Manchester, UK; 2 Department of Chemistry, School of Natural Science, The University of Manchester, Manchester, UK; 3 Institut de Química Computacional i Catàlisi (IQCC) and Departament de Química, Universitat de Girona, Girona, Spain; 4 ICREA, Barcelona, Spain

**Keywords:** X-ray crystallography, Enzymes, Biocatalysis, Enzyme mechanisms

## Abstract

Enzymes that catalyze non-natural C–C bond-forming reactions are powerful tools in asymmetric synthesis, yet reprogramming their active sites to invert stereochemical outcome remains challenging. Building on our recently engineered S_N_Arase, S_N_Ar1.3, which performs enantioselective nucleophilic aromatic substitutions with carbon nucleophiles, we now report the evolution of an enantiocomplementary biocatalyst (eS_N_Ar1.3) that displays enhanced activity and expanded substrate scope. Structural and computational analyses uncover both conserved and divergent features between S_N_Ar1.3 and eS_N_Ar1.3. Despite retaining similar electrophile binding poses and a conserved catalytic arginine, the halide-binding pocket of S_N_Ar1.3 has been abandoned in eS_N_Ar1.3. Instead, His23 has emerged as a key motif that works with Arg124 to accurately position the nucleophilic substrate. Calculations reveal that Arg124 also plays a crucial role in facilitating halide release during catalysis. Our study demonstrates how evolution can reshape enzyme mechanisms in unforeseen ways, highlighting the importance of exploring diverse trajectories to access new functions.

## Abstract (web summary)

Enzymes that catalyse non-natural C–C bond-forming reactions are of interest for asymmetric synthesis, but reprogramming their active sites to invert the stereochemical outcome remains challenging. Here, the authors report on the evolution of eS_N_Ar1.3 with inverted stereochemical outcome, enhanced activity and expanded substrate scope compared with S_N_Ar1.3, their previously engineered S_N_Arase.

## Introduction

Developing new families of enzymes for valuable chemical transformations is a primary objective of modern biocatalysis research^1–5^. With modern directed evolution pipelines, we are now able to enhance the activity of rudimentary protein catalysts by orders of magnitude to deliver specialized enzymes with functions that are unknown in nature^6–17^. Due to a high degree of active site sophistication, these engineered enzymes can offer rate accelerations and selectivity outcomes that surpass those achievable with small molecule catalysts, thus offering valuable tools for sustainable chemical synthesis^18–21^.

**Fig. 1:**
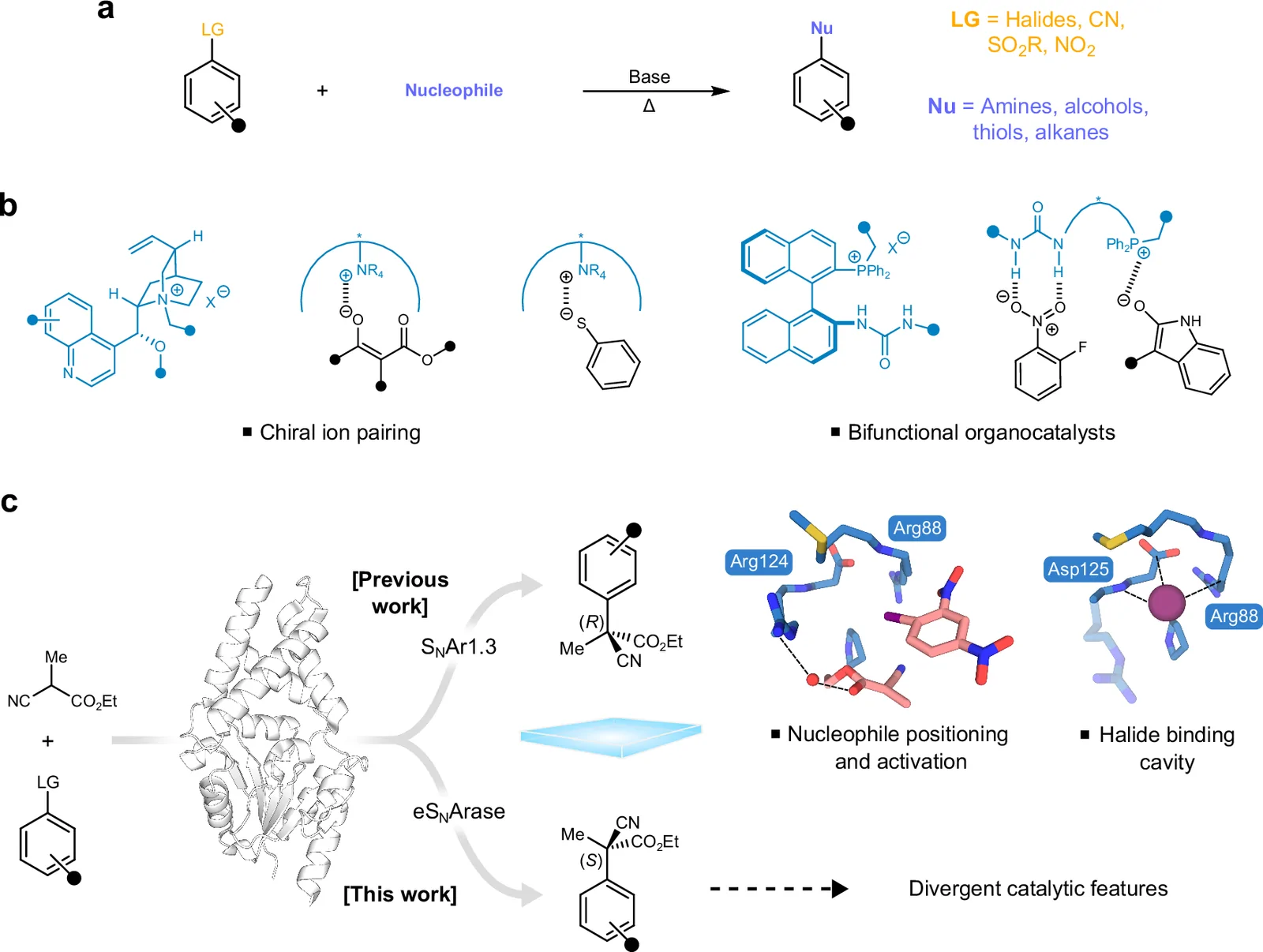
Nucleophilic aromatic substitutions (S_N_Ar). **a** S_N_Ar reactions involve the coupling of aromatic electrophiles with diverse nucleophilic coupling partners. **b** Examples of stereoselective S_N_Ar reactions using chiral organocatalysts. **c** Our previous work reported an engineered enzyme, S_N_Ar1.3, for efficient and selective S_N_Ar chemistry that constructs products containing all-carbon quaternary centers. Structural and mechanistic analysis of S_N_Ar1.3 revealed several active site features, including a catalytic Arg124 residue for nucleophile activation and positioning, and a halide binding site. This work describes the engineering of an enantiocomplementary S_N_Arase, eS_N_Ar1.3, which has distinctive catalytic features compared with S_N_Ar1.3.

In this regard, we recently reported an engineered enzyme, S_N_Ar1.3, for nucleophilic aromatic substitutions^22^. S_N_Ar processes involve the coupling of electron-deficient aryl halides with a range of nucleophilic partners (Fig. 1a) and are amongst the most widely used transformations in the pharmaceutical and agrochemical industries^23,24^. Despite their prevalence, there are only a handful of selective catalytic strategies known for promoting S_N_Ar chemistry that offer limited efficiency (Fig. 1b)^25–36^. To establish a biocatalytic approach to S_N_Ar chemistry, we uncovered promiscuous S_N_Ar activity in an enzyme that was previously engineered to accelerate enantioselective Morita-Baylis-Hillman reactions (Fig. 1c)^10,13^. This promiscuous activity was optimized over successive rounds of directed evolution to afford a proficient S_N_Arase biocatalyst that enabled construction of all-carbon quaternary centers^37^ with exquisite selectivity (>99% *e.e*.) using a range of nucleophilic and electrophilic coupling partners. Structural and biochemical characterization of the evolved enzyme shed light on the active site features responsible for efficient and selective S_N_Ar catalysis, including a halide binding site located directly adjacent to the electrophile binding pocket and a key catalytic Arg thought to be responsible for nucleophile activation and positioning. Interestingly, S_N_Ar1.3 was found to have an unusual halide leaving group reactivity trend which correlates with an increased affinity for heavier halide anions. In principle, this understanding of the S_N_Ar1.3 catalytic mechanism can be used to inform the design of future generations of S_N_Ar biocatalysts with enhanced properties^38^. However, having sampled only one evolutionary trajectory, we are mindful that alternative, and potentially superior, mechanistic solutions to enzymatic S_N_Ar chemistry may exist. With this in mind, we were keen to explore an alternative evolutionary trajectory starting from the common ancestor S_N_Ar1.0. To expand the synthetic utility of our biocatalytic S_N_Ar methodology and potentially encourage the emergence of new mechanistic outcomes, we elected to engineer an S_N_Arase that is enantiocomplementary^39–43^ to our recently reported enzyme S_N_Ar1.3.

In this study, we show that introduction of only a handful of mutations into S_N_Ar1.0 is sufficient to deliver an efficient, enantiocomplementary S_N_Arase with a substantially altered catalytic mechanism.

## Results

### Engineering an enantiocomplementary S_N_Ar enzyme

S_N_Ar1.0, the starting template for our previous engineering of S_N_Ar1.3, promotes the coupling of ethyl 2-cyanopropionate (**1**) and 2,4-dinitrochlorobenzene (**2**) with low selectivity (5% *e.e*. in favor of the (*R*)-enantiomer of **3**). We were therefore optimistic that S_N_Ar1.0 could serve as a template for the development of an enantiocomplementary S_N_Arase. To this end, we evaluated the selectivity of a panel of more active S_N_Ar1.0 variants, which were identified during round 1 of our previous evolution campaign, to identify those displaying inverted stereopreference. Pleasingly, from this screening we identified an A19L variant (subsequently referred to as eS_N_Ar1.0) that displays a 2.1-fold improvement in activity and affords **(*S*)−3** with 55% *e.e*..

**Fig. 2:**
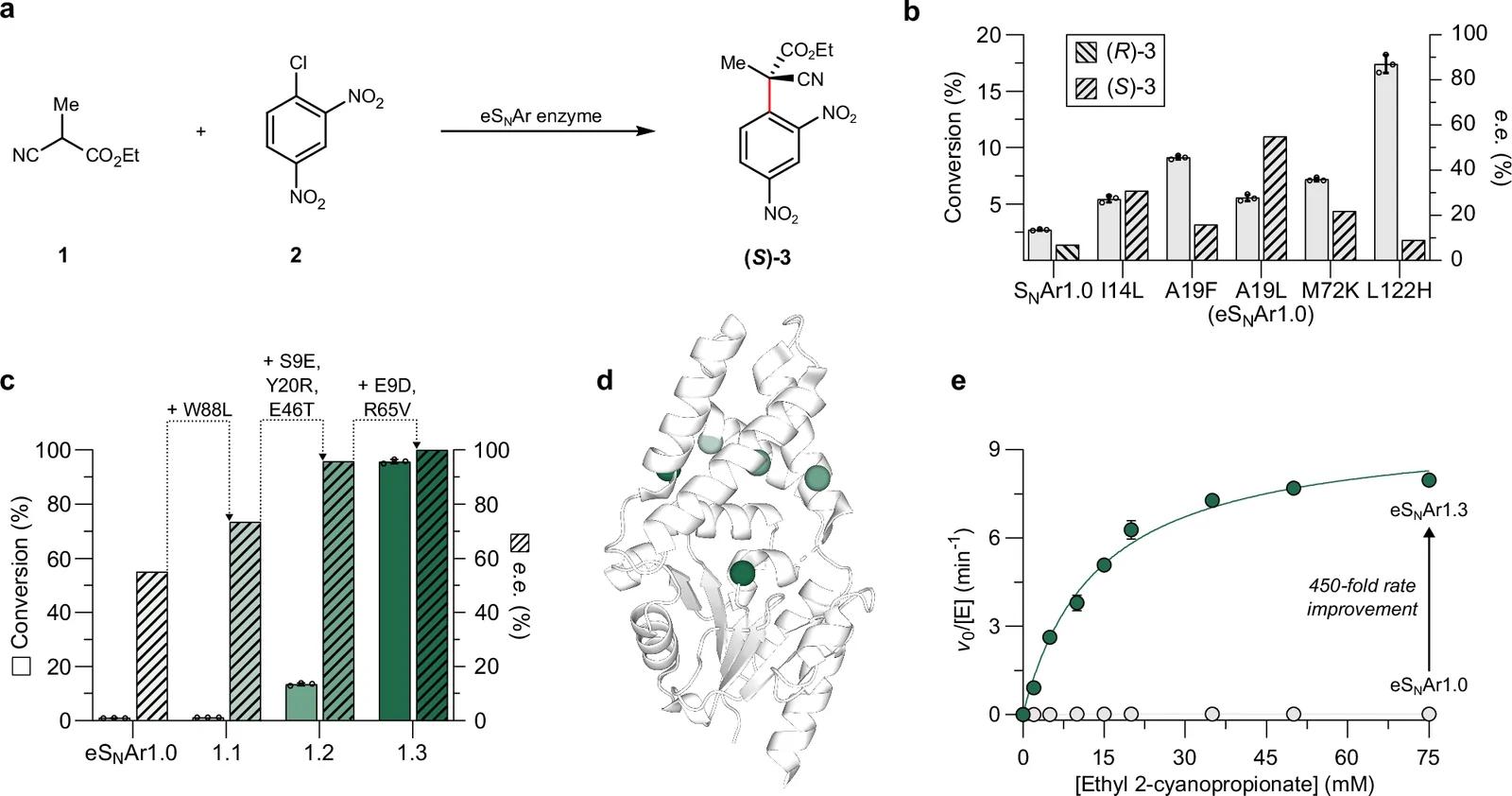
Engineering an enantiocomplementary S_N_Arase. **a** Chemical scheme showing the target S_N_Ar reaction between ethyl 2-cyanopropionate (**1**) and 2,4-dinitrochlorobenzene (**2**), generating product **(*S*)-3** containing an all-carbon quaternary carbon center. **b** Bar chart showing reaction conversion (gray bars) and selectivity (gray patterned bars) achieved by S_N_Ar1.0 and a panel of S_N_Ar1.0 variants that display inverted stereopreference for **3**. Reaction conditions: **1** (25 mM), **2** (2.5 mM), S_N_Ar1.0 variant (3 mol%) in PBS pH 8.0 with 10% *v*/*v* DMSO as a co-solvent, 18 h at 30 °C. **c** Bar chart showing reaction conversion (solid bars) and selectivity (patterned bars) achieved by enantiocomplementary S_N_Arase variants along the evolutionary trajectory. Reaction conditions: **1** (25 mM), **2** (2.5 mM), eS_N_Ar variant (0.2 mol%) in NaP_i_ pH 8.0 with 10% *v*/*v* DMSO as a co-solvent, 3 h at 30 °C. **d** Structure showing the six amino acid positions mutated in eS_N_Ar1.0 to afford eS_N_Ar1.3 mapped onto BH32.7 (PDB: 7O1D, Supplementary Figs. 2 and 3). Mutations are represented as spheres at the *C*_α_ and colored according to their order of introduction, corresponding to the variants in Fig. 2c. **e** Michaelis–Menten kinetic analysis of eS_N_Ar1.0 and eS_N_Ar1.3 reveal a 450-fold improvement in *k*_cat_ following evolution (0.0217 ± 0.0008 and 9.80 ± 0.48 min^−1^ for eS_N_Ar1.0 and eS_N_Ar1.3, respectively; Supplementary Fig. 9). Assays were performed at a fixed concentration of **2** (2.5 mM) and varying concentrations of **1** (2.0–75.0 mM) in NaP_i_ pH 8.0. The plots show the averaged initial rates, which were fitted to the Michaelis–Menten equation using Origin software. Data are the mean ± S.D. of measurements made in triplicate.

**Fig. 3:**
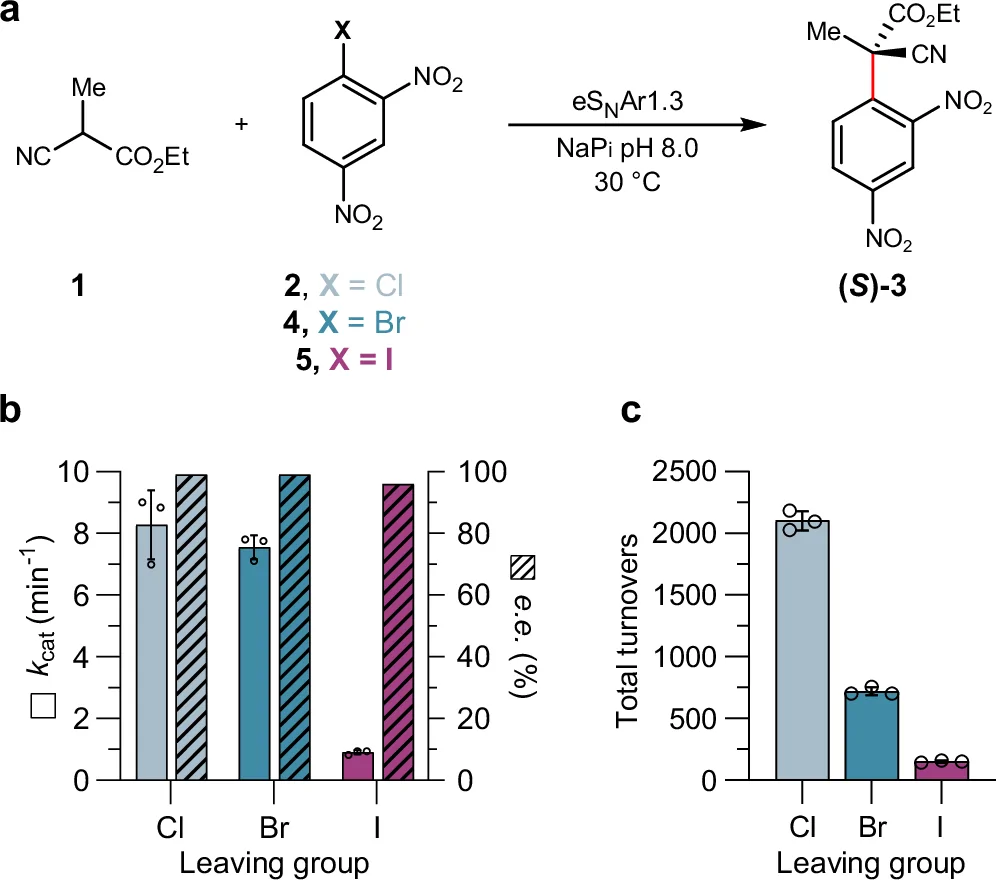
Effect of the halide leaving group on eS_N_Ar1.3 activity and selectivity. **a** Chemical scheme of the S_N_Ar reaction between **1** and different 2,4-dinitrohalobenzene electrophiles (**2,** **4** or **5**) catalyzed by eS_N_Ar1.3. **b** Bar chart showing the *k*_cat_ values (min^−1^, solid bars) and enantioselectivity (*e.e*., %, patterned bars) achieved by eS_N_Ar1.3 with **2,** **4** and **5**. Michaelis–Menten kinetic analysis of eS_N_Ar1.3 was performed at varying concentrations of **2,** **4** or **5** and saturating concentrations of nucleophile **1** (75 mM). The *k*_cat_ parameters were obtained from averaged initial rates fitted to the Michaelis–Menten equation using Origin software (Supplementary Fig. 13). **c** Bar chart showing the total number of turnovers eS_N_Ar1.3 can perform with different 2,4-dinitrohalobenzene electrophiles (Supplementary Fig. 6). Reaction conditions: **1** (10 equiv.), 2,4-dinitrohalobenzene (**2**, 2.5 mM; **4**, 1.5 mM; **5**, 1.0 mM), eS_N_Ar1.3 (0.001 mol% using **2**; 0.01 mol% using **4**; 0.05 mol% using **5**) in NaP_i_ pH 8.0 with 10% *v*/*v* DMSO at 30 °C. Data are the mean ± S.D. of measurements made in triplicate.

To further improve activity and selectivity, eS_N_Ar1.0 was subjected to three rounds of laboratory evolution (Fig. 2c and Supplementary Fig. 2), each individually randomizing between 20 and 30 residues in the active site and secondary coordination sphere using NNK degenerate codons. Individual library variants were arrayed in 96-well plates and evaluated as clarified cell lysate using a UPLC assay monitoring the conversion of **1** and **2** to S_N_Ar product **3**. The most active (*ca*. 1%) clones from each round were selected for further evaluation as purified proteins. Beneficial mutations identified in each round were subsequently combined by DNA shuffling. Following the evaluation of ~6000 clones, a highly selective eS_N_Ar1.3 variant that contains six additional mutations and produces **(*S*)−3** in >99% *e.e*. was identified (Supplementary Figs. 2 and 3). Interestingly, the thermostability of eS_N_Ar variants is essentially unchanged across the evolutionary trajectory (*T*_m_ ~ 78 °C, Supplementary Fig. 4). At the substrate concentrations used during evolution eS_N_Ar1.3 displays a 480-fold rate improvement compared with the starting template eS_N_Ar1.0 (Supplementary Fig. 5) and can perform >2000 turnovers (Fig. 3b, Supplementary Fig. 6). Interestingly the total turnover number can be further increased by addition of fresh nucleophile, suggesting that nucleophile decomposition rather than enzyme deactivation is limiting turnover (Supplementary Fig. 6). NMR analysis shows that nucleophile **1** undergoes slow non-enzymatic ester hydrolysis under the assay conditions (Supplementary Figs. 7 and 8). Detailed kinetic analysis shows that the observed improvements in catalytic activity predominantly arise from a 450-fold improvement in *k*_cat_ (0.0217 ± 0.0008 min^−1^ and 9.80 ± 0.48 min^−1^ for eS_N_Ar1.0 and eS_N_Ar1.3 respectively, Supplementary Fig. 9), alongside a more modest 2-fold improvement in *K*_M**2**_ (1.13 ± 0.20 mM and 0.52 ± 0.13 mM for eS_N_Ar1.0 and eS_N_Ar1.3 respectively, Supplementary Fig. 9). The *K*_M_ for the nucleophilic partner **1** is essentially unchanged following evolution (15.7 ± 1.61 mM and 13.7 ± 2.0 mM for eS_N_Ar1.0 and eS_N_Ar1.3 respectively, Supplementary Fig. 9). To demonstrate synthetic utility, a preparative scale biotransformation was performed with eS_N_Ar1.3 (0.15 mol%) to deliver 191 mg of **(*S*)−3** (98% conversion, 90% isolated yield, >99% *e.e*., Supplementary Figs. 10 and 11). High conversions and enantioselectivities for the production of **(*S*)-3** are also maintained using equimolar concentrations of **1** and **2** with eS_N_Ar1.3 (Supplementary Fig. 12).

We next explored the effect of the halide leaving group on eS_N_Ar1.3 activity using 2,4-dinitrohalobenzene electrophiles **2,** **4**, and **5** featuring chloride, bromide, and iodide leaving groups, respectively. In all cases, the enzyme displays high levels of stereocontrol (>95% *e.e*.). Kinetic analysis shows that *k*_cat_ follows the trend Cl ~ Br > I (8.28 ± 0.65, 7.55 ± 0.22, and 0.91 ± 0.04 min^−1^, respectively, Supplementary Fig. 13), which is similar to the activity trends observed in the uncatalyzed S_N_Ar process. The catalytic efficiency (*k*_cat_/*K*_M_electrophile_) is approximately 2-fold higher with bromide electrophile **4** versus **2** (27.4 ± 2.6 and 15.9 ± 4.2 mM^−1^ min^−1^ for **4** and **2**, respectively) due to its improved *K*_M_ value (0.52 ± 0.13 and 0.28 ± 0.03 mM for **4** and **2**, respectively). Interestingly, these observed activity trends with eS_N_Ar1.3 are in contrast with those found for S_N_Ar1.3, which preferentially reacts with iodide-containing electrophile **5** (*k*_cat_/*K*_M_electrophile_ I > Br > Cl). Notably, the catalytic efficiency of eS_N_Ar1.3 using bromide electrophile **4** is *ca*. 3-fold greater than S_N_Ar1.3 with its preferred iodide-containing electrophile **5**.

**Fig. 4:**
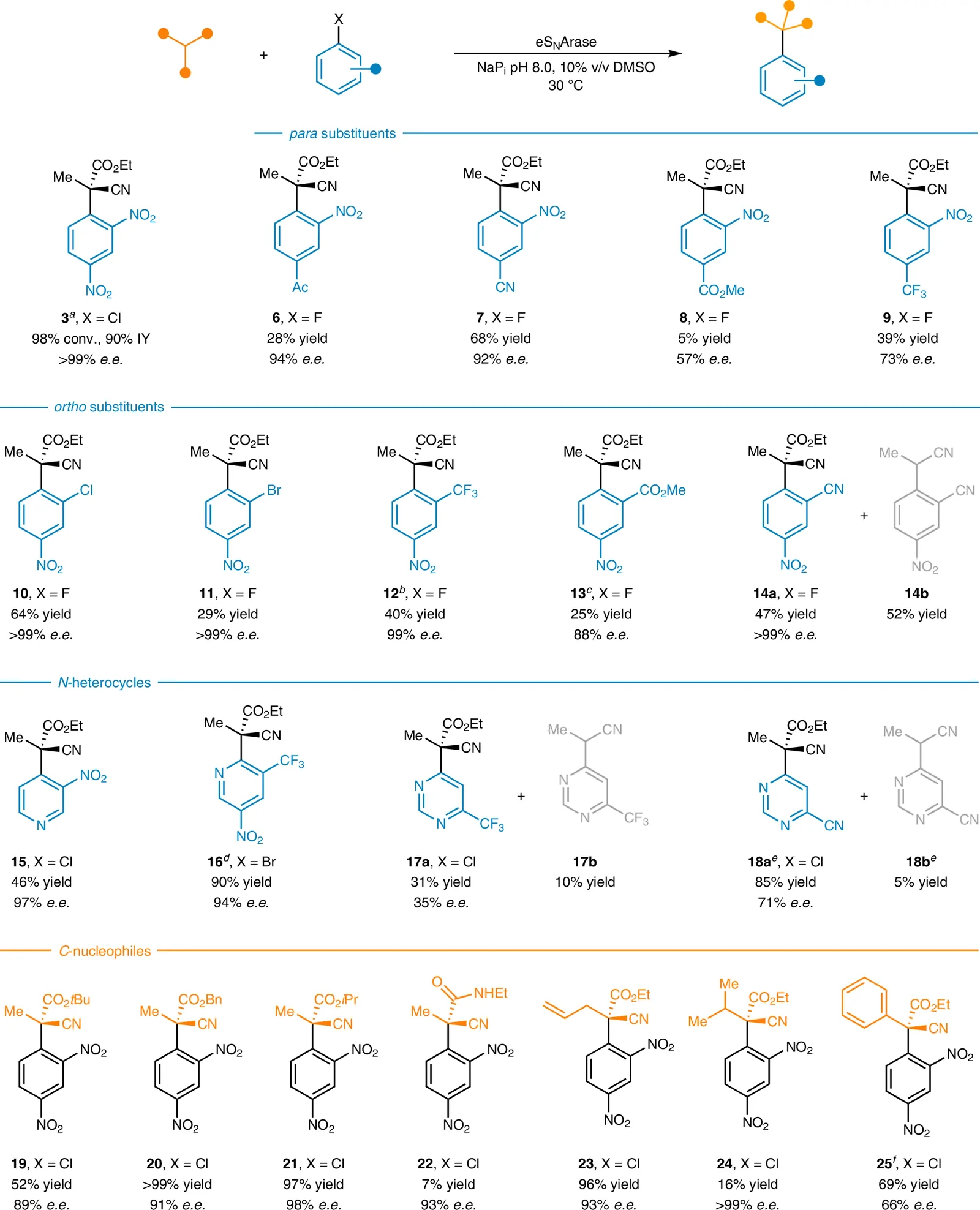
Substrate scope of eS_N_Ar1.3 and selected variants. eS_N_Ar1.3 and selected variants can accommodate a diverse range of electron-deficient (hetero)aryl halide electrophiles and tertiary carbon nucleophiles to generate products containing all-carbon quaternary centers. The stereochemistry of **3** and **25** was unambiguously assigned by X-ray diffraction of optically enriched (>98% *e.e*.) samples (ref.^22^ and Supplementary Figs. 16 and 17). The stereochemistry of **6–25** was tentatively assigned by analogy. For products **3–9**, **15–16**, **19–22**, and** 24–25**, eS_N_Ar1.3 provides the opposite stereochemical outcome to that achieved using S_N_Ar1.3, with the only exception being allyl-substituted nucleophile **23**, which is formed with only low levels of stereocontrol when using S_N_Ar1.3. The identity of the halide leaving group on the electrophilic coupling partner was selected on the basis of commercial availability, as well as assessment of reactivity and selectivity when several substrates were available. Product yields were determined by UPLC analysis by comparison of product peak areas against standard curves of authentic racemic standards. Reactions were performed using 0.5–5.0 mol% enzyme and 10 equivalents of nucleophile. Specific conditions for all reactions are presented in Supplementary Tables 1 and 2. ^*a*^ UPLC conversion and isolated yield (IY) from a preparative-scale reaction using eS_N_Ar1.3 (0.15 mol%) with electrophile **2** to produce optically pure **(*S*)−3** (191 mg, Supplementary Figs. 10 and 11). ^*b*^ Reaction performed using eS_N_Ar1.2(L13F R65V). ^*c*^ Reaction performed using eS_N_Ar1.2(R65V D66E). ^*d*^ Reaction performed using eS_N_Ar1.2(L13F R65V D66E). ^*e*^ Reaction performed using eS_N_Ar1.2(E9D L13F R65V). ^*f*^ Reaction performed in PBS pH 6.0 using 20% *v*/*v* DMSO as a co-solvent with 2 equivalents of nucleophile.

To evaluate its synthetic scope, we next explored the activity of eS_N_Ar1.3 and selected variants towards diverse electrophile and nucleophile coupling partners (Fig. 4). These enzymes can accommodate a wide range of *ortho*- and *para*-nitroarene derivatives, including those containing nitrile, trifluoromethyl, ester, ketone, and halide substituents, as well as pyridine rings, to afford S_N_Ar products with good to excellent *e.e*. Interestingly, eS_N_Ar1.3 variants can also be used to produce S_N_Ar adducts containing pyrimidine motifs, which are common motifs found in bioactive molecules. For the synthesis of **7**, we compared the performance of eS_N_Ar1.3 with electrophile substrates containing different halide leaving groups and observed a reactivity order of F > Cl ~ Br > I (Supplementary Fig. 14). In a small number of cases (**14a,** **17a**, and **18a**), the S_N_Ar products undergo a non-enzymatic hydrolysis-decarboxylation process to generate by-products **14b,** **17b**, and **18b** (Supplementary Fig. 15). In all other cases, a single reaction product was observed with remaining starting material making up the mass balance (>95% mass recovery was observed in all biotransformations). Pleasingly, eS_N_Ar1.3 can also process a range of α-cyano ester (**19–23** and **24**–**25**) and α-cyano amide (**24**) nucleophiles to furnish S_N_Ar adducts with generally excellent stereocontrol, including those containing 1,1-diaryl quaternary stereocentres (**25**) which are particularly challenging to access. Importantly, across a range of electrophilic and nucleophilic coupling partners (**3**–**9**; **15**–**16**; **19**–**22**; **24**–**25**) eS_N_Ar1.3 provides the opposite stereochemical outcome to that achieved using S_N_Ar1.3, with the only exception being allyl-substituted nucleophile **23** which is formed with only low levels of stereocontrol when using S_N_Ar1.3 (19% *e.e*. compared with 93% *e.e*. using eS_N_Ar1.3).

### Structure and mechanism of eS_N_Ar1.3

**Fig. 5:**
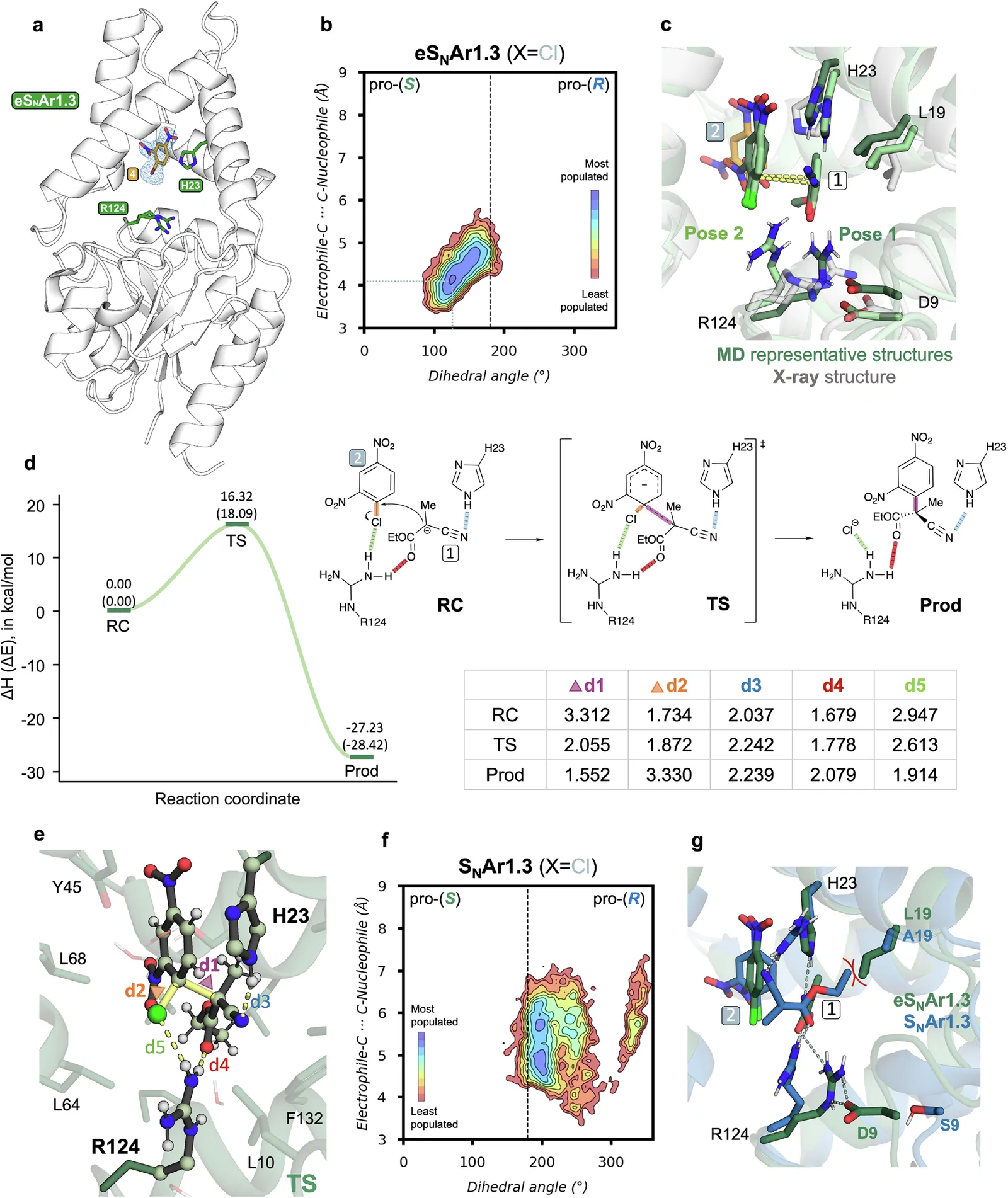
Structural and computational analysis of eS_N_Ar1.3. **a** X-ray crystal structure of eS_N_Ar1.3(K39A) complexed with substrate **4** (orange atom-colored sticks). Key catalytic Arg124 and His23 are shown in green-colored sticks. OMIT electron density map contoured at 4σ (blue mesh). **b** Reconstructed conformational landscape of eS_N_Ar1.3 in the presence of nucleophile **1** and aromatic substrate **2**. For conformational landscape reconstruction, the catalytic electrophile-nucleophile distance (*y*-axis) and a selected dihedral angle between substrates **1** and **2** to discriminate between pro-(*R*)/pro-(*S*) attacks (*x*-axis) are used. Dihedral angles of <180° are associated with a pro-(*S*) attack. Most populated conformations are colored in blue, whereas the least populated ones are depicted in red. **c** Representative MD structure obtained from the most populated conformation in (**b**) (green). The residue Arg124 is adopting two different conformations: (i) Arg124 is directly interacting with Asp9 and substrate **1** (named pose 1, shown in dark green), and (ii) Arg124 is interacting with substrates **1** and **2** (named pose 2, in light green). The catalytic distance is highlighted as a dashed line (the mean value is also provided). eS_N_Ar1.3(K39A) crystal soaked with **4** (orange) is also shown in gray as the reference. **d** QM/MM reaction profiles along the reaction coordinate, reported as ΔH and ΔE in kcal/mol. Relevant interatomic distances are listed to highlight the main structural differences along the reaction pathway. **e** Optimized transition state (TS) structure using QM/MM calculations. The QM region comprises substrates 1 and 2 and residues His23 and Arg124; atoms included in the QM region are shown as spheres. **f** Reconstructed conformational landscape of S_N_Ar1.3 in the presence of nucleophile **1** and aromatic substrate **2**. For conformational landscape reconstruction, the same features described in (**b**) are used. **g** Overlay of MD representative structures of eS_N_Ar1.3 and S_N_Ar1.3. The A19L mutation introduced in eS_N_Ar1.3 is blocking the pro-(*R*) attack, whereas the S9D mutation is stabilizing Arg124 in a productive pose for nucleophile activation and binding.

To gain insights into the eS_N_Ar1.3 catalytic mechanism, we undertook a series of biochemical, structural and computational studies. We first solved an X-ray crystal structure of eS_N_Ar1.3 in complex with electrophile substrate **4** to a resolution of 2.1 Å (PDB: 9SX8, Supplementary Table 9, Fig. 5a). As for S_N_Ar1.3, a K39A mutation was installed at the protein surface of eS_N_Ar1.3 to improve data resolution (Supplementary Fig. 18). Comparison of the substrate bound structures of S_N_Ar1.3 and eS_N_Ar1.3 reveals that the ligand adopts similar binding geometries in both proteins (Supplementary Fig. 19). However, in eS_N_Ar1.3 the substrate adopts a single well-defined binding mode, which contrasts the two distinct conformations observed in S_N_Ar1.3, plausibly explaining the substantially improved *K*_M_ values observed with eS_N_Ar1.3 (Fig. 3b). The key catalytic Arg124, involved in nucleophile activation in S_N_Ar1.3, is preserved in eS_N_Ar1.3. However, this residue is far more ordered in the eS_N_Ar1.3 structure due to its hydrogen bonding interactions with Asp9 that emerged during evolution (Supplementary Fig. 20). Mutation of Arg124 and Asp9 leads to substantial 660- and 24-fold reductions in activity, respectively (Supplementary Figs. 21 and 22), underscoring their importance to the eS_N_Ar1.3 catalytic mechanism.

A key structural and mechanistic feature of S_N_Ar1.3 was the presence of a halide binding site involving interactions with the backbone N–H and carboxylate sidechain of Asp125, which in turn forms an extended network of H-bonding interactions with Arg65 and Arg88. This halide binding site has been abandoned during evolution of eS_N_Ar1.3. Efforts to obtain iodide or bromide structures were unsuccessful (see Supplementary Information, Methods), likely reflecting the >10-fold increase in iodide IC_50_ values observed with eS_N_Ar1.3 versus S_N_Ar1.3 (1.20 ± 0.05 and 16.5 ± 2.8 mM for S_N_Ar1.3 and eS_N_Ar1.3, respectively, Supplementary Fig. 23). Notably, Arg65 and Arg88 were both mutated to hydrophobic residues during the evolution of eS_N_Ar1.3. While Asp125 was preserved, its mutation to Ala results in a modest 1.5-fold increase in activity (Supplementary Figs. 21 and 22), which contrasts with the substantial 22-fold decrease in activity observed upon the analogous mutation in S_N_Ar1.3.

A notable distinction between the S_N_Ar1.3 and eS_N_Ar1.3 evolutionary trajectories is that His23, the key catalytic nucleophile for MBH chemistry^10,44^, was abandoned in S_N_Ar1.3 but has been retained in eS_N_Ar1.3. Mutation of His23 in eS_N_Ar1.3 to either Ala or Phe leads to a significant 180-fold reduction in activity (Supplementary Figs. 21 and 22). We considered the possibility that His23 might serve as a catalytic nucleophile to accelerate S_N_Ar chemistry; however, this hypothesis was excluded as we were unable to accumulate any arylated protein intermediates within catalytically relevant timeframes (see Methods). To gain insights into the role of His23 in the eS_N_Ar1.3 catalytic mechanism, we turned to computational modeling.

To provide insights into the role of His23, Arg124 and Asp9 and the enantiocomplementary nature of eS_N_Ar1.3, we devised a computational pipeline based on the neural network Boltz-2 (ref. ^45^), semiempirical GFN2-xtb calculations^46^, Quantum Mechanics/Molecular Mechanics (QM/MM) calculations and molecular dynamics (MD) simulations (see Methods). Boltz-2 predictions were performed imposing an artificial bond between the nucleophile **1** and aromatic substrate **2**, thus modeling the plausible negatively charged Meisenheimer intermediate (Supplementary Fig. 24a). The predicted Boltz-2 pose was then further refined by means of cluster model calculations at the semiempirical GFN2-xtb level of theory and removing the artificial bond to generate a starting pose of both nucleophile and electrophile substrates for running MD simulations (Supplementary Fig. 24b). The refinement of the Boltz-2/xtb binding pose with 10 replicas of 100 ns MD simulations finally provided a reliable binding pose of nucleophile and aromatic substrate in the active site pocket of eS_N_Ar1.3. This binding pose was further studied by conducting 10 replicas of 100 ns unconstrained MD simulations, which were used to reconstruct a conformational landscape based on the catalytically relevant electrophile-nucleophile distance and a dihedral angle found to discriminate between pro-(*S*) and pro-(*R*) faces of the nucleophilic attack (Fig. 5b, Supplementary Fig. 25).

The conformational landscapes indicate that short catalytically productive distances between the electrophilic carbon and the nucleophile are sampled corresponding to the rear pro-(*S*) attack of **2** (i.e., dihedral angles <180°, Fig. 5b). As predicted by the GFN2-xtb calculations, the nucleophile is properly positioned in the active site pocket along the whole MD simulation time due to interactions with both His23, which establishes a stable hydrogen bond with the nitrile substituent of **1**, and Arg124 that is hydrogen bonded to the enolate anion (Fig. 5c). These hydrogen bonding interactions maintain **1** in a catalytically productive pose for enantioselective nucleophilic aromatic substitution, and potentially also serve to activate the nucleophile by promoting generation of its anionic form. To further clarify the roles of His23 and Arg124 in positioning **1** in a catalytically competent pose, we performed MD simulations of the H23A and R124A single mutants (Supplementary Fig. 26). Compared with eS_N_Ar1.3, both mutants show a marked reduction in the number of catalytic frames, consistent with the loss of activity observed experimentally (Supplementary Figs. 21 and 22). This reduction is equally observed in both mutants, therefore indicating that His23 and Arg124 are both required for properly positioning the nucleophile **1** near the electrophilic carbon for nucleophilic aromatic substitution.

As observed in the X-ray structure, Arg124 forms additional hydrogen-bonding interactions with Asp9 (Fig. 5c). This interaction is well maintained in the MD simulations; however, in some replicas, Arg124 is positioned close to the halide substituent of the aromatic substrate (Fig. 5c). We therefore hypothesized that direct interaction of Arg124 and the halide could facilitate halide release during aromatic nucleophilic substitution. We performed additional MD simulations and subsequent QM/MM calculations to provide mechanistic insight and clarify the role of Arg124 in the reaction (Fig. 5d, Supplementary Fig. 27). Our calculations indicate that the reaction proceeds through a concerted S_N_Ar mechanism, i.e., the Meisenheimer complex is not a stable intermediate, with an activation enthalpy of 16.3 kcal/mol. This value is in line with the experimentally determined *k*_cat_ (Fig. 3). The optimized transition state indicates that Arg124 helps position the nucleophile for the attack, forming a hydrogen bond at *ca.* 1.8 Å with the enolate, while also establishing a hydrogen bond with the halide at 2.6 Å (Fig. 5e), thereby facilitating halide release. This catalytic conformation of Arg124 is further stabilized by Asp9 through a water-mediated hydrogen bonding interaction (Fig. 5e, Supplementary Fig. 28). Substantially higher activation barriers were obtained when Arg124 did not interact with the halide, confirming its crucial role in promoting halide release during S_N_Ar catalysis (Supplementary Fig. 29). Together, the MD and QM/MM analyses suggest that Arg124 can adopt two main conformations: one in which it interacts directly with Asp9, and another in which the interaction with Asp9 is mediated by a water molecule. This latter conformation is essential for halide release and thus promotes the S_N_Ar reaction.

The overlay of the optimized transition state and reactant complex with the X-ray crystal structure of eS_N_Ar1.3, the parent MBH scaffold and several liganded S_N_Ar1.3 structures shows that the catalytically productive Arg124 conformation predicted by QM/MM is also observed crystallographically depending on the ligand present in the active site (PDB: 9FUO, Supplementary Fig. 30). This suggests that the proposed role of Arg124 in facilitating halide release during nucleophilic aromatic substitution might be shared by eS_N_Ar1.3 and S_N_Ar1.3.

To better understand the enantiocomplementary selectivities observed with eS_N_Ar1.3 and S_N_Ar1.3, we also computationally reconstructed the conformational landscape of S_N_Ar1.3 in the presence of **1** and **2** (Fig. 5f). As expected, the front attack pro-(*R*) face of **2** for nucleophilic aromatic substitution is predominantly explored (dihedral angles >180°, Fig. 5d) in S_N_Ar1.3. The conformational landscape also shows a rather high flexibility of the electrophile, as shown with the wide range of electrophile-nucleophile catalytic distances explored (3.5–7 Å). This flexibility is in line with the previously reported X-ray structure, which contains two different binding poses of the aromatic substrate (PDB: 9FUG). The higher proportion of catalytically unproductive nucleophilic attack distances also aligns with the lower catalytic activities observed for S_N_Ar1.3 with electrophile **2** compared with eS_N_Ar1.3. An overlay of the predicted nucleophile binding poses in eS_N_Ar1.3 and S_N_Ar1.3 highlights the critical role of the A19L mutation in achieving enantiocomplementary catalysis (Fig. 5f, Supplementary Fig. 31). In S_N_Ar1.3, the smaller Ala leaves enough space for the ethyl ester substituent of the nucleophile to bind in its preferred orientation. By contrast, in eS_N_Ar1.3, the bulkier Leu blocks this pose, forcing a rotated orientation that instead promotes the pro-(*S*) attack.

## Discussion

In this study, we have expanded the synthetic utility of S_N_Ar biocatalysts by developing an enzyme (eS_N_Ar1.3) that is enantiocomplementary to our previously reported system (S_N_Ar1.3). Notably, these enzymes can be accessed from a common ancestor by installing only a handful of mutations. Interestingly, although S_N_Ar1.3 and eS_N_Ar1.3 share some common active-site features and mechanistic elements, detailed structural, biochemical, and computational analysis reveals that divergent evolution has also given rise to some key mechanistic differences. In S_N_Ar1.3, a well-defined halide-binding site contributes to catalysis, whereas this feature was abandoned during the evolution of eS_N_Ar1.3. Instead, His23 emerged as a key catalytic residue in eS_N_Ar1.3, acting in synergy with Arg124 to accurately position the nucleophilic coupling partner. At the same time, Asp9 evolved to promote a productive orientation of the key catalytic Arg124, which our calculations reveal plays an important role in facilitating halide release during the nucleophilic aromatic substitution process.

These observations show that when developing new enzymes from primitive ancestors, directed evolution can lead to biocatalysts with distinctive mechanisms and active site geometries. The divergent catalytic sites that emerged in S_N_Ar1.3 and eS_N_Ar1.3 can now serve as inputs for future design campaigns to develop de novo S_N_Ar biocatalysts with enhanced properties.

## Methods

### Materials

Authentic, racemic product standards for compounds **3,** **6-9,** **15** and **16** were chemically synthesized according to procedures previously reported^22^. All chemicals and biological materials were obtained from commercial suppliers. Lysozyme and DNase I were purchased from Sigma-Aldrich; polymyxin B sulfate from Apollo Scientific; kanamycin monosulfate, LB agar, 2×YT media and L-arabinose from Formedium; *Escherichia coli* DH5α, Q5 DNA polymerase, T4 DNA ligase, NdeI and XhoI from New England Biolabs. Oligonucleotides were synthesized by Integrated DNA Technologies.

### eS_N_Arase expression and purification

For expression of S_N_Ar1.0 and eS_N_Ar1.0 variants, chemically competent *E. coli* DH5α cells were transformed with the requisite pBbE8k_S_N_Ar or _eS_N_Ar construct. Single colonies of freshly transformed cells were cultured (18 h at 37 °C, 200 r.p.m.) in 2×YT medium (5 mL) containing kanamycin monosulfate (25 µg mL^−1^). Starter cultures (500 µL) were used to inoculate 2×YT medium (50 mL) supplemented with kanamycin monosulfate (25 µg mL^−1^). Cultures were grown (37 °C, 200 r.p.m.) to an optical density at 600 nm (OD_600_) of about 0.6. Protein expression was induced with the addition of L-arabinose (10 mM final concentration). Induced cultures were incubated (20 h at 25 °C), and the cells were subsequently collected by centrifugation (3220 × *g* for 10 min). Pelleted cells were resuspended in lysis buffer (50 mM HEPES, 300 mM NaCl, 20 mM imidazole, pH 7.5) and lysed by sonication. Cell lysates were cleared by centrifugation (27,216 × *g* for 30 min) and supernatants were subjected to affinity chromatography using Ni-NTA agarose (QIAGEN). Purified protein was eluted using elution buffer (50 mM HEPES, 300 mM NaCl, 250 mM imidazole, pH 7.5). Proteins were desalted using 10DG desalting columns (Bio-Rad) with the requisite storage buffer and analyzed by sodium dodecyl sulfate–polyacrylamide gel electrophoresis. Proteins were aliquoted, flash-frozen in liquid nitrogen, and stored at −80 °C. Protein concentrations were determined by measuring the absorbance at 280 nm using calculated extinction coefficients (ExPASy ProtParam); extinction coefficients of 27,390 M^−1^ cm^−1^ for S_N_Ar1.0 eS_N_Ar1.0, 21,890 M^−1^ cm^−1^ for eS_N_Ar1.1, and 20,400 M^−1^ cm^−1^ for eS_N_Ar1.2 and eS_N_Ar1.3.

### eS_N_Arase mass spectrometry

Purified protein samples were buffer-exchanged into 0.1% acetic acid using a 10 k MWCO Vivaspin unit (Sartorius) and diluted to a final concentration of 0.5 mg mL^−1^. Mass spectrometry was performed using a 1200 series Agilent LC system, with a 5 µL injection into 5% acetonitrile (with 0.1% formic acid) and desalted inline for 1 min. Protein was eluted over 1 min using 95% MeCN with 5% H_2_O. The resulting multiply charged spectrum was analyzed using an Agilent 6510 Q-TOF instrument and deconvoluted using Agilent MassHunter software.

### Construction of eS_N_Arase libraries

For site saturation mutagenesis, positions were individually randomized using degenerate NNK codons. DNA libraries were constructed by overlap extension polymerase chain reaction (PCR). Primers for library generation are given in Supplementary Table 7. Assembled genes and the pBbE8k vector were digested using NdeI and XhoI endonucleases, gel-purified, and subsequently ligated using T4 DNA ligase in a 4:1 ratio, respectively. Ligations were transformed into *E. coli* DH5α cells; the resulting colonies were pooled, and plasmid DNA was extracted using a Miniprep Kit (QIAGEN) to yield plasmid DNA for each library. Sequencing was performed by Source BioScience.

### DNA shuffling by overlap extension PCR

After each round of screening, beneficial mutations were combined by DNA shuffling of fragments generated by overlap extension PCR. Primers were designed that encoded either the parent amino acid or the identified mutation. These primers were used to generate short fragments that were gel-purified and mixed for assembly of the full-length gene by overlap extension PCR. Final full-length genes contain all possible combinations of mutations at specified positions. Genes were cloned as described above.

### Screening of eS_N_Arase library variants

For protein expression and screening, all transfer and aliquoting steps were performed using a Hamilton liquid-handling robot. Chemically competent *E. coli* DH5α cells were transformed with the appropriate library plasmids. Freshly transformed colonies were used to inoculate 2×YT medium (150 μL) supplemented with kanamycin sulfate (25 μg mL^−1^) in Corning Costar 96-well microtitre round or flat-bottom plates. Each plate also contained six freshly transformed clones of the parent template and two clones of pBbE8k_RFP as an internal reference. Plates were incubated overnight (30 °C, 80% humidity, 850 r.p.m.), then an aliquot of overnight culture (20 µL) was used to inoculate 2×YT medium (480 μL) supplemented with kanamycin sulfate (25 μg mL^−1^). The cultures were incubated (30 °C, 80% humidity, 850 r.p.m.) until an OD_600_ of about 0.6 was reached, and L-arabinose was added (10 mM final concentration). Induced plates were incubated (20 h, 30 °C, 80% humidity, 850 r.p.m.). Cells were collected by centrifugation (2900 × *g* for 10 min). The supernatant was discarded, and the pelleted cells were resuspended in lysis buffer (400 μL: PBS or NaP_i_ pH 8.0 buffer supplemented with lysozyme (1.0 mg mL^−1^), polymyxin B (0.5 mg mL^−1^), and DNase I (10 μg mL^−1^)) and incubated (2 h, 30 °C, 80% humidity) with shaking (850 r.p.m.). Cell debris was removed by centrifugation (2900 × *g*, 10 min) to afford clarified cell lysate that was immediately used in biotransformations.

### Screening of round 1 and 2 variants

Clarified lysate (50 μL) was added to a 96-well microtitre plate, then the reaction was initiated by the addition of assay mix (50 μL) containing 2,4-dinitrochlorobenzene (2.5 mM final concentration) and ethyl-2-cyanopropionate (25 mM final concentration) in PBS pH 8.0 with DMSO (10% *v*/*v* final concentration). Reactions were heat-sealed and incubated overnight (30 °C, 850 r.p.m., 80% humidity). then quenched with the addition of MeCN (100 μL), heat-sealed and incubated (1 h, 30 °C, 80% humidity, 850 r.p.m.). Precipitated proteins were removed by centrifugation (2900 × *g*, 10 min). An aliquot of the clarified, quenched reaction mixture (100 µL) was transferred to a Greiner 96-well polypropylene microtitre plate and heat-sealed with pierceable foil. Reactions were evaluated by HPLC analysis as described below.

### Screening of round 3 variants

Following the second round of enzyme engineering, a 4-fold improvement in activity was achieved with eS_N_Ar1.2 when switching from PBS pH 8.0 to NaP_i_ pH 8.0 (Supplementary Fig. 1). All subsequent assays and biotransformations were performed in NaP_i_ pH 8.0 buffer.

Prior to removal of cell debris, each well was diluted with NaP_i_ pH 8.0 (1 mL). Clarified lysate (50 μL) was added to a 96-well microtitre plate, then the reaction was initiated by the addition of assay mix (50 μL) containing 2,4-dinitrochlorobenzene (2.5 mM final concentration) and ethyl-2-cyanopropionate (5 mM final concentration) in NaP_i_ pH 8.0 with DMSO (10% *v*/*v* final concentration). Reactions were heat-sealed and incubated for 2 h (30 °C, 850 r.p.m., 80% humidity). then quenched with the addition of MeCN (100 μL), heat-sealed and incubated (1 h, 30 °C, 80% humidity, 850 r.p.m.). Precipitated proteins were removed by centrifugation (2900 × *g*, 10 min). An aliquot of the clarified, quenched reaction mixture (100 µL) was transferred to a Greiner 96-well polypropylene microtitre plate and heat-sealed with pierceable foil. Reactions were evaluated by HPLC analysis as described below.

Following each round, the most active variants (*ca*. 1%) were rescreened as purified proteins using the HPLC assay. Proteins were produced and purified as described above. However, starter cultures were inoculated from glycerol stocks prepared from the original overnight cultures.

### General procedure for analytical-scale biotransformations with eS_N_Arase variants

Analytical-scale biotransformations (typically 100 μL) were performed in Greiner 96-well polypropylene microtitre plate or Eppendorf microcentrifuge tubes (1.5 mL) using **1** (1.0 to 25 mM), **2,** **4** or **5** (1.0, 1.5 or 2.5 mM respectively) and the relevant S_N_Ar or eS_N_Ar variant at the specified concentration in PBS or NaPi (pH 8.0) with 10% *v*/*v* DMSO co-solvent at 30 °C for the specified time period. For analysis of conversion by reverse-phase UPLC, reactions were quenched with MeCN (1 volume). Quenched reactions were shaken (850 r.p.m.) for 30 min. Precipitated protein was removed by centrifugation (2900 × *g* or 14,000 × *g* for 15 min, for reactions in 96-well microtitre plates or microcentrifuge tubes, respectively) and the supernatant transferred to a fresh microtitre plate or polypropylene autosampler vial for UPLC analysis (see “Chromatographic analysis” section). For normal-phase chiral HPLC analysis, the substrates and products were extracted with methyl tert-butyl ether (MTBE; 2 volumes). Centrifugation (14,000 × *g* for 10 min) was used to remove precipitated protein and achieve biphasic separation; then the organic phase was transferred to a polypropylene autosampler vial and directly injected onto the normal-phase HPLC.

### Chromatographic analysis of analytical-scale biotransformations

UPLC analysis was performed on a 1290 Infinity II LC system (Agilent) with a Kinetex 5 µm XB-C18 100 Å LC Column, 50 × 2.1 mm (Phenomenex). The separation methods for all substrates/products and extinction coefficients used to calculate the conversion are reported in Supplementary Tables 3 and 4, respectively, measured at 260 nm with an injection volume of 3 μL. For library screening, an isocratic method using 35% MeCN in MQ H_2_O (with 0.1% TFA) at 1.2 mL min^−1^ for 1.6 min. For other analytical-scale biotransformations, electrophile standards and chemically synthesized product standards (**3** to **25**) were characterized by eluting over 10 min using a gradient of 5–95% MeCN in MQ H_2_O (with 0.1% TFA) at 1 mL min^−1^. Peaks were assigned by comparison with chemically synthesized standards, and the peak areas were integrated using Agilent’s OpenLab software.

Chiral analysis was performed using a HPLC 1260 system (Agilent). For all products, the major stereoisomer formed in the biotransformations was assigned by analogy to eS_N_Ar1.3-derived **(*S*)−3**. Peaks were assigned by comparison with chemically synthesized standards and peak areas were integrated using Agilent’s OpenLab software. Separation methods for all product enantiomers used to determine *e.e*. are reported in Supplementary Table 5.

### Protein melting temperature analysis

The melting temperatures (*T*_m_) of S_N_Ar1.0 and eS_N_Ar variants were determined using differential scanning fluorimetry using a Bio-Rad CFX Connect Real Time System on the fluorescence channel set to use the 450-490 nm excitation and 515-530 nm emission filters. A 50 µL sample of each protein at 10 µM was prepared in 50 mM NaP_i_ pH 8 with a final concentration of 1x SYPRO Orange dye stock solution (Invitrogen) in optically clear, lidded PCR tubes (Life Technologies). The temperature was increased from 25 to 95 °C in 0.5 °C increments and held at each temperature for 5 s before collecting the measurement.

### Michaelis–Menten kinetic characterization

Initial velocity (*v*_0_) versus [ethyl 2-cyanopropionate] kinetic data were measured using His_6_-tagged purified eS_N_Ar1.0 (varying concentration) and eS_N_Ar1.3 (varying concentration), a fixed concentration of **2** (2.5 mM) and varying concentrations of **1** (2.0–75.0 mM). Reactions were performed with a volume of 500 μL in NaPi pH 8.0 with 10% *v*/*v* DMSO co-solvent and were incubated at 30 °C with shaking (850 r.p.m.). eS_N_Ar1.0 was sampled at 45 min intervals and eS_N_Ar1.3 was sampled at 10 min intervals. Aliquots (50 μL) were quenched by addition to a Greiner 96-well polypropylene microtitre plate containing MeCN (2 volumes) and were prepared for analysis by UPLC as described above (see “Chromatographic analysis” section). *v*_0_ versus [2,4-dinitrochlorobenzene], [2,4-dinitrobromobenzene] and [2,4-dinitroiodobenzene] kinetic data in NaP_i_ pH 8.0 were measured using a fixed concentration of **1** (75 mM) and varying concentrations of electrophile (0.05–2.50 mM) and S_N_Ar1.3 (varying concentration) as described above. The plots of the averaged initial rates were fitted to the Michaelis–Menten equation using Origin software (Eq. 1):

1 $${{{\rm{Y}}}}={V}_{max}\times {{{\rm{X}}}}/({K}_{M}+{{{\rm{X}}}})$$

### Total turnover numbers

eS_N_Ar1.3-catalyzed biotransformations were performed in screw cap glass vials (1.5 ml) in triplicate using **1** (10 equiv) and **2,** **4** or **5** (2.5, 1.5 or 1.0 mM respectively) in NaP_i_ pH 8.0 with 10% *v*/*v* DMSO (Fig. 3c and Supplementary Fig. 9). Reactions were incubated at 30 °C with shaking (850 r.p.m.) and aliquots (50 μL) were taken at 3, 18, 42, 76 and 100 h, added to Eppendorf microcentrifuge tubes (1.5 mL) containing MeCN (2 volumes, 100 μL) then quenched samples shaken (30 °C, 850 r.p.m., 15 min) and precipitated proteins removed by centrifugation (14,000 × *g* for 10 min). An aliquot of the supernatant (85 μL) was transferred to a Greiner 96-well polypropylene microtitre plate and analyzed using UPLC.

To understand what limits total turnover number in eS_N_Ar1.3-catalyzed transformations, biotransformations were performed in screw-cap glass vials (1.5 ml) in triplicate using eS_N_Ar1.3 (0.25 μM), **1** (25 mM) and **2** (2.5 mM) in NaP_i_ pH 8.0 with 10% *v*/*v* DMSO at a final volume of 600 μL. Reactions were incubated at 30 °C with shaking (850 r.p.m.) and aliquots (50 μL) were taken at 2, 6, 23, 45, 71, 94 and 119 h, added to Eppendorf microcentrifuge tubes (1.5 mL) containing MeCN (2 volumes, 100 μL) then quenched samples shaken (30 °C, 850 r.p.m., 15 min) and precipitated proteins removed by centrifugation (14,000 × *g* for 10 min). An aliquot of the supernatant (85 μL) was transferred to a Greiner 96-well polypropylene microtitre plate and analyzed using UPLC. After 119 h, to the remaining 250 μL reaction mixtures was added eS_N_Ar1.3 (5 μL, 13 μM in NaP_i_ pH 8.0) or **1** (5 μL, 1.27 M in DMSO) or eS_N_Ar1.3 (2.5 μL, 26 μM in NaP_i_ pH 8.0) and **1** (2.5 μL, 2.54 M in DMSO). Aliquots were then subsequently taken at 2, 24, and 46 h as described above.

### Protein arylation assay

To investigate the accumulation of arylated protein intermediates in eS_N_Ar1.3 variants, the requisite protein (eS_N_Ar1.3 or eS_N_Ar1.3(H23F), both 250 μM) and **2** (2.5 mM) in NaP_i_ (pH 8.0) with 10% *v*/*v* DMSO co-solvent were incubated at 30 °C for 30 min. The proteins were diluted 10-fold with NaP_i_ pH 8.0, then immediately analyzed by protein mass spectrometry as described above.

### Data collection for small molecule crystals

Crystals suitable for diffraction were isolated by slow evaporation of an EtOAc/hexane solution. Single-crystal X-ray diffraction data for compound **25** were collected at 100 K on a Rigaku FR-X equipped with a Hypix6000HE detector and Oxford cryosystem. Data were collected with Cu – Κ*α* (*λ* = 1.54184 Å) radiation. Data were collected using the CrysAlisPro program.

### Structure determination and refinements for small molecule crystals

Data processing and reduction were performed with CrysAlisPro. Empirical absorption correction was implemented with the SCALE3 ABSPACK algorithm. The crystal structure was solved and refined using the SHELX suite of programs in Olex2 (refs. ^2,3^). All non-hydrogen atoms were refined anisotropically. Hydrogen atom positions were calculated and refined with fixed isotropic displacement parameters. Absolute structure determination was based on anomalous dispersion. The absolute configuration of compound **25** was found to be (*R*)^47,48^.

### Protein crystallization, refinement and model building

The structure presented contained an additional K39A mutation, which improved the resolution of the corresponding diffraction data. K39A had a minimal effect on the rate or selectivity of eS_N_Ar1.3 (Supplementary Fig. 14). During the purification of eS_N_Ar1.3(K39A) as described above (see “Protein production and purification” section), the protein was eluted from the desalting column into NaP_i_ pH 8.0 containing 100 mM EDTA, incubated (25 °C, 800 r.p.m., 3 h) then buffer-exchanged into NaP_i_ pH 8.0 and concentrated using a 10 k MWCO Vivaspin unit (Sartorius). This adjusted purification procedure did not lead to any changes in the rate or enantioselectivity of eS_N_Ar1.3(K39A) (Supplementary Fig. 14). Crystals of eS_N_Ar1.3(K39A) were prepared by mixing 300 nL Morpheus condition C5 (10% *w*/*v* PEG 20000, 20% *v*/*v*, PEG MME 550, 0.03 M of each NPS, 0.1 M MOPS/HEPES-Na pH 7.5) with 300 nL of 20 mg mL^−1^ eS_N_Ar1.3 in 50 mM NaP_i_. All trials were conducted by sitting vapor drop diffusion with incubation at 4 °C. Crystals were flash-cooled in liquid N_2_. When attempting to obtain structures of eS_N_Ar1.3 with bound halide, Br^–^ or I− soaks were performed by adding NaBr or NaI to the drop at a final concentration of 100 mM before freezing. The data were collected from single crystals at Diamond Light Source and subsequently scaled and reduced with Xia2 (ref. ^49^). Preliminary phasing was performed by molecular replacement in Phaser using S_N_Ar1.3 (PDB:9FUG) as a search model. Iterative cycles of rebuilding and refinement were performed in COOT^50^ and Phenix.refine^51^, respectively. The complete data collection and refinement statistics are provided in Supplementary Table 9. Coordinates and structure factors have been deposited in the Protein Data Bank (PDB) under accession number 9SX8.

### Molecular modeling system preparation

The X-ray structures of eS_N_Ar1.3 and S_N_Ar1.3 (PDB codes: 9SX8 and 9FUG, respectively) were used as starting points for running the MD simulations. The binding pose of the nucleophile **1** and aromatic substrate **2** was estimated by Boltz-2^45^. An artificial bond between **1** and **2** was imposed, thus modeling the negatively charged Meisenheimer intermediate. The predicted Boltz-2 pose was then further refined by means of cluster model calculations at the semiempirical GFN2-xtb^46^ level of calculation and removing the artificial bond to generate a starting pose of both nucleophile and electrophile.

The MD parameters for the substrate nucleophile **1** and electrophiles **2** and **5** were generated with the antechamber and parmchk2 modules of AMBER20^52^ using the 2nd generation of the general amber force-field (GAFF2)^52,53^. All substrates were optimized at the B3LYP/6−31G(d) level of theory, including Grimme’s dispersion correction with Becke-Johnson Damping (D3-BJ) and the polarizable conductor model (diethyl ether, *ε* = 4.2) as an estimation of the dielectric permittivity in the enzyme active site^54^. The partial charges (RESP model)^55^ were set to fit the electrostatic potential generated at the HF/6-31G(d) level of theory. The charges were calculated according to the Merz-Singh-Kollman^56^ scheme using the Gaussian16 software package^57^. The protonation states were predicted using PROPKA^58,59^. The enzyme structures were solvated in a pre-equilibrated truncated octahedral box of 10 Å edge distance using the OPC water model and neutralized by the addition of explicit counterions (i.e., Na^+^) using the AMBER20 leap module. All MD simulations were performed using a modification of the Amber99 force field (ff19SB)^60^.

### Molecular dynamics simulations of S_N_Arase variants

MD equilibration phase was done following the protocol described by Roe and Brooks with small differences fine-tuned to our systems^61^. The bonds involving hydrogen are constrained by the SHAKE algorithm during the non-minimization steps. Long-range electrostatic effects were modeled using the particle mesh-Ewald method^62^. For Lennard–Jones and electrostatic interactions, a 10 Å cut-off was applied. The MD protocol starts with the minimization phase of 1500 steps of the steepest descent method, followed by 3500 steps of the conjugate gradient method with a positional restraint (i.e., a force constant of 5.0 kcal mol^−1^ Å^−2^) to the protein heavy atoms. In the following heating phase, a temperature increment from 25 K to 300 K during 20 ps of MD simulation time, a Langevin thermostat with a collision frequency of 5 ps^−1^, and a positional restraint (i.e., a force constant of 5.0 kcal mol^−1^ Å^−2^) to the protein heavy atoms are performed. A minimization and heating of all atoms in the system is the following step. This starts with two minimization stages of 1000 steps of the steepest descent method, followed by 1500 steps of the conjugate gradient method, each with a positional restraint (i.e., force constant of 2.0 kcal mol^−1^ Å^−2^ in the first minimization and 0.1 kcal mol^−1^ Å^−2^ in the second) to the protein heavy atoms. Following, a third minimization phase of 1500 steps of the steepest descent method followed by 3500 steps of the conjugate gradient method without any positional restraint is performed. The system is then heated in accordance with the previously established procedure. Finally, a five-round equilibration phase at the NPT ensemble with a constant pressure of 1 atm is performed. The first four rounds were done with the Berendsen barostat, whereas the fifth one was done with a Monte-Carlo barostat. For all equilibration rounds, a Langevin thermostat with a collision frequency of 1 ps^−1^ was used. A positional restraint to the protein-heavy atoms with a force constant of 1.0 and 0.5 kcal mol^−1^ Å^−2^ was applied to the first and second equilibration rounds, respectively. In the third round of 10 ps equilibration, a positional restraint to the backbone-heavy atoms with a force constant of 0.5 kcal mol^−1^ Å^−2^ was used. The fourth and fifth equilibrations of 10 ps and 1 ns, respectively, were performed without any restraint. The production runs were performed at the NVT ensemble with the Langevin thermostat with a collision frequency of 1 ps^−1^ during 100 ns for all systems. A total of 10 replicas of equilibration and production runs were performed, reaching a total simulation time of 1 μs/system (10 replicas × 100 ns) for eS_N_Ar1.3 in the presence of **1** and **2**, eS_N_Ar1.3 in the presence of **1** and **5**, and S_N_Ar1.3 in the presence of **1** and **2**. The most representative structure of each system was extracted, being the initial conformation for new MD simulations. This procedure is done to discriminate and study the binding poses sampled during the MD simulations. A total of 10 replicas of equilibration and production runs were performed again following the exact same protocol, reaching a total simulation time of 1 μs/system (10 replicas × 100 ns). The MD trajectories were analyzed using the Python packages MDTraj^63^, pytraj^64^, which is part of the cpptraj package^61^, MDAnalysis^65^, and PyEMMA^66^.

### Conformational landscape reconstruction

MD simulations allow the sampling of the population distribution of biomolecules by integrating Newton’s laws of motion. This process enables the recovery of thermodynamic properties such as the free energy. However, due to the vast number of atoms involved in the MD simulations, this probability distribution of molecular states is represented in an extremely high-dimensional space. This is usually solved by focusing on a selected set of degrees of freedom (DOF) relevant to the process of interest. In our case we used the dihedral angle between the nitrogen atom of the *ortho*-nitro substituent (electrophile), the halocarbon (electrophile), the halogen atom (electrophile) and the chiral carbon in the nucleophile (*y*-axis in Fig. 5, dihedral shown in Supplementary Fig. 25), and the catalytic distance between the halocarbon in the electrophile and the chiral carbon atom in the nucleophile (*x*-axis in Fig. 5). High dimensional data obtained from MD simulations can be projected onto these DOFs for obtaining the probability distributions and reconstructing the conformational landscape (free energy, Eq. 2).

2 $${{{\rm{G}}}} \sim -{k}_{B}{{{\rm{T}}}}\,\log ({{{\rm{P}}}})$$

where the free energy (*G*) is defined as the negative logarithm of the population distribution (*P*) in *k*_*B*_*T* units (e.g., kcal/mol K). A maximum in the distribution corresponds to a minimum in the free energy landscape.

### QM/MM calculations

QM/MM calculations were performed using the ONIOM method starting from eS_N_Ar1.3 X-ray and MD-derived structures. Input files were prepared with MolUP, and all geometry optimizations and energy calculations were carried out with Gaussian16 (*Gaussian 16 Rev. C.01*; Wallingford, CT, 2016). The QM region comprised substrates **1** and **2**, together with residues His23 and Arg124 (56 atoms and 3 H-link atoms). The remaining protein environment was described at the MM level using the AMBER ff14SB force field. Residues and water molecules within 10 Å of the QM region were included in the active region.

Geometry optimizations were performed using mechanical embedding. The QM region was treated at the B3LYP-GD3/6-31G(d) level during geometry optimizations. Solvation effects were included using the SMD continuum solvation model. Single-point energy refinements were performed on the optimized geometries at the B3LYP-GD3/6–311+G(2d,2p) level. Transition states were confirmed by frequency analysis. The corresponding reactant-complex and product structures were identified and confirmed through intrinsic reaction coordinate calculations.

### Reporting summary

Further information on research design is available in the Nature Portfolio Reporting Summary linked to this article.

## Supplementary information

Supplementary Information

Reporting Summary

Transparent Peer Review file

## Source data

Source data

## Data Availability

The data generated in this study are provided within the paper and in the Supplementary Information. Source data are provided with this paper. Coordinates and structure factors have been deposited in the Protein Data Bank under accession number 9SX8 (https://www.rcsb.org/structure/9SX8). Crystallographic data for **(*****R*****)-25** has been deposited with the CCDC number 2494693. These data can be obtained free of charge from The Cambridge Crystallographic Data Centre via www.ccdc.cam.ac.uk/data_request/cif. Data supporting the findings of the study are available from the corresponding author(s) upon request. All the computational data obtained from the single-point calculations have been uploaded onto the ioChem-BD platform (https://iochem.udg.edu/browse/handle/100/7772). Source data are provided with this paper.
