# Supplementary material for: Directed evolution of an enantiocomplementary SNArase reveals divergent catalytic features
Source: Nat Commun. 2026 Aug 21;17:10033. doi: 10.1038/s41467-026-76922-5 (PMC13594109; doi:10.1038/s41467-026-76922-5)
Supplement: Supplementary file 1 [file 41467_2026_76922_MOESM1_ESM.pdf]

Supplementary Information

Directed Evolution of an Enantiocomplementary S_NArase Reveals Divergent Catalytic Features

Thomas M. Lister, George W. Roberts, Cristina Duran, Guillem Casadevall, Fei Zhao, Alexander A. V. Millman, Igor Larrosa & Sílvia Osuna*, Anthony P. Green*

Table of Contents

Supplementary Figures 5

Supplementary Figure 1: Effect of buffer composition on activity and selectivity for eS$_N$Ar1.2. 5

Supplementary Figure 2: Directed evolution of an efficient and enantiocomplementary S$_N$Ar enzyme. 6

Supplementary Figure 3: Comparison of the evolutionary trajectories of the enantiodivergent S$_N$Arases S$_N$Ar1.3 and eS$_N$Ar1.3. 7

Supplementary Figure 4: Comparison of the melting temperatures (T_m) for the variants across evolution to eS$_N$Ar1.3. 8

Supplementary Figure 5: Comparison of initial rates between eS$_N$Ar1.0 and eS$_N$Ar1.3 shows a ca. 480-fold rate increase under the reaction conditions used during enzyme engineering. 9

Supplementary Figure 6: Total turnovers achieved by eS$_N$Ar1.3. 10

Supplementary Figure 7: Monitoring the degradation of nucleophile 1 by ^{1}H NMR 11

Supplementary Figure 8: The degradation of nucleophile 1 is proposed to occur *via* a hydrolysis pathway 12

Supplementary Figure 9: Kinetic characterization of eS$_N$Ar1.0 and eS$_N$Ar1.3. 13

Supplementary Figure 10: ^{1}H NMR data comparing a chemically synthesised authentic standard of rac-3 with (*S*)-3 produced in a preparative scale biotransformation with eS$_N$Ar1.3. 14

Supplementary Figure 11: Chiral HPLC data comparing a chemically synthesised authentic standard of rac-3 with (*S*)-3 produced in a preparative scale biotransformation with eS$_N$Ar1.3. 15

Supplementary Figure 12: High activity and selectivity are maintained with eS$_N$Ar1.3 when using equimolar quantities of nucleophile 1 and chloride-containing electrophile 2. 16

Supplementary Figure 13: Kinetic characterization of eS$_N$Ar1.3 with different 2,4-dinitrohalobenzene electrophiles. 17

Supplementary Figure 14: The effect of leaving group on the activity and selectivity for the 4-halo-3- nitrobenzonitrile electrophile 18

Supplementary Figure 15: Product 14a undergoes non-enzymatic decarboxylation under conditions used in biotransformations. 19

Supplementary Figure 16: ^{1}H NMR data comparing a chemically synthesised authentic standard of rac-25 with (*R*)-25 produced in a preparative scale biotransformation with S$_N$Ar$_{Ph}$1.0. 20

Supplementary Figure 17: Chiral HPLC data comparing a chemically synthesised authentic standard of rac-25 with (*R*)-25 produced in a preparative scale biotransformation with eS$_N$Ar1.3. 21

Supplementary Figure 18: The effect of the K39A point mutation in eS$_N$Ar1.3 and incubation of this variant with different concentrations of EDTA during the protein purification procedure on the initial rate and enantioselectivity. 22

Supplementary Figure 19: Comparison of the electrophile binding modes in S$_N$Ar1.3 and eS$_N$Ar1.3. 23

Supplementary Figure 20: Polar interactions between Asp9 and key catalytic Arg124 in an X-ray crystal structure of eS$_N$Ar1.3(K39A) that emerged during evolution. 24

Supplementary Figure 21: The effect of mutating eS$_N$Ar1.3 active site residues on initial rate and enantioselectivity. 25

Supplementary Figure 22: The effect of the eS$_N$Ar1.3 point mutations on the reaction rate with electrophile substrate 2. 26

Supplementary Figure 23: Relationship between eS$_N$Ar1.3 initial rate and concentration of chloride, bromide and iodide in the reaction. 27

Supplementary Figure 24: eS_NAr1.3 predicted structure using Boltz-2 and optimized at GFN2-xtb level. 28

Supplementary Figure 25: Graphical definition of the dihedral angle and catalytic distance used for reconstructing the conformational landscapes. 29

Supplementary Figure 26: Conformational landscapes of eS_NAr1.3 and the single mutants H23A and R124A in the presence of nucleophile 1 and aromatic substrate 2. 30

Supplementary Figure 27: Optimized Reactant-complex (RC), Transition-State (TS), and Product (Prod) structures obtained from QM/MM calculations.. 31

Supplementary Figure 28: Overlay of the optimized reactant-complex (RC), transition-state (TS), and product (Prod) structures obtained from QM/MM calculations.. 32

Supplementary Figure 29: QM/MM mechanistic analysis of the alternative Arg124 conformation. 33

Supplementary Figure 30: Arg124 flexibility in related X-ray structures.. 34

Supplementary Figure 31: Overlay of eS_NAr1.3 and S_NAr1.3 representative structures extracted from MD simulations. 35

Supplementary Tables 36

Supplementary Table 1: Electrophile scope of eS_NAr1.3 36

Supplementary Table 2: Nucleophile scope of eS_NAr1.3 37

Supplementary Table 3: Extinction coefficients of electrophiles and products 38

Supplementary Table 4: Reverse-phase UPLC analysis methods 39

Supplementary Table 5: Normal-phase chiral HPLC analysis methods 40

Supplementary Table 6: Calculated and experimental masses of apo enzymes 41

Supplementary Table 7: Primer sequences used to generate DNA libraries 42

Supplementary Table 8: Primer sequences used to point mutant variants 48

Supplementary Table 9: Crystallographic data for compound (*R*)-25 (CDCC 2494693) 49

Supplementary Table 10: Data collection and refinement statistics. Entries in parentheses refer to statistics in the highest resolution bin. 50

DNA and protein sequences for eS_NAr1.3 51

Preparative Scale Biotransformations 52

Chemical synthesis 54

NMR spectra 65

Chiral HPLC chromatograms 79

References 99

Supplementary Figures

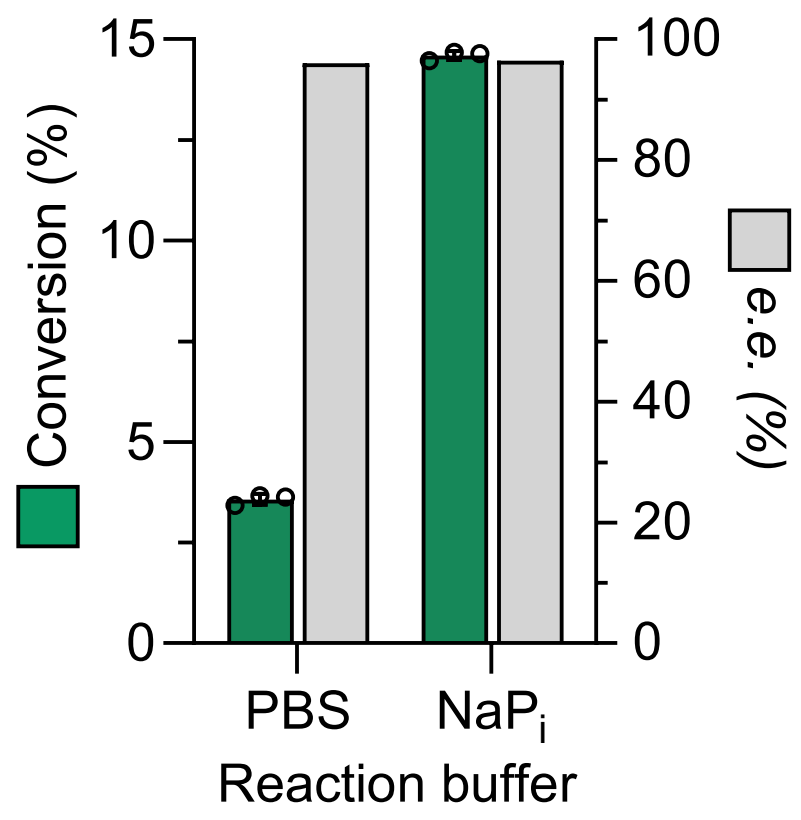

Supplementary Figure 1: Effect of buffer composition on activity and selectivity for $eS_NAr1.2$. Bar chart showing the effect of buffer, specifically the presence of exogenous chloride, on the conversion (green bars) and enantioselectivity (grey bars) achieved with $eS_NAr1.2$. Reaction conditions: **1** (25 mM), **2** (2.5 mM), $eS_NAr1.2$ (2.5 μM) in PBS or NaP_i pH 8.0 with 10% *v*/*v* DMSO as a co-solvent, 16 h at 30 °C. Data are a mean ± S.D. of measurements made in triplicate.

Round	Description	Clones Screened	Beneficial Mutations	Best variant
1	**Saturation mutagenesis** of active site positions and flexible loop regions: S9, L10, I14, V22, I26, P63, L64, L68, E69, M72, L87, S91, A95, L122, R124, P128, A131, F132, R65, L92, Q96, D125, A129, L133	2112	E69P, W88L, W88V, W88Y, S91T, S91P	**eS$_N$Ar1.1** = eS$_N$Ar1.0 + W88L
2	**Saturation mutagenesis** of active site positions and flexible loop regions: S9, L10, Y20, V22, M27, L42, Y45, E46, A49, P63, E69, M72, G80, L87, S91, L92, A95, G99, L122, R124, P128, A129, A131, F132, S148	2200	S9E, Y20R, E46Y, E46S, E46R, E46A, P63S	**eS$_N$Ar1.2** = S$_N$Ar1.1 + S9E_Y20R_E46T
3	**Saturation mutagenesis** of active site positions and flexible loop regions: E9, L10, G11, T12, L13, I14, G18, L19, V22, H23, I126, M27, L42, A49, P60, P63, R65, D66, L87, L88, S91, L92, A95, V120, L122, R124, P128, A129, S148	1584	E9D, L10Q, L13F, G18K, R65V, D66E, S91A, S148F, S148H	**eS$_N$Ar1.3** = eS$_N$Ar1.2 + E9D_R65V

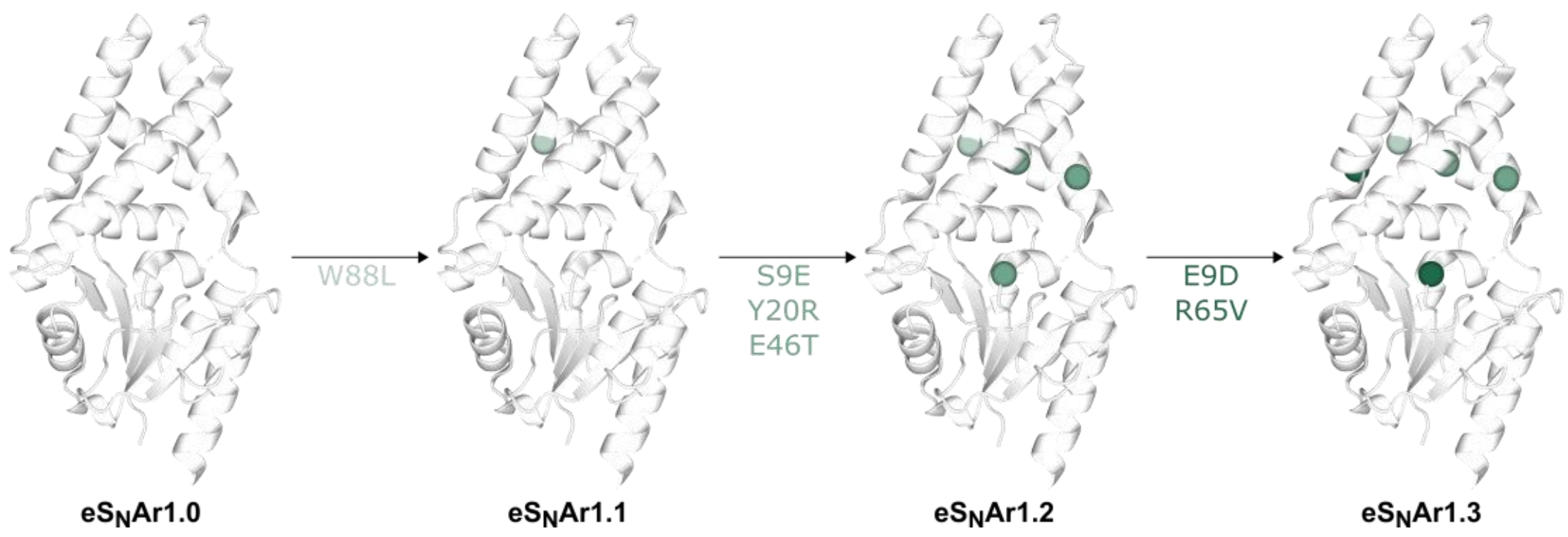

Supplementary Figure 2: Directed evolution of an efficient and enantiocomplementary S$_N$Ar enzyme. Schematic showing the trajectory from S$_N$Ar1.0 to eS$_N$Ar1.3. Mutations introduced are represented as CPK spheres at the C_β. Three rounds of evolution from eS$_N$Ar1.0 afforded eS$_N$Ar1.3, which contains six mutations. Library generation method, positions targeted, the number of clones evaluated, beneficial mutations and the most improved variant for each round are given in the table.

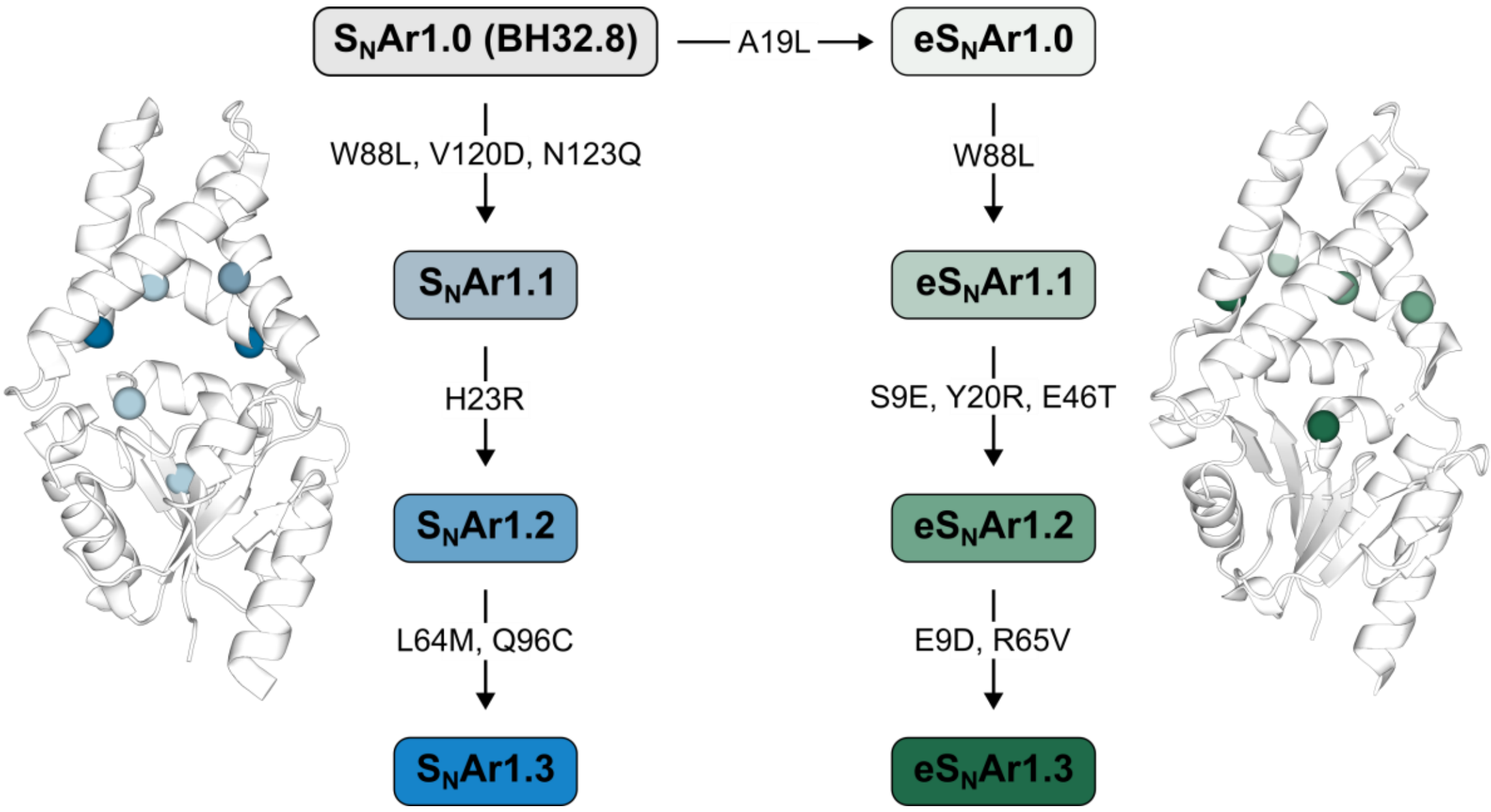

Supplementary Figure 3: Comparison of the evolutionary trajectories for the enantiodivergent $S_NAr$ases $S_NAr1.3$ and e$S_NAr1.3$. Figure showing the mutations installed during each round of the evolution towards our previous $S_NAr$ase ($S_NAr1.3$, left) and the enantiodivergent $S_NAr$ase of this study (e$S_NAr1.3$, right). Mutations are represented as spheres at the C_α and coloured according to their order of introduction.

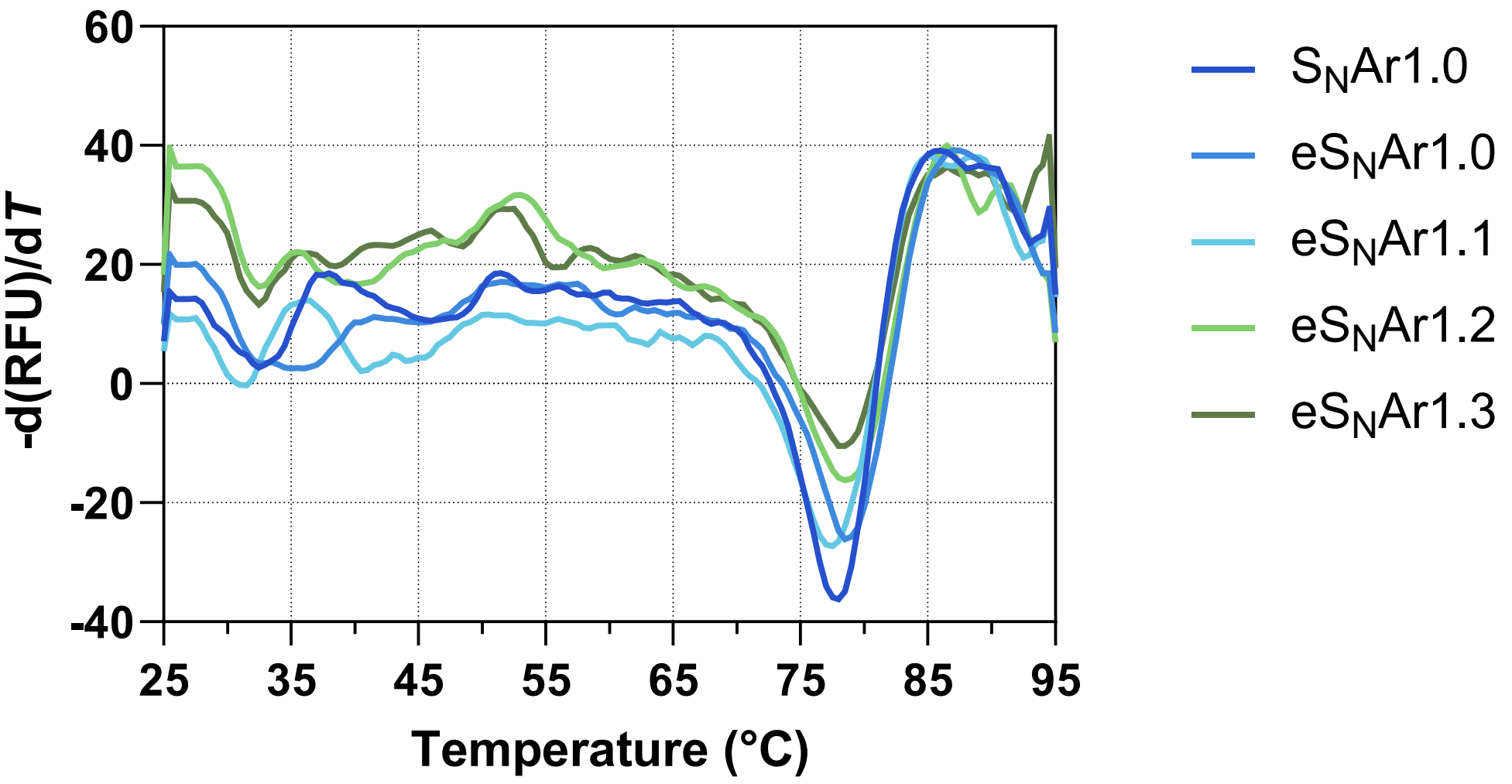

Supplementary Figure 4: Comparison of the melting temperatures (T_m) for the variants across evolution to eS$_N$Ar1.3. Plot of the negative derivative of RFU (relative fluorescence units) as a function of temperature (dT), versus the temperature (°C) for S$_N$Ar1.0 and eS$_N$Arase variants eS$_N$Ar1.0 to eS$_N$Ar1.3. A higher T_m indicates improved thermal stability and is calculated as the lowest point of the negative derivative.

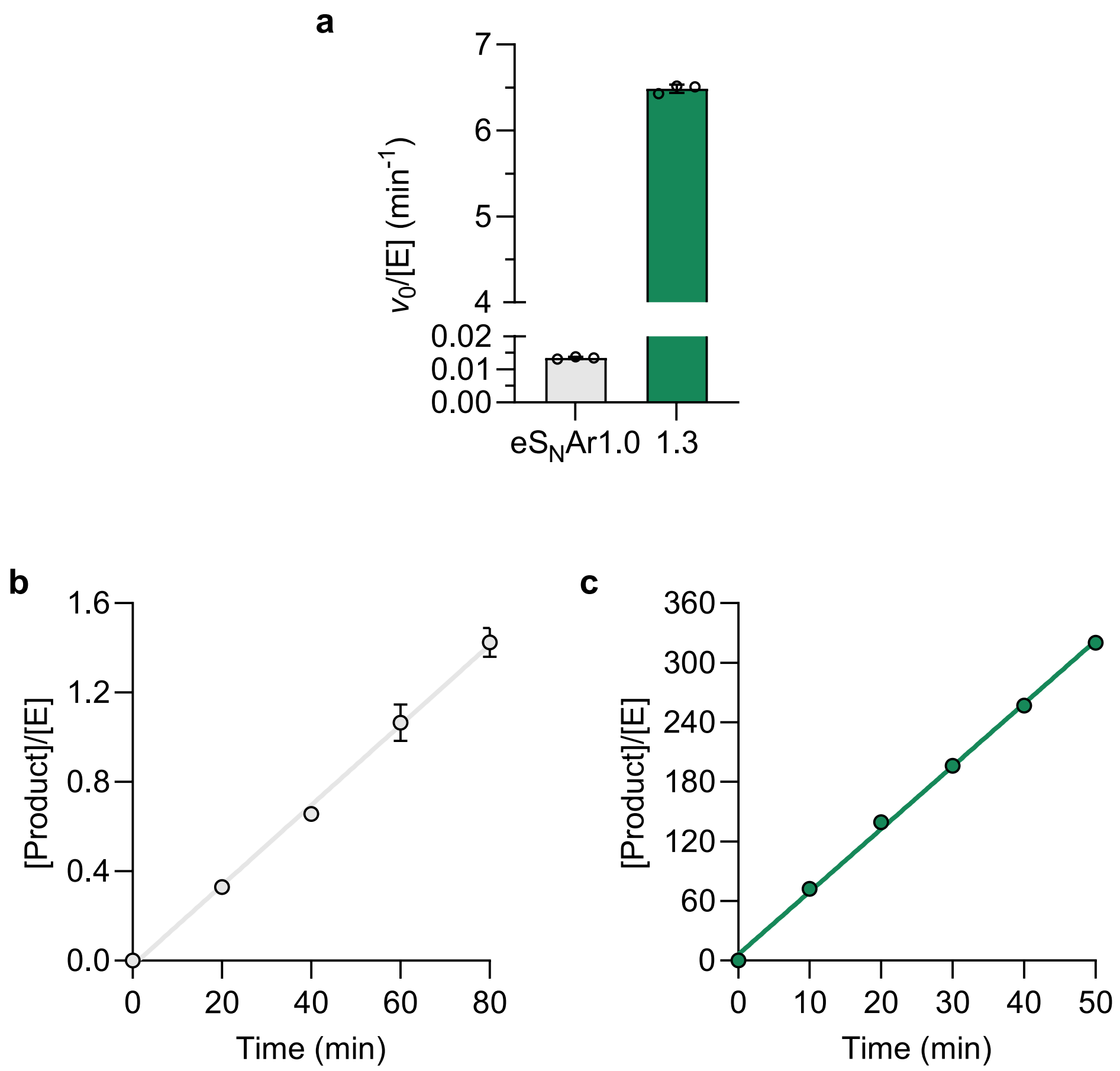

Supplementary Figure 5: Comparison of initial rates between eS$_N$Ar1.0 and eS$_N$Ar1.3 shows a ca. 480-fold rate increase under the reaction conditions used during enzyme engineering. a, Bar chart comparison of the initial rates for eS$_N$Ar1.0 and eS$_N$Ar1.3. **b** & **c**, Line graphs of [**3**]/[E] against time for point eS$_N$Ar1.0 and eS$_N$Ar1.3, respectively. Data were fitted using Linear Regression equation in GraphPad Prism. Reaction conditions: **1** (75 mM), **5** (1.0 mM) and eS$_N$Ar1.0 (70 µM) or eS$_N$Ar1.3 (1 µM) in NaP$_i$ pH 8.0 with 10% *v*/*v* DMSO at 30 °C. Error bars represent the standard deviation of measurements made in triplicate.

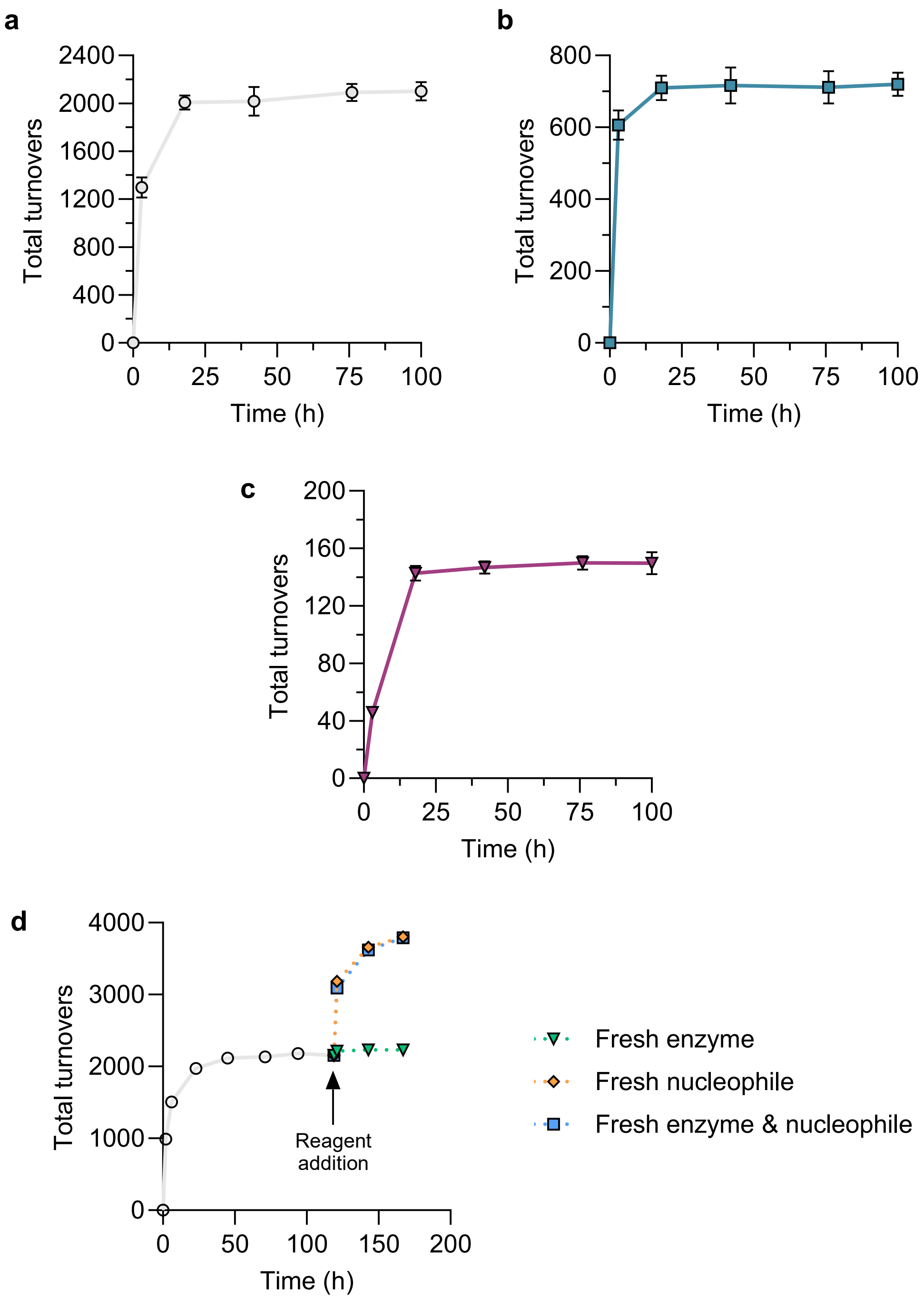

Supplementary Figure 6: Total turnovers achieved by eS$_N$Ar1.3. Line graphs showing the time course to the determine the total turnover number of eS$_N$Ar1.3 with chloride, bromide and iodide leaving groups (**a**, **b** and **c** respectively) for the 2,4-dinitrohalobenzene electrophile series in addition to the basis of turnover limitation with 2,4-dinitrochlorobenzene (**d**). Reaction conditions: **1** (10 equiv), electrophile (2, 2.5 mM; 4, 1.5 mM; 5, 1.0 mM), eS$_N$Ar1.3 (0.001 mol% using **2**; 0.01 mol% using **4**; 0.05 mol% using **5**) in NaP$_i$ pH 8.0 with 10% *v*/*v* DMSO at 30 °C. Error bars represent the standard deviation of measurements made in triplicate.

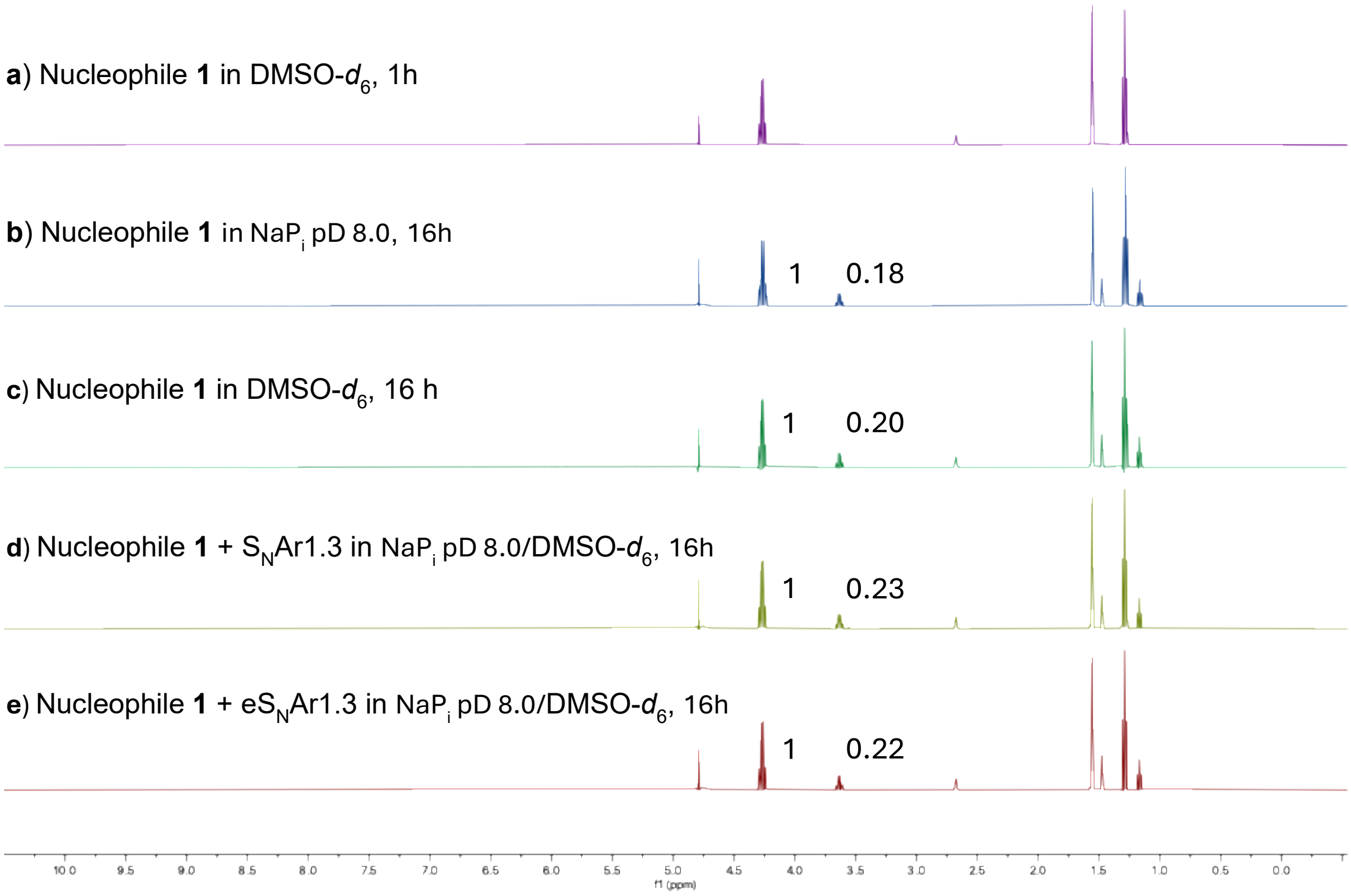

Supplementary Figure 7: Monitoring the degradation of nucleophile 1 by ^{1}H NMR. ^{1}H NMR data (400 MHz) for nucleophile **1** in the presence and absence of S_NArases which demonstrates that the degradation is non-enzymatic. The integrals shown are the ratio of the C$\underline{H_2}$ signals of the starting material (4.25 ppm) and the hydrolysis, degradation product (3.65 ppm). Reaction conditions: **1** (25 mM), S_NArase (10 µM if used) in 50 mM NaP_i pD 8.0 with 10% v/v DMSO-d_6 (if used) at 30 °C.

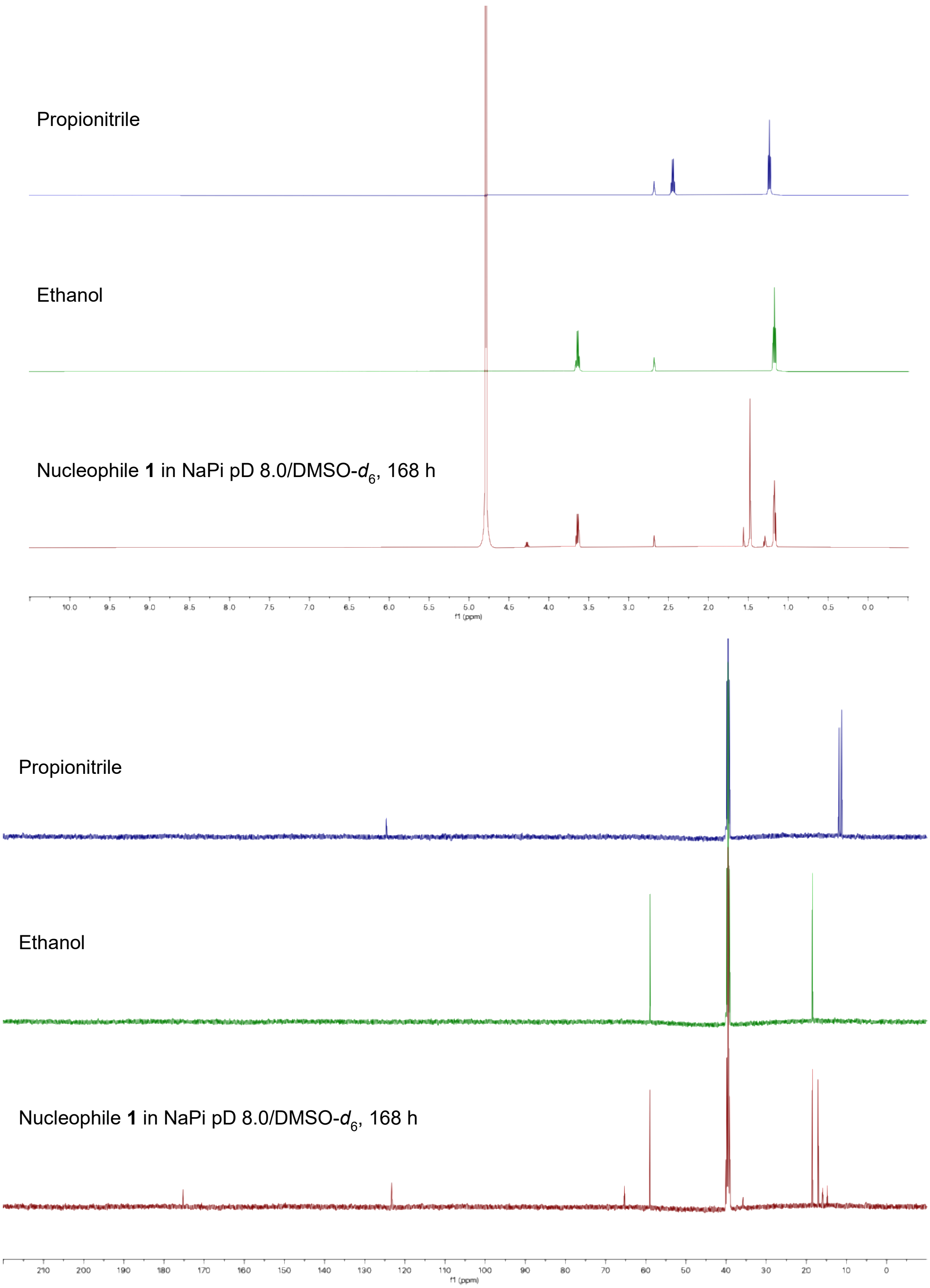

Supplementary Figure 8: The degradation of nucleophile 1 is proposed to occur *via* a hydrolysis pathway. Comparison of the ^{1}H (500 MHz) and ^{13}C (126 MHz) NMR data (top and bottom figures, respectively) for propionitrile, ethanol and the reaction of nucleophile **1** in NaP_i pD 8.0/DMSO-d_6 suggests that the nucleophile degrades *via* solely a hydrolysis pathway and does not involve decarboxylation. Reaction conditions: **1** (25 mM) in 50 mM NaP_i pD 8.0 with 10% v/v DMSO-d_6 at 30 °C for 168 h.

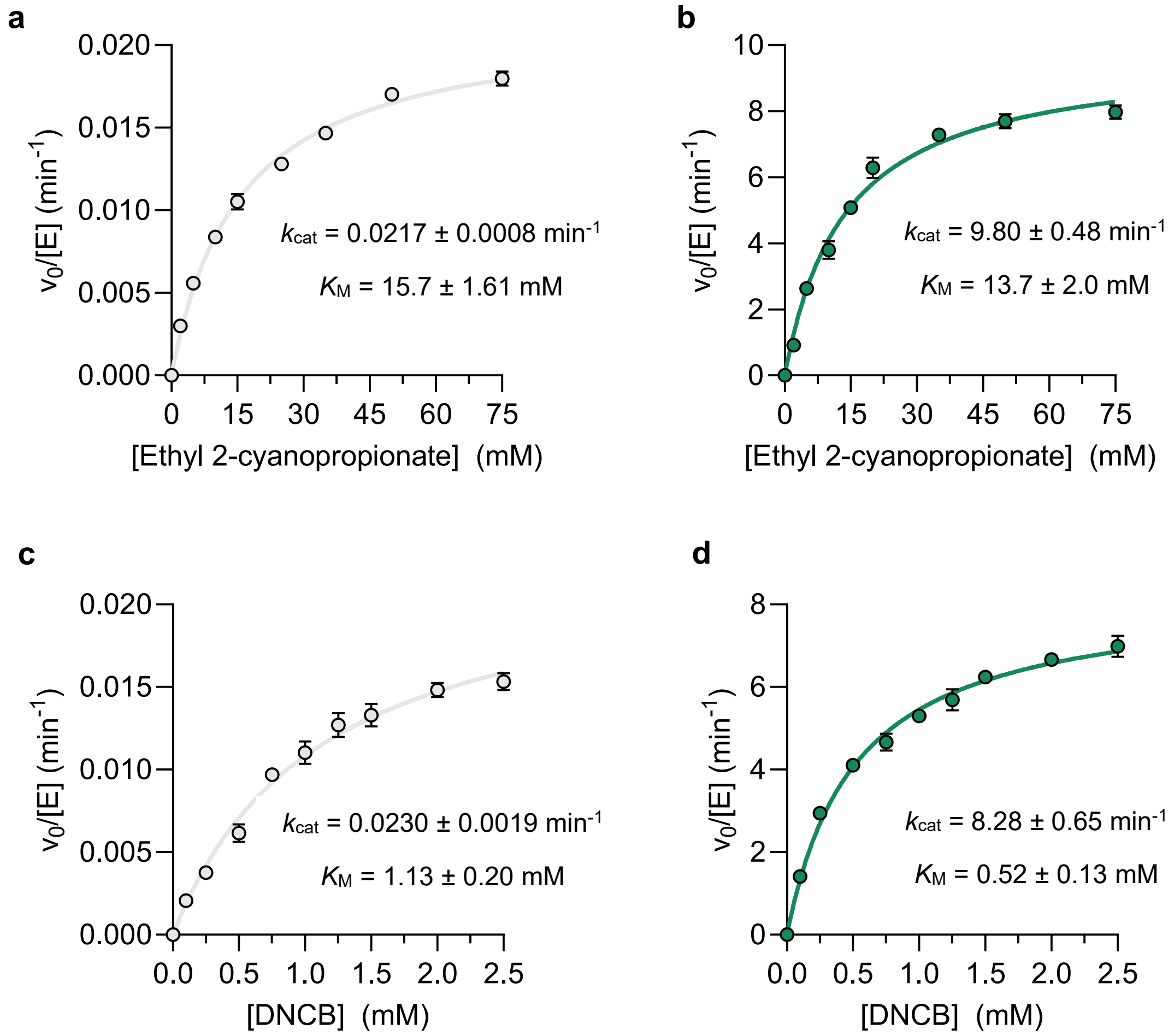

Supplementary Figure 9: Kinetic characterization of eS_NAr1.0 and eS_NAr1.3. Michaelis–Menten plots for the S_NAr reaction between **1** and **2** catalysed by either eS_NAr1.0 (**a** and **c**) or eS_NAr1.3 (**b** and **d**). Assays were performed at a fixed concentration of **2** (2.5 mM) and varying concentrations of **1** (**a** and **c**), or a fixed concentration **1** (75 mM) and varying concentrations of **2** (**b** and **d**). The plots show the averaged initial rates that were fitted to the Michaelis–Menten equation using GraphPad Prism software. Error bars represent the standard deviation of measurements made in triplicate.

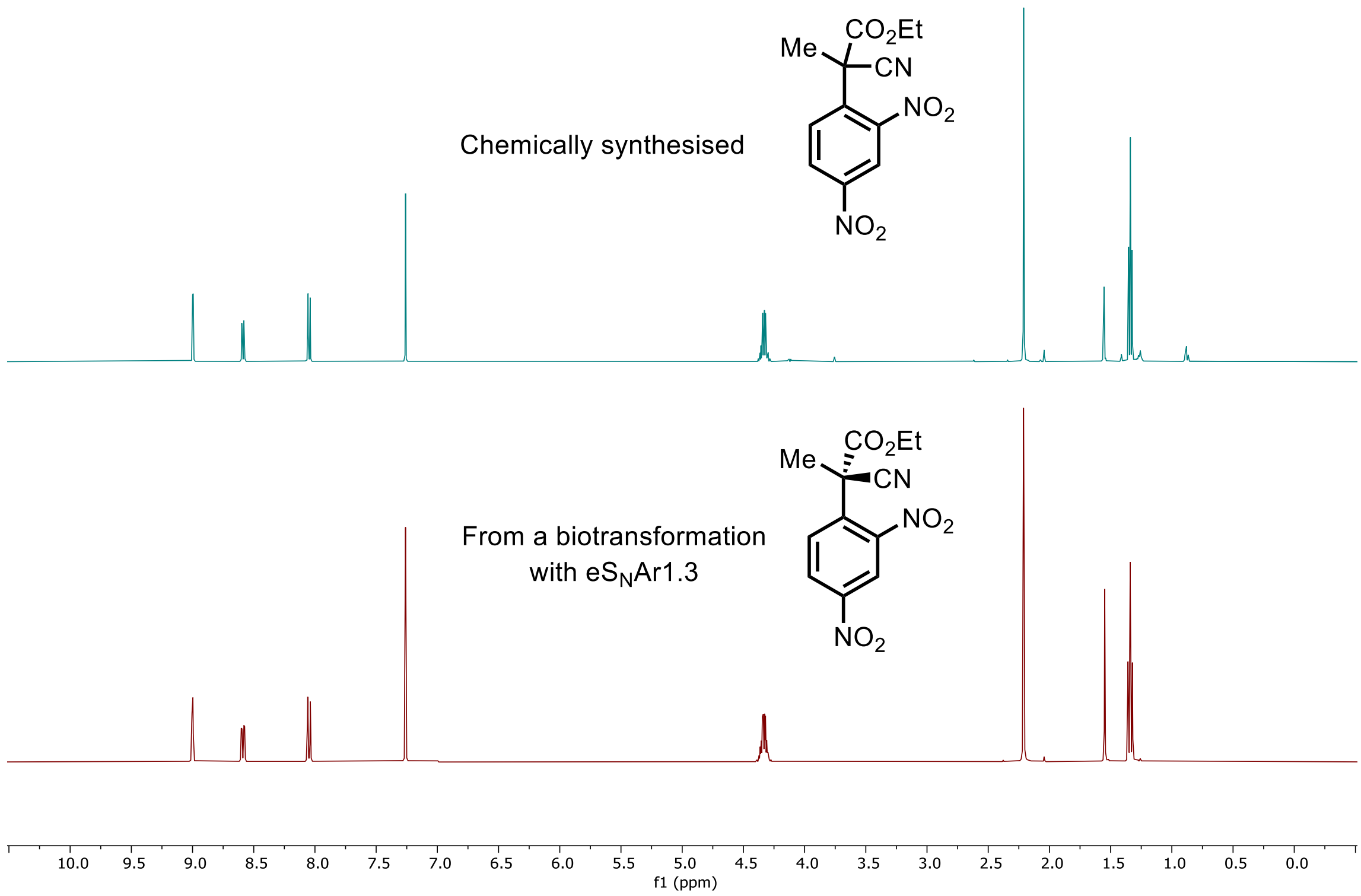

Supplementary Figure 10: ^{1}H NMR data comparing a chemically synthesised authentic standard of rac-3 with (*S*)-3 produced in a preparative scale biotransformation with eS$_N$Ar1.3. Top: ^{1}H NMR data (500 MHz, $CDCl_3$) for the synthetic standard of **rac-3**. Bottom: ^{1}H NMR data (400 MHz, $CDCl_3$) for **(*S*)-3** produced in a preparative-scale biotransformation using eS$_N$Ar1.3.

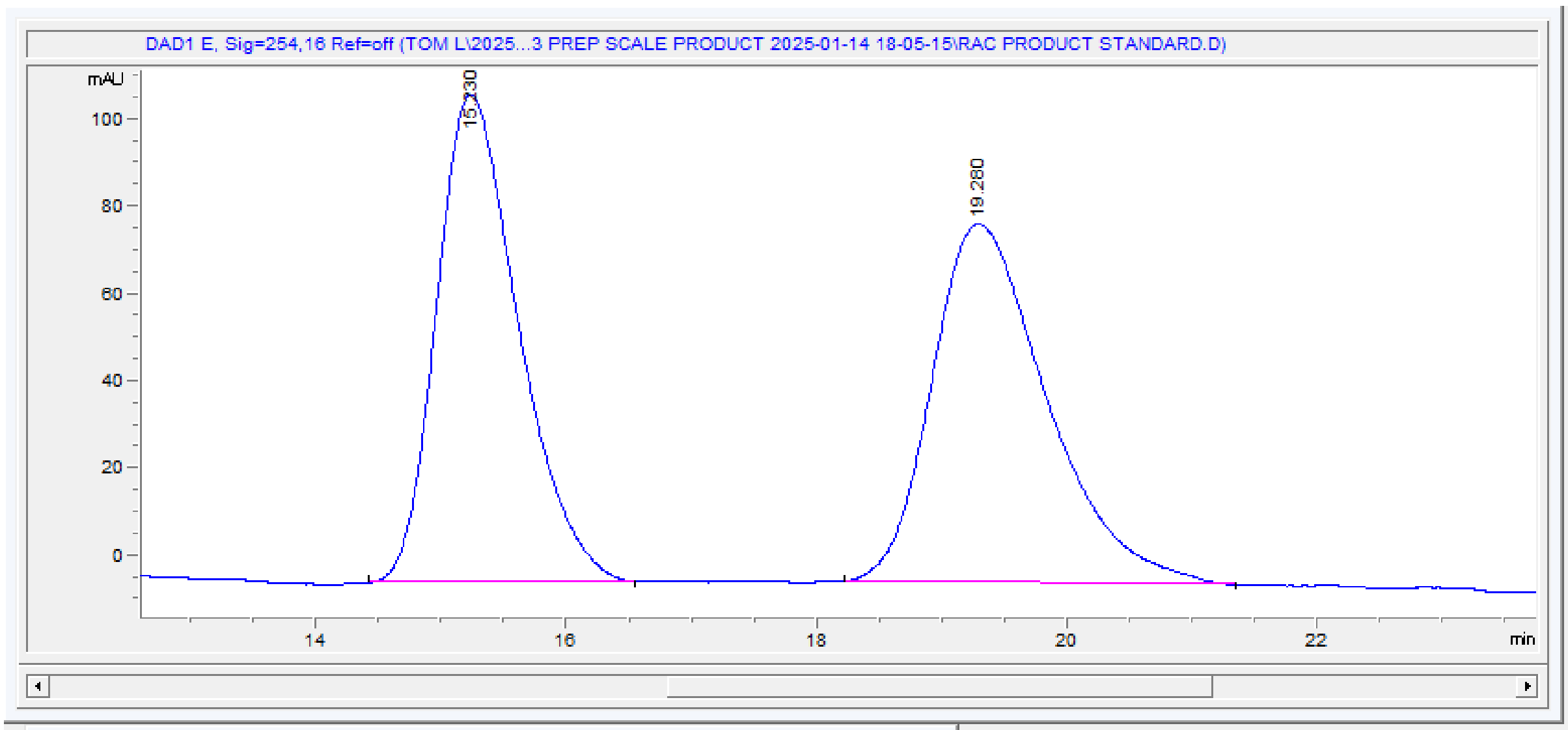

#	Time	Area	Height	Width	Area%	Symmetry
1	15.23	5092.5	111.9	0.655	49.215	0.685
2	19.28	5254.9	82.2	0.8042	50.785	0.605

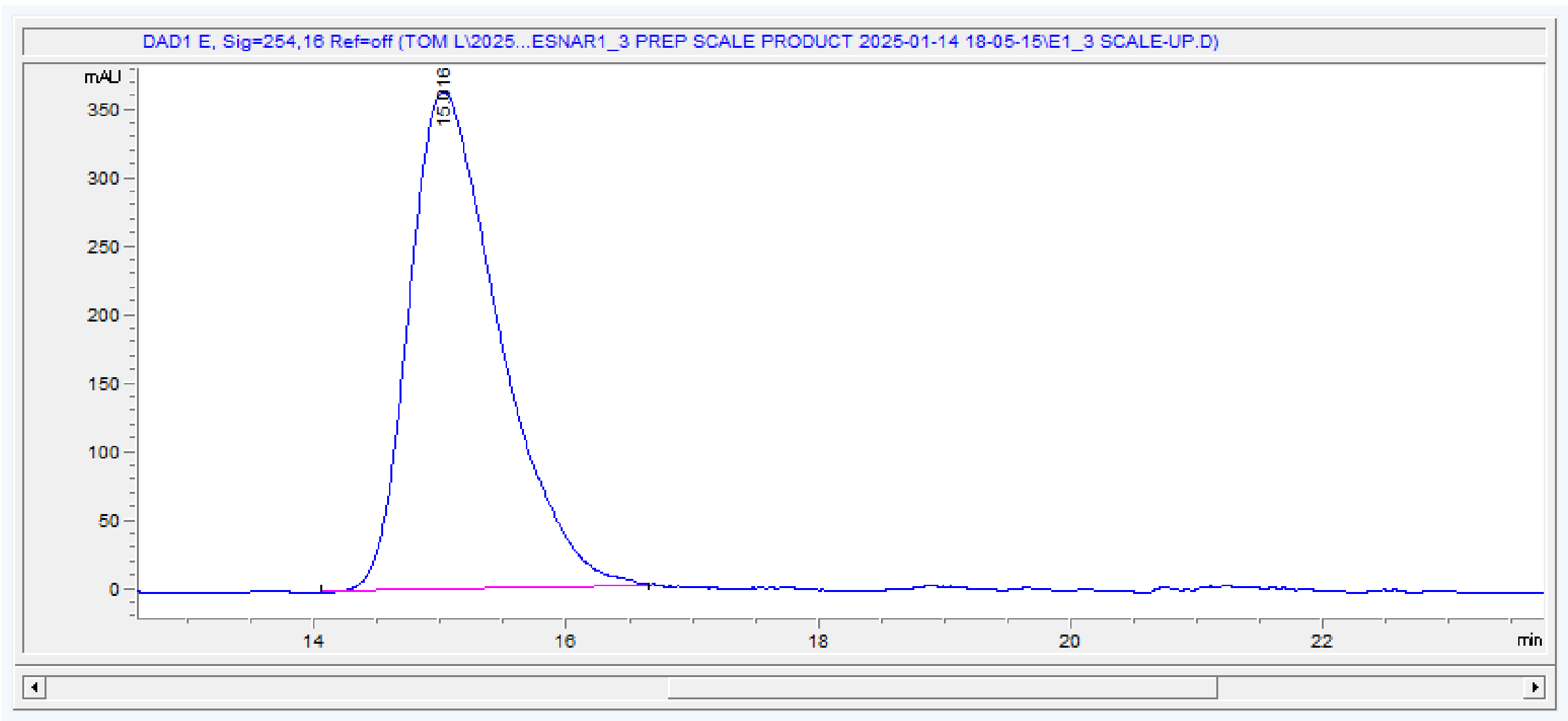

#	Time	Area	Height	Width	Area%	Symmetry
1	15.016	18250.9	363.9	0.7171	100.000	0.584

Supplementary Figure 11: Chiral HPLC data comparing a chemically synthesised authentic standard of rac-3 with (*S*)-3 produced in a preparative scale biotransformation with eS$_N$Ar1.3. Top: Chiral HPLC trace for the synthetic standard of **rac-3**. Bottom: Chiral HPLC trace for enantiopure **(*S*)-3** produced in a preparative-scale biotransformation using eS$_N$Ar1.3.

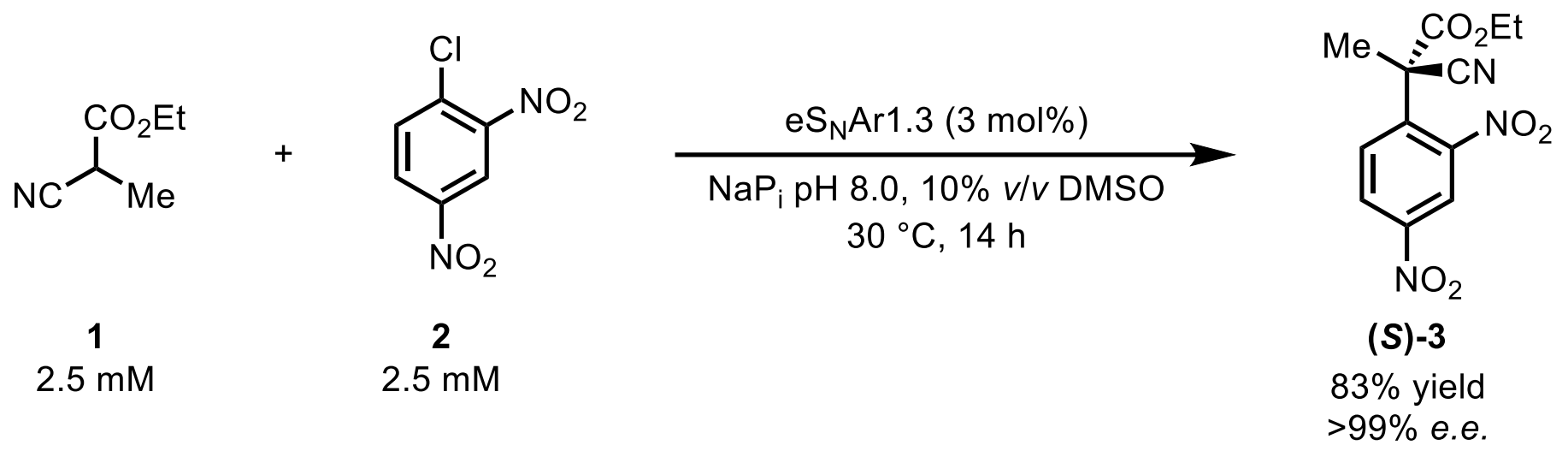

Supplementary Figure 12: High activity and selectivity are maintained with eS_NAr1.3 when using equimolar quantities of nucleophile 1 and chloride-containing electrophile 2. Chemical scheme showing the enantiodivergent synthesis of ***(S)*-3** using eS$_N$Ar1.3, from 1 equivalent of nucleophile **1** and 1 equivalent of nucleophile **2**.

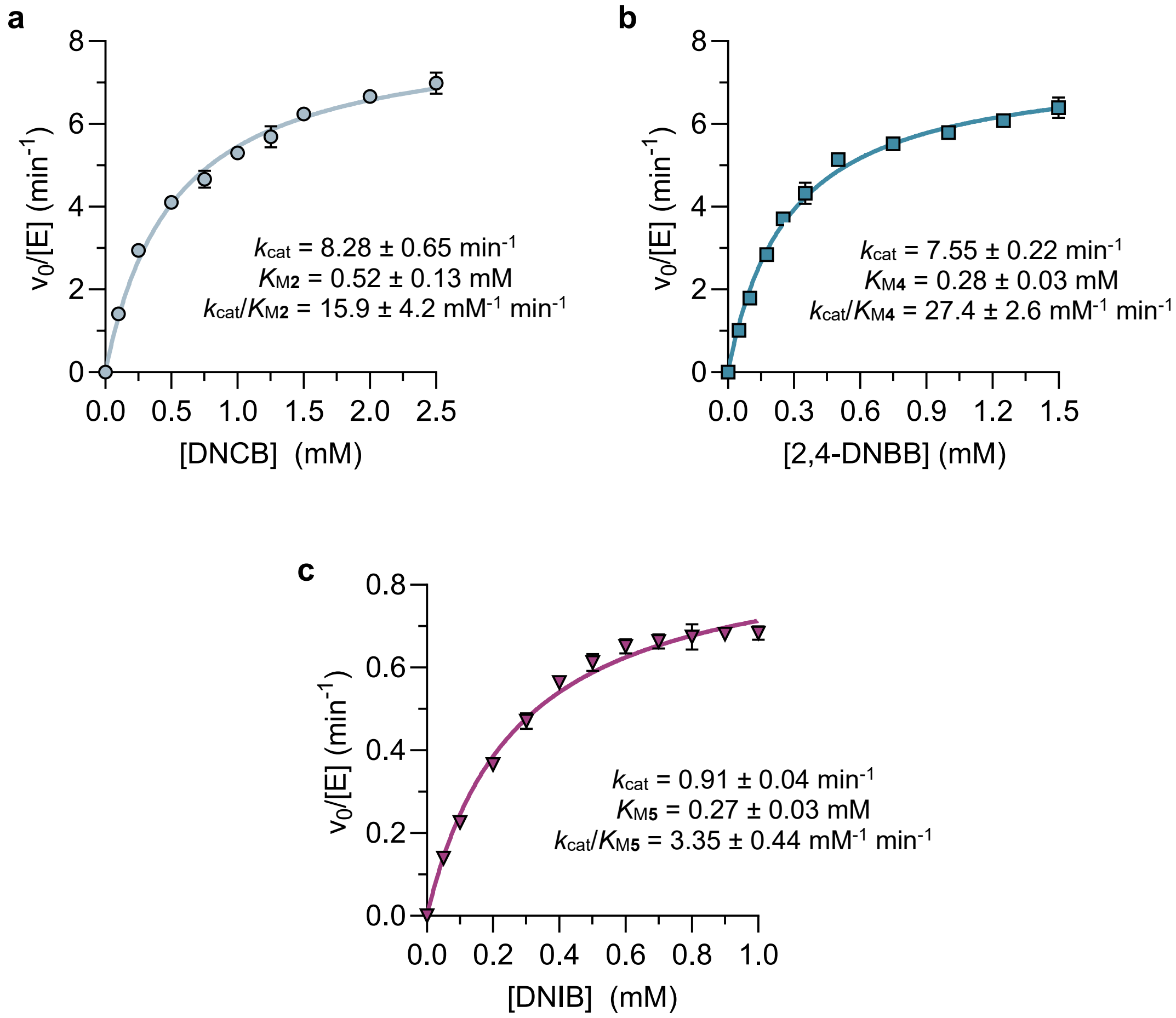

Supplementary Figure 13: Kinetic characterization of eS$_N$Ar1.3 with different 2,4-dinitrohalobenzene electrophiles. Michaelis–Menten plots for the S$_N$Ar reaction between **1** and either **2**, **4** or **5** (**a**, **b** and **c** respectively). Assays were performed at a fixed concentration of **1** (75 mM) and varying concentrations of **2**, **4** or **5**. The plots show the averaged initial rates that were fitted to the Michaelis–Menten equation using GraphPad Prism software. Error bars represent the standard deviation of measurements made in triplicate.

c

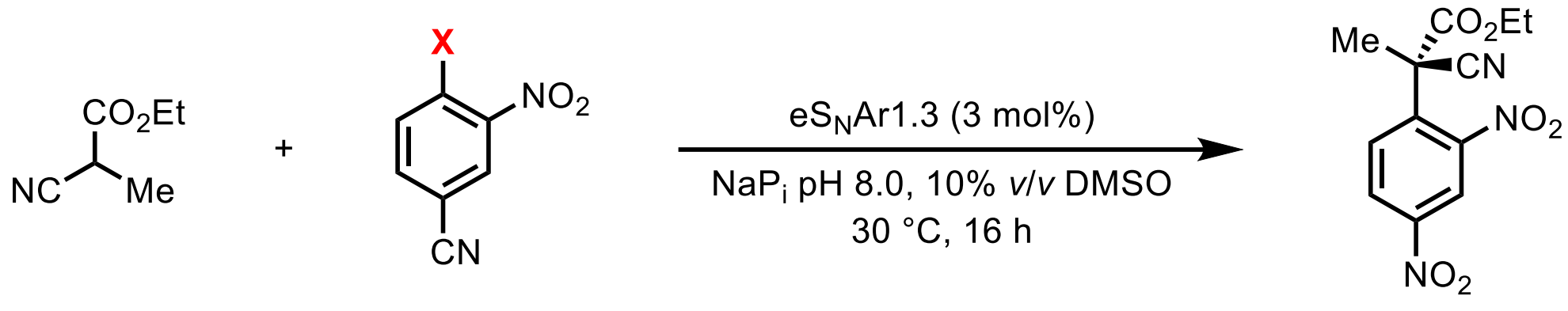

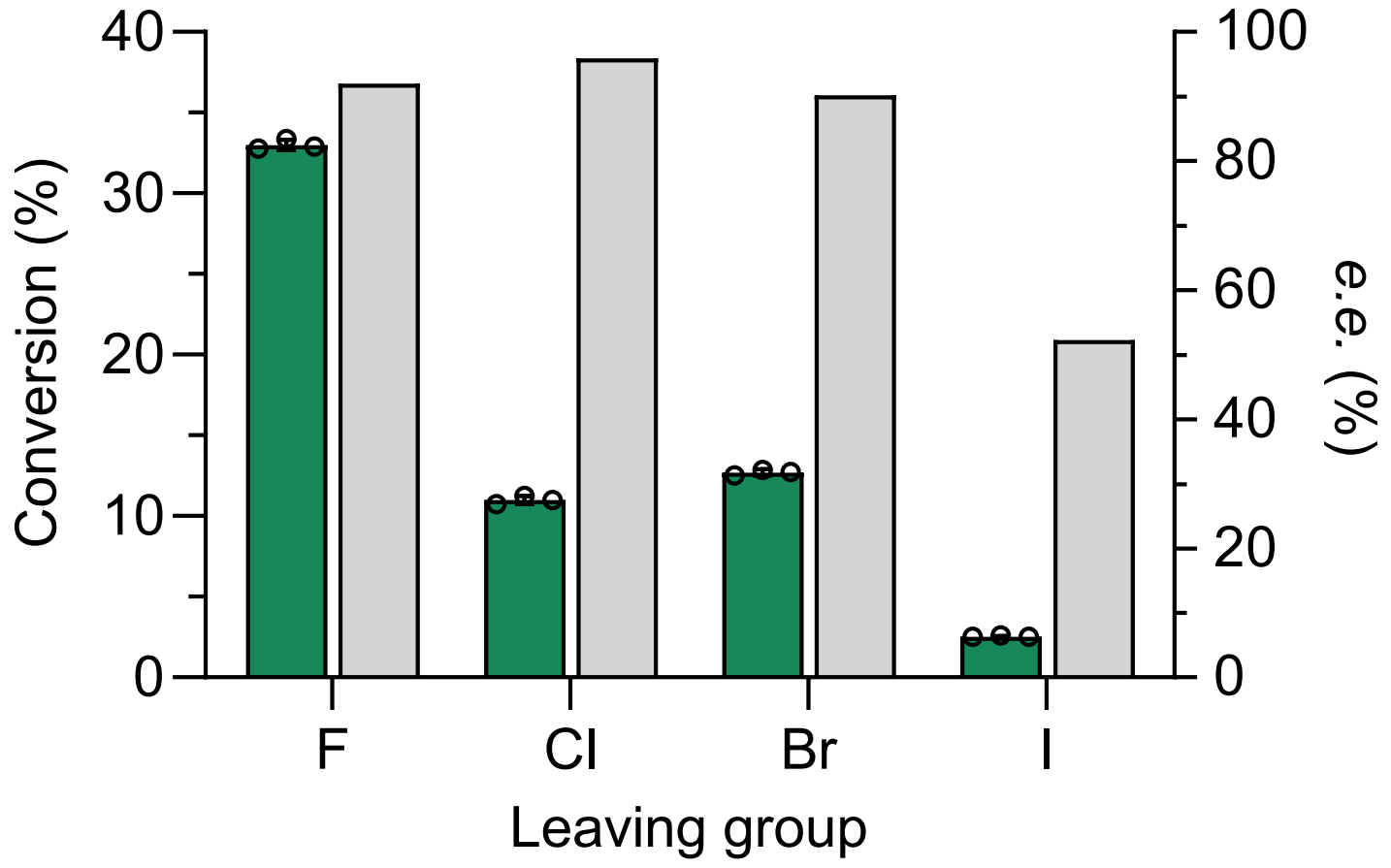

Supplementary Figure 14: The effect of leaving group on the activity and selectivity for the 4-halo-3-nitrobenzonitrile electrophile. Bar chart showing the yield (solid green bar) and selectivity (solid grey bar) achieved by eS$_N$Ar1.3 with different halide leaving groups in the 4-halo-3-nitrobenzonitrile electrophile series. Reaction conditions: **1** (10 mM), 4-halo-3-nitrobenzonitrile electrophile (1.0 mM), eS$_N$Ar1.3 (30 μM) in NaPi pH 8.0 with 10% v/v DMSO at 30 °C for 16 h. Error bars represent the standard deviation of measurements made in triplicate. Source Data are provided as a Source Data file.

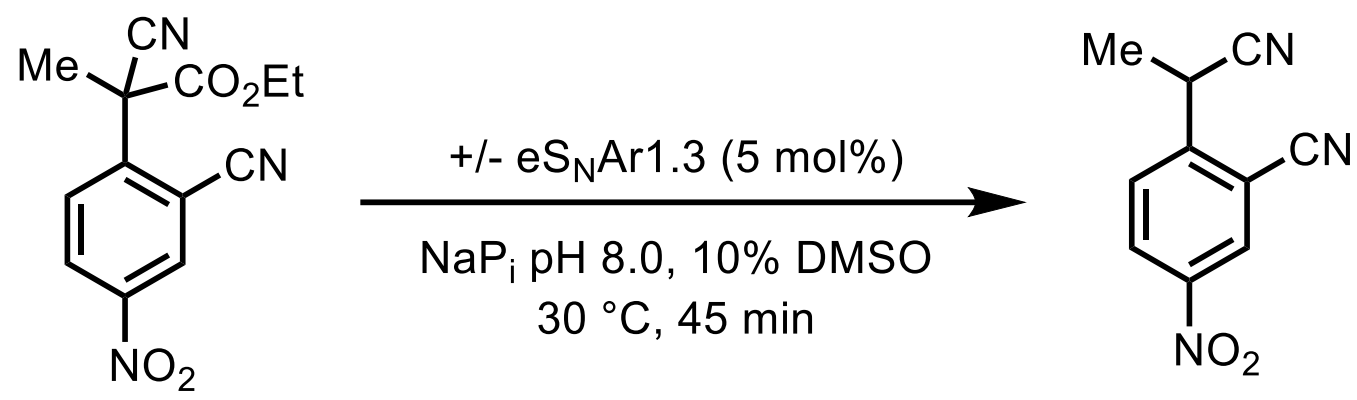

	14a	14b
NaP_i	67% yield	27% yield
$eS_NAr1.3$	66% yield	26% yield

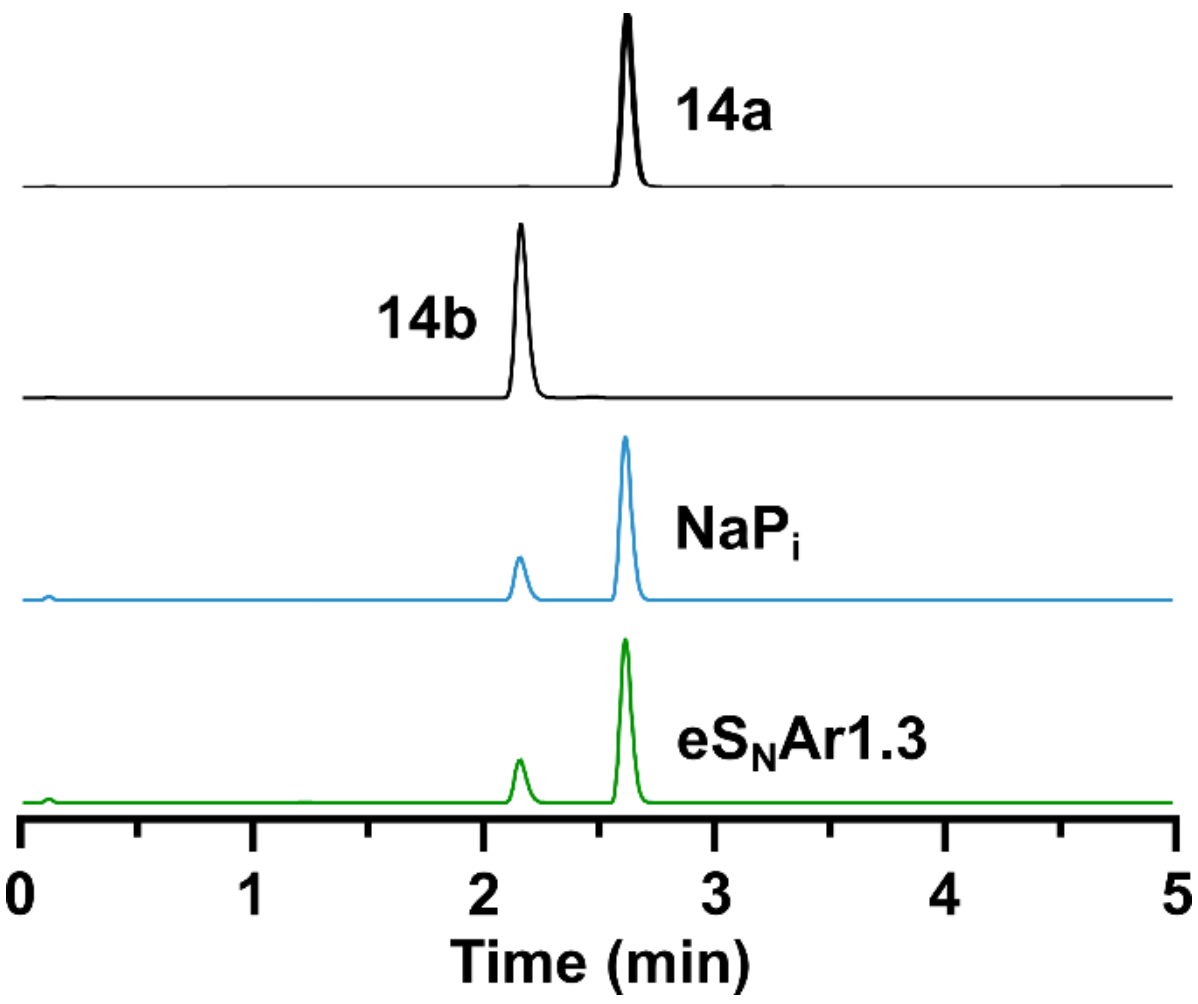

Supplementary Figure 15: Product 14a undergoes non-enzymatic decarboxylation under conditions used in biotransformations. Top, Chemical scheme showing the decarboxylation of product 14a in the absence and presence of $eS_NAr1.3$. Bottom, UPLC (260 nm) chromatogram comparing chemically synthesized standards of 14a (S_NAr product), 14b (decarboxylation product) and the reactions with (green) and without (blue) $eS_NAr1.3$, demonstrating that decarboxylation is non-enzymatic.

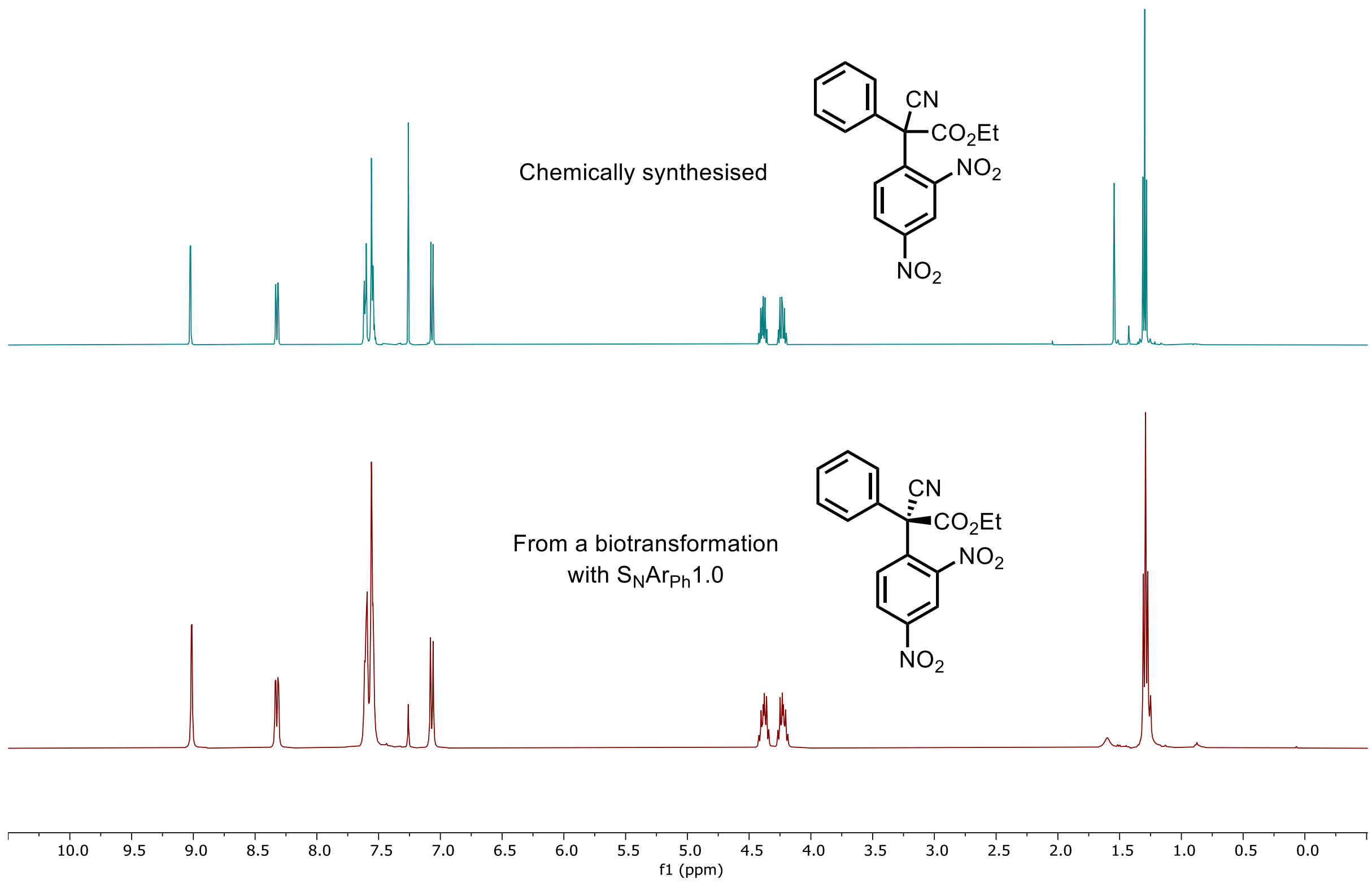

Supplementary Figure 16: ^{1}H NMR data comparing a chemically synthesised authentic standard of rac-25 with (*R*)-25 produced in a preparative scale biotransformation with S_NAr_{Ph}1.0. Top: ^{1}H NMR data (500 MHz, $CDCl_3$) for the synthetic standard of **rac-3**. Bottom: ^{1}H NMR data (400 MHz, $CDCl_3$) for **(*R*)-25** produced in a preparative-scale biotransformation using S_NAr_{Ph}1.0.

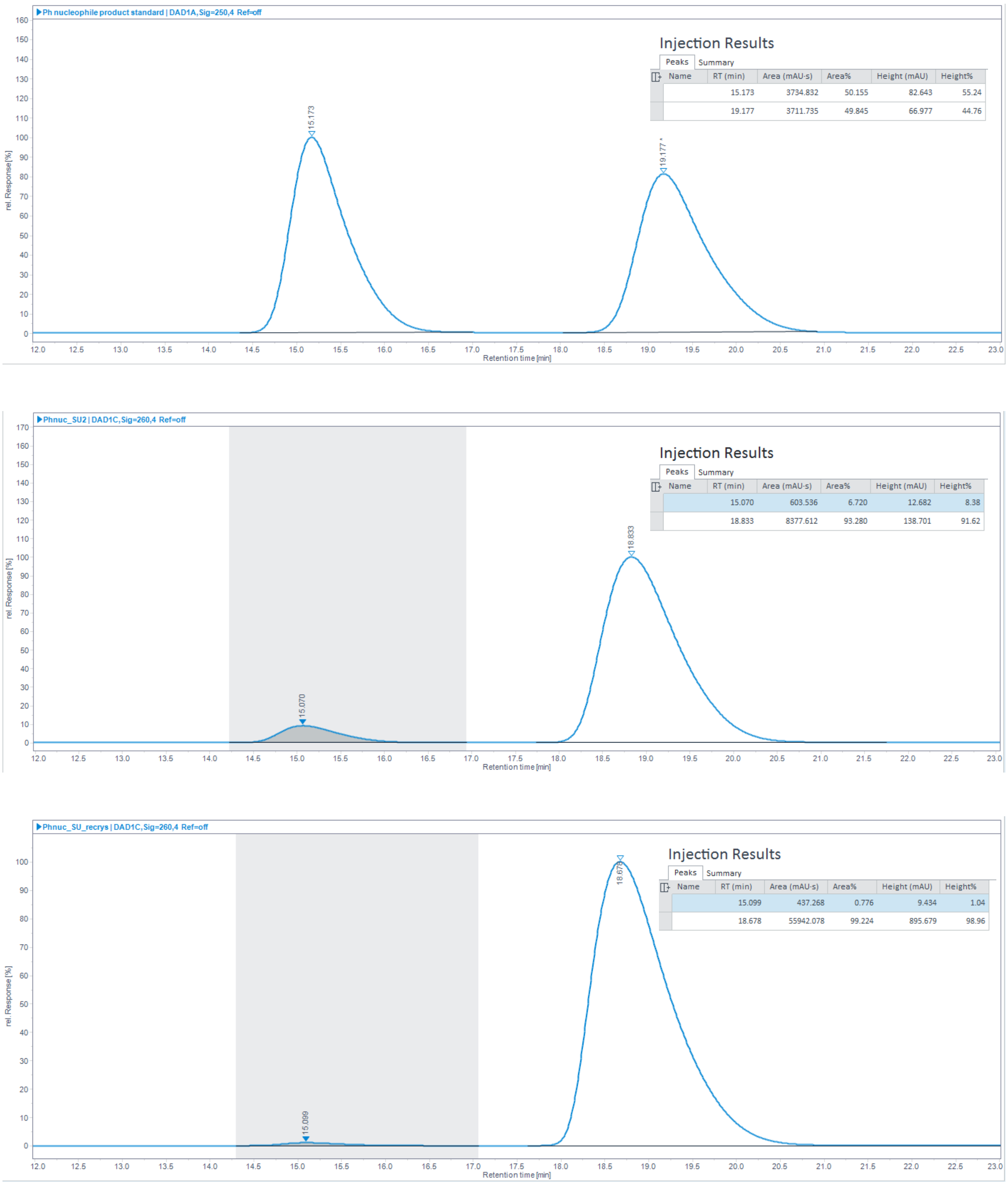

Supplementary Figure 17: Chiral HPLC data comparing a chemically synthesised authentic standard of rac-25 with (*R*)-25 produced in a preparative scale biotransformation with eS$_N$Ar1.3. Top: Chiral HPLC trace for the synthetic standard of **rac-25**; Middle: Chiral HPLC trace for enantioenriched **(*R*)-25** produced in a preparative-scale biotransformation using S$_N$Ar$_{Ph}$1.0; Bottom: Chiral HPLC trace of colourless, tetragonal single crystal of enantioenriched **(*R*)-25** that was used for X-ray diffraction studies and $[\alpha]_D$ measurements.

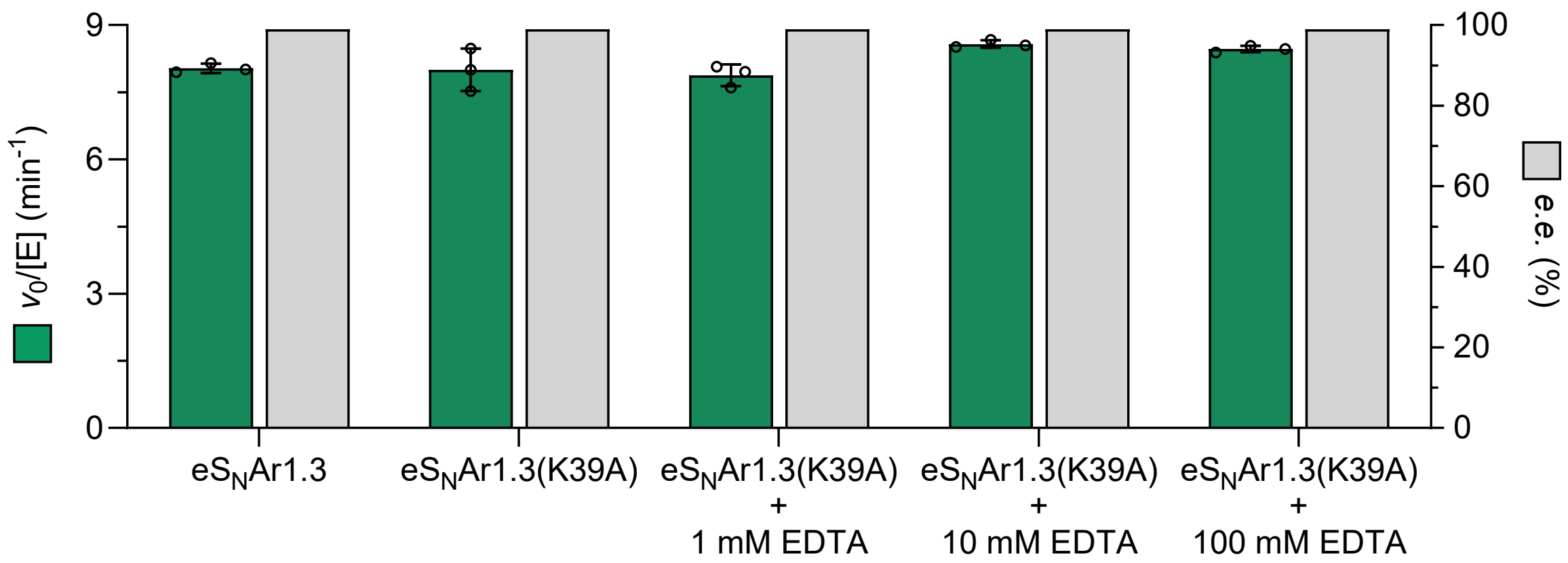

Supplementary Figure 18: The effect of the K39A point mutation in eS$_N$Ar1.3 and incubation of this variant with different concentrations of EDTA during the protein purification procedure on the initial rate and enantioselectivity. Bar chart showing the initial rates (v_0/[E], solid green bars) and enantioselectivities (*e.e.*, patterned grey bars) for eS$_N$Ar1.3, and eS$_N$Ar1.3(K39A) incubated with 0, 1, 10 or 100 mM ethylenediaminetetraacetic acid (EDTA) during protein purification. Reaction conditions for measuring initial rates: **1** (75 mM), **2** (2.5 mM) and eS$_N$Ar1.3 variant (1 μM) in NaP$_i$ pH 8.0 with 10% *v*/*v* DMSO at 30 °C. Reaction conditions for measuring enantioselectivity: **1** (25 mM), **2** (2.5 mM) and eS$_N$Ar1.3 variant (1 mol%) in NaP$_i$ pH 8.0 with 10% *v*/*v* DMSO at 30 °C for 8 h.

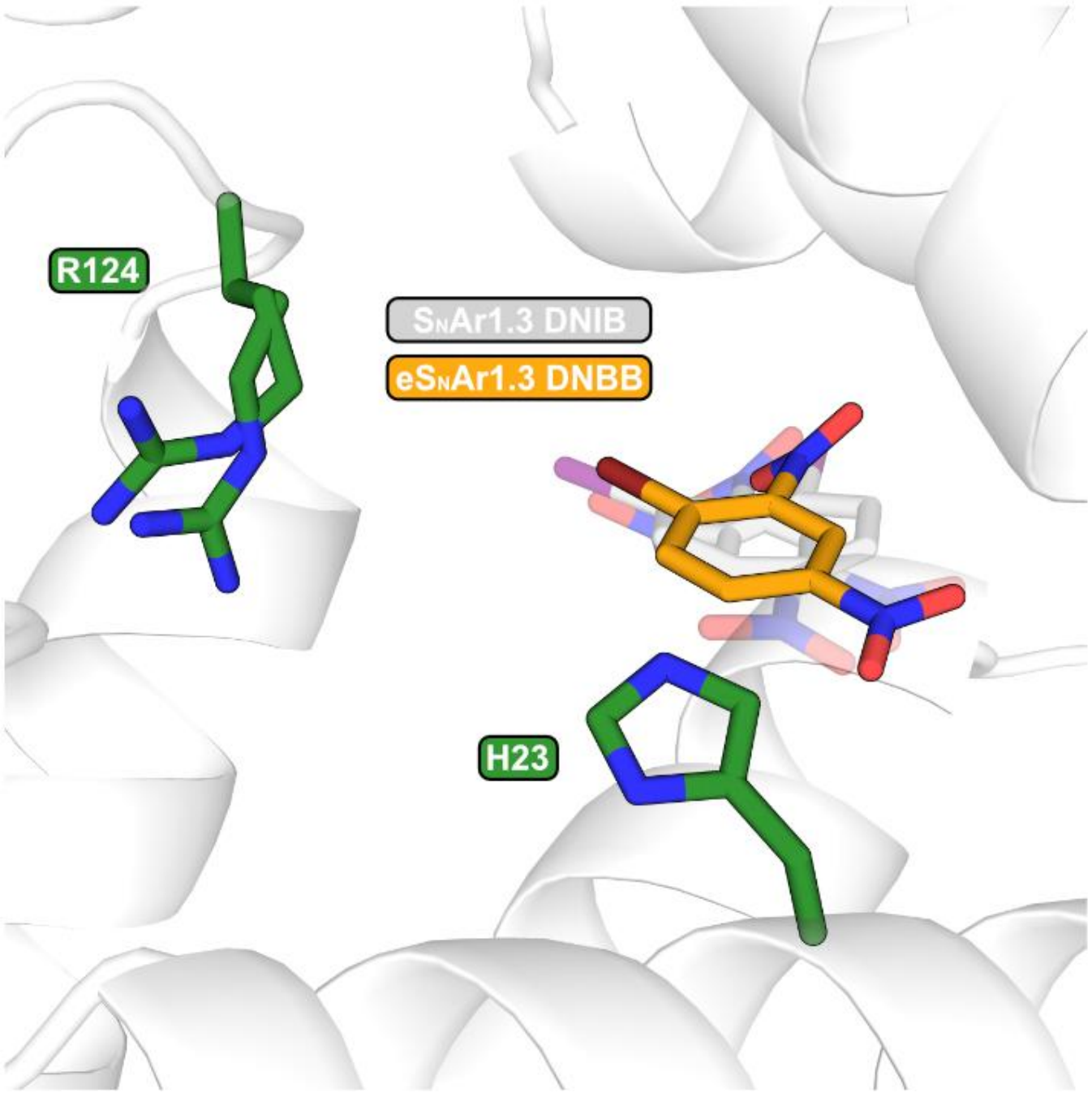

Supplementary Figure 19: Comparison of the electrophile binding modes in S_NAr1.3 and eS_NAr1.3. eS_NAr1.3(K39A) crystals soaked with **4** (orange, PDB: 9SX8) reveal one anomalous signal, in contrast to S_NAr1.3(K39A) in which two electrophile binding poses were observed (grey, PDB: 9FUG). The single binding conformation in eS_NAr1.3(K39A) overlaps well with the previously observed 'major' pose, thought to be the catalytically relevant conformation.

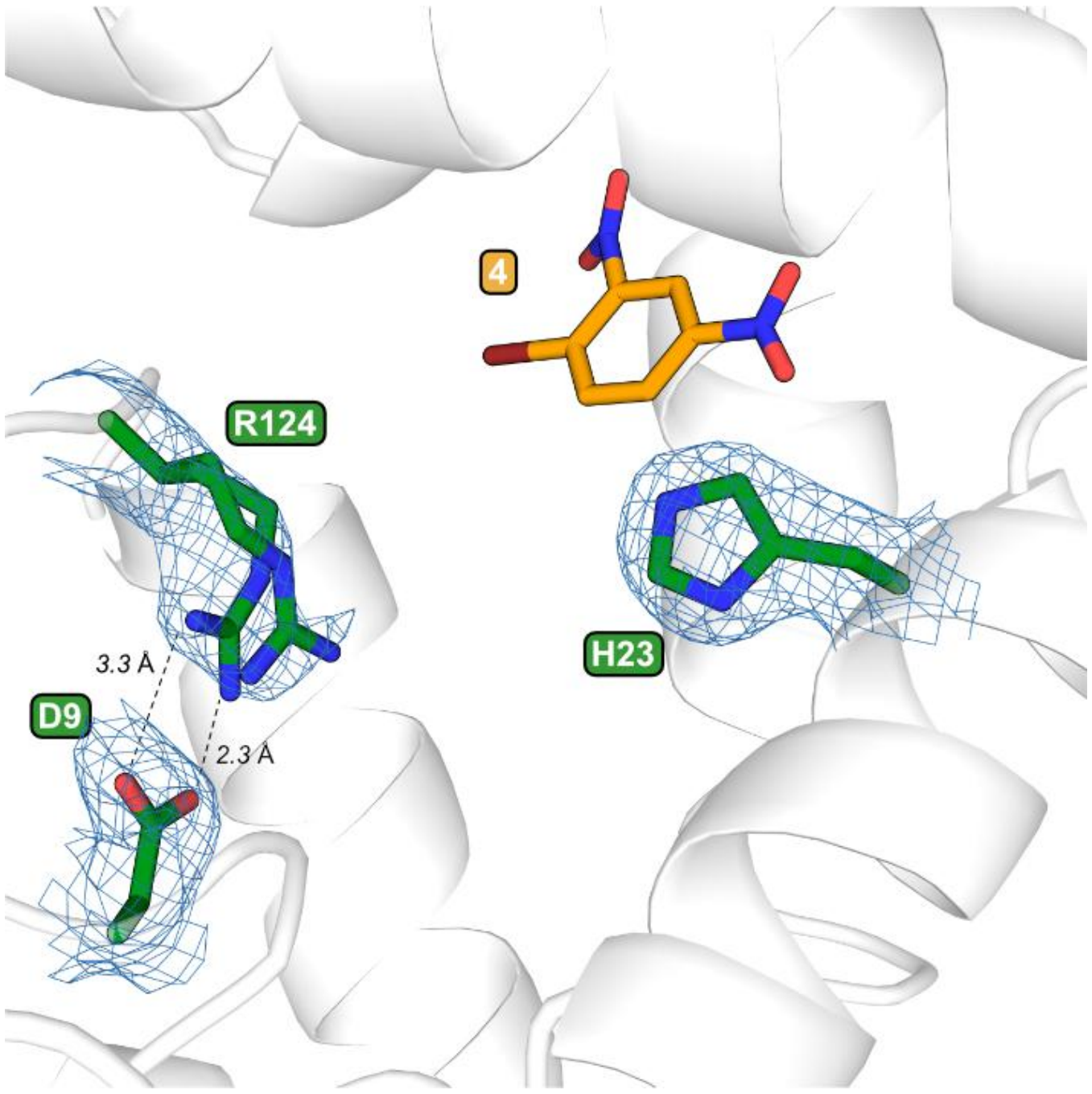

Supplementary Figure 20: Polar interactions between Asp9 and key catalytic Arg124 in an X-ray crystal structure of eS$_N$Ar1.3(K39A) that emerged during evolution. The Arg124 residue in eS$_N$Ar1.3(K39A) (green) forms a salt bridge with Asp9, leading to a significantly more ordered Arg124 residue in comparison to S$_N$Ar1.3(K39A) in complex with 2,4-dinitroiodobenzene (PDB: 9FUG). 2F$_o$-F$_c$ map for the Asp9, His23 and Arg124 residues is shown as a blue mesh contoured at 0.9σ.

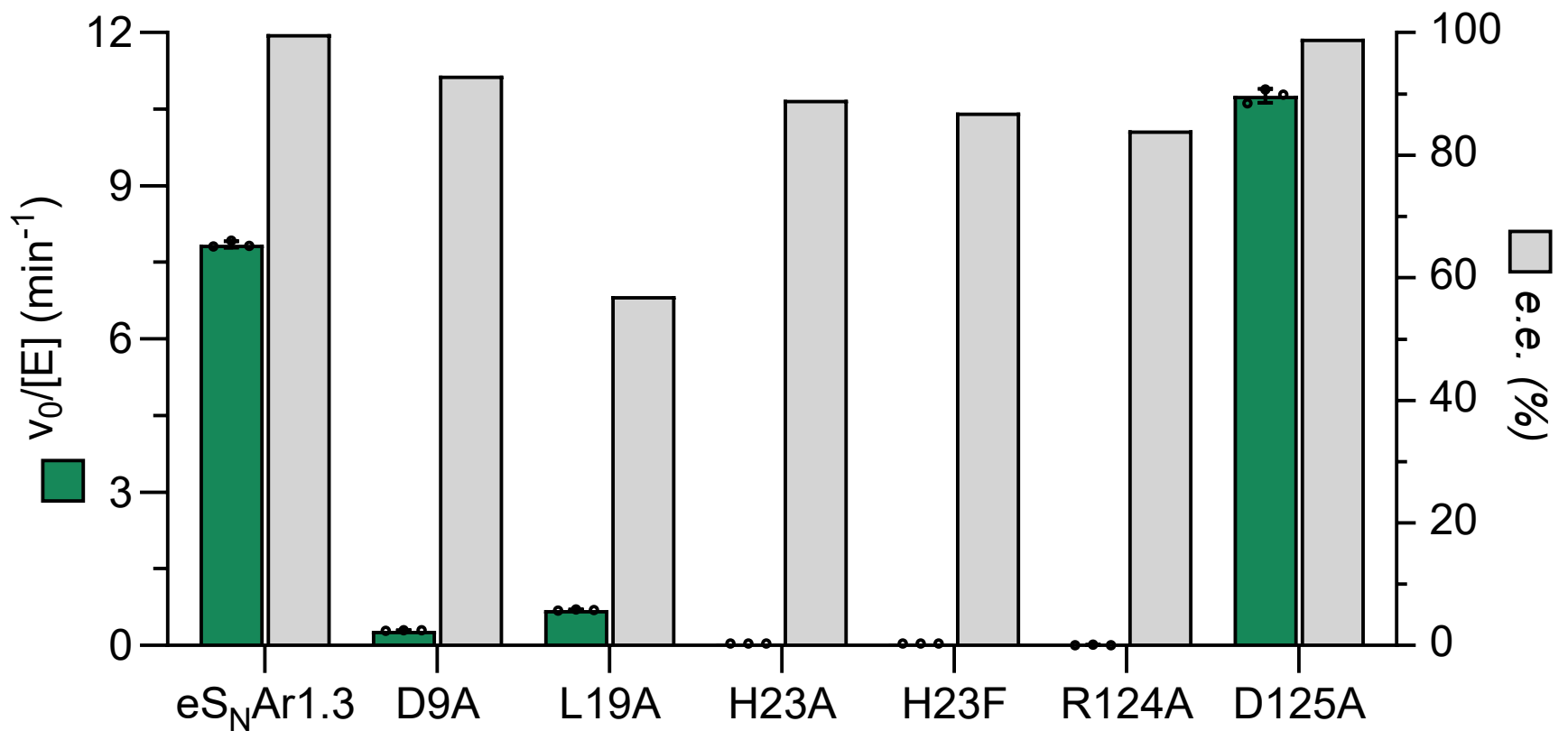

Supplementary Figure 21: The effect of mutating eS$_N$Ar1.3 active site residues on initial rate and enantioselectivity. Residues lining the halide binding site and secondary coordination sphere were mutated to understand their relative contributions to catalysis. The effects of mutating these residues on initial rate (green solid bars) and stereoselectivity (grey solid bars) were measured using 2,4-dinitrochlorobenzene (**2**) as the electrophile. Reaction conditions for measuring initial rates: **1** (75 mM), **2** (2.5 mM) and eS$_N$Ar1.3 variant (varied) in NaP$_i$ pH 8.0 with 10% *v*/*v* DMSO at 30 °C. Reaction conditions for measuring enantioselectivity: **1** (25 mM), **2** (2.5 mM) and eS$_N$Ar1.3 variant (5 mol%) in NaP$_i$ pH 8.0 with 10% *v*/*v* DMSO at 30 °C for 16 h. Error bars represent the standard deviation of measurements made in triplicate.

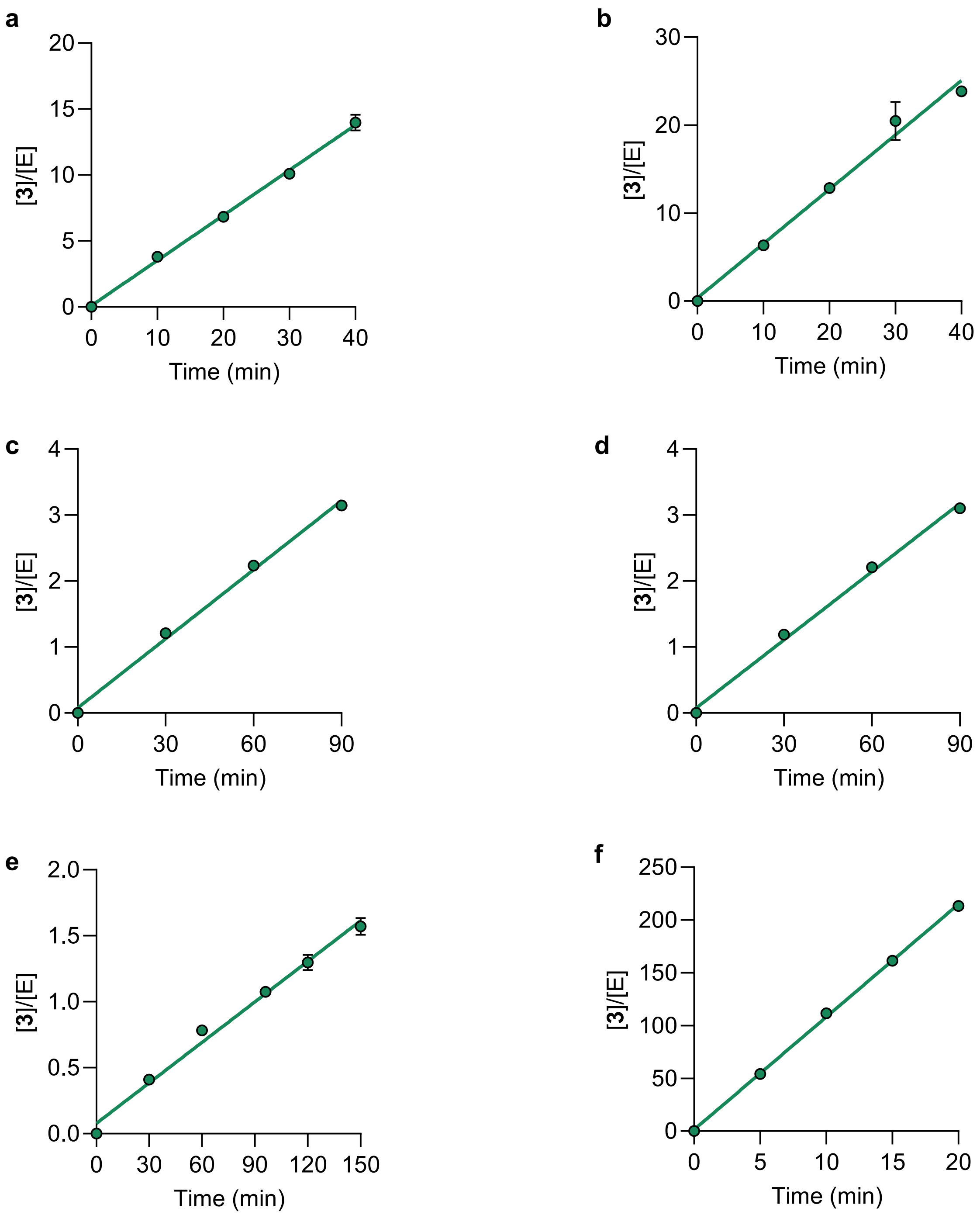

Supplementary Figure 22: The effect of the eS$_N$Ar1.3 point mutations on the reaction rate with electrophile substrate 2. Line graphs of [**3**]/[E] against time (min) for point mutations in eS$_N$Ar1.3 for residues that lining the active site: **a**, eS$_N$Ar1.3(D9A); **d**, eS$_N$Ar1.3(L19A); **e**, eS$_N$Ar1.3(H23A); **f**, eS$_N$Ar1.3(H23F); **g**, eS$_N$Ar1.3(R124A); **h**, eS$_N$Ar1.3(D125A). Data were fitted using Linear Regression equation in GraphPad Prism. Reaction conditions: **1** (75 mM), **2** (2.5 mM) and eS$_N$Ar1.3 variant in NaP$_i$ pH 8.0 with 10% *v*/*v* DMSO at 30 °C. Enzyme concentrations: eS$_N$Ar1.3(D9A), 40 µM; eS$_N$Ar1.3(L19A), 10 µM; eS$_N$Ar1.3(H23A), 100 µM; eS$_N$Ar1.3(H23F), 100 µM; eS$_N$Ar1.3(R124A), 100 µM; eS$_N$Ar1.3(D125A), 1 µM. Error bars represent the standard deviation of measurements made in triplicate

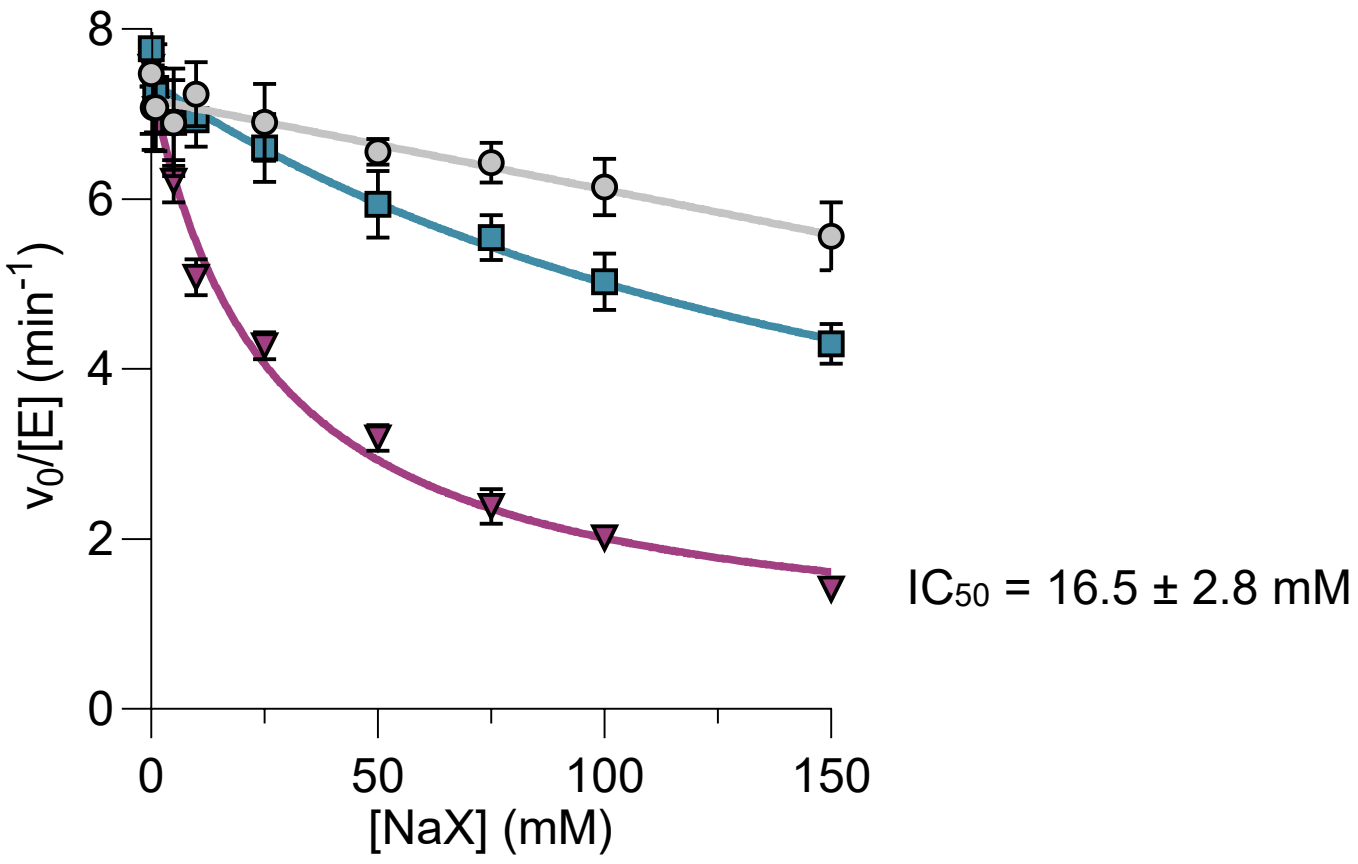

Supplementary Figure 23: Relationship between eS$_N$Ar1.3 initial rate and concentration of chloride, bromide and iodide in the reaction. Assays were performed at a fixed concentration of **1** (75 mM) and **2** (1 mM) with varying concentrations of either NaCl (grey circles), NaBr (blue squares) or NaI (purple inverted triangles). Linear fits of conversion versus time allowed determination of v_0, and the v_0 *versus* halide concentration steady-state kinetic data were fitted to the '[inhibitor] versus response' equation (Y = Bottom + (Top − Bottom)/ (1 + (X/IC$_{50}$))) using GraphPad Prism, allowing for the calculation of IC$_{50}$ values. Data points shown are averages of triplicate measurements, with error bars representing standard deviation. *Note: IC$_{50}$ values could not be obtained for assays performed with NaCl or NaBr.*

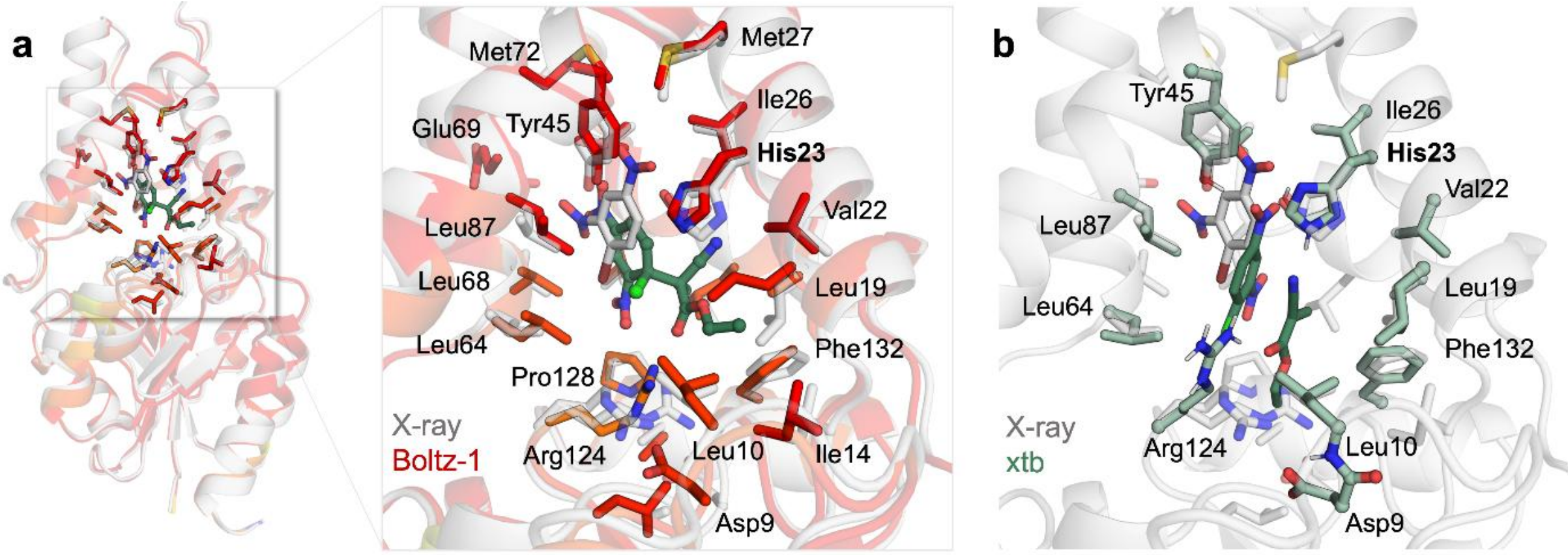

Supplementary Figure 24: eS$_N$Ar1.3 predicted structure using Boltz-2 and optimized at GFN2-xtb level. **a**, eS$_N$Ar1.3 structure complexed with the nucleophile **1** and aromatic substrate **2** was predicted with Boltz-1. An artificial bond was imposed between **1** and **2**, thus modelling the negatively charged Meisenheimer intermediate. **b**, Cluster model calculations at the semiempirical GFN2-xtb level were used to further refine the structure obtained with Boltz-2. The artificial bond between **1** and **2** was removed. This optimized pose of the nucleophile **1** and aromatic substrate **2** was used as the starting point of the first set of MD simulations.

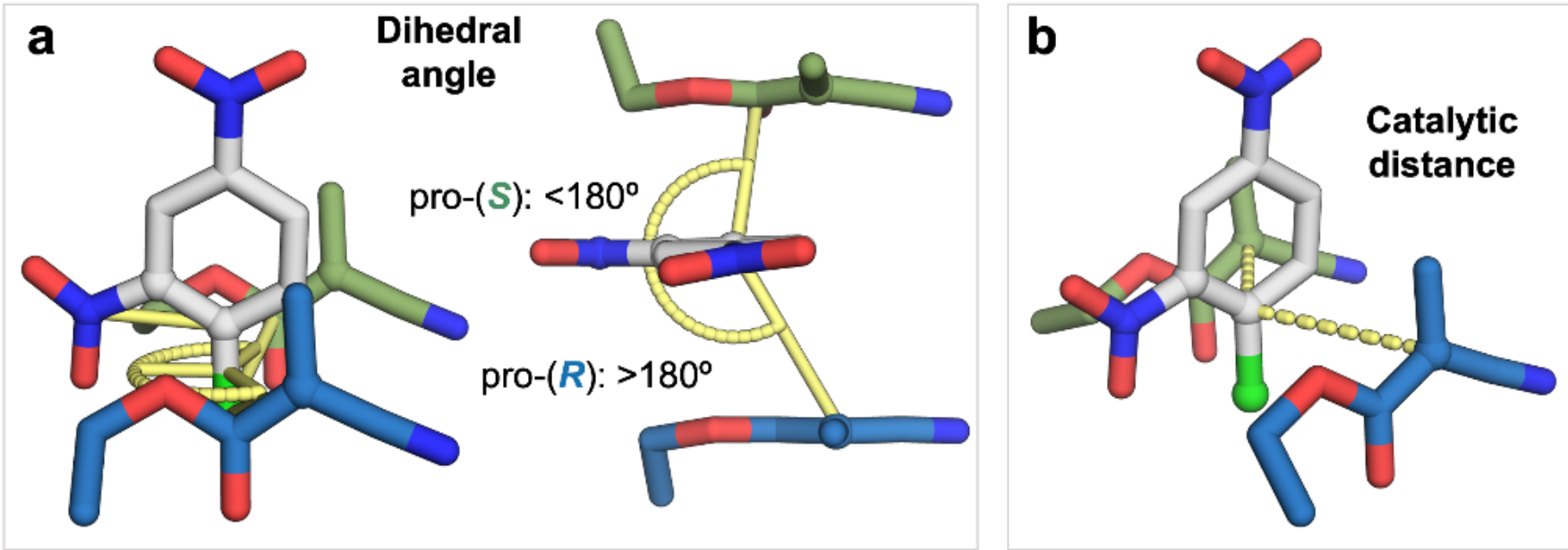

Supplementary Figure 25: Graphical definition of the dihedral angle and catalytic distance used for reconstructing the conformational landscapes. a, The dihedral angle is defined between the nitrogen atom of the *ortho*-nitro substituent (electrophile), the halocarbon (electrophile), the halogen atom (electrophile) and the chiral carbon (nucleophile). Thus, the rear pro-(*S*) attack exhibits dihedral angles < 180º, whereas the front pro-(*R*) attack > 180º. **b**, The catalytic distance is defined between the halocarbon in the electrophile and the chiral carbon atom in the nucleophile.

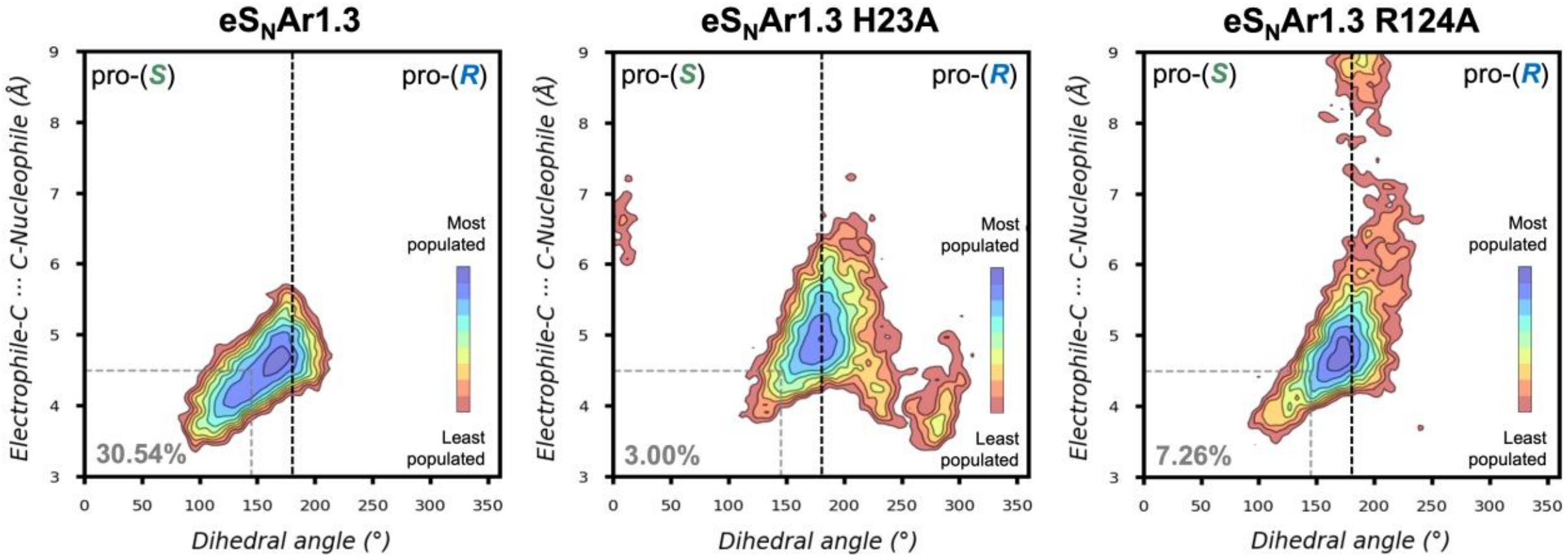

Supplementary Figure 26: Conformational landscapes of eS$_N$Ar1.3 and the single mutants H23A and R124A in the presence of nucleophile 1 and aromatic substrate 2. Conformational landscapes were reconstructed using the catalytic electrophile–nucleophile distance as the y-axis and a selected dihedral angle between substrates **1** and **2** as the x-axis to discriminate between pro-R and pro-S attack geometries (shown in Figure S23). Dihedral angles <180° correspond to pro-S attack. The most populated conformations are shown in blue, whereas the least populated conformations are shown in red. The percentage of frames adopting a catalytically favorable conformation, defined by an electrophile–nucleophile distance <4.5 Å and a dihedral angle <145°, is indicated for each system; this region is highlighted with a grey dashed box.

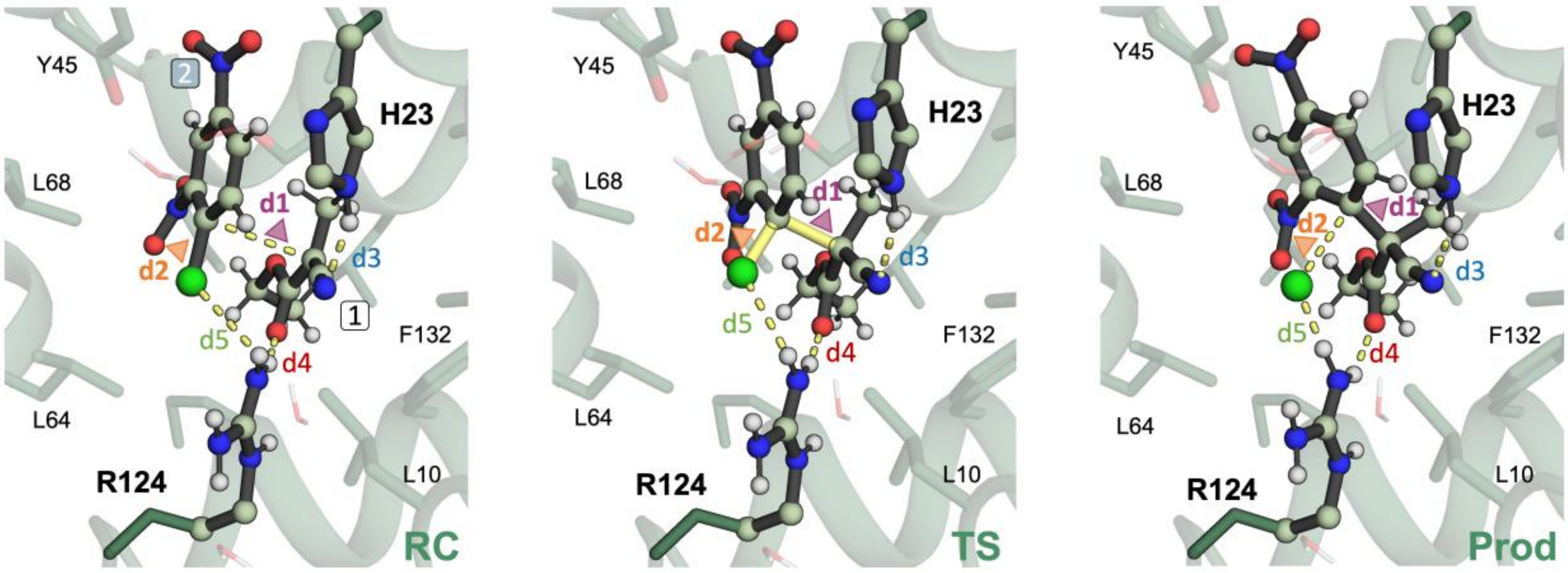

Supplementary Figure 27: Optimized Reactant-complex (RC), Transition-State (TS), and Product (Prod) structures obtained from QM/MM calculations. Key interatomic distances are highlighted, with the corresponding values reported in Figure 5d. The QM region comprises substrates **1** and **2** and residues His23 and Arg124; atoms included in the QM region are shown as spheres.

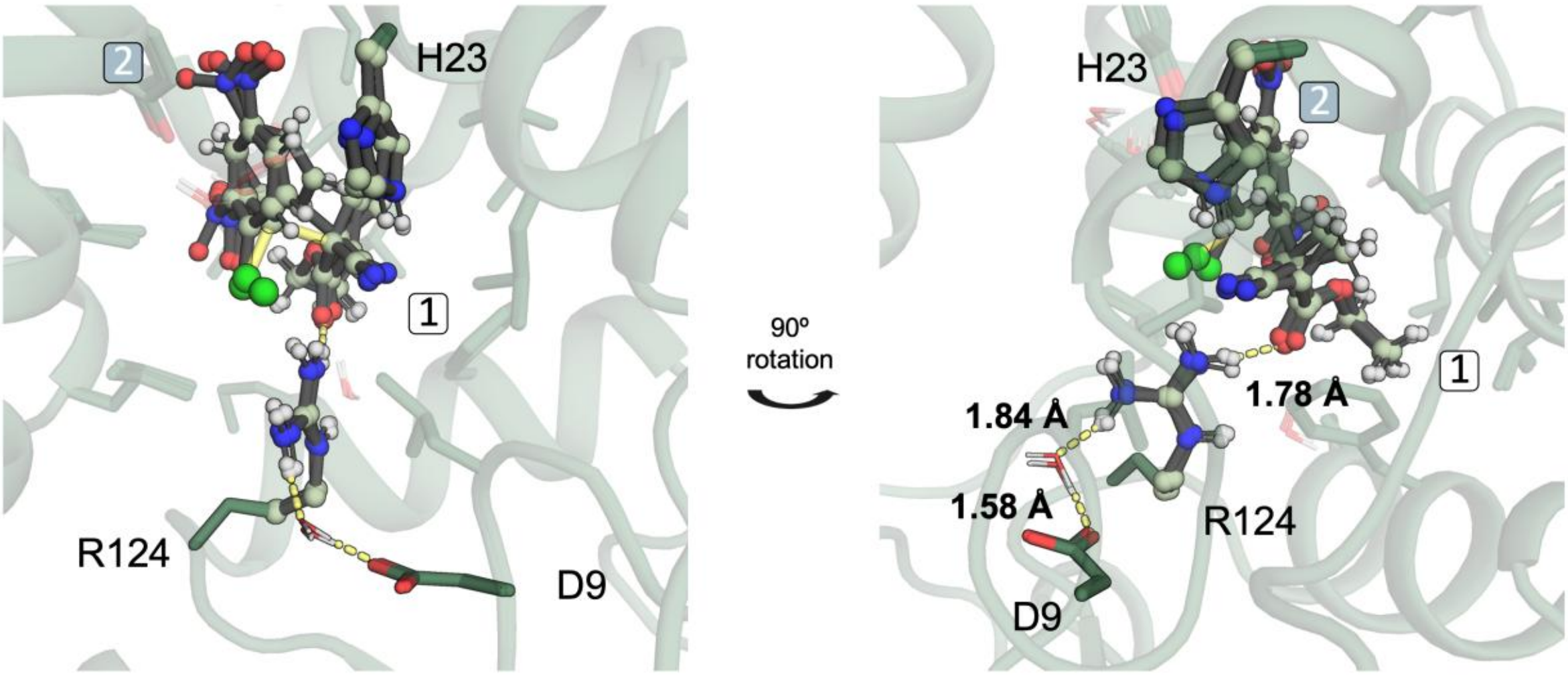

Supplementary Figure 28: Overlay of the optimized reactant-complex (RC), transition-state (TS), and product (Prod) structures obtained from QM/MM calculations. Arg124 is stabilized by Asp9 through a water-mediated hydrogen-bonding interaction. The involved distances are shown (in Å) in the right panel.

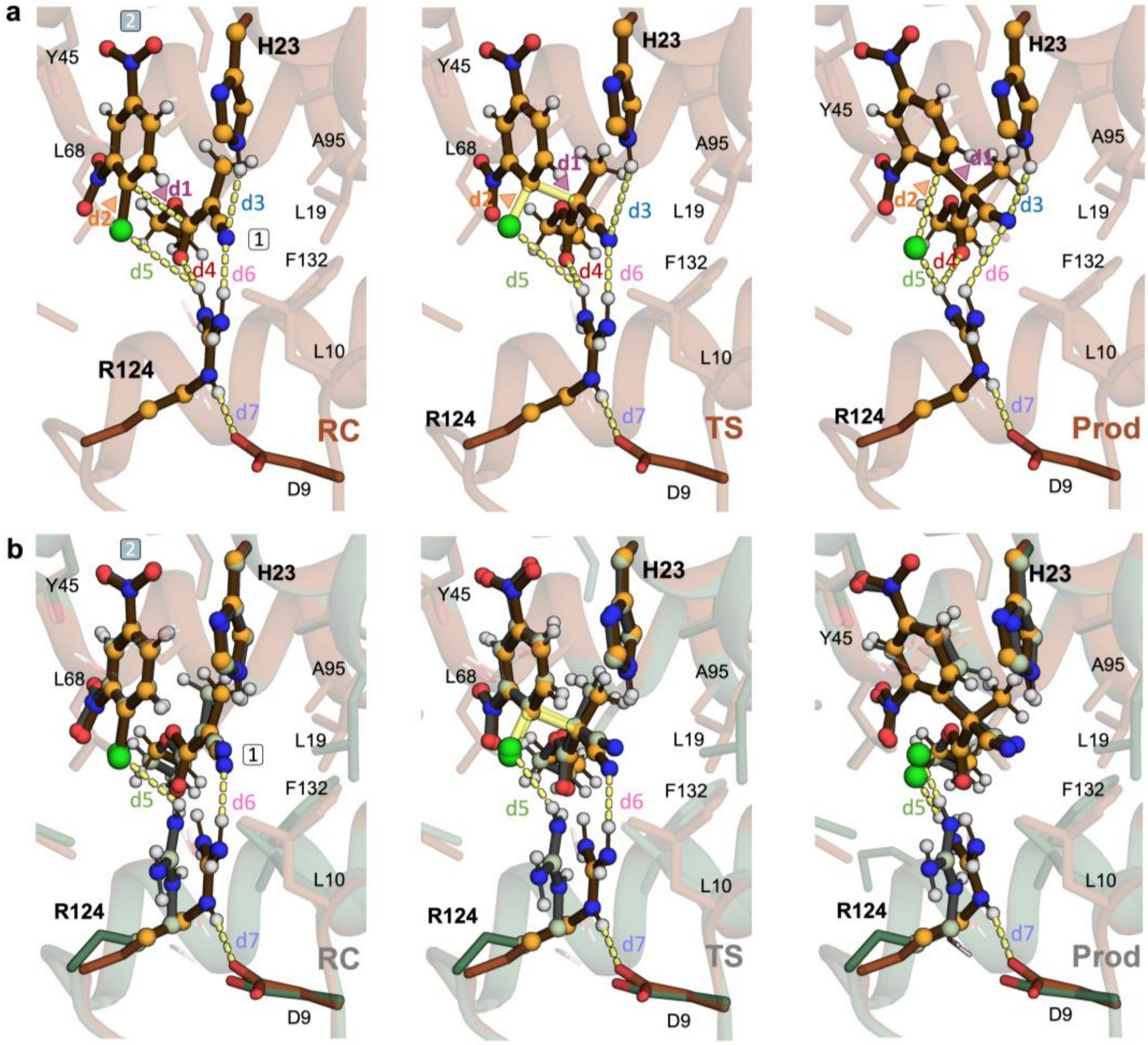

c

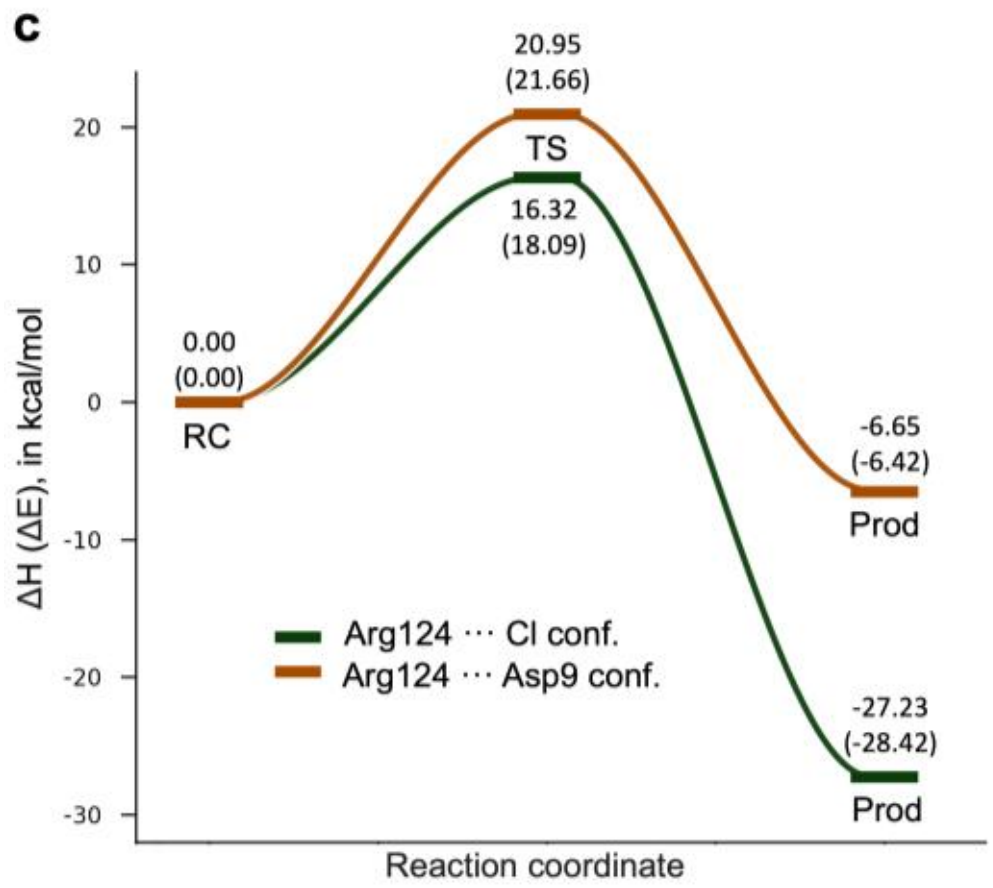

Arg124 ··· Cl conf.

	▲ d1	▲ d2	d3	d4	d5	d6
RC	3.312	1.734	2.037	1.679	2.947	2.467
TS	2.055	1.872	2.242	1.778	2.613	3.050
Prod	1.552	3.330	2.239	2.079	1.914	3.391

Arg124 ··· Asp9 conf.

	▲ d1	▲ d2	d3	d4	d5	d6	d7
RC	3.400	1.733	2.501	1.786	3.593	1.993	1.909
TS	2.034	1.851	3.007	2.090	3.601	2.190	1.878
Prod	1.546	3.491	2.317	2.597	2.045	3.027	2.040

Supplementary Figure 29: QM/MM mechanistic analysis of the alternative Arg124 conformation. a, Reactant-complex (RC), transition-state (TS), and product (Prod) structures obtained from QM/MM calculations with Arg124 in the Arg124···Asp9 conformation. Key interatomic distances are highlighted. **b,** Structural overlays comparing the Arg124···Asp9 and Arg124···Cl conformations for the RC, TS, and Prod states. Distances associated with the main conformational differences are highlighted. **c,** Comparison of the QM/MM reaction profiles along the reaction coordinate for the Arg124···Asp9 and Arg124···Cl conformations, reported as ΔH and ΔE in kcal/mol. Relevant interatomic distances are listed for both conformations to highlight the main structural differences along the reaction pathway.

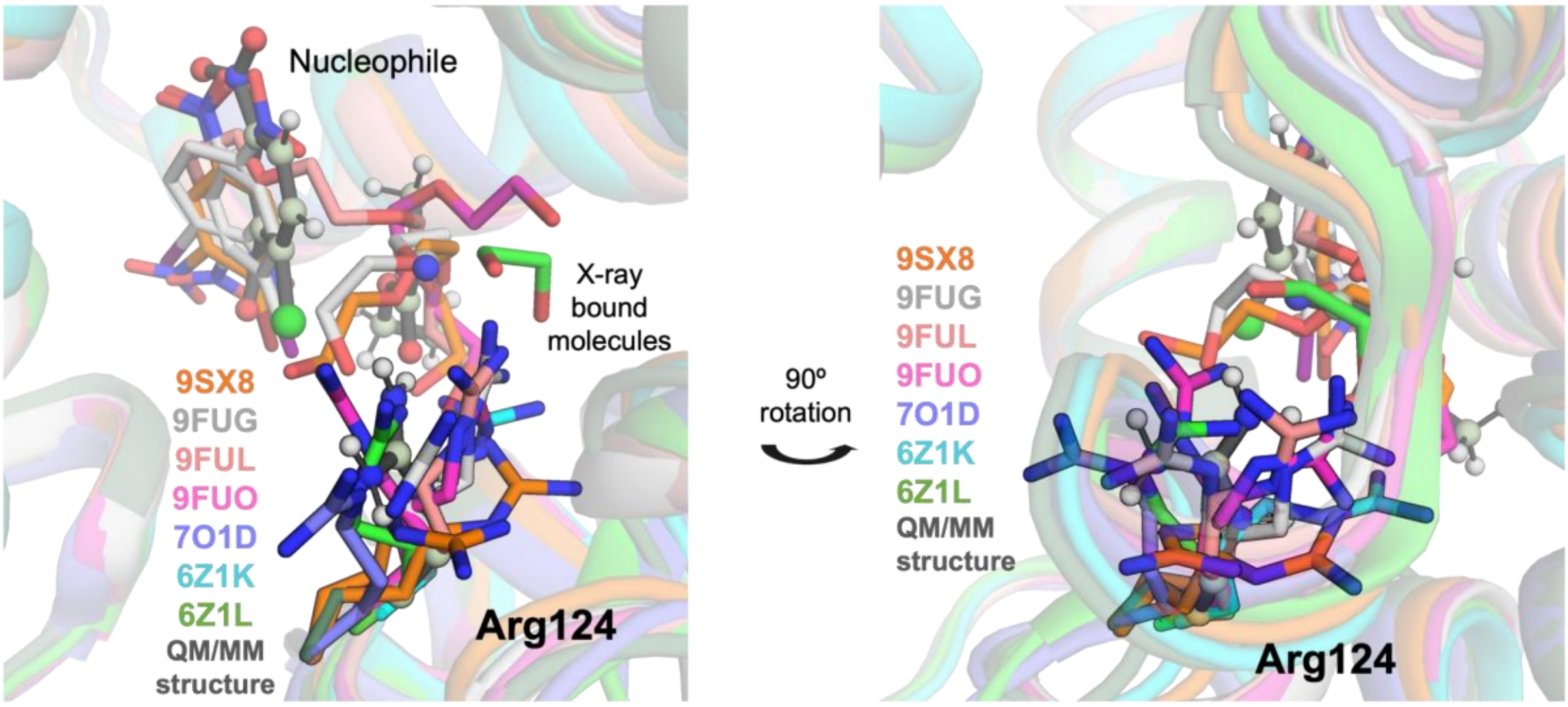

Supplementary Figure 30: Arg124 flexibility in related X-ray structures. Overlay of available X-ray structures of eS$_N$Ar1.3 (PDB: 9SX8), S$_N$Ar1.3 (PDB: 9FUG, 9FUL, 9FUO) and related BH32 variants (PDB: 7O1D, 6Z1K, 6Z1L), highlighting the conformational flexibility of Arg124. The QM/MM-derived reactant-complex structure is also shown as spheres.

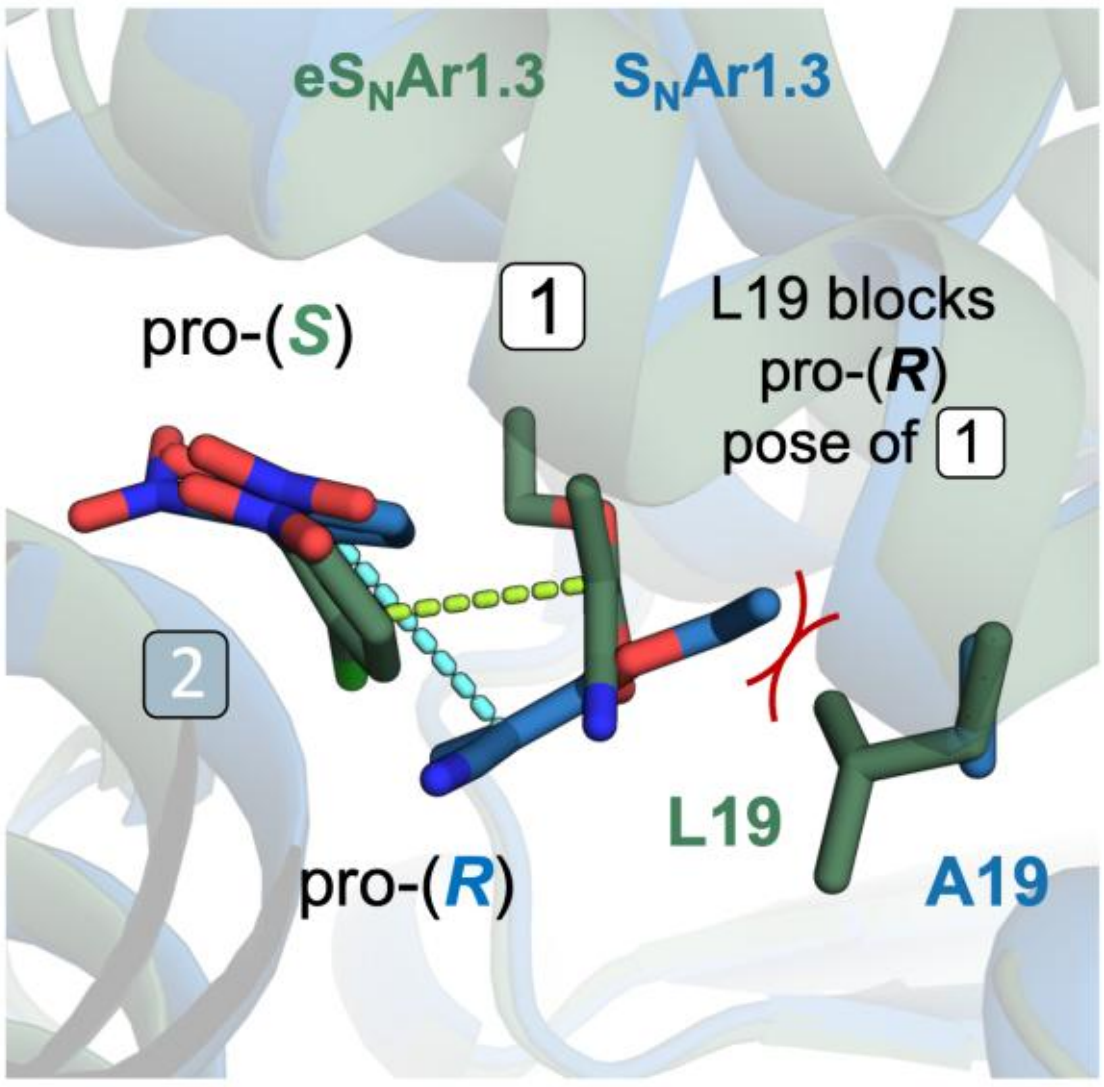

Supplementary Figure 31: Overlay of eS$_N$Ar1.3 and S$_N$Ar1.3 representative structures extracted from MD simulations. eS$_N$Ar1.3 (in green) and S$_N$Ar1.3 (in blue) were modelled with the nucleophile **1** and chloride-containing electrophile **2**. The A19L mutation introduced in eS$_N$Ar1.3 blocks the front pro-(*R*) attack. The nucleophilic attack distances are indicated with dashed lines.

Supplementary Tables

Supplementary Table 1: Electrophile scope of eS$_N$Ar1.3

Reaction conditions for the production of **3** and **6**-**18** using eS$_N$Ar1.3 or a variant of eS$_N$Ar1.2 on an analytical scale. Reactions were performed in NaP$_i$ pH 8.0 with 10% *v*/*v* DMSO at 30 °C unless stated otherwise. Standard deviations are given for measurements in triplicate. Reaction conversions were determined based on UPLC calibration curves of authentic standards of starting materials and products. Extinction coefficients of electrophiles and products are given in Supplementary Table 3 and UPLC analysis methods are given in Supplementary Table 4. The *e.e.* values were determined using chiral HPLC analysis, chiral HPLC methods are given in Supplementary Table 5. Chiral HPLC chromatograms of product standards and biotransformations are provided at the end of the Supplementary Information.

Product	Electrophile, conc. (mM)	Nucleophile, conc. (mM)	Catalyst loading (mol%)	Time (h)	Yield (%)	S.D.	*e.e.* (%)
**3**	**2,** 2.5	**1**, 2	0.5	20	>99	0	99
**6**	1-(4-fluoro-3-nitrophenyl)ethan-1-one, 2.5	**1**, 25	5	20	28	0.416	94
**7**	4-Fluoro-3-nitrobenzonitrile, 2.5	**1**, 25	5	20	68	0.093	92
**8**	Methyl 4-fluoro-3-nitrobenzoate, 2.5	**1**, 25	5	20	5	0.212	57
**9**	1-Fluoro-2-nitro-4-(trifluoromethyl)benzene, 2.5	**1**, 25	5	20	39	0.178	73
**10**	2-Chloro-1-fluoro-4-nitrobenzene, 2.5	**1**, 25	5	20	64	0.374	>99
**11**	2-Bromo-1-fluoro-4-nitrobenzene, 2.5	**1**, 25	5	20	29	0.795	>99
**12** [a]	1-Fluoro-4-nitro-2-(trifluoromethyl)benzene, 2.5	**1**, 25	5	20	40	0.217	99
**13** [b]	Methyl 2-fluoro-5-nitrobenzoate, 2.5	**1**, 25	5	20	25	0.212	88
**14a**	2-Fluoro-5-nitrobenzonitrile, 2.5	**1**, 25	5	20	47	0.188	>99
**14b**			5	20	52	0.506	n/a
**15**	4-Chloro-3-nitropyridine, 2.5	**1**, 25	5	20	46	0.214	99
**16** [c]	2-Bromo-5-nitro-3-(trifluoromethyl)pyridine, 2.5	**1**, 25	5	20	90	0.692	94
**17a**	4-Chloro-6-(trifluoromethyl)pyrimidine, 2.5	**1**, 25	5	20	31	0.957	35
**17b**			5	20	10	0.351	n/a
**18a** [d]	6-Chloropyrimidine-4-carbonitrile, 2.5	**1**, 25	5	20	85	0.219	71
**18b** [d]		**1**, 25	5	20	5	0.115	n/a

[a] Reaction performed using eS$_N$Ar1.2(L13F R65V). [b] Reaction performed using eS$_N$Ar1.2(R65V D66E). [c] Reaction performed using eS$_N$Ar1.2(L13F R65V D66E). [d] Reaction performed using eS$_N$Ar1.2(E9D L13F R65V).

Supplementary Table 2: Nucleophile scope of eS$_N$Ar1.3

Reaction conditions for the production of **19**-**25** using eS$_N$Ar1.3 on an analytical scale. Reactions were performed in NaP_i pH 8.0 with 10% *v*/*v* DMSO at 30 °C unless stated otherwise. Standard deviations are given for measurements in triplicate. Reaction conversions were determined based on UPLC calibration curves of authentic standards of starting materials and products. Extinction coefficients of products are given in Supplementary Table 3 and UPLC analysis methods are given in Supplementary Table 4. The *e.e.* values were determined using chiral HPLC analysis, chiral HPLC methods are given in Supplementary Table 5. Chiral HPLC chromatograms of product standards and biotransformations are provided at the end of the Supplementary Information.

Product	Electrophile, conc. (mM)	Nucleophile, conc. (mM)	Catalyst loading (mol%)	Time (h)	Yield (%)	S.D.	*e.e.* (%)
**19**	**2,** 2.5	*t*-Butyl 2-cyanopropanoate, 25	5	20	53	0.510	89
**20**	**2,** 2.5	Benzyl 2-cyanopropanoate, 25	5	20	>99	0.241	>99
**21**	**2,** 2.5	*i*-Propyl 2-cyanopropanoate, 25	1	20	97	0.132	98
**22**	**2,** 2.5	2-Cyano-*N*-ethylpropanamide, 25	5	20	7	0.478	93
**23**	**2,** 2.5	Ethyl 2-cyanopent-4-enoate, 25	5	20	96	0.278	93
**24**	**2,** 2.5	Ethyl 2-cyano-3-methylbutanoate, 25	5	20	16	0.539	>99
**25** [a]	**2**, 1	Ethyl 2-cyano-2-phenylacetate, 2	5	20	69	0.185	66

[a] Reaction performed using NaP_i pH 6.0 as the buffer.

Supplementary Table 3: Extinction coefficients of electrophiles and products

Compound	Extinction coefficient (mM^{-1} cm^{-1}) [a]
**2**	1620
**3**	1450
**4**	1600
**5**	960
1-(4-Fluoro-3-nitrophenyl)ethan-1-one	1000
**6**	860
4-Fluoro-3-nitrobenzonitrile	810
**7**	770
Methyl 4-fluoro-3-nitrobenzoate	800
**8**	800
1-Fluoro-2-nitro-4-(trifluoromethyl)benzene	860
**9**	640
2-Chloro-1-fluoro-4-nitrobenzene	
**10**	1250
2-Bromo-1-fluoro-4-nitrobenzene	960
**11**	1250
1-Fluoro-4-nitro-2-(trifluoromethyl)benzene	1220
**12**	680
Methyl 2-fluoro-5-nitrobenzoate	1080
**13**	300
2-Fluoro-5-nitrobenzonitrile	1190
14a	1450
14b	1705
4-Chloro-3-nitropyridine	630
**15**	650
2-Bromo-5-nitro-3-(trifluoromethyl)pyridine	1550
**16**	900
4-Chloro-6-(trifluoromethyl)pyrimidine	410
**17a**	130
**17b**	370
6-Chloropyrimidine-4-carbonitrile	1030
**18a**	1150
**18b**	600
**19**	1805
**21**	1820
**20**	1835
**22**	1720
**23**	1750
**24**	1780
**25**	2030

[a] Using an injection volume of 3 µL with the detector wavelength set to 260 nm.

Supplementary Table 4: Reverse-phase UPLC analysis methods

Product	Electrophile	Flow rate (mL min^{-1})	Mobile phase (% MeCN in MQ H_2O with 0.1% TFA)	Run time (min)
**3** [a]	**2**	1.2	35	1.6
	**4**	1.2	25	5
	**5**	1.2	25	5
**6 to 24**	**Varied**	1	5 (0.3 min)<br>5-95% (8.5 min)<br>95-5% (8.6 min)<br>5% (10 min)	10
**25** [b]	**2**	1	30 (0.1 min)<br>30-70% (2.25 min)<br>70-30% (2.3 min)<br>30% (2.5 min)	2.5

[a] Column temperature set to 20 °C
[b] Column temperature set to 30 °C

Supplementary Table 5: Normal-phase chiral HPLC analysis methods

Product	Flow rate (mL min^{-1})	Mobile phase (% *i*PrOH in hexane)	Run time (min)
**3** [a]	1	20	25
**6** [a]	1	10	40
**7** [a]	1	10	35
**8** [b]	1	10	25
**9** [b]	0.8	2	25
**10** [a]	1	10	20
**11** [a]	1	10	20
**12** [a]	1	10	15
**13** [a]	1	10	25
**14a** [a]	1	10	25
**15** [a]	0.5	5	60
**16** [b]	1	20	20
**17a** [c]	0.7	2	25
**18a** [d]	1	3 (0-15 min)<br>3-20 (15-16 min)<br>20 (16-26 min)<br>20-3 (26-27 min)<br>3 (27-40 min)	40
**19** [b]	1	8	30
**20** [a]	1	10	50
**21** [a]	1	10	25
**22** [a]	1	18	25
**23** [a]	1	20	15
**24** [a]	0.7	5	35
**30** [a]	1	10	25

[a] Daicel CHIRALPAK® AS (25 cm, 0.46 cmØ)

[b] Daicel CHIRALPAK® OD-H (25 cm, 0.40 cmØ)

[c] Phenomenex i-Amylose (25 cm, 0.46 cmØ)

[d] Daicel CHIRALPAK® IA (25 cm, 0.46 cmØ)

Supplementary Table 6: Calculated and experimental masses of apo enzymes

Variant	Expected Mass (Da)	Observed Mass (Da)
$eS_NAr1.0$	27621.8	27622.0
$eS_NAr1.1$	27548.7	27548.0
$eS_NAr1.2$	27555.8	27555.3
$eS_NAr1.3$	27484.7	27484.2
$eS_NAr1.3$(D9A)	27440.7	27441.1
$eS_NAr1.3$(L19A)	27442.6	27442.9
$eS_NAr1.3$(H23A)	27418.6	27419.3
$eS_NAr1.3$(H23F)	27494.7	27495.0
$eS_NAr1.3$(K39A)	27427.6	27428.0
$eS_NAr1.3$(R124A)	27399.6	27400.5
$eS_NAr1.3$(D125A)	27440.7	27440.7

Supplementary Table 7: Primer sequences used to generate DNA libraries

**Flanking primers**	
Nde_F	catgcatgCATATGATTCGTGCGGTATTC
Xho_R	catgcatgCTCGAGAGAGCCCTGACC
**Round 1**	
L10x_F	CatgcatgcatATGATTCGTGCGGTATTCTTTGATAGC NNK GGTACTCTGATTAGCGTTG
I14x_F	CatgcatgcatATGATTCGTGCGGTATTCTTTGATAGCCTGGGTACTCTG NNK AG CGTTGAAGGCGCT
V22x_F	GAAGGCTTGTATAAA NNK CATCTGAAAATTATGGAGGAAGTG
V22x_R	TTTATACAAGCCTTCAACGCTA
I26x_F	AAAGTGCATCTGAAA NNK ATGGAGGAAGTGCTGG
I26x_R	TTTCAGATGCACTTTATACAAGC
M27x_F	GTGCATCTGAAAATT NNK GAGGAAGTGCTGGGT
M27x_R	AATTTTCAGATGCACTTTATACAAGC
Y45x_F	ACCCTGCTGGACGAA NNK GAGAAACTGGCTCGC
Y45x_R	TTCGTCCAGCAGGGT
E46x_F	CTGCTGGACGAATAC NNK AAACTGGCTCGCGAA
E46x_R	GTATTCGTCCAGCAGGG
A49x_F	GAATACGAGAAACTG NNK CGCGAAGCGTTCTCT
A49x_R	CAGTTTCTCGTATTCGTCCAG
F53x_F	CTGGCTCGCGAAGCG NNK TCTAACTATGCGGGCAA
F53x_R	CGCTTCGCGAGC
L64x_F	AAACCGTATCGTCCG NNK CGTGATATCCTGGAAGAAGTAAT
L64x_R	GGACGATACGGTTTGCC
R65x_F	CCGTATCGTCCGCTG NNK GATATCCTGGAAGAAGTAATGCG
R65x_R	CAGCGGACGATACGG
E69x_F	CTGCGTGATATCCTG NNK GAAGTAATGCGTAAACTGGC

E69x_R	CAGGATATCACGCAGCG
M72x_F	GATATCCTGGAAGAAGTA NNK CGTAAACTGGCGGAAAAG
M72x_R	TACTTCTTCCAGGATATCACGC
Y83x_F	AAGTACGGTTTCAAA NNK CCTGAAAACTTGTGGGAAATC
Y83x_R	TTTGAAACCGTACTTTTCCG
L87x_F	AAATACCCTGAAAAC NNK TGGGAAATCTCCCTGC
L87x_R	GTTTTCAGGGTATTTGAAACCG
W88x_F	TACCCTGAAAACTTG NNK GAAATCTCCCTGCGTATG
W88x_R	CAAGTTTTCAGGGTATTTGAAACC
S91x_F	AACTTGTGGGAAATC NNK CTGCGTATGGCGC
S91x_R	GATTTCCCACAAGTTTTCAGG
R124x_F	GGCGTGATCCTGAAT NNK GATACCGAGCCGGC
R124x_R	ATTCAGGATCACGCCAAC
P128x_F	AATAGGGATACCGAG NNK GCCACGGCATTCCT
P128x_R	CTCGGTATCCCTATTCAGGA
**Round 2**	
S9x_F	catgcatgcatATGATTCGTGCGGTATTCTTTGAT NNK CTGGGTACTCTGATTAGC
L10x_F	CatgcatgcatATGATTCGTGCGGTATTCTTTGATAGC NNK GGTACTCTGATTAGCGTTG
Y20x_F	AGCGTTGAAGGCTTG NNK AAAGTGCATCTGAAAATTATGGA
Y20x_R	CAAGCCTTCAACGCT
V22x_F	GAAGGCTTGTATAAA NNK CATCTGAAAATTATGGAGGAAGTG
V22x_R	TTTATACAAGCCTTCAACGCTA
M27x_F	GTGCATCTGAAAATT NNK GAGGAAGTGCTGGGT
M27x_R	AATTTTCAGATGCACTTTATACAAGC
L42x_F	AACCCGAAAACCCTG NNK GACGAATACGAGAAACTG
L42x_R	CAGGGTTTTCGGGTT

Y45x_F	ACCCTGCTGGACGAA NNK GAGAAACTGGCTCGC
Y45x_R	TTCGTCCAGCAGG
E46x_F	CTGCTGGACGAATAC NNK AAACTGGCTCGCGAA
E46x_R	GTATTCGTCCAGCAGGG
A49x_F	GAATACGAGAAACTG NNK CGCGAAGCGTTCTCT
A49x_R	CAGTTTCTCGTATTCGTCCAG
P63x_F	GGCAAACCGTATCGT NNK CTGCGTGATATCCTGGA
P63x_R	ACGATACGGTTTGCCC
E69x_F	CTGCGTGATATCCTG NNK GAAGTAATGCGTAAACTGG
E69x_R	CAGGATATCACGCAGC
M72x_F	ATCCTGGAAGAAGTA NNK CGTAAACTGGCGGAAAA
M72x_R	TACTTCTTCCAGGATATCACG
G80x_F	CTGGCGGAAAAGTAC NNK TTCAAATACCCTGAAAACTTGTT
G80x_R	GTACTTTTCCGCCAGTTT
L87x_F	AAATACCCTGAAAAC NNK TTGGAAATCTCCCTGC
L87x_R	GTTTTCAGGGTATTTGAAACC
S91x_F	AACTTGTTGGAAATC NNK CTGCGTATGGCGC
S91x_R	GATTTCCAACAAGTTTTCAGG
L92x_F	TTGTTGGAAATCTCC NNK CGTATGGCGCAACG
L92x_R	GGAGATTTCCAACAAGTTTTC
A95x_F	ATCTCCCTGCGTATG NNK CAACGCTACGGCG
A95x_R	CATACGCAGGGAGATTTC
G99x_F	ATGGCGCAACGCTAC NNK GAGCTGTACCCGGAA
G99x_R	GTAGCGTTGCGCCATA
L122x_F	CACGTTGGCGTGATC NNK AATAGGGATACCGAGCC
L122x_R	GATCACGCCAACGT

R124x_F	GGCGTGATCCTGAAT NNK GATACCGAGCCGG
R124x_R	ATTCAGGATCACGCCA
P128x_F	AATAGGGATACCGAG NNK GCCACGGCATTCCT
P128x_R	CTCGGTATCCCTATTCAG
A129x_F	AGGGATACCGAGCCG NNK ACGGCATTCCTGGAC
A129x_R	CGGCTCGGTATCCC
A131x_F	ACCGAGCCGGCCACG NNK TTCCTGGACGCACT
A131x_R	CGTGGCCGGCT
F132x_F	GAGCCGGCCACGGCA NNK CTGGACGCACTGGG
F132x_R	TGCCGTGGCCG
S148x_F	GATTCCATCACCACG NNK GAAGAAGCTGGTTTCTTTAAAC
S148x_R	CGTGGTGATGGAATCG
**Round 3**	
E9x_F	catgcatgcatATGATTCGTGCGGTATTCTTTGAT NNK CTGGGTACTCTGATTAGC
L10x_F	catgcatgcatATGATTCGTGCGGTATTCTTTGATGAG NNK GGTACTCTGATTAGCGTTG
G11x_F	CatgcatgcatATGATTCGTGCGGTATTCTTTGATGAGCTG NNK ACTCTGATTAGCGTTGAA
T12x_F	CatgcatgcatATGATTCGTGCGGTATTCTTTGATGAGCTGGGT NNK CTGATTAGCGTTGAAGG
L13x_F	CatgcatgcatATGATTCGTGCGGTATTCTTTGATGAGCTGGGTACT NNK ATTAGCGTTGAAGGCTT
I14x_F	CatgcatgcatATGATTCGTGCGGTATTCTTTGATGAGCTGGGTACTCTG NNK AGCGTTGAAGGCTTG
G18x_F	CatgcatgcatATGATTCGTGCGGTATTCTTTGATGAGCTGGGTACTCTGATTAGCGTTGAA NNK TTGAGGAAAGTGCATCT
L19x_F	CatgcatgcatATGATTCGTGCGGTATTCTTTGATGAGCTGGGTACTCTGATTAGCGTTGAAGGC NNK AGGAAAGTGCATCTGAA
V22x_F	GAAGGCTTGAGGAAA NNK CATCTGAAAATTATGGAGGA
V22x_R	TTTCCTCAAGCCTTCAAC
H23x_F	GGCTTGAGGAAAGTG NNK CTGAAAATTATGGAGGAAGT
H23x_R	CACTTTCCTCAAGCCTT
I26x_F	AAAGTGCATCTGAAA NNK ATGGAGGAAGTGCTG

I26x_R	TTTCAGATGCACTTTCC
M27x_F	GTGCATCTGAAAATT NNK GAGGAAGTGCTGGGT
M27x_R	AATTTTCAGATGCACTTTATACAAGC
L42x_F	AACCCGAAAACCCTG NNK GACGAATACACGAAACT
L42x_R	CAGGGTTTTCGGGTT
A49x_F	GAATACACGAAACTG NNK CGCGAAGCGTTCT
A49x_R	CAGTTTCGTGTATTCGT
P60x_F	AACTATGCGGGCAAA NNK TATCGTCCGCTGC
P60x_R	TTTGCCCGCATAGTT
R65x_F	CCGTATCGTCCGCTG NNK GATATCCTGGAAGAAGTAATG
R65x_R	CAGCGGACGATACG
D66x_F	TATCGTCCGCTGCGT NNK ATCCTGGAAGAAGTAATG
D66x_R	ACGCAGCGGACGATA
L87x_F	AAATACCCTGAAAAC NNK TTGGAAATCTCCCTGC
L87x_R	GTTTTCAGGGTATTTGAAACC
L88x_F	TACCCTGAAAACTTG NNK GAAATCTCCCTGCGT
L88x_R	CAAGTTTTCAGGGTATTTG
S91x_F	AACTTGTTGGAAATC NNK CTGCGTATGGCGC
S91x_R	GATTTCCAACAAGTTTTCAGG
L92x_F	TTGTTGGAAATCTCC NNK CGTATGGCGCAACG
L92x_R	GGAGATTTCCAACAAGTTTTC
A95x_F	ATCTCCCTGCGTATG NNK CAACGCTACGGCG
A95x_R	CATACGCAGGGAGATTTC
V120x_F	AAATATCACGTTGGC NNK ATCCTGAATAGGGATACC
V120x_R	GCCAACGTGATATTTACC
L122x_F	CACGTTGGCGTGATC NNK AATAGGGATACCGAGCC

L122x_R	GATCACGCCAACGT
R124x_F	GGCGTGATCCTGAAT NNK GATACCGAGCCGG
R124x_R	ATTCAGGATCACGCCA
P128x_F	AATAGGGATACCGAG NNK GCCACGGCATTCCT
P128x_R	CTCGGTATCCCTATTCAG
A129x_F	AGGGATACCGAGCCG NNK ACGGCATTCCTGGAC
A129x_R	CGGCTCGGTATCCC
S148x_F	GATTCCATCACCACG NNK GAAGAAGCTGGTTTCTTTAAAC
S148x_R	CGTGGTGATGGAATCG

Supplementary Table 8: Primer sequences used to point mutant variants

Primers for point mutations	
$eS_NAr1.3$_D9A_F	CATGCATGCATATGATTCGTGCGGTATTCTTTGAT GCT CTGGGTACTCTGATTAGC
$eS_NAr1.3$_L19A_F	CATGCATGCATATGATTCGTGCGGTATTCTTTGATGAGCTGGGTACTCTGATTAGCG TTGAAGGCGCGAGGAAAGTGCATCTGAA
$eS_NAr1.3$_R23A_F	GGCTTGAGGAAAGTG GCT CTGAAAATTATGGAGGAAGTG
$eS_NAr1.3$_R124A_F	GGCGTGATCCTGAAT GCG GATACCGAGCCGG
$eS_NAr1.3$_D125A_F	GTGATCCTGAATAGG GCG ACCGAGCCGGCCA

Supplementary Table 9: Crystallographic data for compound (*R*)-25 (CDCC 2494693)

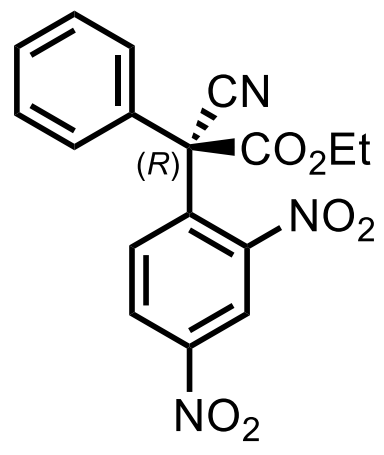

(*R*)-25
CDCC - 2494693

=

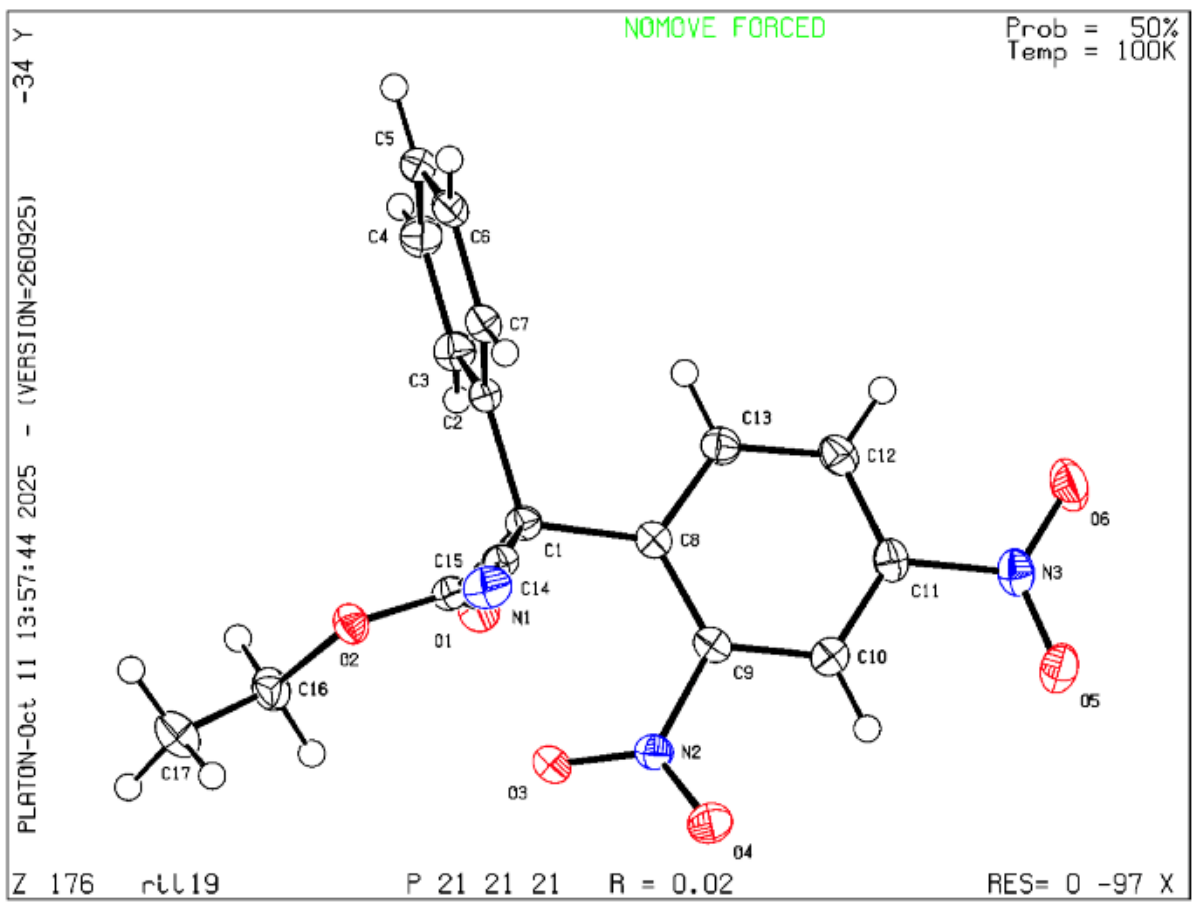

Empirical formula	$C_{17}H_{13}N_3O_6$
Formula weight	355.30
Temperature/K	100.00(10)
Crystal system	orthorhombic
Space group	$P2_12_12_1$
a/Å	7.89930(10)
b/Å	11.97720(10)
c/Å	17.01440(10)
α/°	90
β/°	90
γ/°	90
Volume/Å^3	1609.76(3)
Z	4
ρ_{calc}g/cm^3	1.466
μ/mm^{-1}	0.964
F(000)	736.0
Crystal size/mm^3	0.34 × 0.19 × 0.08
Radiation	Cu Kα (λ = 1.54184)
2Θ range for data collection/°	9.03 to 155.948
Index ranges	-9 ≤ h ≤ 10, -15 ≤ k ≤ 14, -20 ≤ l ≤ 20
Reflections collected	49050
Independent reflections	3337 [R_{int} = 0.0511, R_{sigma} = 0.0172]
Data/restraints/parameters	3337/0/236
Goodness-of-fit on F^2	1.088
Final R indexes [I>=2σ (I)]	R_1 = 0.0245, wR_2 = 0.0624
Final R indexes [all data]	R_1 = 0.0252, wR_2 = 0.0628
Largest diff. peak/hole / e Å^{-3}	0.15/-0.21
Flack parameter	0.00(4)

Supplementary Table 10: Data collection and refinement statistics. Entries in parentheses refer to statistics in the highest resolution bin.

	eS$_N$Ar1.3 **4** soak (K39A)
PDB accession code	9SX8
Wavelength (Å)	0.9763
Resolution range (Å)	54.08 – 2.10<br>(2.16-2.10)
Space group	P 3_1 2 1
Unit cell dimensions a, b, c, (Å) α, β, γ (°)	70.40, 70.40,117.12<br>90, 90, 120
Total reflections	788172 (65017)
Unique reflections	20243 (1619)
Multiplicity	38.9 (40.2)
Completeness (%)	100.0 (100.0)
Mean I/sigma(**I**)	29.9 (7.9)
Wilson B-factor (Å^2)	37.4
R-merge	0.06 (0.429)
R-meas	0.061 (0.435)
R-pim	0.010 (0.068)
$CC_{1/2}$	1.0 (0.988)
R-work	0.183 (0.197)
R-free	0.228 (0.269)
Number of non-hydrogen atoms	2099
macromolecules	1890
ligands	58
solvent	151
Protein residues	232
RMS(bonds) (Å)	0.003
RMS(angles) (°)	0.514
Ramachandran favoured (%)	98.26
Ramachandran allowed (%)	1.74
Ramachandran outliers (%)	0.0
Rotamer outliers (%)	0.49
Molprobity clashscore	2.0
Average B-factor (Å^2)	50.0

DNA and protein sequences for eS$_N$Ar1.3

eS$_N$Ar1.3

ATGATTCGTGCGGTATTCTTTGATGATCTGGGTACTCTGATTAGCGTTGAAGGCTTGAGG
AAAGTGCATCTGAAAATTATGGAGGAAGTGCTGGGTGACTATCCGCTGAACCCGAAAAC
CCTGCTGGACGAATACACGAAACTGGCTCGCGAAGCGTTCTCTAACTATGCGGGCAAAC
CGTATCGTCCGCTGGTTGATATCCTGGAAGAAGTAATGCGTAAACTGGCGGAAAAGTAC
GGTTTCAAATACCCTGAAAACTTGTTGGAAATCTCCCTGCGTATGGCGCAACGCTACGG
CGAGCTGTACCCGGAAGTGGTGGAAGTACTGAAATCTCTGAAAGGTAAATATCACGTTG
GCGTGATCCTGAATAGGGATACCGAGCCGGCCACGGCATTCCTGGACGCACTGGGCAT
CAAAGACCTGTTCGATTCCATCACCACGTCTGAAGAAGCTGGTTTCTTTAAACCGCACC
CACGCATCTTCGAACTGGCTCTGAAGAAAGCCGGCGTTAAAGGCGAGAAAGCAGTGTA
CGTTGGTGACAACCCGGTCAAAGACGCGGGTGGTTCTAAGAACCTGGGTATGACTAGC
ATCCTGCTGGATCGTAAAGGTGAGAAACGTGAATTCTGGGATAAGGCGGACTTTATCGT
CTCCGACCTGCGCGAAGTTATTAAGATTGTTGACGAACTGAACGGTCAGGGCTCTCTCG
AGCACCACCACCACCACCAC

MIRAVFFDDLGTLISVEGLRKVHLKIMEEVLGDYPLNPKTLLDEYTKLAREAFSNYAGKPYRP
LVDILEEVMRKLAEKYGFKYPENLLEISLRMAQRYGELYPEVVEVLKSLKGKYHVGVILNRDT
EPATAFLDALGIKDLFDSITTSEEAGFFKPHPRIFELALKKAGVKGEKAVYVGDNPVKDAGGS
KNLGMTSILLDRKGEKREFWDKADFIVSDLREVIKIVDELNGQGSLEHHHHHH

Preparative Scale Biotransformations

Preparation of ethyl (*S*)-2-cyano-2-(2,4-dinitrophenyl)propanoate (**(*S*)-3**)

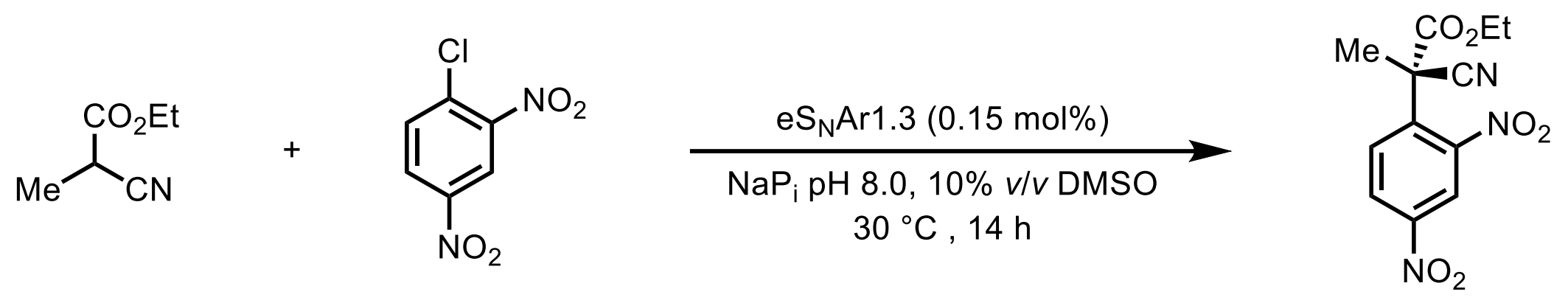

A 1 L Erlenmeyer flask was charged sequentially with **2** (162 mg, 0.80 mmol, 1.00 equiv), DMSO (32 mL) and **1** (1007 µL, 8.00 mmol, 10 equiv). The solution was gently swirled to homogeneity before the addition of eS$_N$Ar1.3 in NaP$_i$ pH 8.0 (288 mL, 0.0012 mmol, 0.15 mol%). The flask was sealed with a foam bung and foil and then incubated (30 °C, 120 r.p.m.) for 14 h. After this time, an aliquot (50 µL) of the reaction mixture was analysed by UPLC, showing 98% conversion. EtOAc (3 × 300 mL) was used to extract the reaction mixture then the combined organic phases washed with brine and dried over $MgSO_4$, followed by concentration in vacuo. The crude product was purified by flash column chromatography (SiO_2, celite dry loading, 15–25% EtOAc in hexane) to afford (*S*)-ethyl 2-cyano-2-(2,4-dinitrophenyl)propanoate (**(*S*)-3**) as a pale yellow solid (191 mg, 90% isolated yield, >99% *e.e.*). Spectroscopic data were consistent with that reported previously.[1]

^{1}H NMR: 400 MHz, $CDCl_3$
δ 9.00 (d, *J* = 2.4 Hz, 1H), 8.59 (dd, *J* = 8.7, 2.5 Hz, 1H), 8.05 (d, *J* = 8.7 Hz, 1H), 4.41 – 4.25 (m, 2H), 2.21 (s, 3H), 1.34 (t, *J* = 7.1 Hz, 3H).

^{13}C NMR: 101 MHz, $CDCl_3$
δ 166.1, 148.3, 148.0, 136.8, 131.2, 128.3, 121.9, 117.4, 64.4, 47.3, 24.7, 14.0

$[\alpha]_D^{24,3}$: –102.0° (c = 0.50, $CHCl_3$)

HRMS: (APCI$^+$) Calcd. for $C_{12}H_{12}N_3O_6$ ($[M+H]^+$): 294.0726, found: 294.0721

Preparation of ethyl (*R*)-2-cyano-2-(2,4-dinitrophenyl)-2-phenylacetate (**(*R*)-25**)

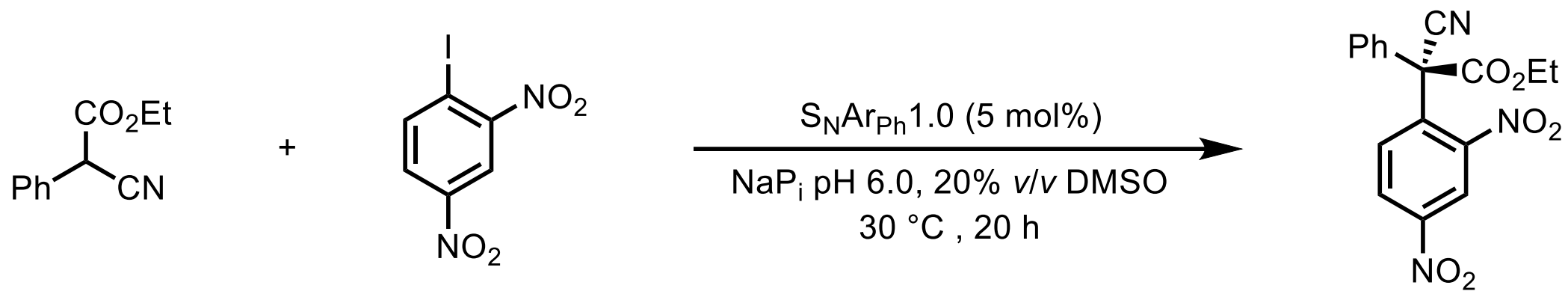

A 500 mL Erlenmeyer flask was charged sequentially with **5** (38.8 mg, 0.13 mmol, 1.00 equiv) in DMSO (13 mL), S_NAr_{Ph}1.0 in NaP_i pH 6.0 (106 mL at 62 μM, 50 μM final concentration) and ethyl 2-cyano-2-phenylacetate (46 μL, 0.26 mmol, 2 equiv) in DMSO (13 mL). The flask was sealed with a foam bung and foil and then shaken in an incubator (30 °C, 200 r.p.m.) for 20 h. After this time, an aliquot (50 μL) of the reaction mixture was taken, quenched and analysed by UPLC, showing 95% conversion of **5**. The reaction mixture was transferred to a separating funnel and extracted with EtOAc (3 × 100 mL), the organic layers were combined and washed with water (3 x 100 mL), brine and then dried over $MgSO_4$, followed by concentration *in vacuo*. The crude product was purified by flash column chromatography (SiO_2, loaded in CH_2Cl_2, 0–20% EtOAc in hexane) to afford enantioenriched (*R*)-ethyl 2-cyano-2-(2,4-dinitrophenyl)-2-phenylacetate (**(*R*)-25**) as an off-white solid (41 mg, 87% isolated yield, 86% *e.e.*). Spectroscopic data were consistent with the racemic standard.

Single crystals of **(*R*)-25** were obtained by slow evaporation from a saturated solution of hot EtOAc/hexane. Crystals of the same morphology (colourless, tetragonal) were used in X-ray diffraction studies for assignment of absolute configuration, [α] measurement chiral HPLC analysis (showed increase in enantiopurity to 98% *e.e.*).

^{1}H NMR: 400 MHz, $CDCl_3$
δ 9.01 (d, *J* = 2.5 Hz, 1H), 8.32 (dd, *J* = 8.7, 2.5 Hz, 1H), 7.67 – 7.47 (m, 5H), 7.07 (d, *J* = 8.8 Hz, 1H), 4.38 (dq, *J* = 10.6, 7.1 Hz, 1H), 4.23 (dq, *J* = 10.7, 7.1 Hz, 1H), 1.29 (t, *J* = 7.1 Hz, 3H).

^{13}C NMR: 101 MHz, $CDCl_3$
δ 165.3, 148.8, 148.2, 138.2, 133. 9, 132.2, 130.6, 130.2, 128.15, 127.6, 121.4, 115.7, 64.6, 56.8, 13.9

$[\alpha]_D^{24,9}$: –66.0 ° (c = 1.00, $CHCl_3$)

HRMS: (APCI$^+$) Calcd. for $C_{17}H_{14}N_3O_6$ ($[M+H]^+$): 356.0883, found: 356.0877

Chemical synthesis

Authentic, racemic product standards for compounds **3**, **6-9**, **15** and **16** were chemically synthesised according to procedures previously reported.[1]

Solvents used were of HPLC grade and anhydrous solvents over molecule sieves were purchased from Acros. Thin layer chromatography was performed on silica coated plates (Merck, silica 60 F_{254}) with detection by UVlight (λ = 254 nm) and/or by staining with a potassium permanganate solution [KMnO4]. Flash column chromatography was performed on silica (230–400 mesh, ASTM) with particle size 40–63 µm (SI 60) from Merck. Spectra from ^{1}H and ^{13}C NMR runs were recorded on a Bruker Avance 400 and 500 instruments (400 MHz for ^{1}H and 100 MHz for ^{13}C; 500 MHz for ^{1}H and 126 MHz for ^{13}C) in $CDCl_3$ using residual protic solvent as an internal standard. Reported chemical shifts (δ) (in parts per million (ppm)) are relative to the residual protic solvent signal ($CHCl_3$ in $CDCl_3$, ^{1}H = 7.26; ^{13}C = 77.0). Optical rotation measurements were performed on a Rudolph Autopol-I polarimeter at fixed 589 nm wavelength in a 0.5 cm polarimetry cell. Concentrations, *c*, where *c* = 1.00 corresponds to 10 mg mL^{-1}.

General procedure 1

A heat gun-dried round-bottomed flask equipped with a stirrer bar was charged sequentially with Na_2CO_3 (2.50 equiv), nucleophile (if solid, 1.05 equiv) and electrophile (if solid, 1.00 equiv) then placed under an atmosphere of N_2. Anhydrous DMF (0.2 M final concentration), nucleophile (if liquid, 1.05 equiv) and electrophile (if liquid, 1.00 equiv) were added sequentially then the reaction mixture heated at 60 °C for the specific time period. The reaction mixture was cooled to room temperature, quenched with 1 M HCl (30 mL per mmol), transferred into a separating funnel and extracted with EtOAc (3 × 15 mL per mmol). The combined organics were washed with H_2O (3 × 15 mL per mmol), brine then dried over $MgSO_4$ and concentrated *in vacuo*. The crude product was then purified by flash column chromatography.

General Procedure 2

A heat gun-dried round-bottomed flask equipped with a stirrer bar was charged with NaH (60% in dispersion oil), flushed with N_2 and then charged with THF (50% of total volume). The stirring suspension was cooled to 0 °C then the requisite nucleophile in THF (30% of total volume) added dropwise. The suspension was stirred at rt for 30 min, cooled to 0 °C and the requisite electrophile in THF (20% of total volume) was added dropwise. The reaction mixture was warmed to rt and stirred for overnight. The reaction mixture was quenched dropwise with 1 M HCl (10 mL per mmol), transferred to a separating funnel and extracted with EtOAc (3 × 10 mL per mmol). The organic phases were combined, washed with brine, dried over $MgSO_4$ and concentrated in vacuo. The crude product was then purified by flash column chromatography.

Preparation of ethyl 2-(2-chloro-4-nitrophenyl)-2-cyanopropanoate (**10**)

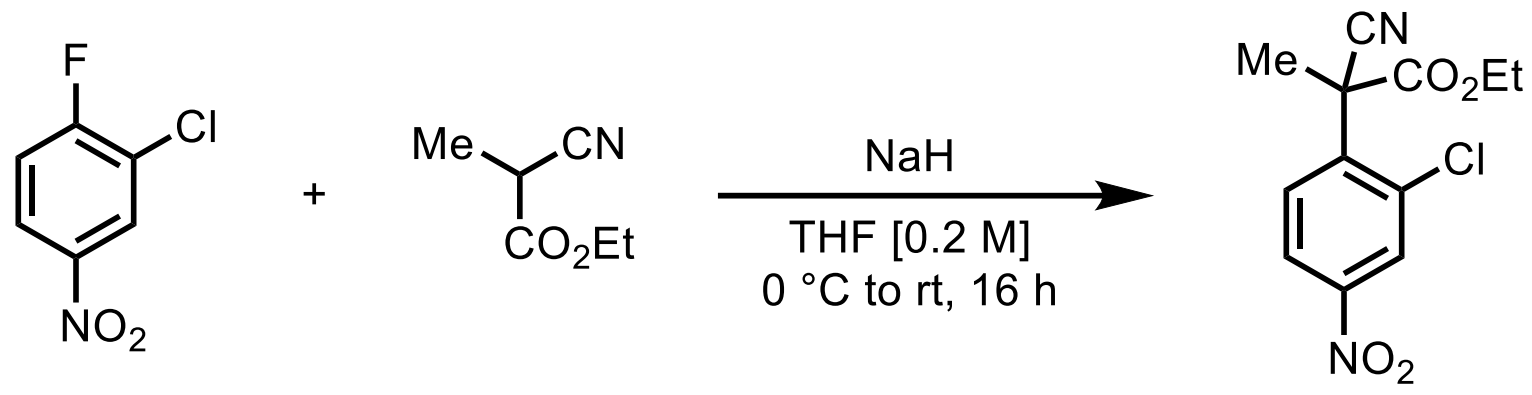

Following general procedure 2, floro-2-chloro-4-nitrobenzene (350 mg, 2.00 mmol, 1.00 equiv), ethyl-2-cyanopropanoate (559 µL, 4.40 mmol, 2.20 equiv) and NaH (176 mg (60% in dispersion oil), 4.40 mmol, 2.20 equiv), and were combined in anhydrous THF (10 mL) for 16 h at rt. Following the work-up described in general procedure 2, the crude product was purified by flash column chromatography (SiO_2, Celite dry-loading, 17% EtOAc in hexane) to afford the title compound as a yellow oil (43 mg, 8% yield).

Data for **10**:

TLC: R_f 0.24 (17% EtOAc in hexane) [UV]

^{1}H NMR: 400 MHz, $CDCl_3$
δ 8.33 (d, *J* = 2.4 Hz, 1H), 8.24 (dd, *J* = 8.7, 2.4 Hz, 1H), 7.82 (d, *J* = 8.7 Hz, 1H), 4.33 (q, *J* = 7.1 Hz, 2H), 2.11 (s, 3H), 1.31 (t, *J* = 7.1 Hz, 3H)

^{13}C NMR: 101 MHz, $CDCl_3$
δ 166.6, 140.05, 134.7, 129.2, 126.1, 122.4, 117.4, 77.35, 64.1, 47.6, 23.2, 13.8

HRMS: ($APCI^+$) Calcd. for $C_{12}H_{12}ClN_2O_4$ ($[M+H]^+$): 283.0407, found: 283.0480

Preparation of ethyl 2-(2-bromo-4-nitrophenyl)-2-cyanopropanoate (**11**)

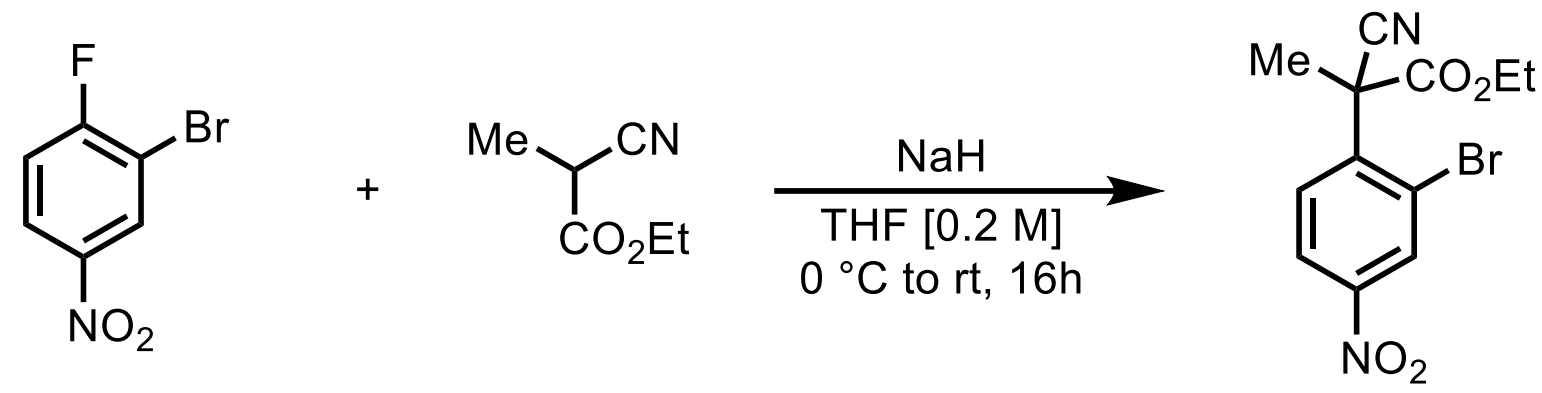

Following General Procedure 2, 2-bromo-1-fluoro-4-nitrobenzene (440 mg, 2.00 mmol, 1.00 equiv), ethyl 2-cyanopropanoate (553 µL, 559 mg, 4.40 mmol, 2.00 equiv) and NaH (60% in dispersion oil, 176 mg, 4.40 mmol, 2.20 equiv) were reacted in THF (10 mL) for 16 h. Following the work up described in General Procedure 2, the crude product was purified by flash column chromatography (SiO_2, Celite dry-loaded, 10-30% EtOAc in hexane) to afford the title compound as a pale yellow solid (242 mg, 37%).

Data for **11**:

TLC: R_f 0.24 (20% EtOAc in hexane) [UV]

m.p.: 83 °C (EtOAc)

^{1}H NMR: 400 MHz, $CDCl_3$
δ 8.52 (d, *J* = 2.4 Hz, 1H), 8.28 (dd, *J* = 8.7, 2.4 Hz, 1H), 7.79 (d, *J* = 8.7 Hz, 1H), 4.33 (q, *J* = 7.1 Hz, 2H), 2.14 (s, 3H), 1.32 (t, *J* = 7.1 Hz, 3H)

^{13}C NMR: 101 MHz, $CDCl_3$
δ 166.85, 148.3, 141.5, 129.65, 129.5, 124.0, 123.0, 117.55, 64.2, 49.2, 23.8, 13.9

HRMS: ($APCI^–$) Calcd for $C_{12}H_{12}N_2O_4Br$ ($[M–H]^+$): 326.9975, found: 326.9981

Preparation of ethyl 2-cyano-2-(4-nitro-2-(trifluoromethyl)phenyl)propanoate (**12**)

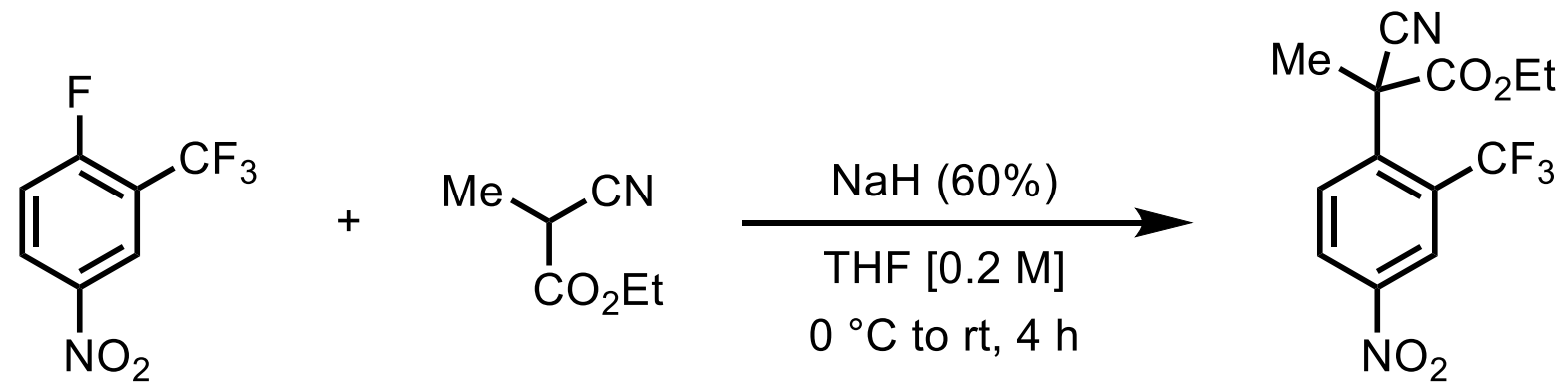

Following General Procedure 2, 1-fluoro-4-nitro-2-(trifluoromethyl)benzene (275 µL, 418 mg, 2.00 mmol, 1.00 equiv), NaH 60% in dispersion oil (120 mg, 3.00 mmol, 1.50 equiv) and ethyl 2-cyanopropanoate (377 µL, 381 mg, 3.00 mmol, 1.50 equiv) were reacted in THF (10 mL) for 4 h. Following the work-up as described in the General Procedure, the crude product was purified by column chromatography (SiO_2, Celite dry-loading, 15-25% EtOAc in hexane) to afford the title compound as a pale yellow solid (270 mg, 43%).

Data for **12**:

TLC: R_f 0.18 (15% EtOAc in hexane) [UV]

m.p.: 74 °C (EtOAc)

^{1}H NMR: 400 MHz, $CDCl_3$
8.64 (d, J = 2.5 Hz, 1H), 8.48 (dd, J = 8.8, 2.5 Hz, 1H), 8.00 (d, J = 8.8 Hz, 1H), 4.31 (q, J = 7.1 Hz, 2H), 2.18 (s, 3H), 1.30 (t, J = 7.1 Hz, 3H).

^{13}C NMR: 126 MHz, $CDCl_3$
δ 167.0, 148.1, 139.5, 130.75, 130.38 (q, J = 33.4 Hz), 127.1, 124.6 (q, J = 5.5 Hz), 123.0 (d, J = 274.9 Hz), 117.9, 64.3, 47.6, 25.5, 13.8

^{19}F NMR: 376 MHz, $CDCl_3$
δ –55.9

HRMS: (APCI$^+$) Calcd. for $C_{13}H_{12}F_3N_2O_4$ ([M+H]$^+$): 317.0749, found: 317.0744

Preparation of methyl 2-(2-cyano-1-ethoxy-1-oxopropan-2-yl)-5-nitrobenzoate (**13**)

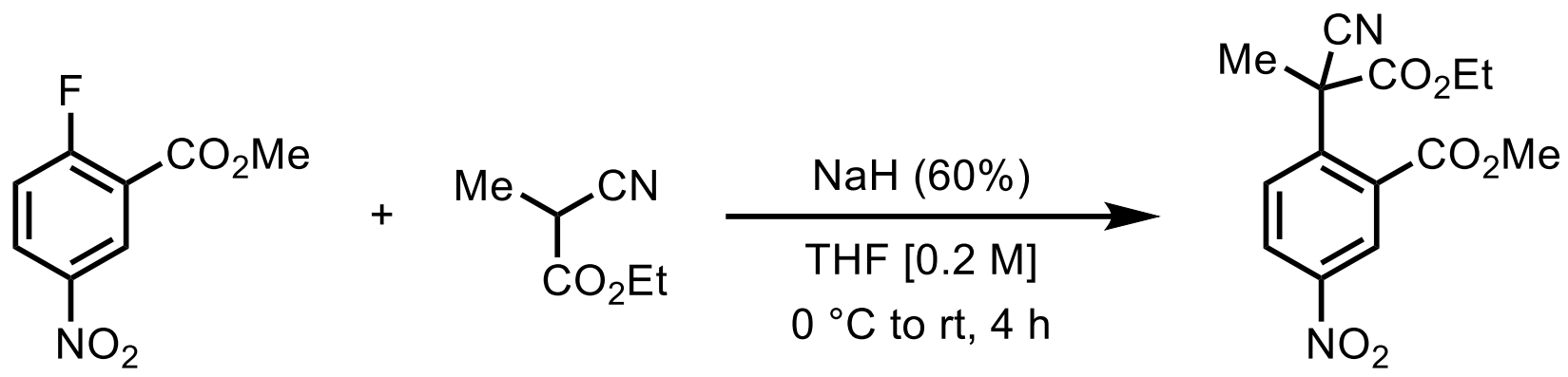

Following General Procedure 2, methyl 2-fluoro-5-nitrobenzoate (398 mg, 2.00 mmol, 1.00 equiv), NaH 60% in dispersion oil (120 mg, 3.00 mmol, 1.50 equiv) and ethyl 2-cyanopropanoate (377 µL, 381 mg, 3.00 mmol, 1.50 equiv) were reacted in THF (10 mL) for 4 h. Following the work-up as described in the General Procedure, the crude product was purified by column chromatography (SiO_2, Celite dry-loading, 10-20% EtOAc in hexane) to afford the title compound as a pale yellow oil (344 mg, 56%).

Data for **13**:

TLC: R_f 0.18 (15% EtOAc in hexane) [UV]

^{1}H NMR: 400 MHz, $CDCl_3$
δ 8.88 (d, J = 1.9 Hz, 1H), 8.44 (dd, J = 8.7, 2.1 Hz, 1H), 7.87 (d, J = 8.7 Hz, 1H), 4.36 – 4.22 (m, 2H), 3.97 (s, 3H), 2.16 (s, 2H), 1.31 (t, J = 7.1 Hz, 2H)

^{13}C NMR: 101 MHz, $CDCl_3$
δ 167.6, 165.4, 147.8, 142.7, 130.9, 129.5, 127.2, 126.85, 118.75, 63.5, 53.3, 47.65, 24.7, 13.9

HRMS: (APCI$^+$) Calcd for $C_{14}H_{15}O_6N_2$ ([M+H]$^+$): 307.0925, found: 307.0925

Preparation of ethyl 2-cyano-2-(2-cyano-4-nitrophenyl)propanoate (**14a**)

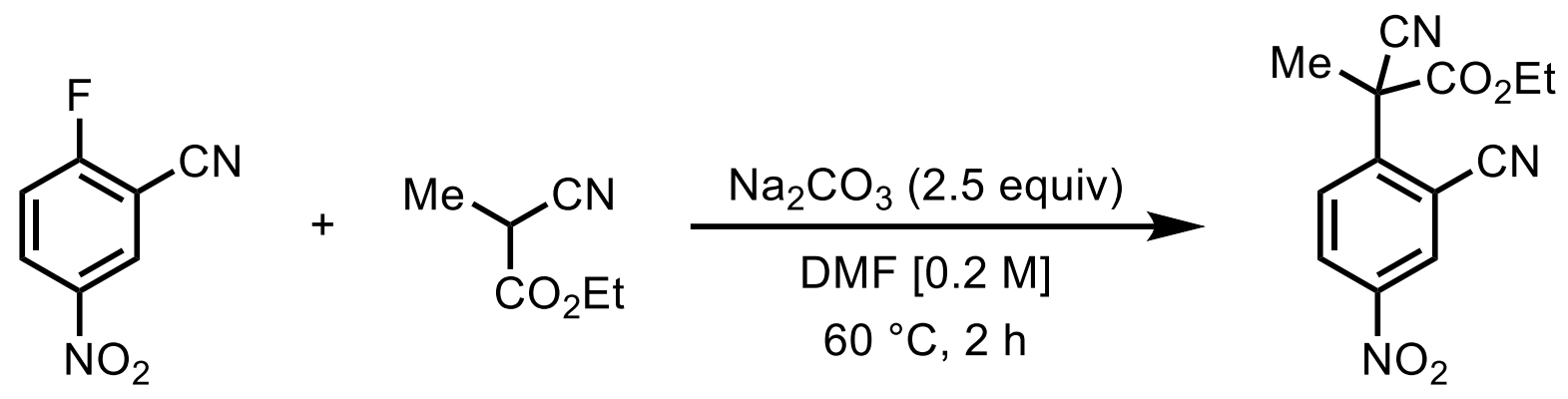

Following General Procedure 1, 2-fluoro-5-nitrobenzonitrile (166 mg, 1.00 mmol, 1.00 equiv), Na_2CO_3 (265 mg, 2.50 mmol, 2.50 equiv) and ethyl 2-cyanopropanoate (133 μL, 133 mg, 1.05 mmol, 1.05 equiv) were combined in DMF (5 mL) and stirred at 60 °C for 2 h. Following the work-up as described in the General Procedure, the crude product was purified by column chromatography (SiO_2, Celite dry-loading, 20-30% EtOAc in hexane) to afford the title compound as a pale yellow solid (101 mg, 37%).

Data for **14a**:

TLC: R_f 0.16 (20% EtOAc in hexane) [UV]

m.p. 68-70 °C (EtOAc)

^{1}H NMR: 400 MHz, $CDCl_3$
δ 8.61 (s, 1H), 8.53 (t, J = 8.7 Hz, 1H), 8.02 (d, J = 8.7 Hz, 1H), 4.46 – 4.35 (m, 2H), 2.18 (s, 3H), 1.36 (t, J = 7.1 Hz, 3H)

^{13}C NMR: 101 MHz, $CDCl_3$
δ 165.6, 147.8, 145.0, 129.9, 129.7, 128.2, 117.1, 114.8, 113.2, 64.9, 48.5, 24.3, 13.9

HRMS: (APCI$^+$) Calcd for $C_{12}H_{12}O_4N_3$ ($[M+H]^+$): 274.0828, found: 274.0831

Preparation of 2-(1-cyanoethyl)-5-nitrobenzonitrile (**14b**)

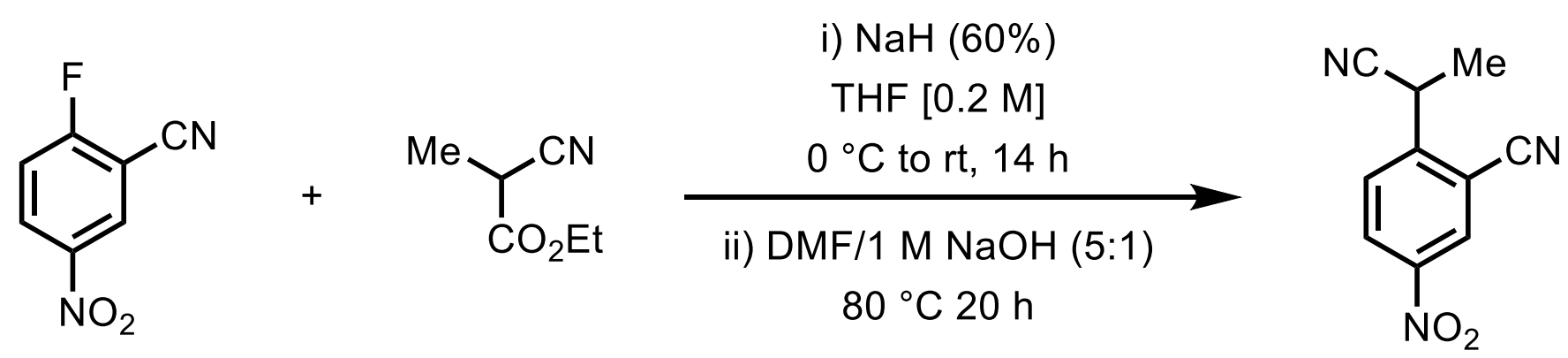

Following General Procedure 2, 2-fluoro-5-nitrobenzonitrile (332 mg, 2.00 mmol, 1.00 equiv), NaH 60% in dispersion oil (120 mg, 3.00 mmol, 1.50 equiv) and ethyl 2-cyanopropanoate (377 μL, 381 mg, 3.00 mmol, 1.50 equiv) were combined in THF (10 mL) and reacted for 16 h. Following the work-up as described in the General Procedure, the crude product contained **14a** and **14b** in a ~1.0:1.5 mixture that were inseparable by flash column chromatography.

To fully convert **14a** into **14b**, the crude material was dissolved in DMF (5 mL), transferred to a 20 mL microwave vial and 1 M NaOH was added. The vial was sealed, the solution sparged with N_2 for 10 min then heated at 80 °C for 20 h. The reaction was quenched with sat. NH_4Cl (20 mL), acidified to pH 1 with 2 M HCl then extracted with EtOAc (3 × 20 mL), washed sequentially with H_2O (3 × 20 mL) and brine then dried over $MgSO_4$. The crude product was purified by flash column chromatography (SiO_2, Celite dry-loading, 10-30% EtOAc in hexane) to afford the title compound as a yellow solid (68 mg, 17%).

Data for **14b**:

TLC: R_f 0.28 (30% EtOAc in hexane) [UV]

m.p. 94-95 °C (H_2O)

^{1}H NMR: 400 MHz, $CDCl_3$
δ 8.57 (s, 1H), 8.54 (d, J = 8.9 Hz, 1H), 7.97 (d, J = 8.6 Hz, 1H), 4.43 (q, J = 7.2 Hz, 1H), 1.77 (d, J = 7.2 Hz, 2H).

^{13}C NMR: 101 MHz, $CDCl_3$
δ 147.7, 147.15, 129.45, 128.7, 128.6, 118.9, 114.65, 113.1, 30.6, 21.2

HRMS: (APCI$^+$) Calcd for $C_5H_4N_2O_3Br$ ([M+H]$^+$): 202.0617, found: 202.0620

Preparation of ethyl 2-cyano-2-(6-(trifluoromethyl)pyrimidin-4-yl)propanoate (**17a**)

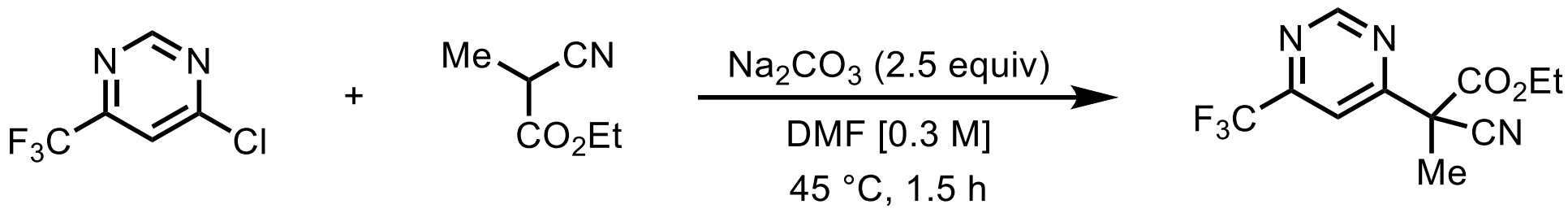

Following General Procedure 1, 4-chloro-6-(trifluoromethyl)pyrimidine (255 µL, 364 mg, 2.00 mmol, 1.00 equiv), Na_2CO_3 (515 mg, 5.00 mmol, 2.50 equiv) and ethyl 2-cyanopropanoate (377 µL, 381 mg, 3.00 mmol, 1.50 equiv) were combined in DMF (7 mL) and stirred at 45 °C for 1.5 h. Following the work-up as described in the General Procedure, the crude product was purified by column chromatography (SiO_2, Celite dry-loading, 5-15% EtOAc in hexane) to afford the title compound as a yellow oil (170 mg, 31%).

Data for **17a**:

TLC: R_f 0.34 (15% EtOAc in hexane) [UV]

^{1}H NMR: 400 MHz, $CDCl_3$
δ 9.38 (s, 1H), 7.97 (s, 1H), 4.30 (m, 2H), 2.03 (s, 2H), 1.28 (t, J = 7.1 Hz, 3H)

^{13}C NMR: 101 MHz, $CDCl_3$
δ 166.4, 165.3, 159.45, 157.45 (q, J = 36.9 Hz), 120.2 (q, J = 275.5 Hz), 117.4, 114.5 (q, J = 2.8 Hz), 64.4, 50.65, 23.4, 13.9

^{19}F NMR: 376 MHz, $CDCl_3$
δ -69.8

HRMS: (APCI$^+$) Calcd for $C_{11}H_{11}O_2N_3F_3$ ($[M+H]^+$): 274.0798, found: 274.0793

Preparation of 2-(6-(trifluoromethyl)pyrimidin-4-yl)propanenitrile (**17b**)

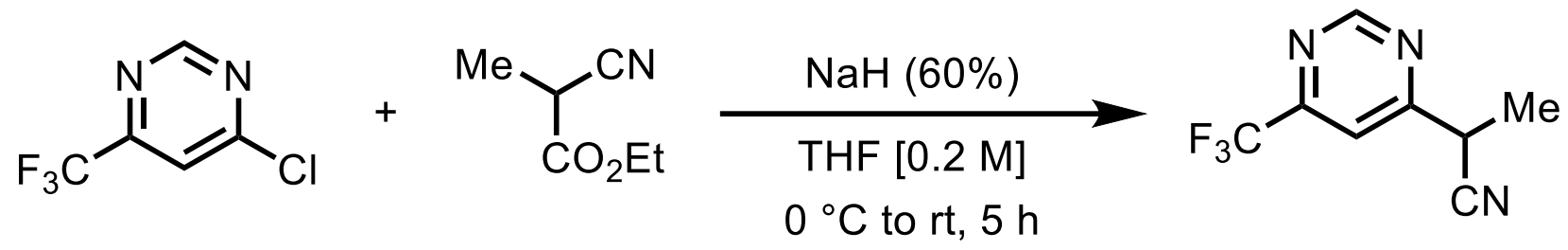

Following General Procedure 2**,** 4-chloro-6-(trifluoromethyl)pyrimidine (255 µL, 364 mg, 2.00 mmol, 1.00 equiv), NaH 60% in dispersion oil (120 mg, 3.00 mmol, 1.50 equiv) and ethyl 2-cyanopropanoate (377 µL, 381 mg, 3.00 mmol, 1.50 equiv) were combined in THF (10 mL) and reacted for 5 h. Following the work-up as described in the General Procedure, the crude product was purified by column chromatography (SiO_2, Celite dry-loading, 20% EtOAc in hexane) to afford the title compound as a yellow oil (120 mg, 30%).

Data for **17b**:

TLC: R_f 0.32 (20% EtOAc in hexane) [UV]

^{1}H NMR: 400 MHz, $CDCl_3$
δ 9.37 (s, 1H), 7.85 (s, 1H), 4.15 (q, *J* = 7.3 Hz, 1H), 1.78 (d, *J* = 7.3, 3H)

^{13}C NMR: 101 MHz, $CDCl_3$
δ 167.4, 159.63, 157.2 (q, *J* = 36.9 Hz), 120.3 (q, *J* = 275.4 Hz), 118.8, 114.6 (q, *J* = 2.8 Hz), 33.8, 19.0

^{19}F NMR: 376 MHz, $CDCl_3$
δ -69.9

HRMS: ($APCI^+$) Calcd for $C_8H_7F_3N_3$ ($[M+H]^+$): 202.0592, found: 202.0597

Preparation of ethyl 2-cyano-2-(6-cyanopyrimidin-4-yl)propanoate (**18a**) and 6-(1-cyanoethyl)pyrimidine-4-carbonitrile (**18b**)

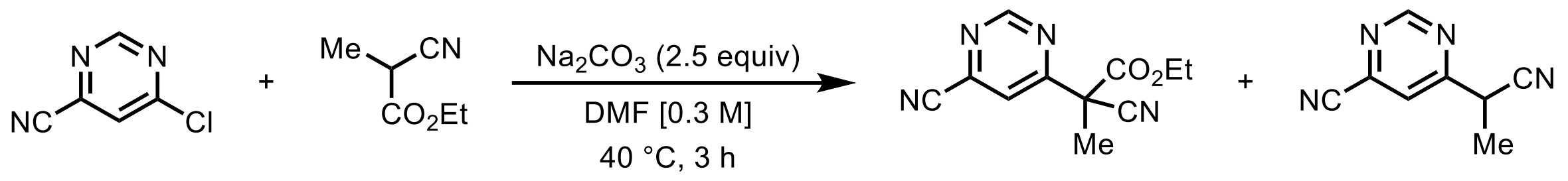

Following General Procedure 1, 6-chloropyrimidine-4-carbonitrile (185 µL, 278 mg, 2.00 mmol, 1.00 equiv), Na_2CO_3 (515 mg, 5.00 mmol, 2.50 equiv) and ethyl 2-cyanopropanoate (377 µL, 381 mg, 3.00 mmol, 1.50 equiv) were combined in DMF (7 mL) and stirred at 40 °C for 3 h. Following the work-up as described in the General Procedure, the crude product was purified by column chromatography (SiO_2, Celite dry-loading, 5-40% EtOAc in hexane) to afford **18a** as a pale yellow oil (130 mg, 28%) and **18b** as a bright orange solid (80 mg, 25%).

Data for **18a**:

TLC: R_f 0.28 (20% EtOAc in hexane) [UV]

^{1}H NMR: 400 MHz, $CDCl_3$
δ 9.37 (s, 1H), 7.99 (s, 1H), 4.40 – 4.23 (m, 2H), 2.02 (s, 3H), 1.29 (t, J = 6.7 Hz, 3H).

^{13}C NMR: 101 MHz, $CDCl_3$
δ 165.9, 165.1, 159.7, 143.1, 121.8, 117.1, 114.9, 64.6, 50.5, 23.3, 13.9

HRMS: (APCI$^+$) Calcd for $C_{11}H_{11}O_2N_4$ ($[M+H]^+$): 231.0882, found: 231.0886

Data for **18b**:

TLC: R_f 0.39 (40% EtOAc in hexane) [UV; visibly yellow]

mp: 120-122 °C (EtOAc)

^{1}H NMR: 400 MHz, $CDCl_3$
δ 9.35 (s, 2H), 7.86 (s, 2H), 4.12 (q, J = 7.4 Hz, 2H), 1.78 (d, J = 7.3 Hz, 6H)

^{13}C NMR: 101 MHz, $CDCl_3$
δ 167.0, 159.9, 142.8, 121.8, 118.5, 115.0, 33.6, 18.8

HRMS: (APCI$^+$) Calcd for $C_8H_7N_4$ ($[M+H]^+$): 159.0665, found: 159.0667

NMR spectra

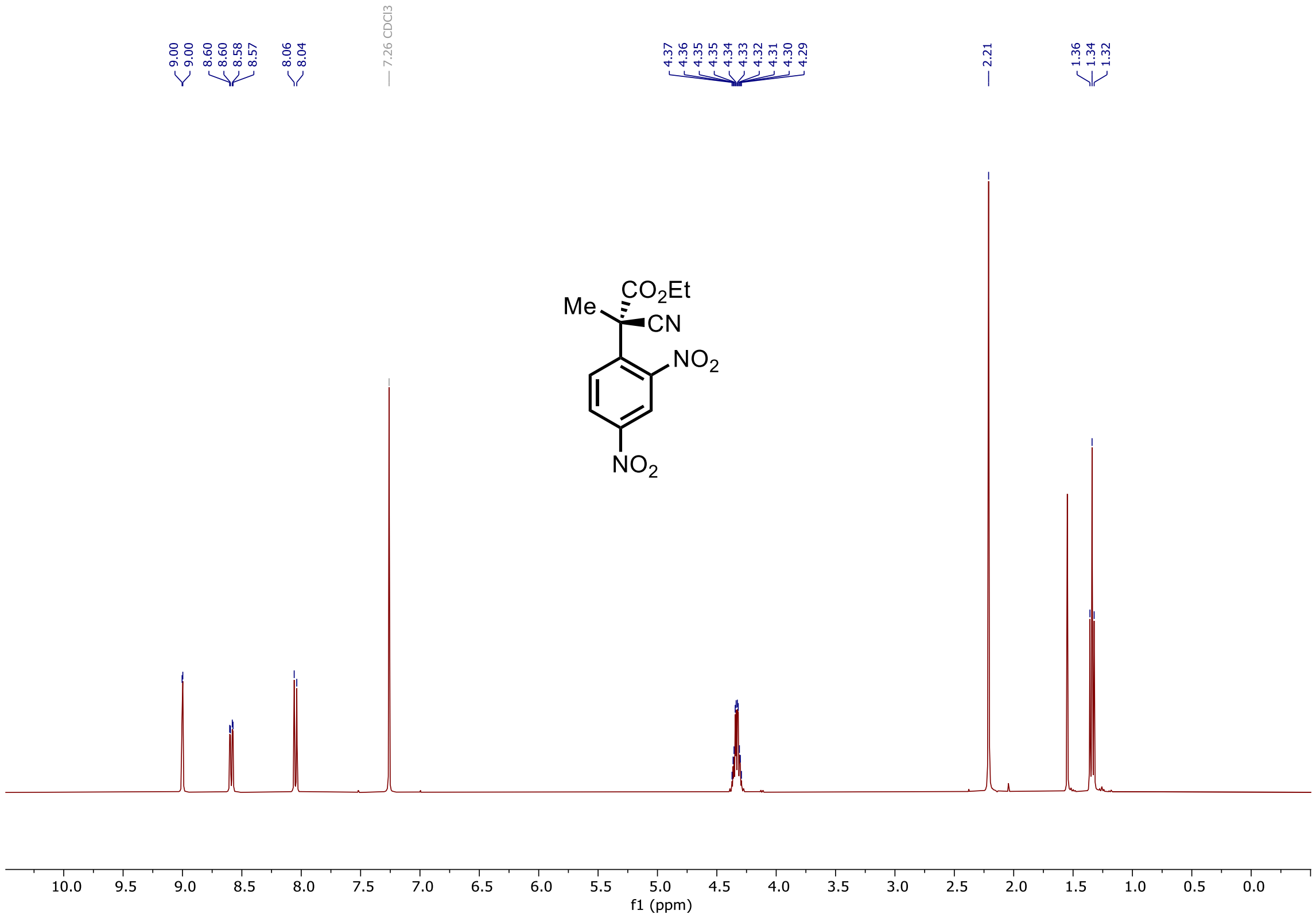

Supplementary Figure 32: ^{1}H NMR (400 MHz, $CDCl_3$) of **(*S*)-3** from a preparative scale biotransformation with eS_NAr1.3

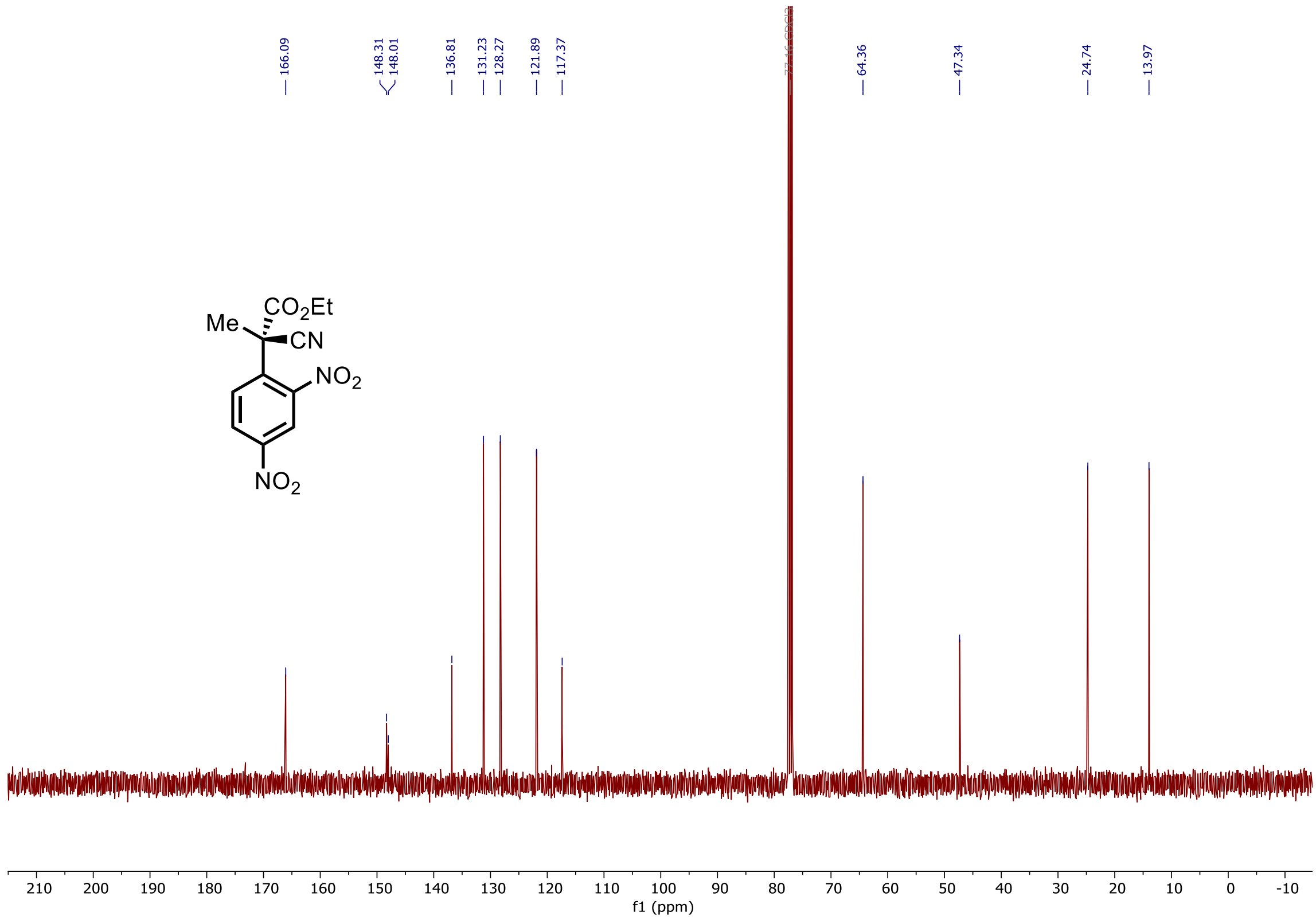

Supplementary Figure 33: $^{13}C\{^1H\}$ NMR (101 MHz, $CDCl_3$) of **(*S*)-3** from a preparative scale biotransformation with eS_NAr1.3

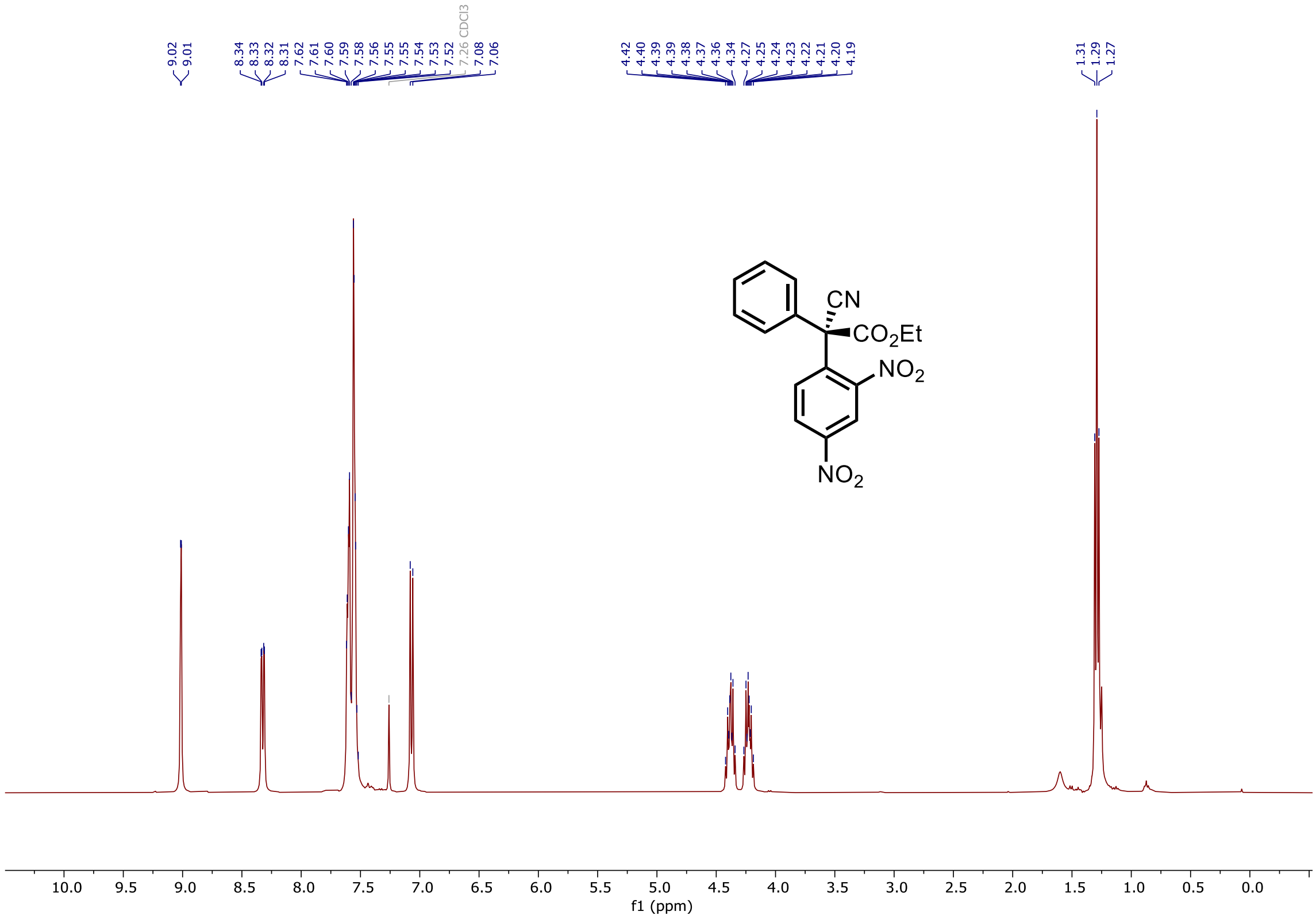

Supplementary Figure 34: ^{1}H NMR (400 MHz, $CDCl_3$) of ***(R)*-25** from a preparative scale biotransformation with $S_NAr_{Ph}1.0$

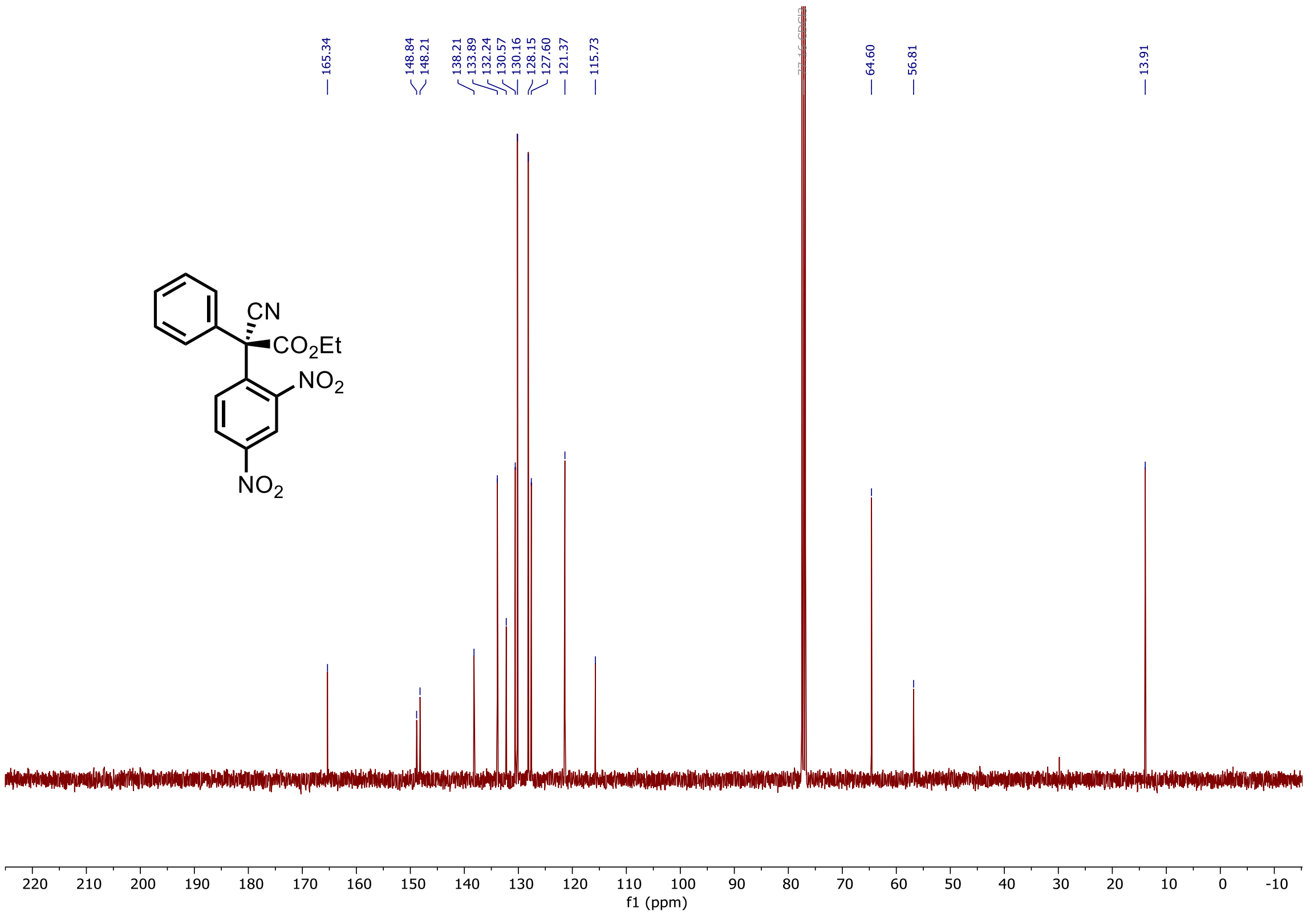

Supplementary Figure 35: $^{13}C\{^{1}H\}$ NMR (101 MHz, $CDCl_3$) of ***(R)*-25** from a preparative scale biotransformation with $S_NAr_{Ph}1.0$

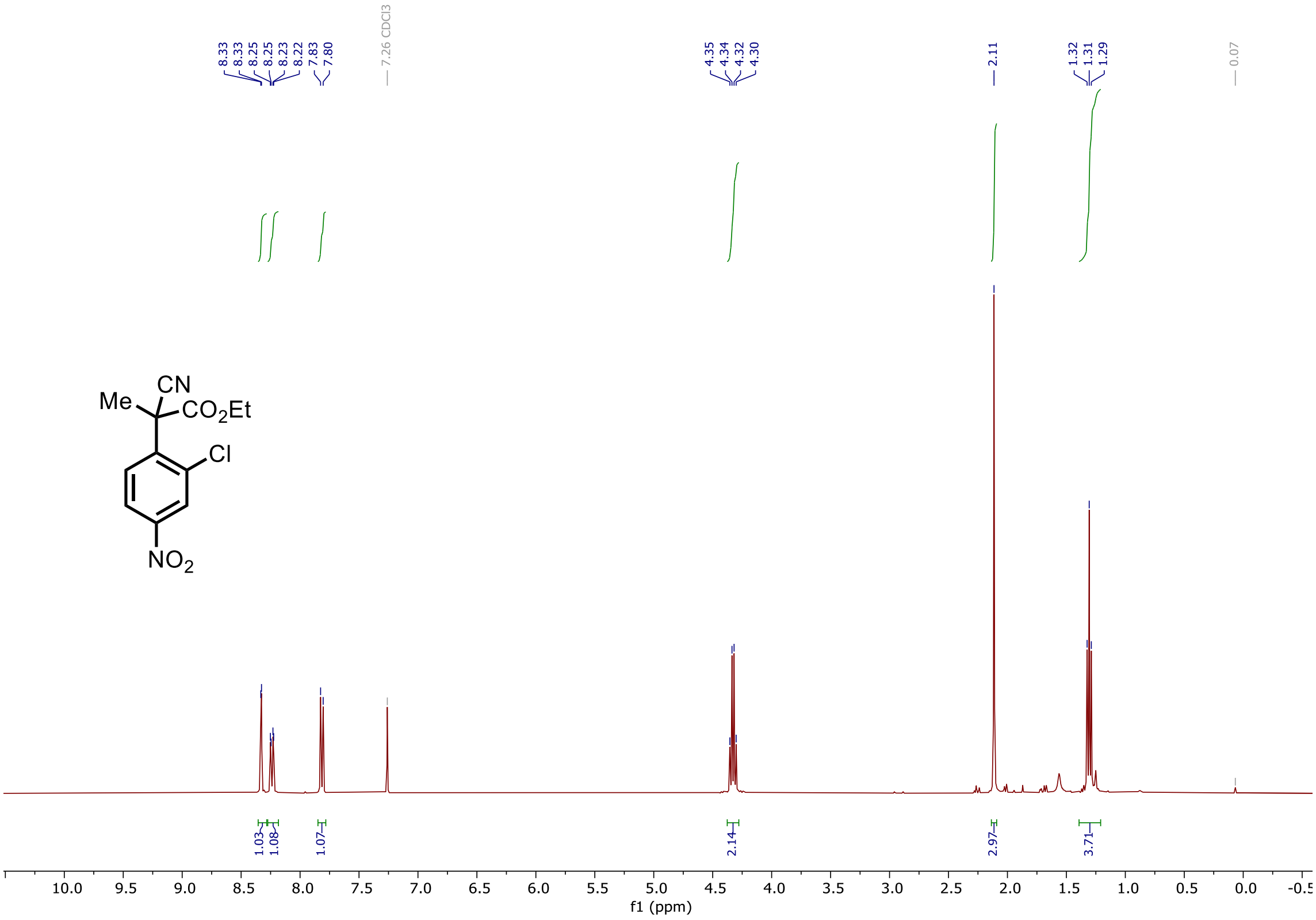

Supplementary Figure 36: ^{1}H NMR (400 MHz, $CDCl_3$) of **10**

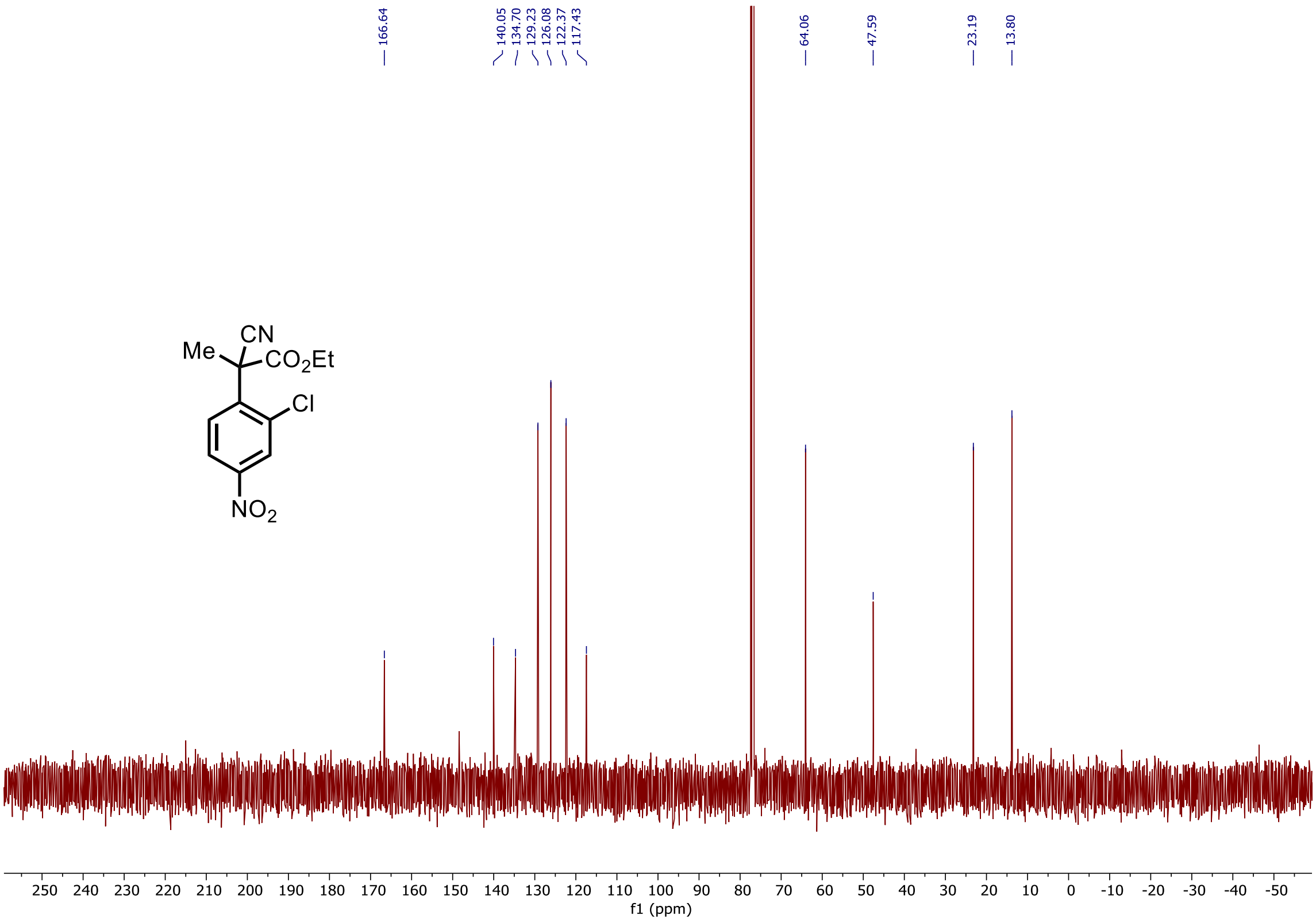

Supplementary Figure 37: $^{13}C\{^{1}H\}$ NMR (101 MHz, $CDCl_3$) of **10**

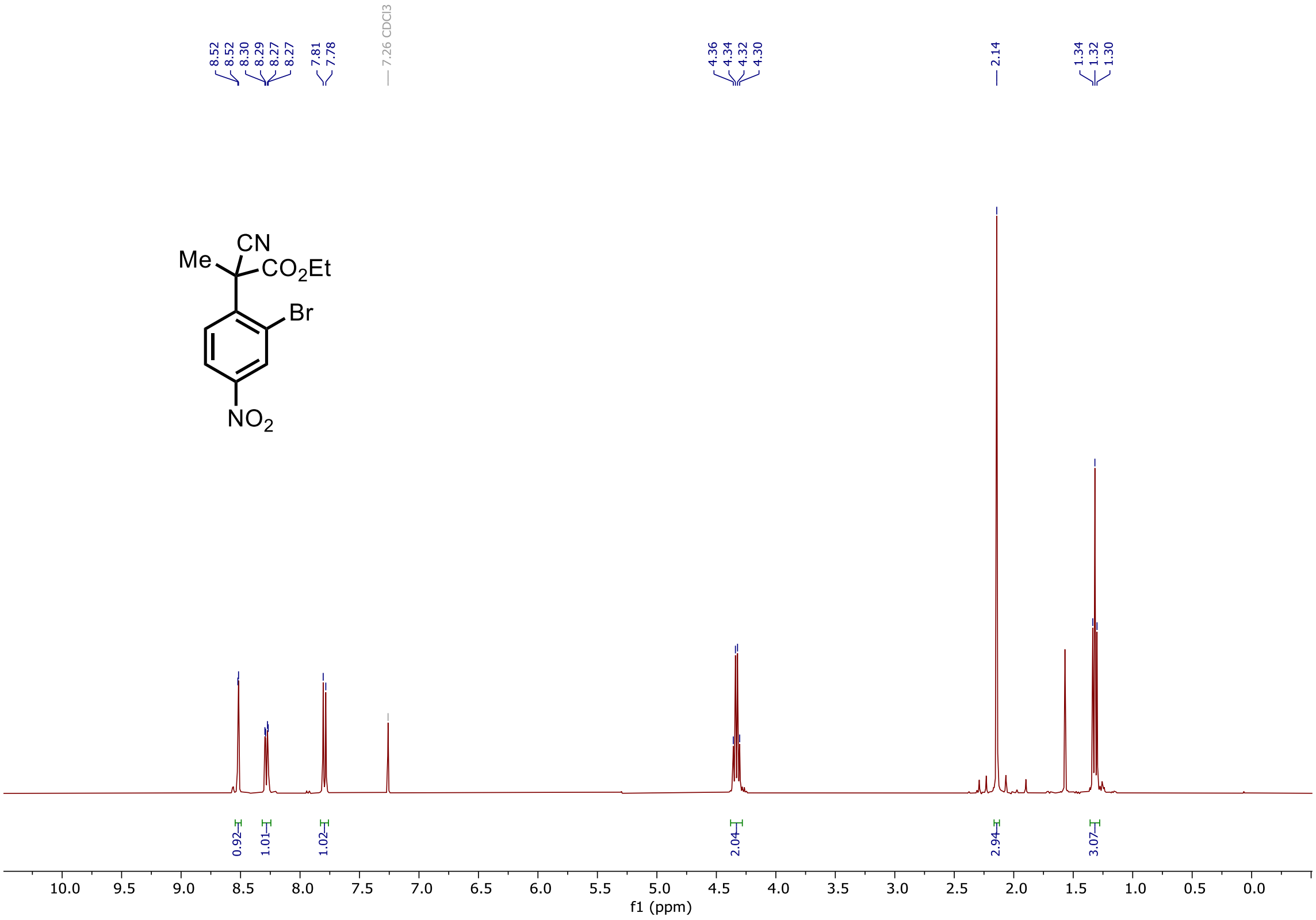

Supplementary Figure 38: ^{1}H NMR (400 MHz, $CDCl_3$) of **11**

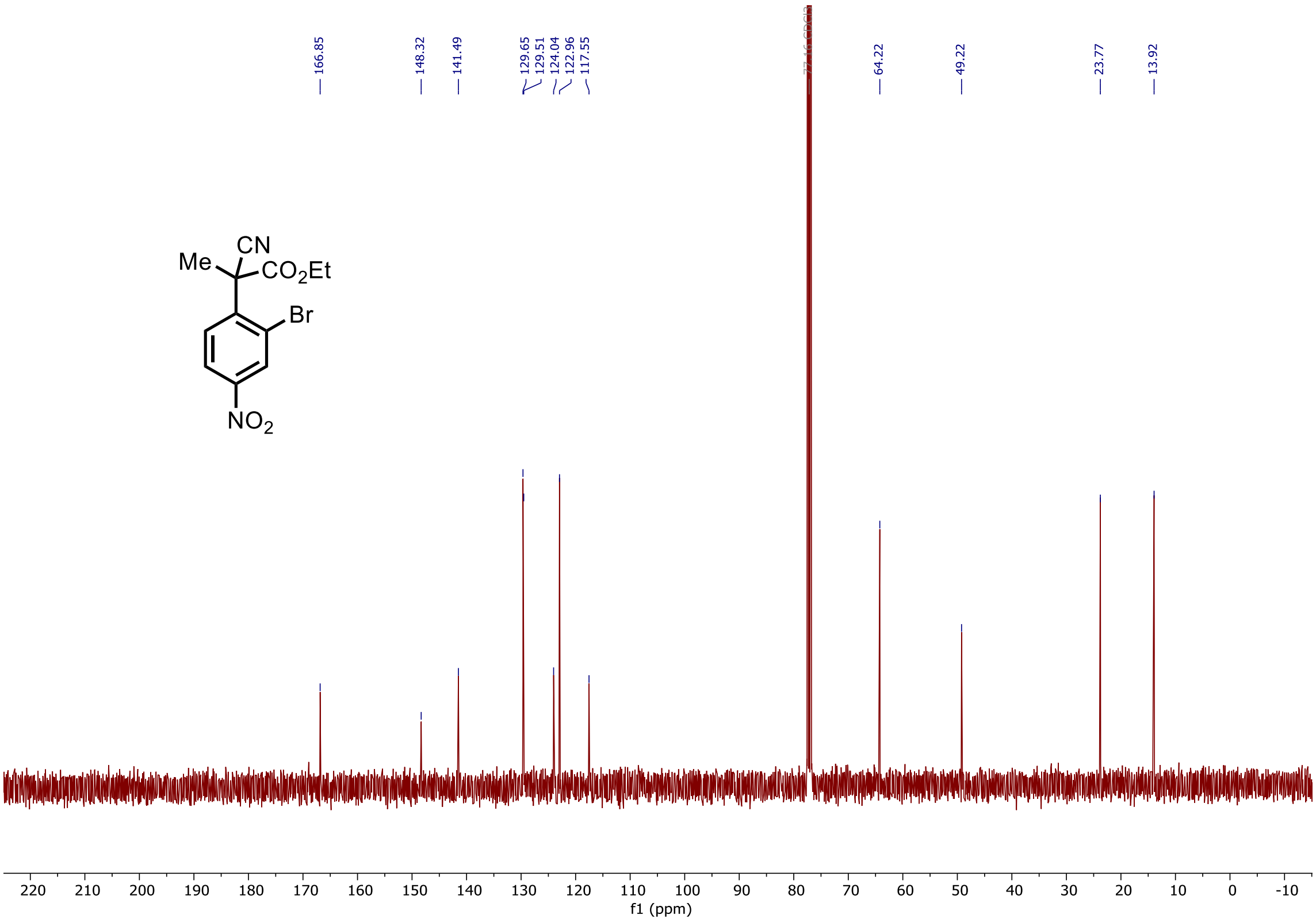

Supplementary Figure 39: $^{13}C\{^{1}H\}$ NMR (101 MHz, $CDCl_3$) of **11**

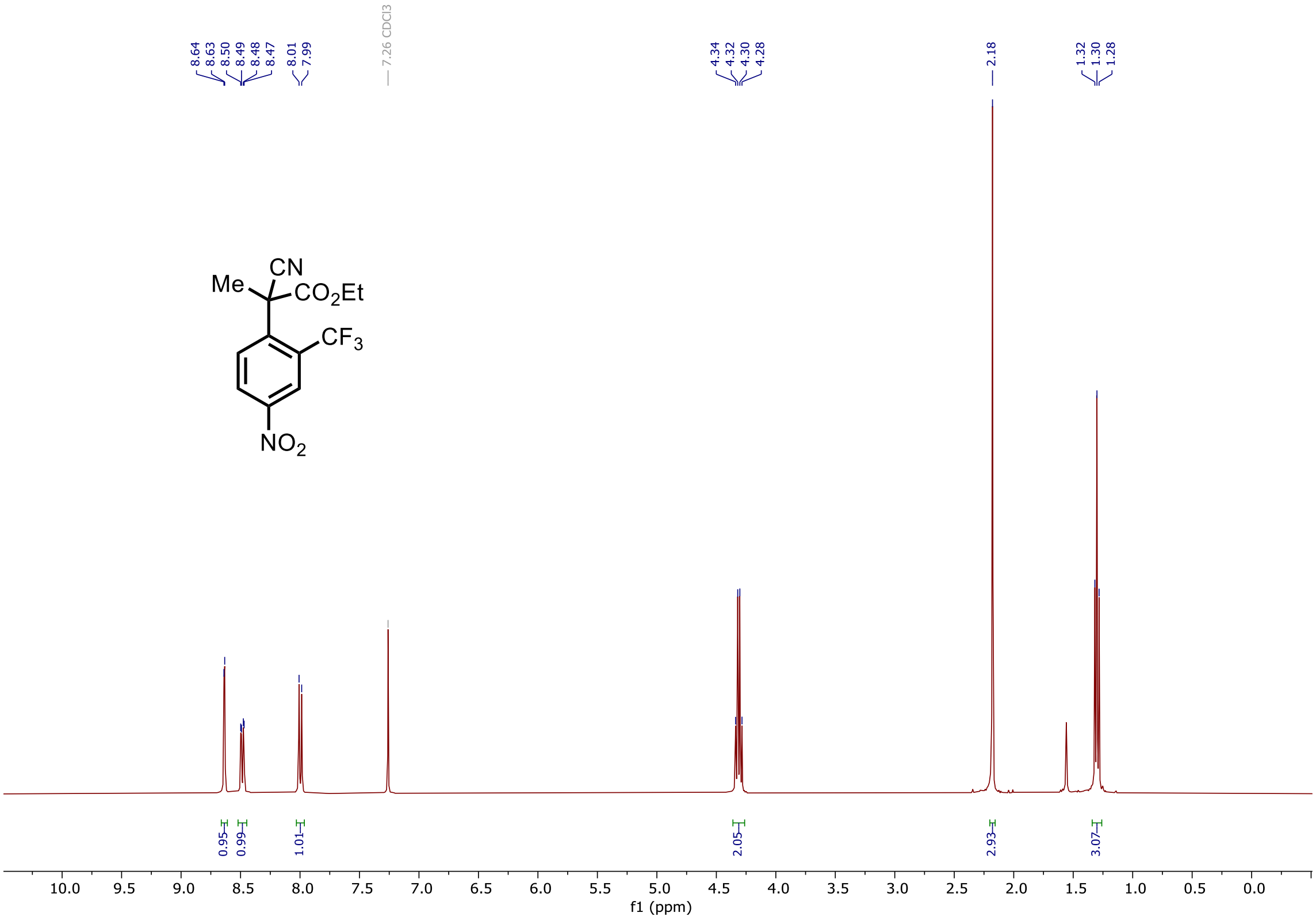

Supplementary Figure 40: ^{1}H NMR (400 MHz, $CDCl_3$) of **12**

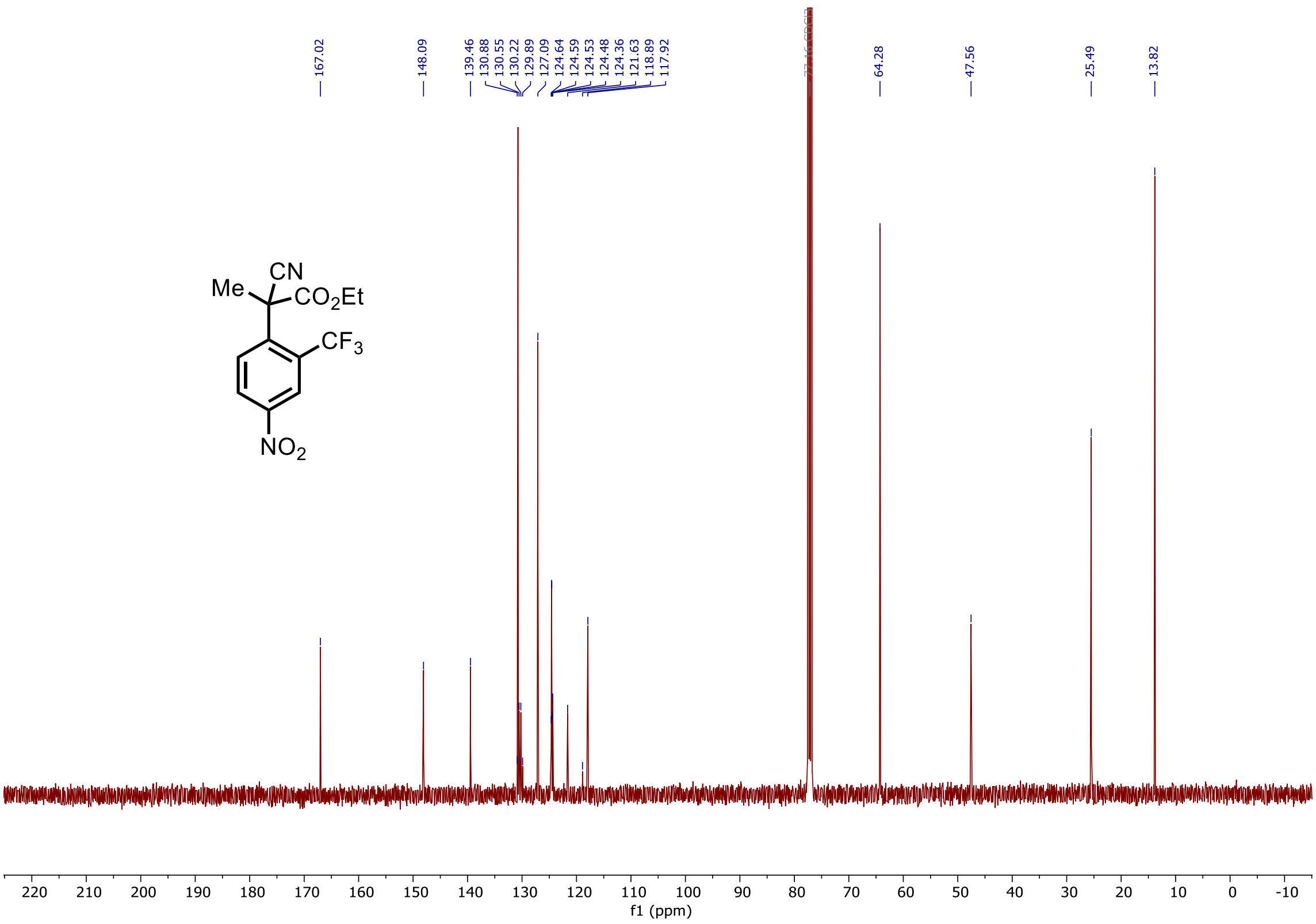

Supplementary Figure 41: $^{13}C\{^{1}H\}$ NMR (101 MHz, $CDCl_3$) of **12**

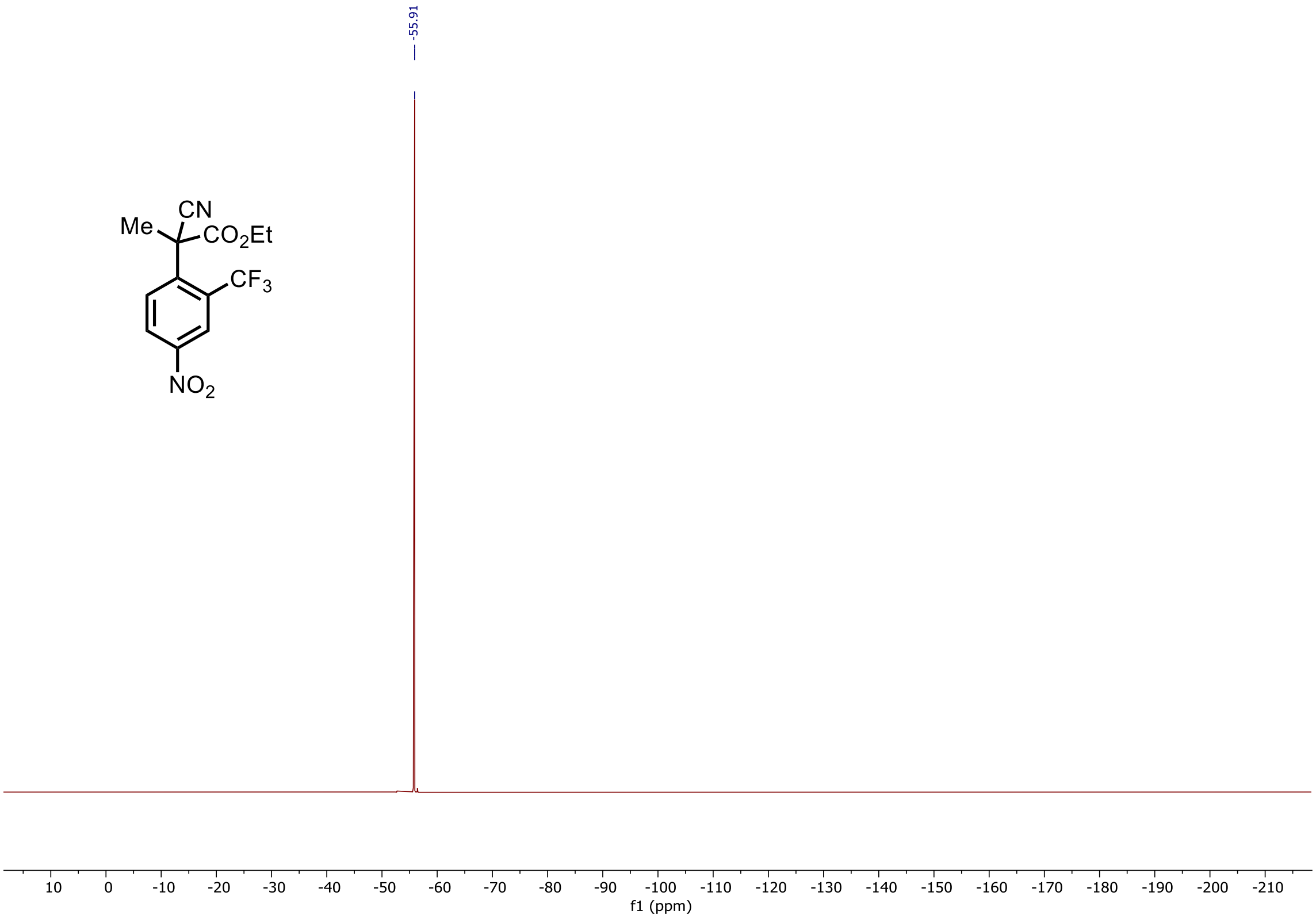

Supplementary Figure 42: $^{19}F\{^{1}H\}$ NMR (376 MHz, $CDCl_3$) of **12**

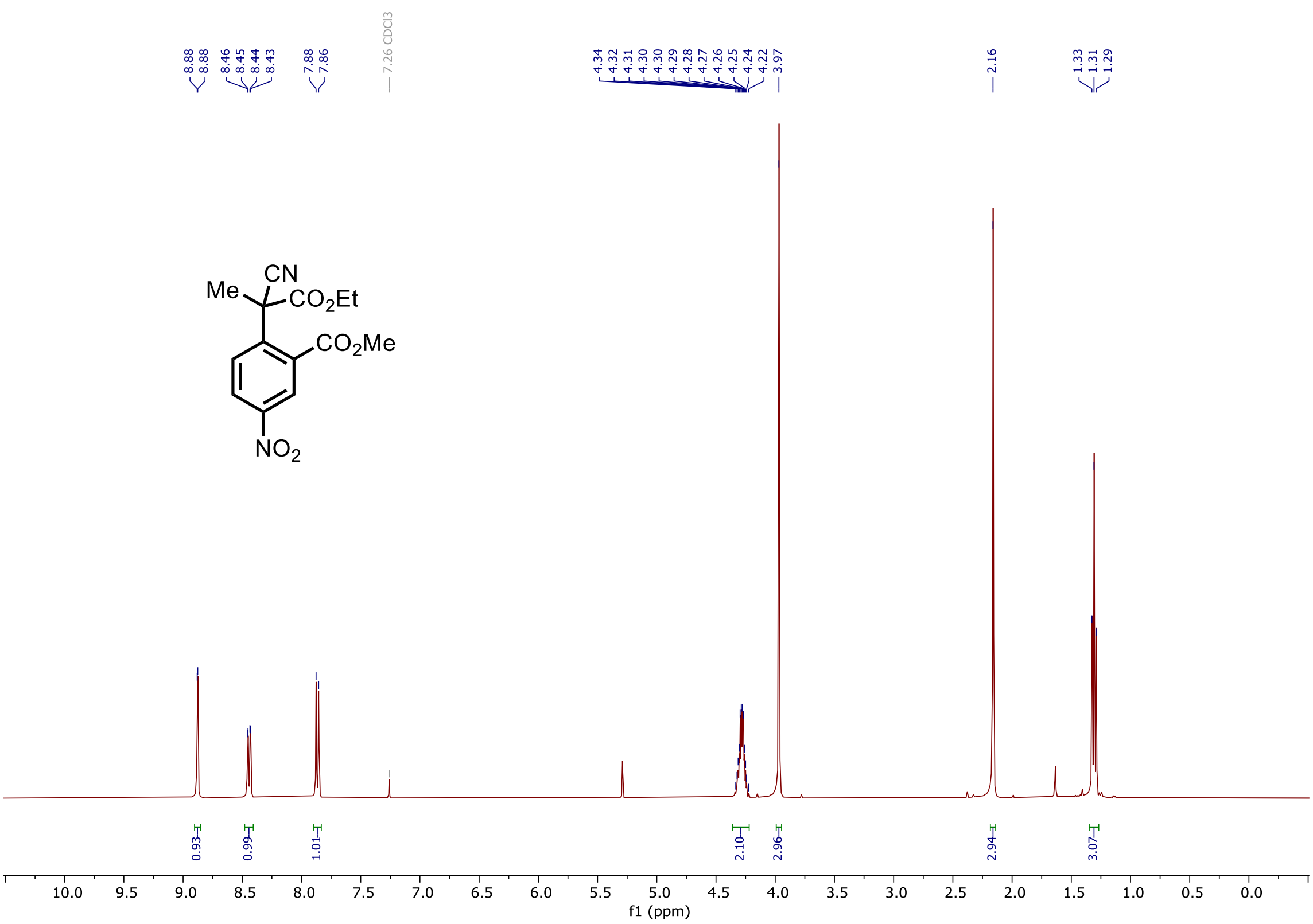

Supplementary Figure 43: ^{1}H NMR (400 MHz, $CDCl_3$) of **13**

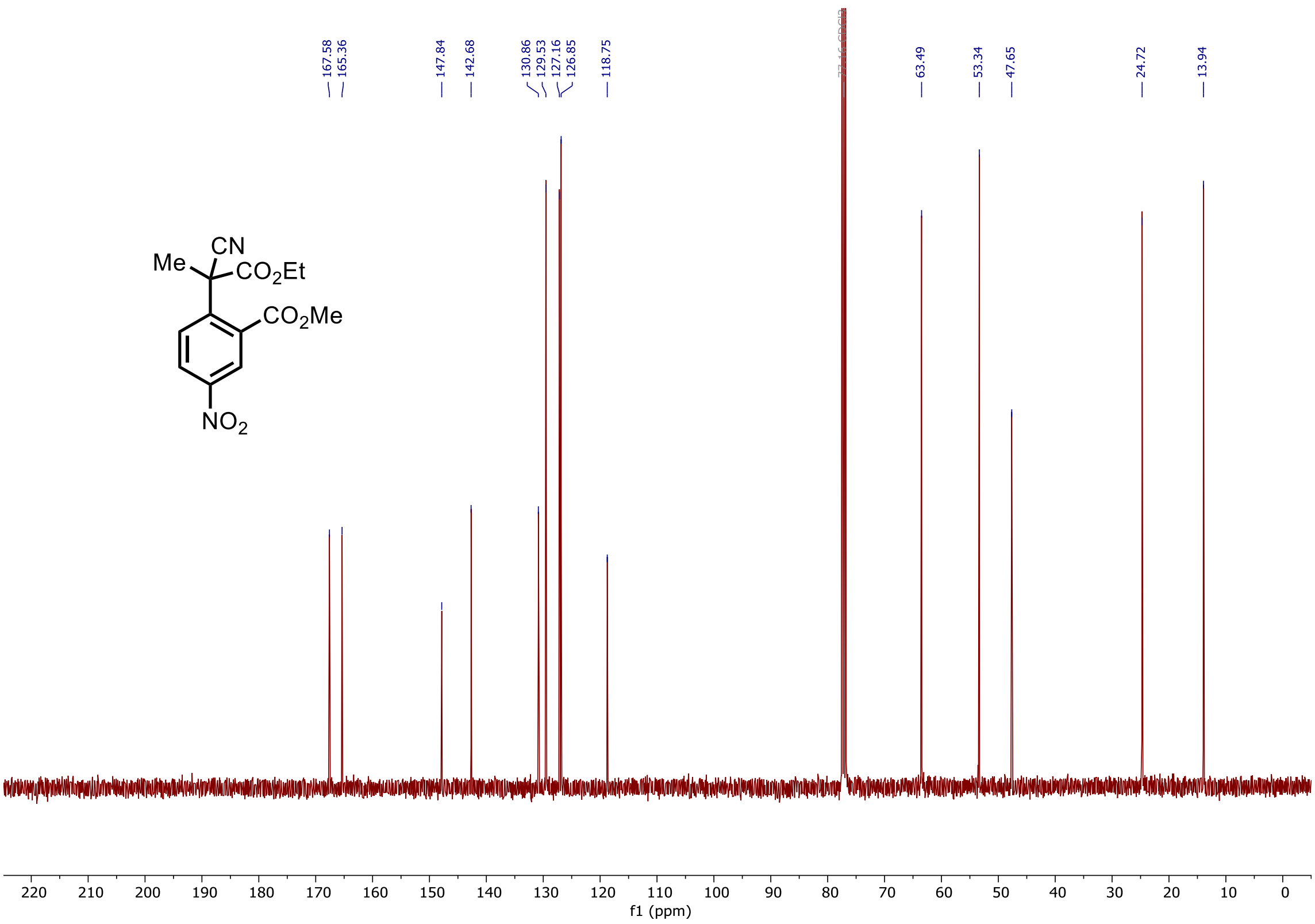

Supplementary Figure 44: $^{13}C\{^{1}H\}$ NMR (101 MHz, $CDCl_3$) of **13**

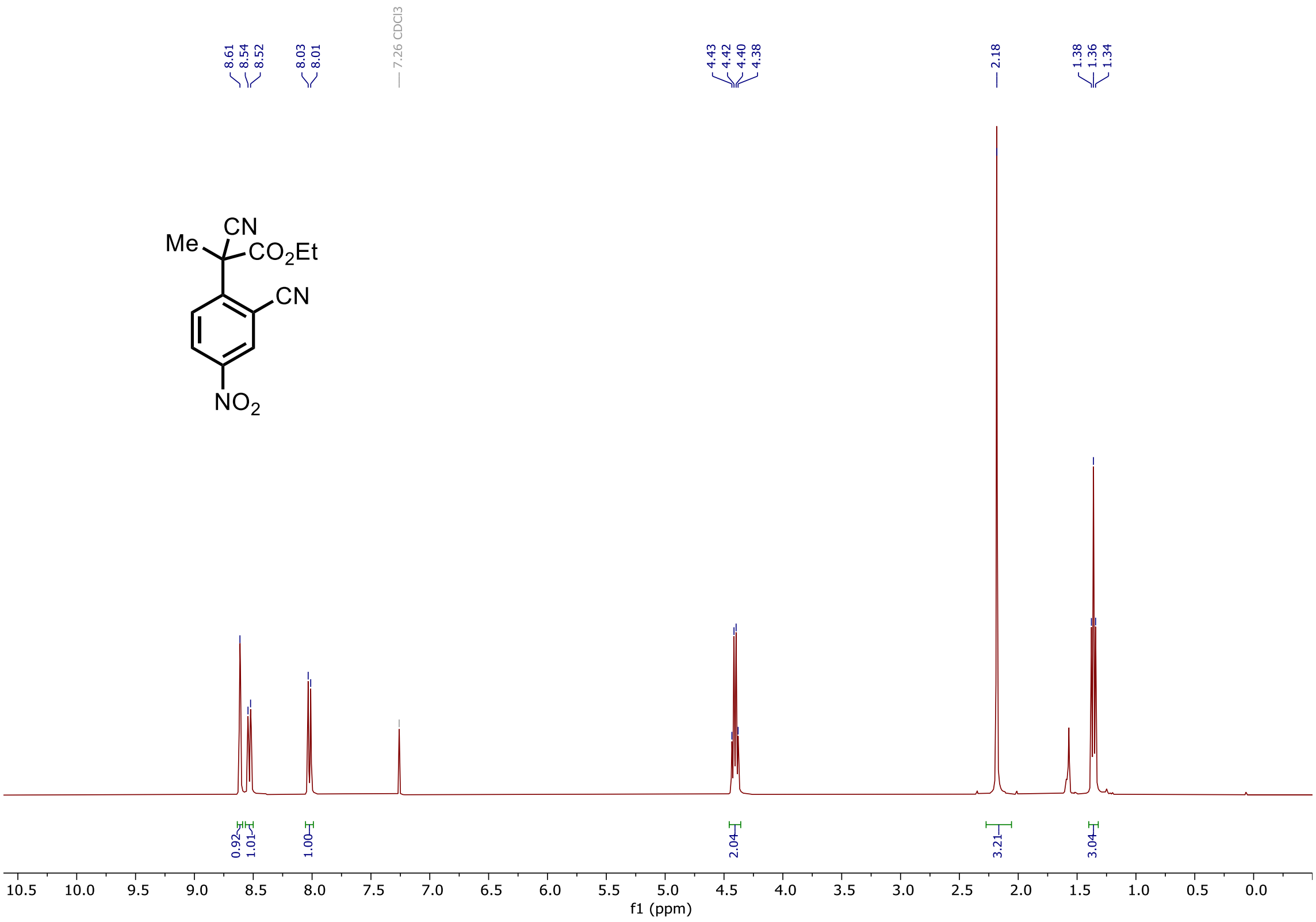

Supplementary Figure 45: ^{1}H NMR (400 MHz, $CDCl_3$) of **14a**

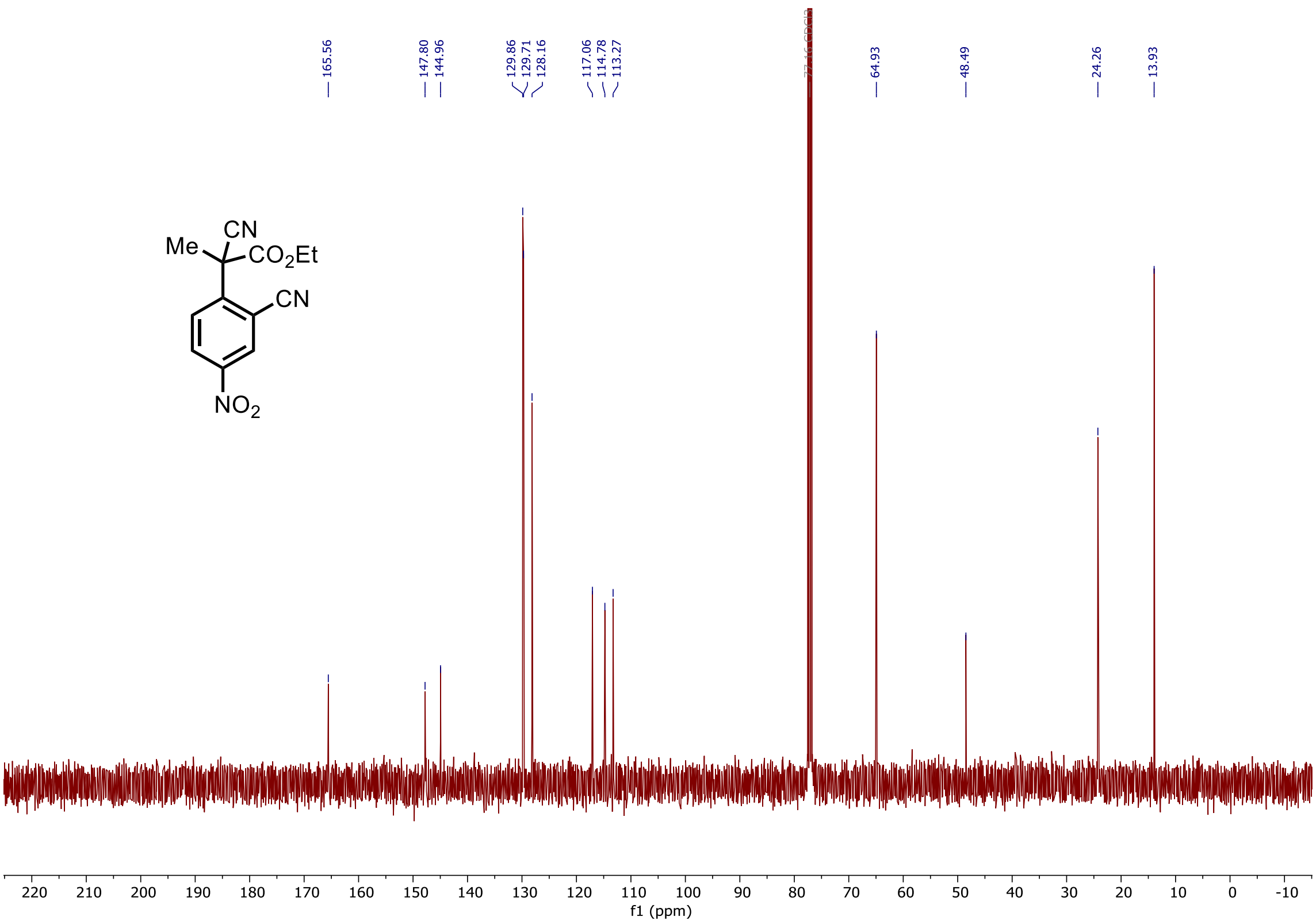

Supplementary Figure 46: $^{13}C\{^{1}H\}$ NMR (101 MHz, $CDCl_3$) of **14a**

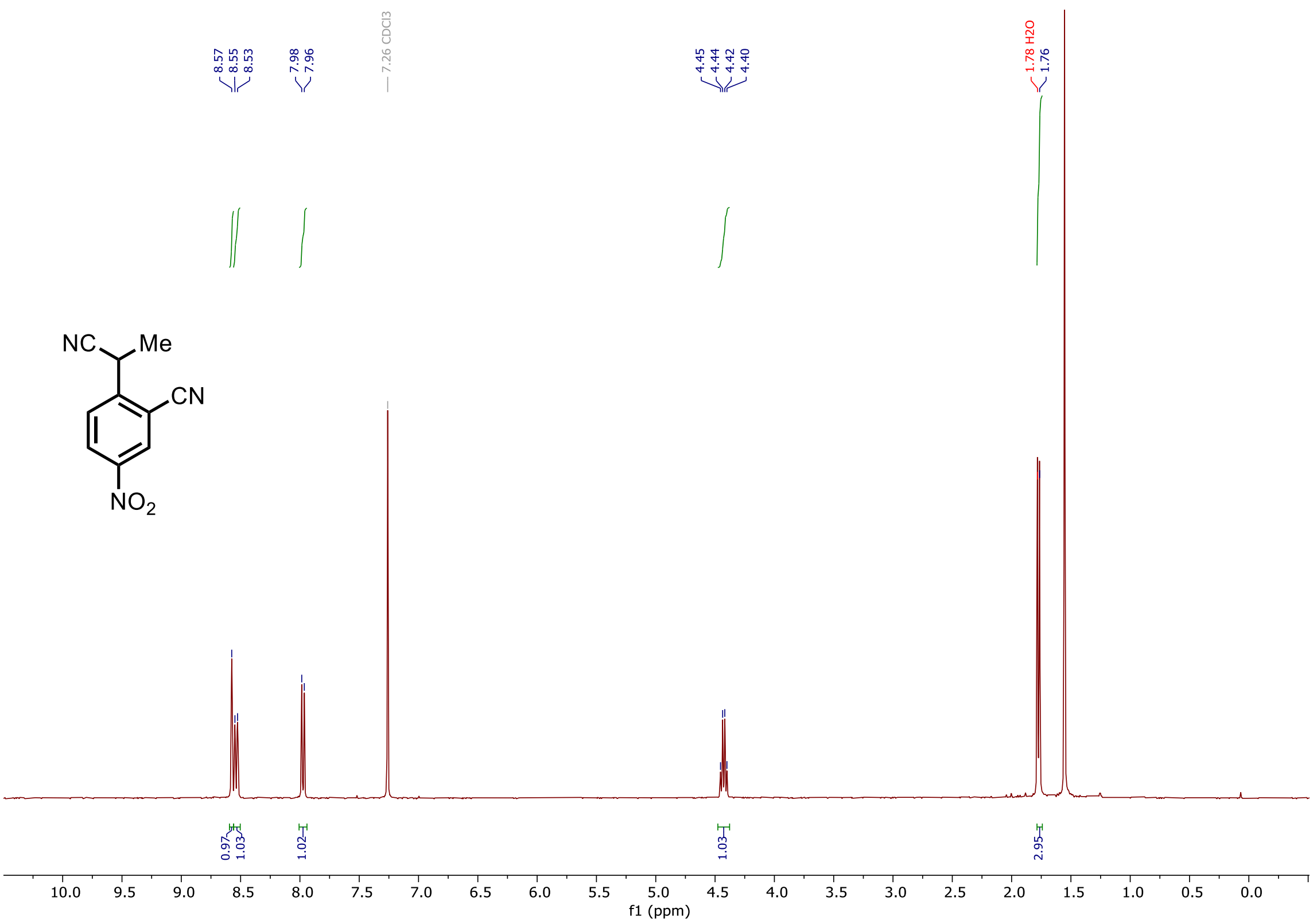

Supplementary Figure 47: ^{1}H NMR (400 MHz, $CDCl_3$) of **14b**

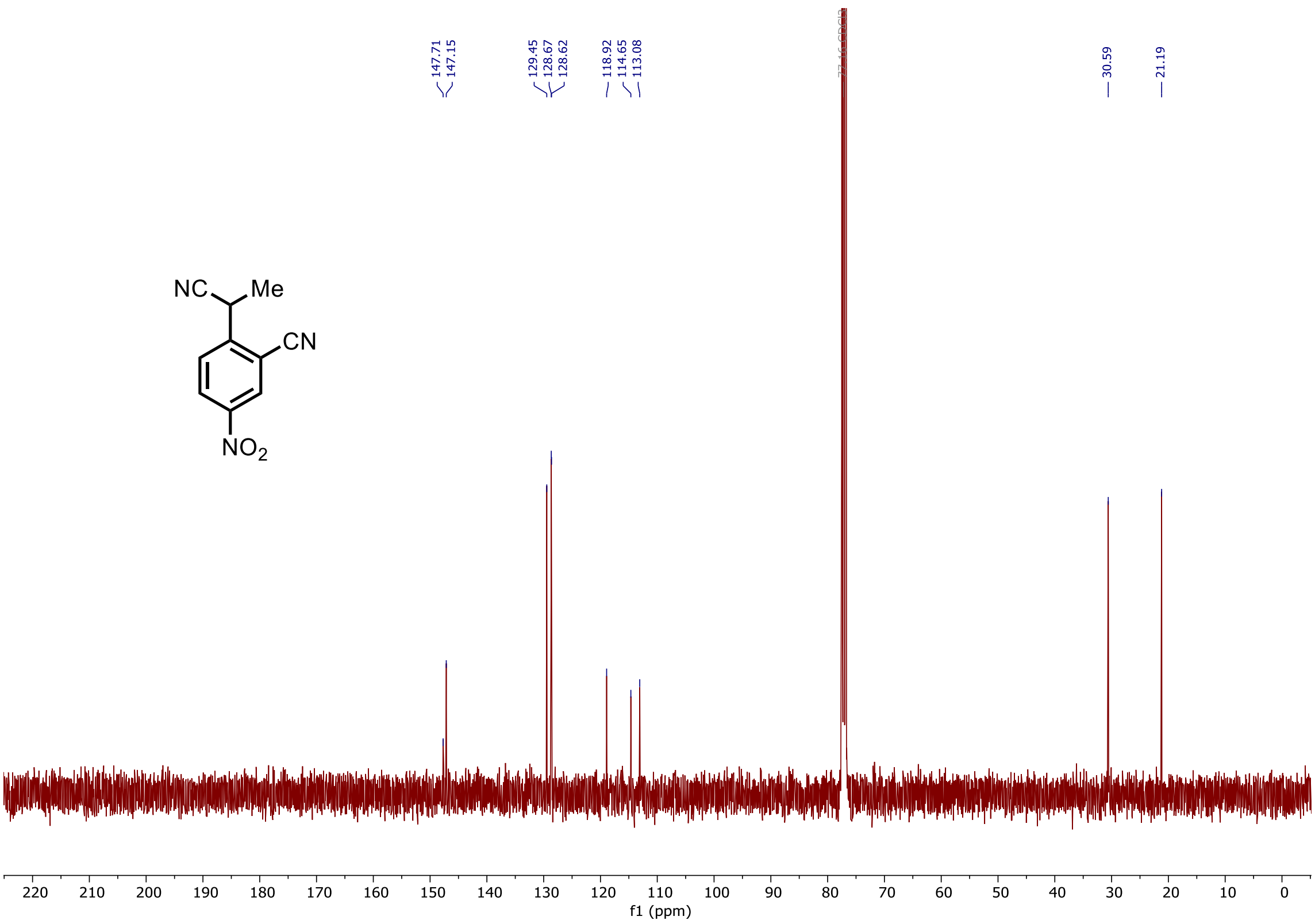

Supplementary Figure 48: $^{13}C\{^1H\}$ NMR (101 MHz, $CDCl_3$) of **14b**

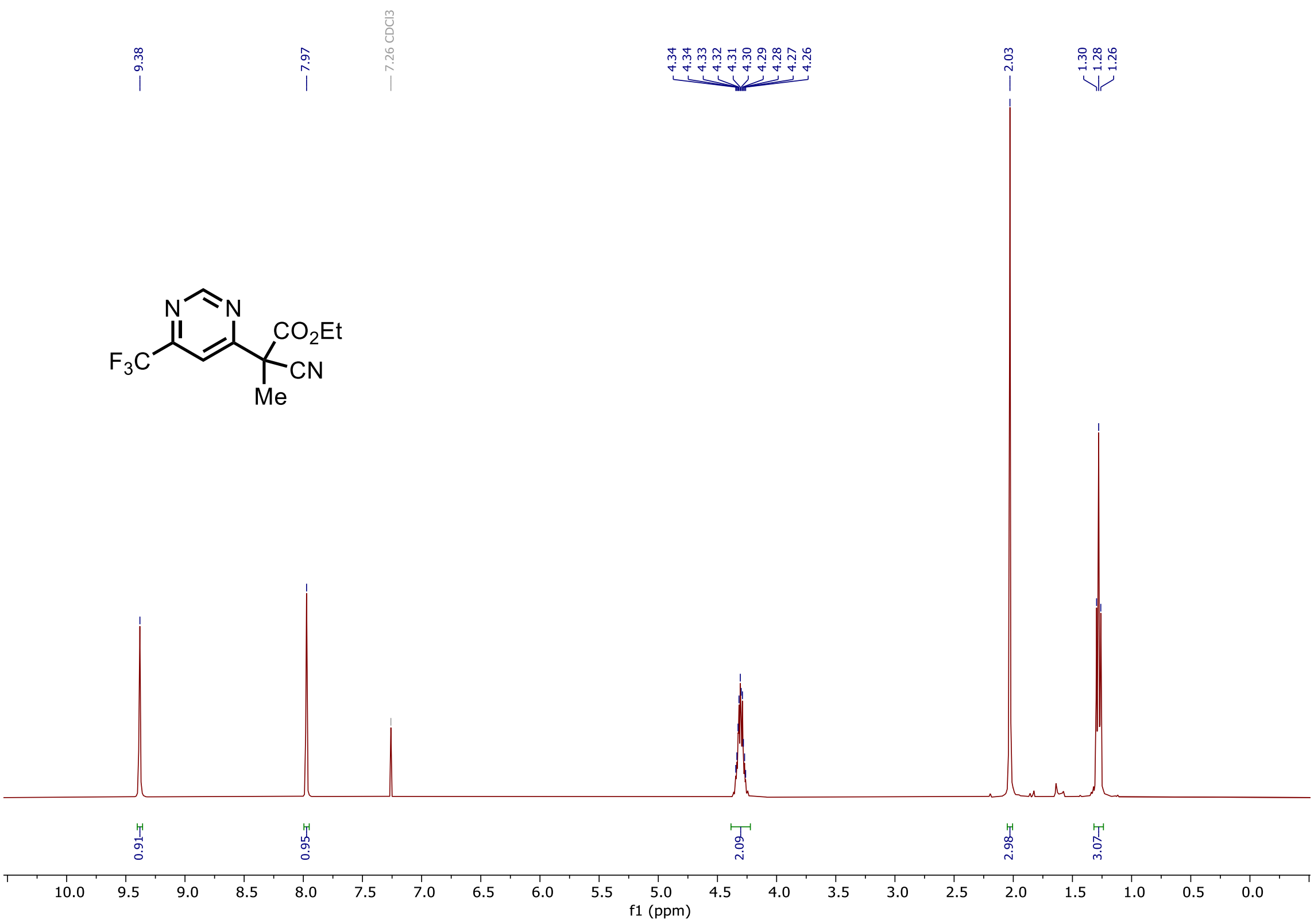

Supplementary Figure 49: ^1H NMR (400 MHz, $CDCl_3$) of **17a**

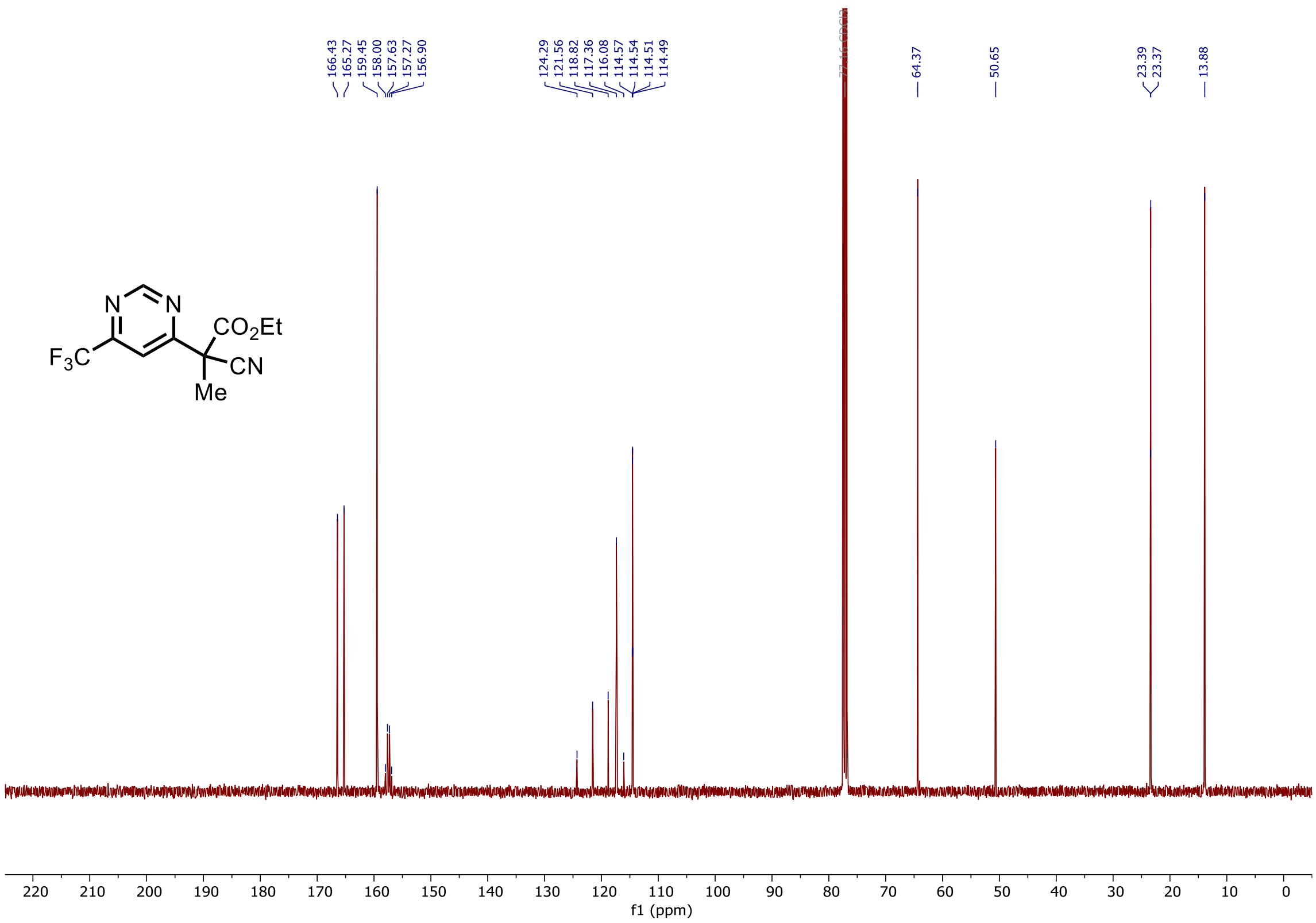

Supplementary Figure 50: $^{13}C\{^{1}H\}$ NMR (101 MHz, $CDCl_3$) of **17a**

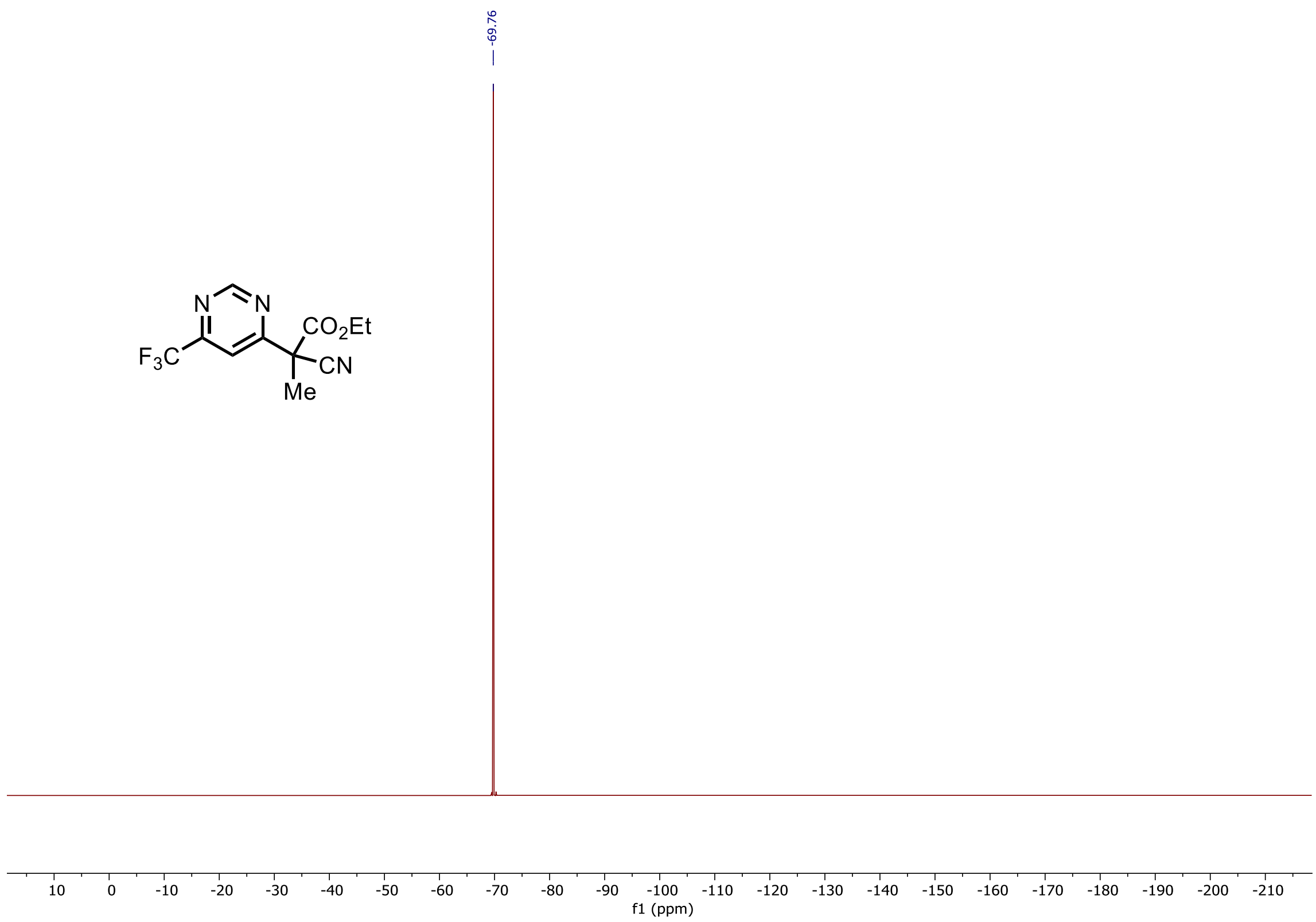

Supplementary Figure 51: $^{19}F\{^{1}H\}$ NMR (376 MHz, $CDCl_3$) of **17a**

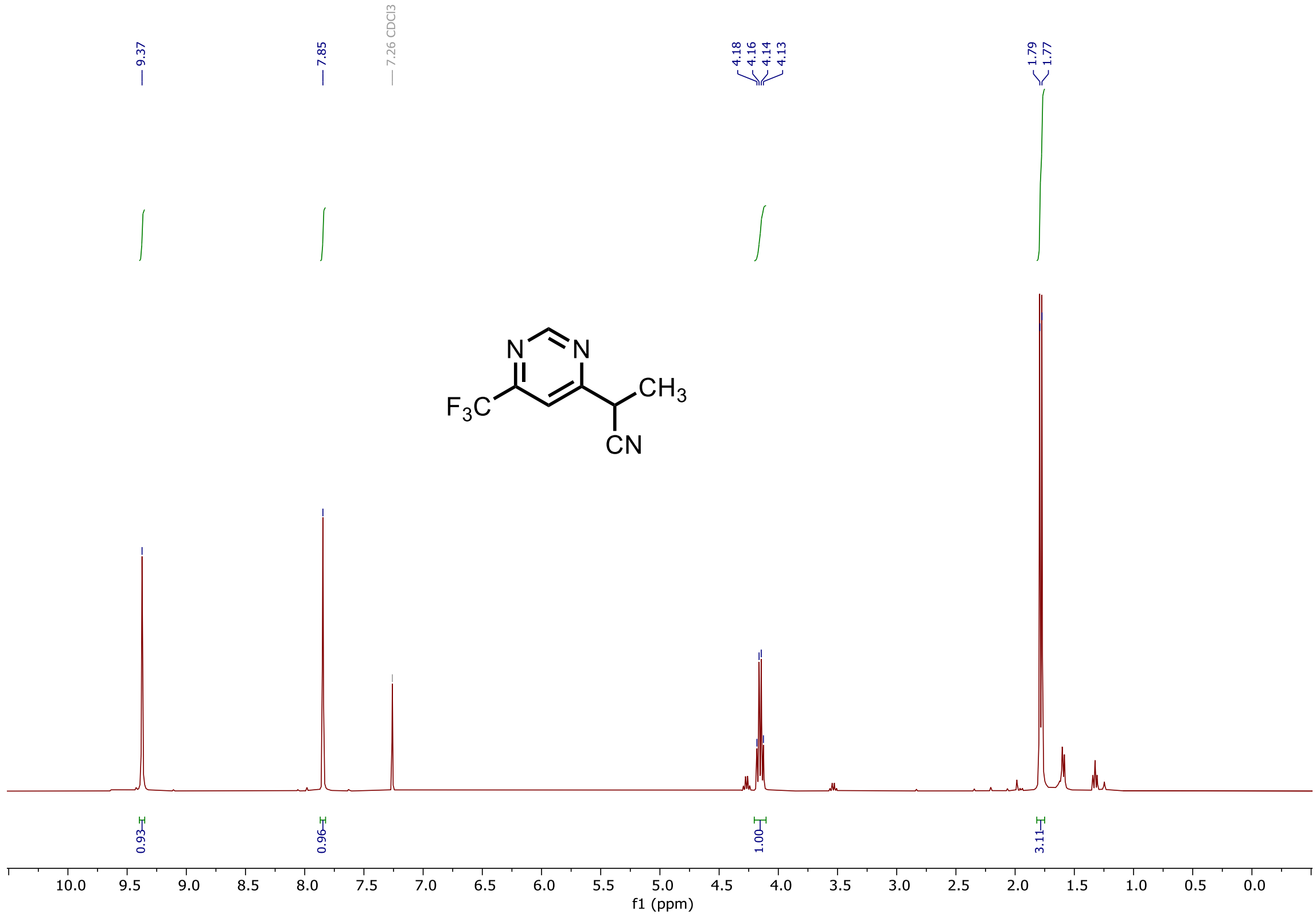

Supplementary Figure 51: ^{1}H NMR (400 MHz, $CDCl_3$) of **17b**

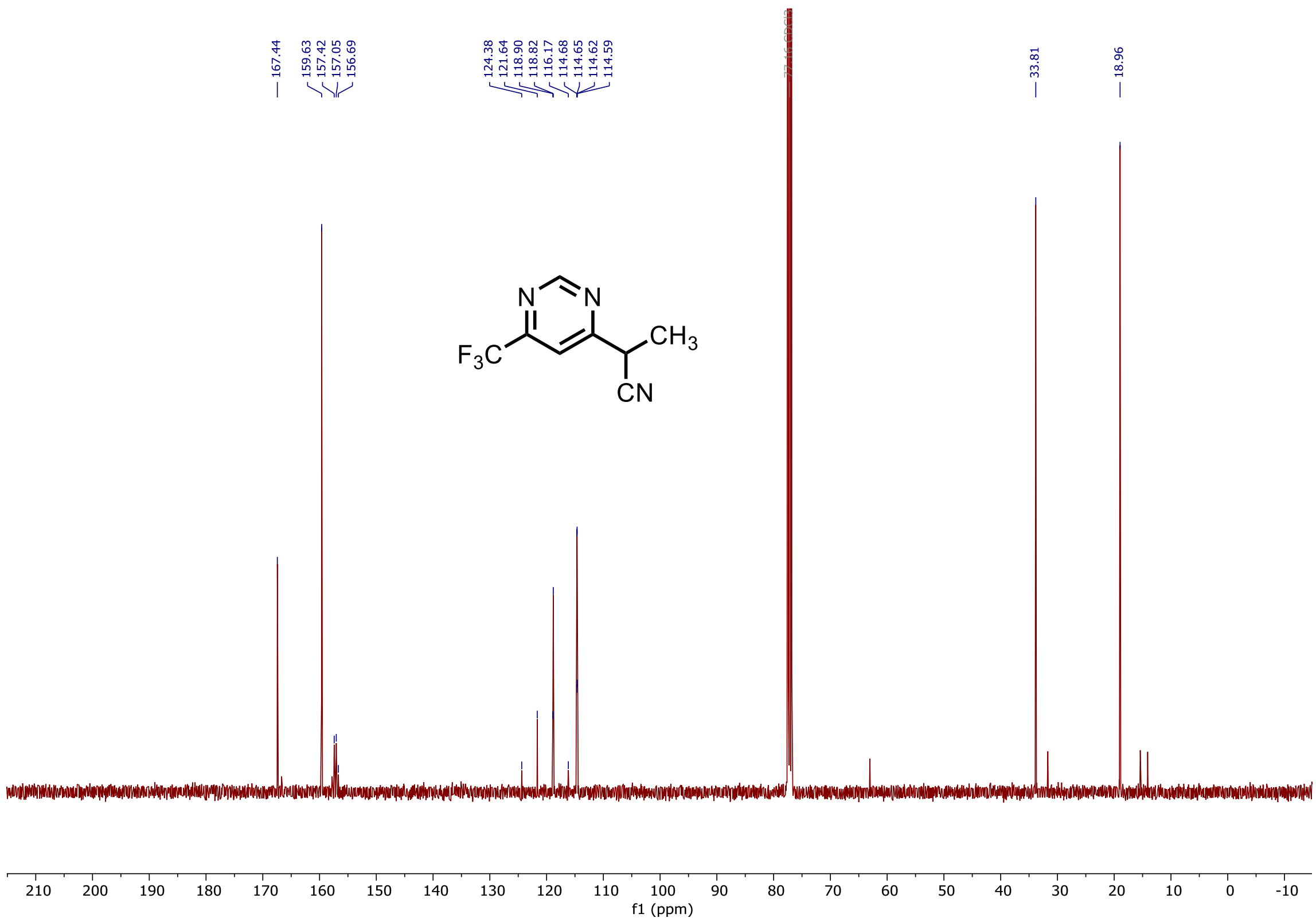

Supplementary Figure 52: $^{13}C\{^1H\}$ NMR (101 MHz, $CDCl_3$) of **17b**

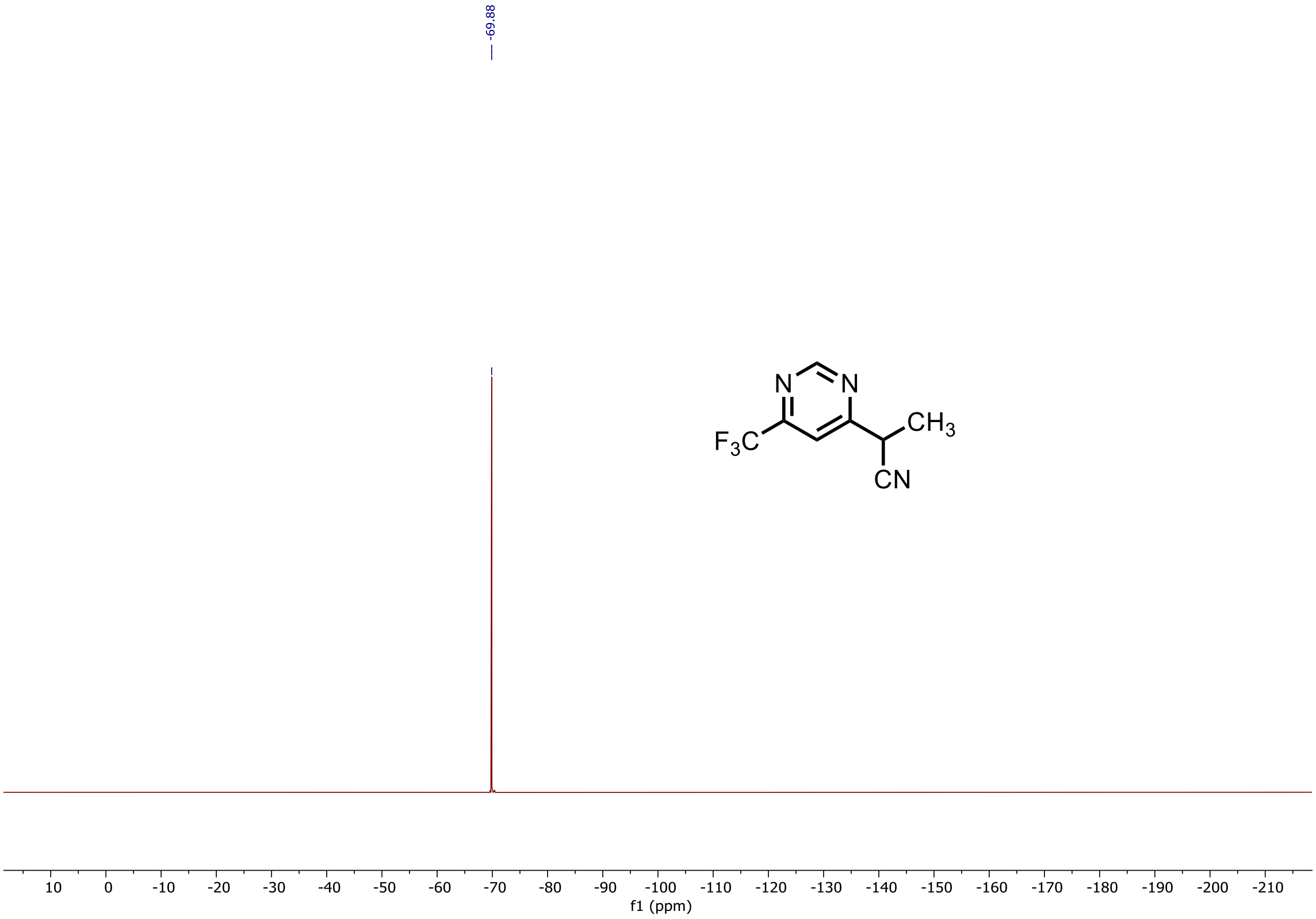

Supplementary Figure 53: $^{19}F\{^{1}H\}$ NMR (376 MHz, $CDCl_3$) of **17b**

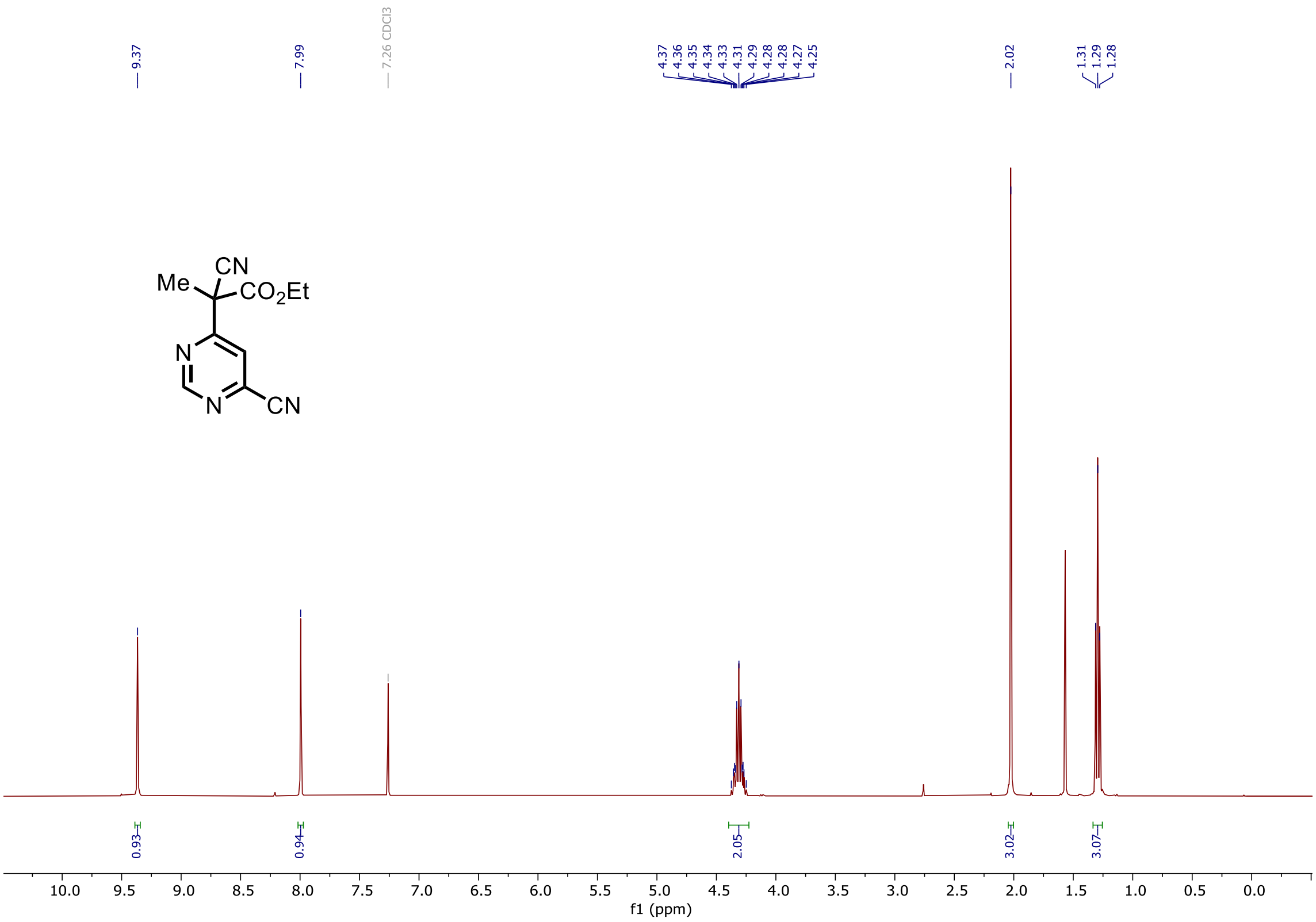

Supplementary Figure 54: ^{1}H NMR (400 MHz, $CDCl_3$) of **18a**

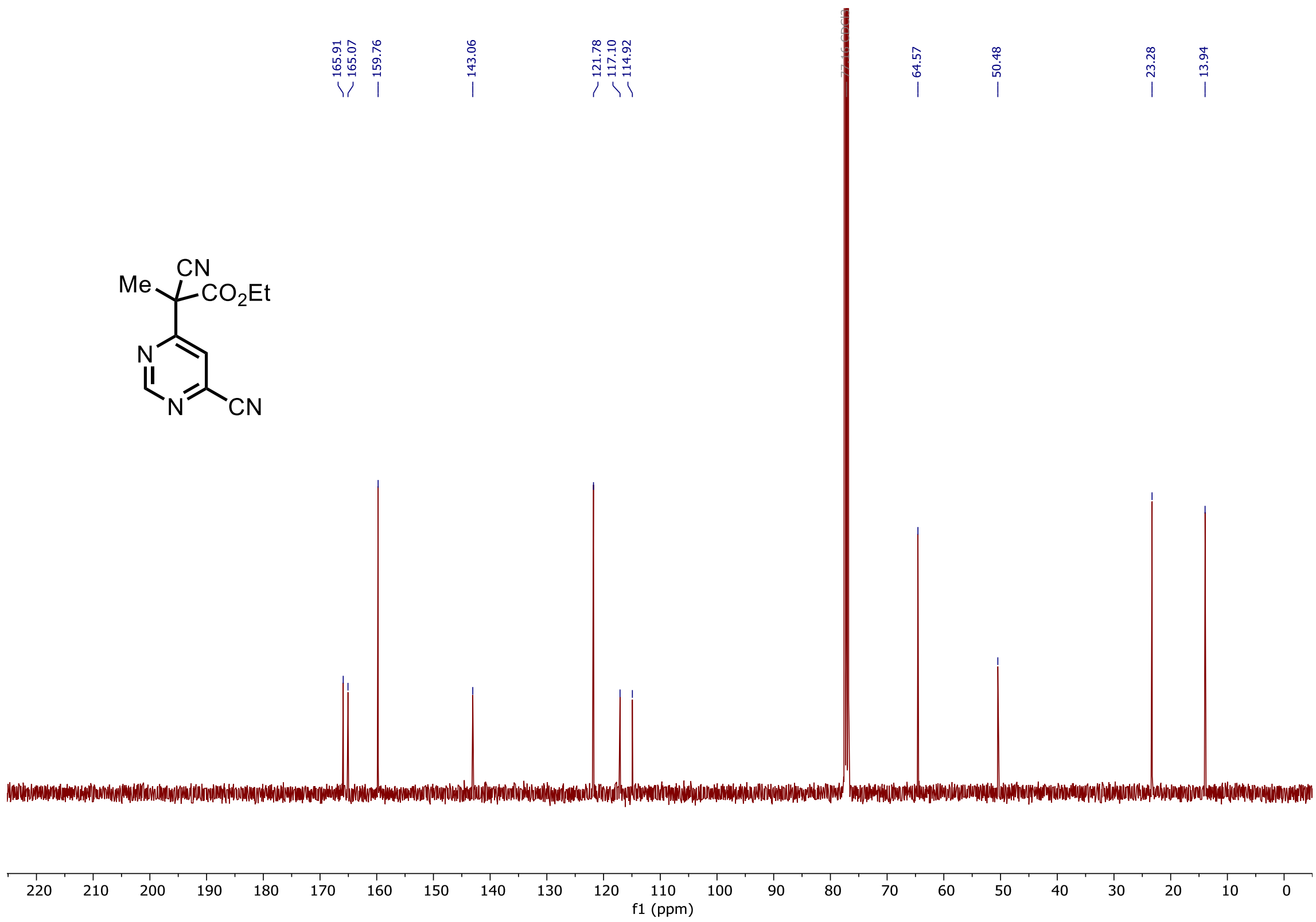

Supplementary Figure 55: $^{13}C\{^{1}H\}$ NMR (101 MHz, $CDCl_3$) of **18a**

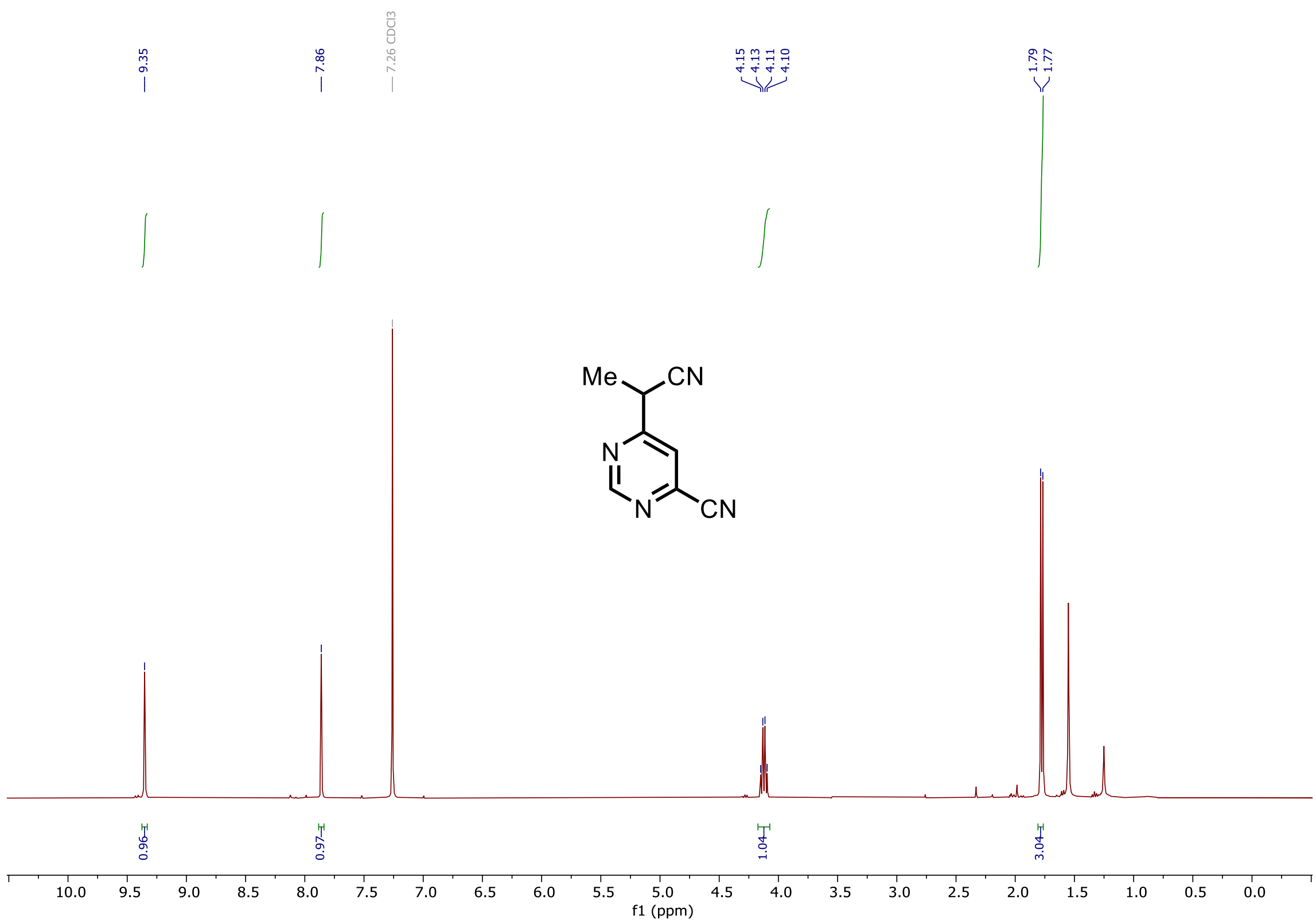

Supplementary Figure 56: ^{1}H NMR (400 MHz, $CDCl_3$) of **18b**

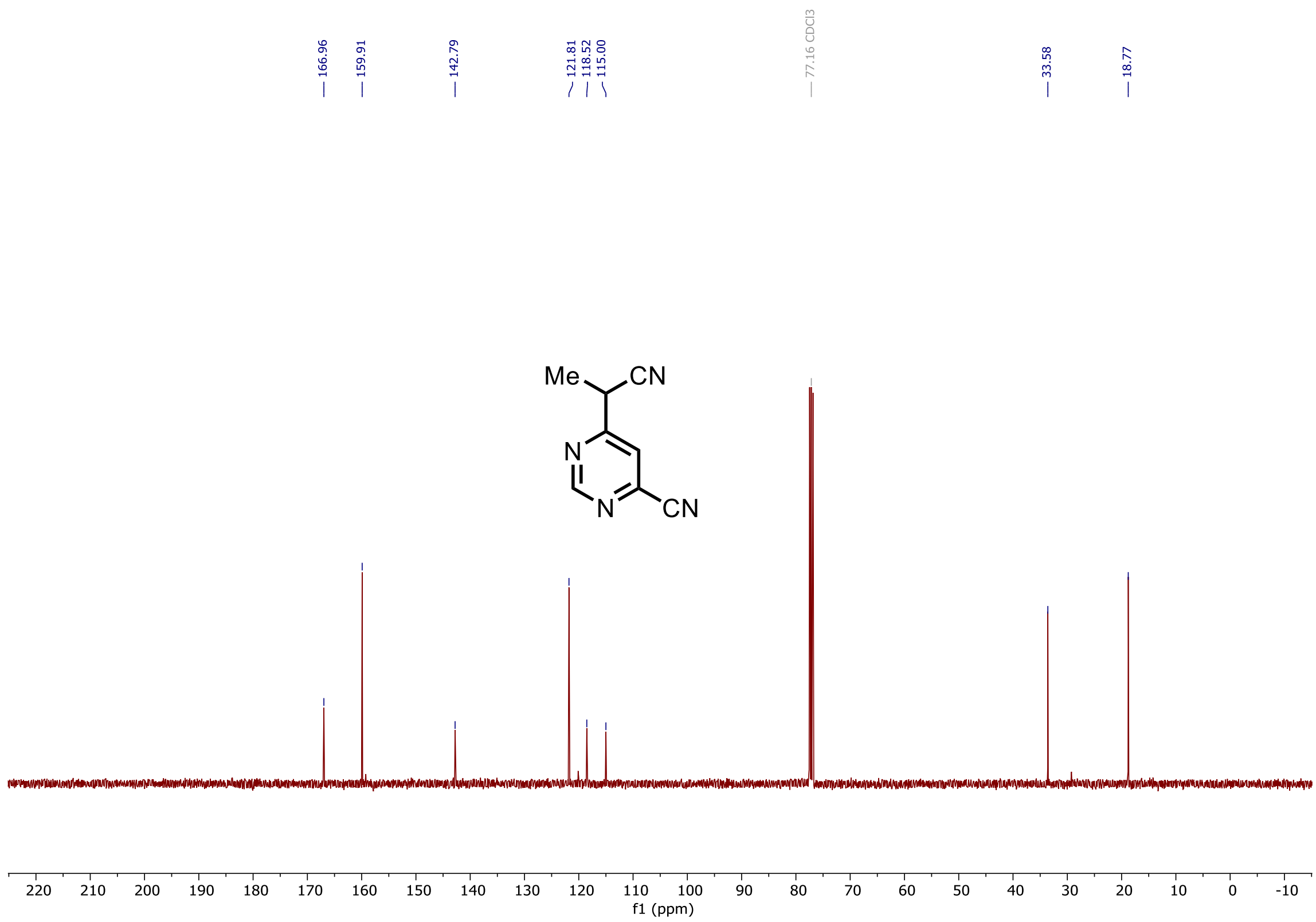

Supplementary Figure 57: $^{13}C\{^{1}H\}$ NMR (101 MHz, $CDCl_3$) of **18b**

Chiral HPLC chromatograms

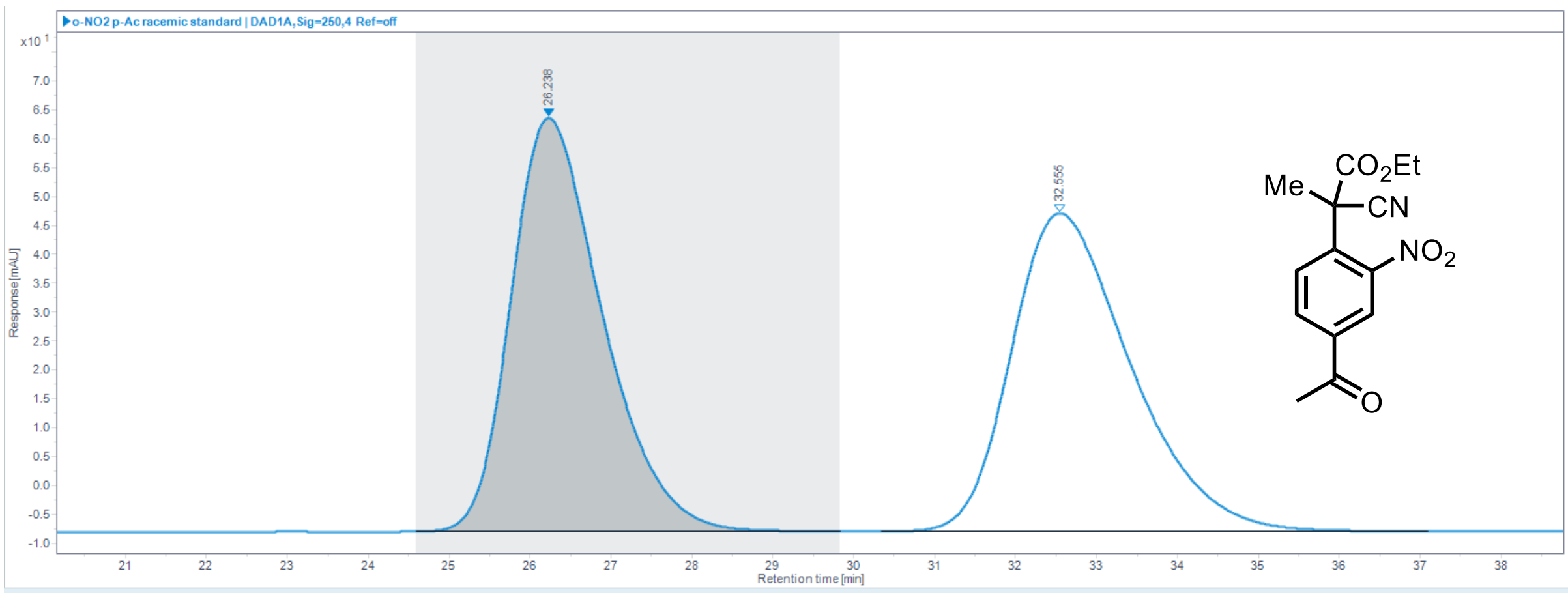

Injection Results

Peaks | Summary

#	Name	Signal description	RT (min)	Area (mAU·s)	Area%	Height (mAU)	Height%	Amount	Concentration	Start time (min)	End time (min)
1		DAD1A,Sig=250,4 Ref=off	26.238	5633.164	49.941	71.568	56.52			24.600	29.836
2		DAD1A,Sig=250,4 Ref=off	32.555	5646.572	50.059	55.061	43.48			30.353	37.110

Chiral HPLC chromatogram of rac-**6**

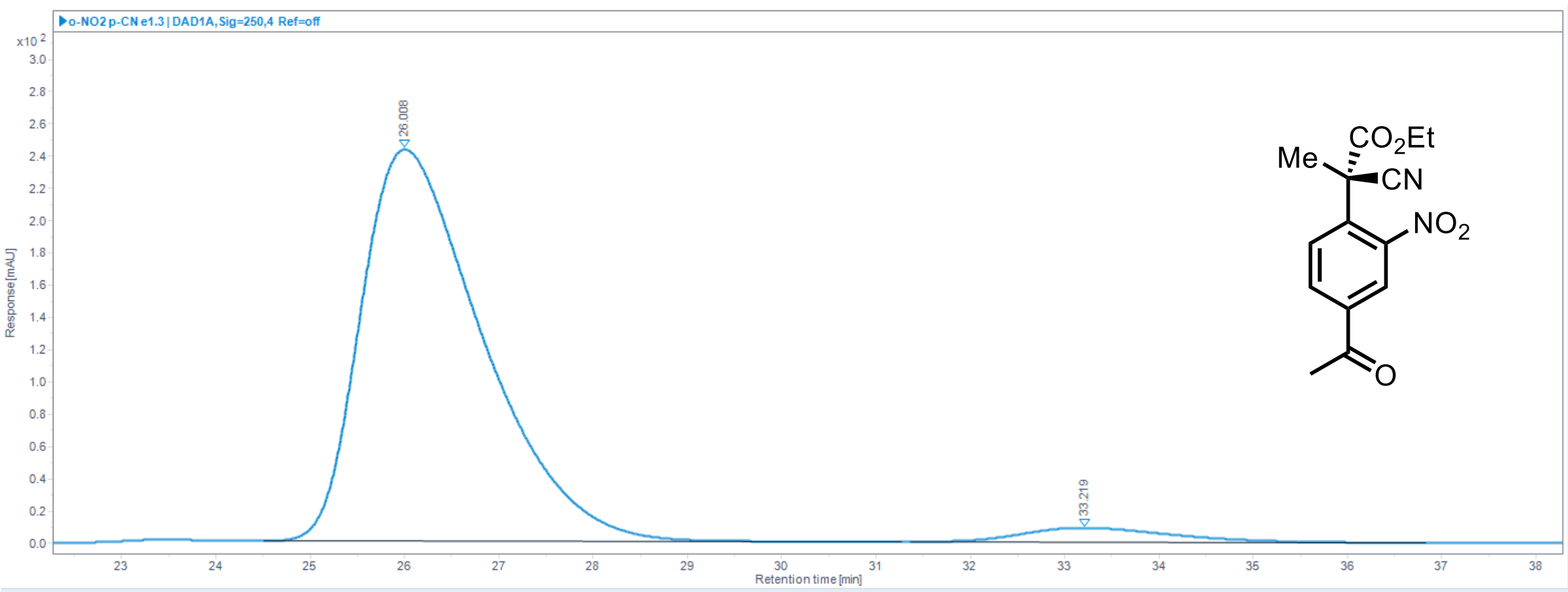

Injection Results

Peaks | Summary

#	Name	Signal description	RT (min)	Area (mAU·s)	Area%	Height (mAU)	Height%	Amount	Concentration	Start time (min)	End time (min)
1		DAD1A,Sig=250,4 Ref=off	26.008	21784.448	95.808	242.737	96.51			24.514	31.280
2		DAD1A,Sig=250,4 Ref=off	33.219	953.210	4.192	8.782	3.49			31.374	36.840

Chiral HPLC chromatogram of **(S)-6** from an analytical scale biotransformation using eS$_N$Ar1.3

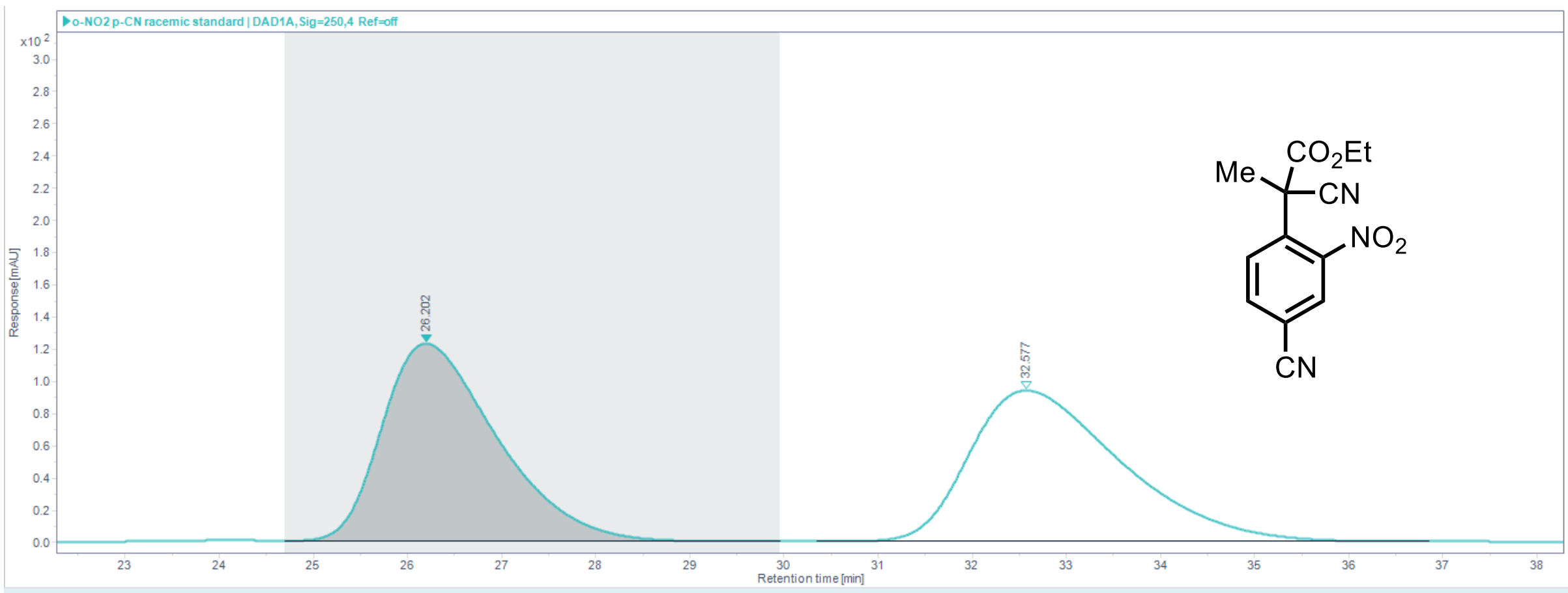

Injection Results

Peaks | Summary

#	Name	Signal description	RT (min)	Area (mAU·s)	Area%	Height (mAU)	Height%	Amount	Concentration	Start time (min)	End time (min)
1		DAD1A,Sig=250,4 Ref=off	26.202	10424.485	49.835	122.608	56.69			24.701	29.968
2		DAD1A,Sig=250,4 Ref=off	32.577	10493.640	50.165	93.687	43.31			30.361	36.858

Chiral HPLC chromatogram of rac-**7**

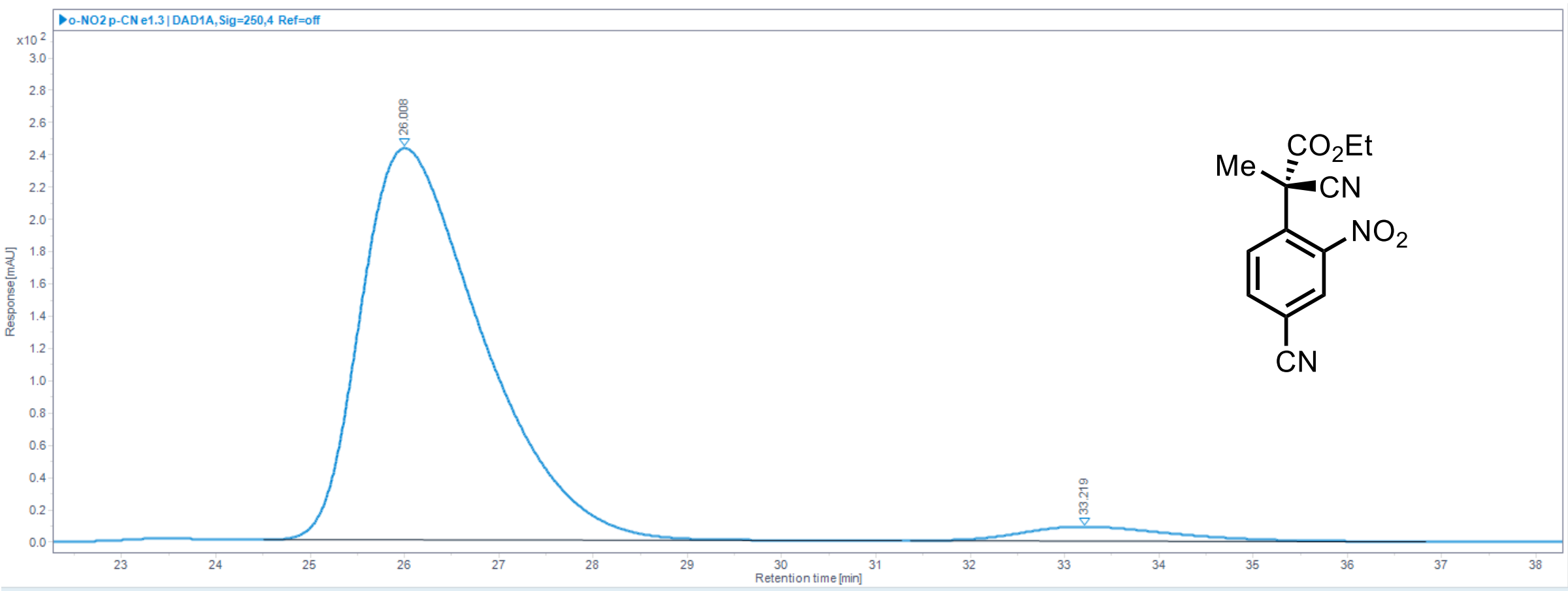

Injection Results

Peaks | Summary

#	Name	Signal description	RT (min)	Area (mAU·s)	Area%	Height (mAU)	Height%	Amount	Concentration	Start time (min)	End time (min)
1		DAD1A,Sig=250,4 Ref=off	26.008	21784.448	95.808	242.737	96.51			24.514	31.280
2		DAD1A,Sig=250,4 Ref=off	33.219	953.210	4.192	8.782	3.49			31.374	36.840

Chiral HPLC chromatogram of **(S)-7** from an analytical scale biotransformation using eS$_N$Ar1.3

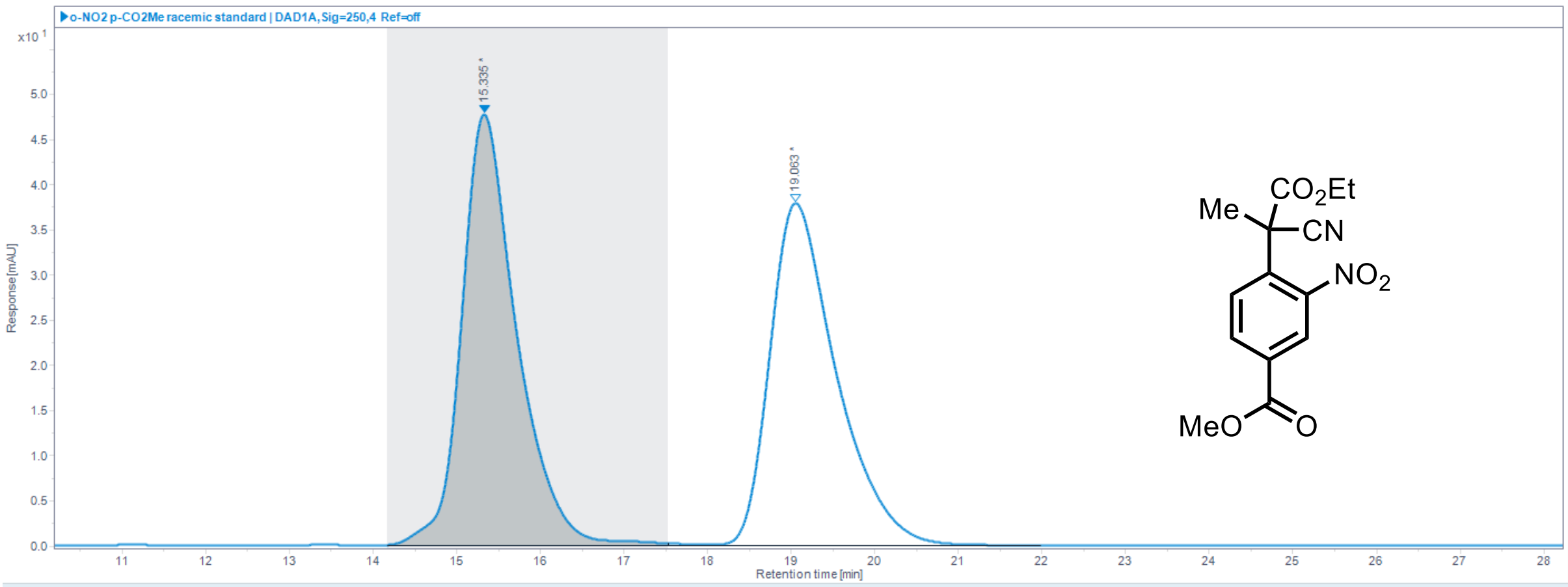

Injection Results

Peaks | Summary

#	Name	Signal description	RT (min)	Area (mAU·s)	Area%	Height (mAU)	Height%	Amount	Concentration	Start time (min)	End time (min)
1		DAD1A,Sig=250,4 Ref=off	15.335	2191.804	51.082	47.675	55.70			14.173	17.537
2		DAD1A,Sig=250,4 Ref=off	19.063	2098.966	48.918	37.917	44.30			17.537	21.983

Chiral HPLC chromatogram of rac-**8**

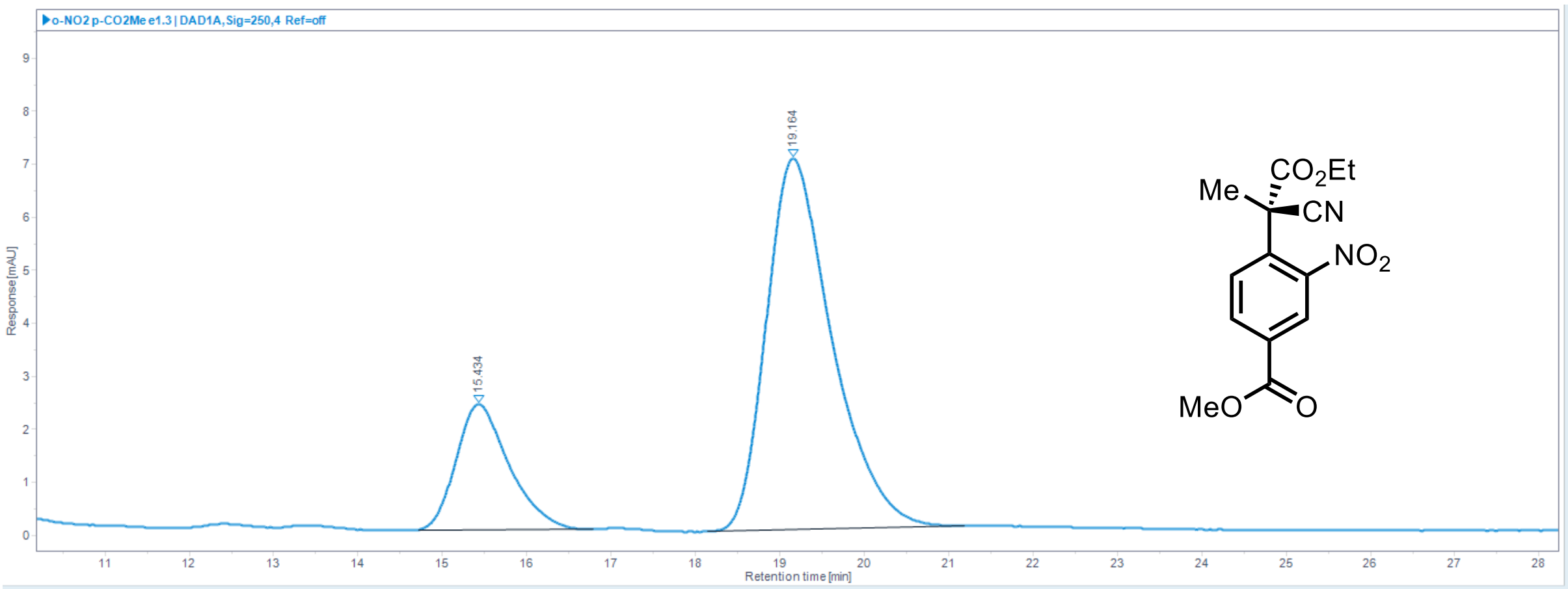

Injection Results

Peaks | Summary

#	Name	Signal description	RT (min)	Area (mAU·s)	Area%	Height (mAU)	Height%	Amount	Concentration	Start time (min)	End time (min)
1		DAD1A,Sig=250,4 Ref=off	15.434	103.189	21.356	2.364	25.23			14.721	16.798
2		DAD1A,Sig=250,4 Ref=off	19.164	379.999	78.644	7.005	74.77			18.148	21.195

Chiral HPLC chromatogram of **(S)-8** from an analytical scale biotransformation using $eS_NAr1.3$

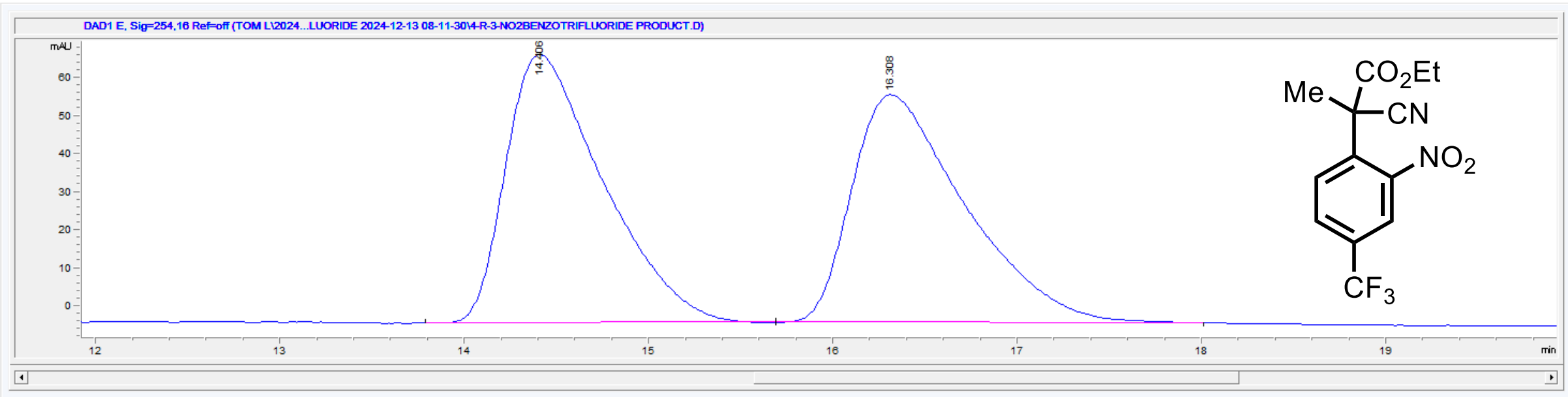

#	Time	Area	Height	Width	Area%	Symmetry
1	14.406	2587.4	70.6	0.5358	50.260	0.502
2	16.308	2560.6	59.9	0.6146	49.740	0.485

Chiral HPLC chromatogram of rac-**9**

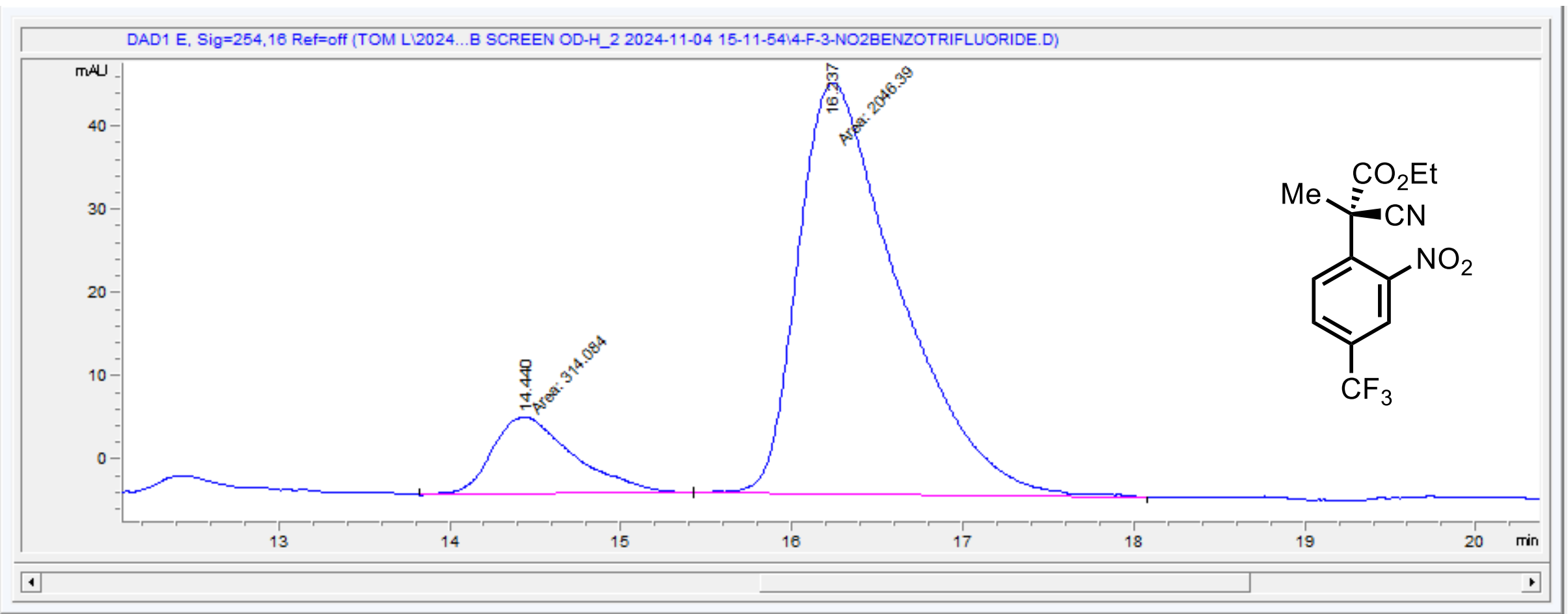

#	Time	Area	Height	Width	Area%	Symmetry
1	14.44	314.1	9.3	0.5648	13.306	0.671
2	16.237	2046.4	49.6	0.6883	86.694	0.512

Chiral HPLC chromatogram of **(S)-9** from an analytical scale biotransformation using eS$_N$Ar1.3

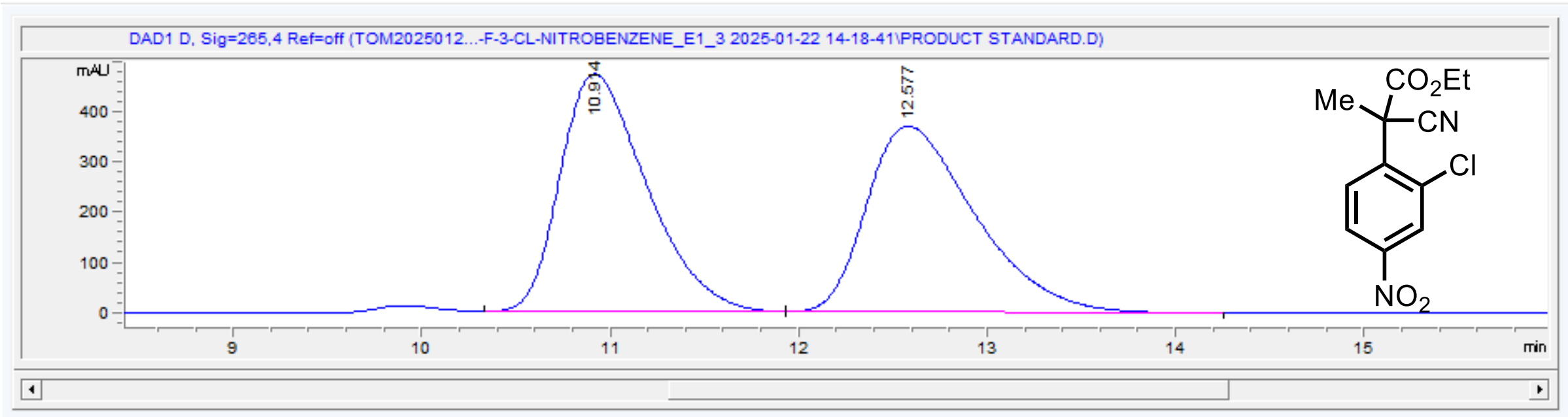

#	Time	Area	Height	Width	Area%	Symmetry
1	10.914	15342.3	476	0.496	50.641	0.625
2	12.577	14954.2	371.9	0.6051	49.359	0.587

Chiral HPLC chromatogram of rac-**10**

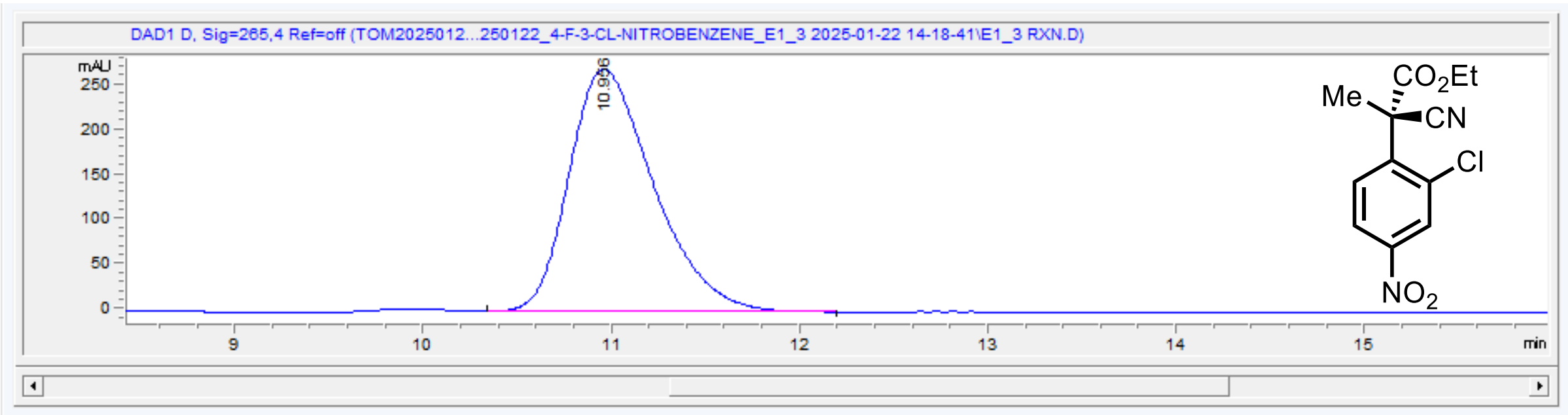

#	Time	Area	Height	Width	Area%	Symmetry
1	10.956	8424.1	269.4	0.4828	100.000	0.656

Chiral HPLC chromatogram of **(S)-10** from an analytical scale biotransformation using eS$_N$Ar1.3

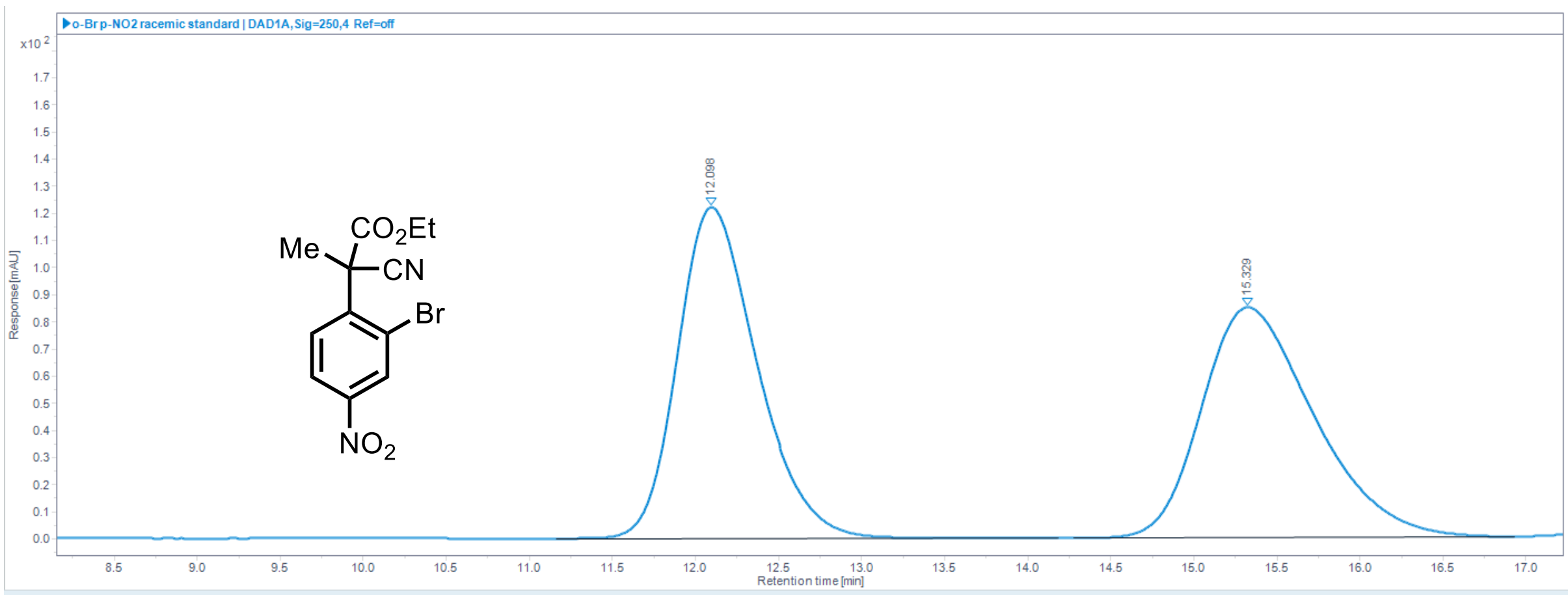

Injection Results

Peaks Summary

#	Name	Signal description	RT (min)	Area (mAU·s)	Area%	Height (mAU)	Height%	Amount	Concentration	Start time (min)	End time (min)
1		DAD1A,Sig=250,4 Ref=off	12.098	4054.775	50.312	121.948	58.97			11.169	14.185
2		DAD1A,Sig=250,4 Ref=off	15.329	4004.481	49.688	84.866	41.03			14.282	16.932

Chiral HPLC chromatogram of rac-**11**

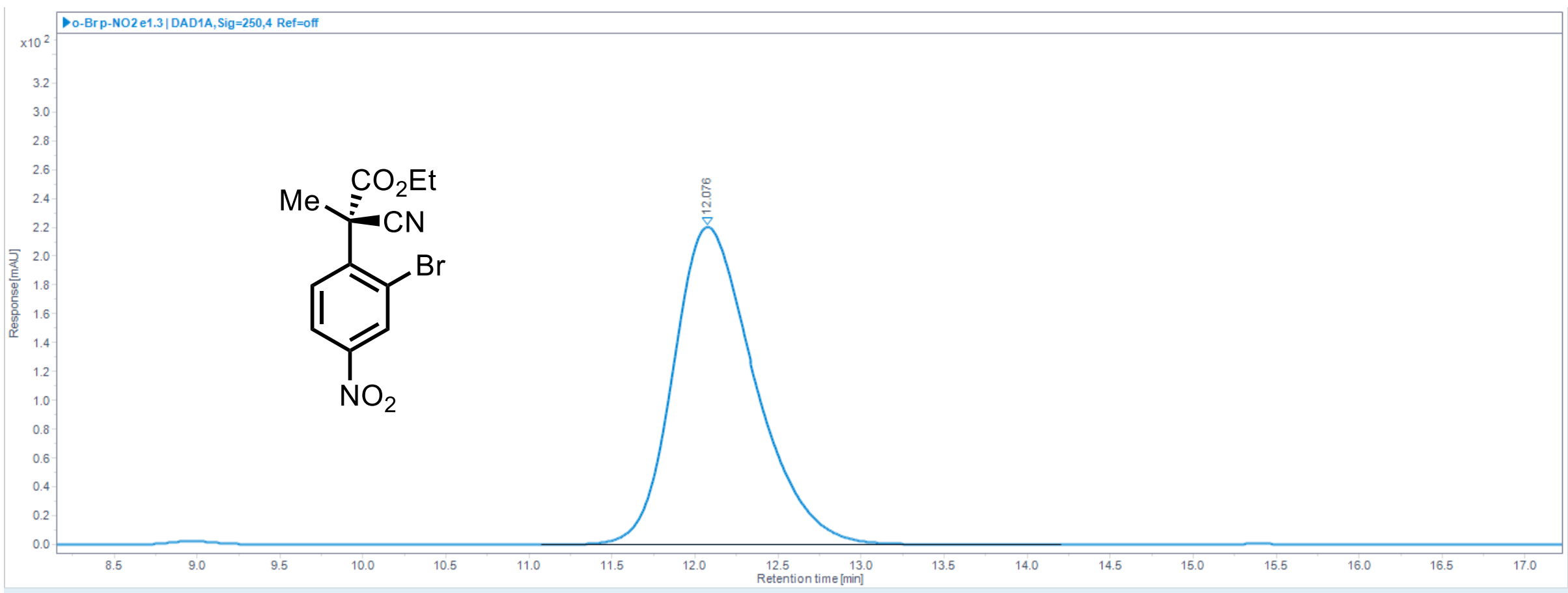

Injection Results

Peaks Summary

#	Name	Signal description	RT (min)	Area (mAU·s)	Area%	Height (mAU)	Height%	Amount	Concentration	Start time (min)	End time (min)
1		DAD1A,Sig=250,4 Ref=off	12.076	7540.893	100.000	220.077	100.00			11.081	14.208

Chiral HPLC chromatogram of **(S)-11** from an analytical scale biotransformation using eS$_N$Ar1.3

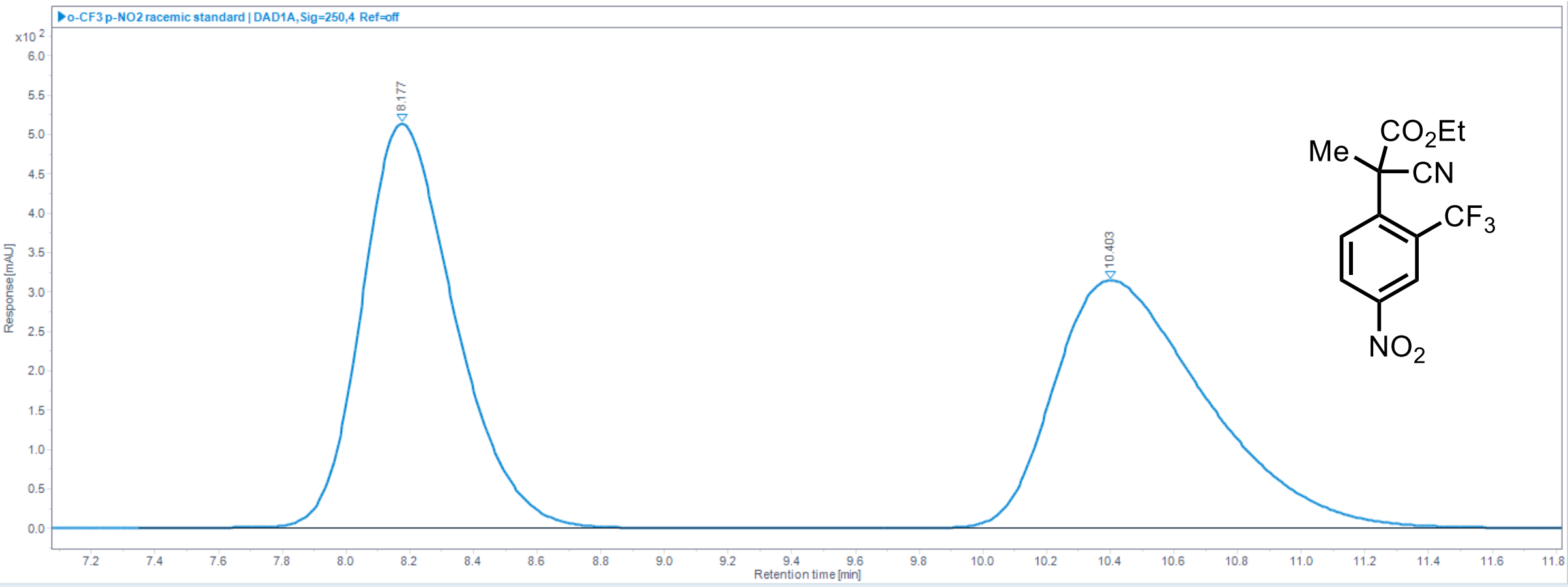

Injection Results

Peaks Summary

#	Name	Signal description	RT (min)	Area (mAU·s)	Area%	Height (mAU)	Height%	Amount	Concentration	Start time (min)	End time (min)
1		DAD1A,Sig=250,4 Ref=off	8.177	10465.279	50.061	513.817	62.00			7.350	9.688
2		DAD1A,Sig=250,4 Ref=off	10.403	10439.787	49.939	314.954	38.00			9.690	12.663

Chiral HPLC chromatogram of rac-**12**

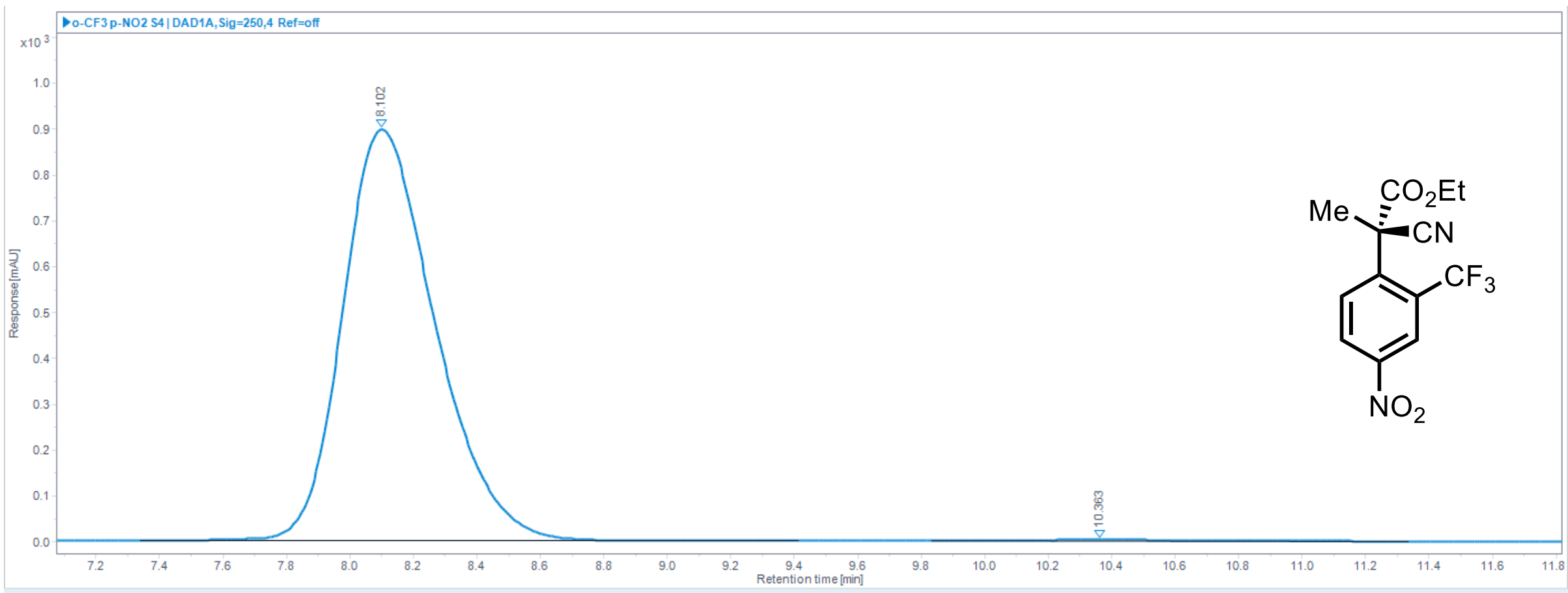

Injection Results

Peaks Summary

#	Name	Signal description	RT (min)	Area (mAU·s)	Area%	Height (mAU)	Height%	Amount	Concentration	Start time (min)	End time (min)
1		DAD1A,Sig=250,4 Ref=off	8.102	18270.557	99.488	898.607	99.69			7.342	9.414
2		DAD1A,Sig=250,4 Ref=off	10.363	93.963	0.512	2.810	0.31			9.835	11.335

Chiral HPLC chromatogram of **(S)-12** from an analytical scale biotransformation using eS$_N$Ar1.2(L13F R65V)

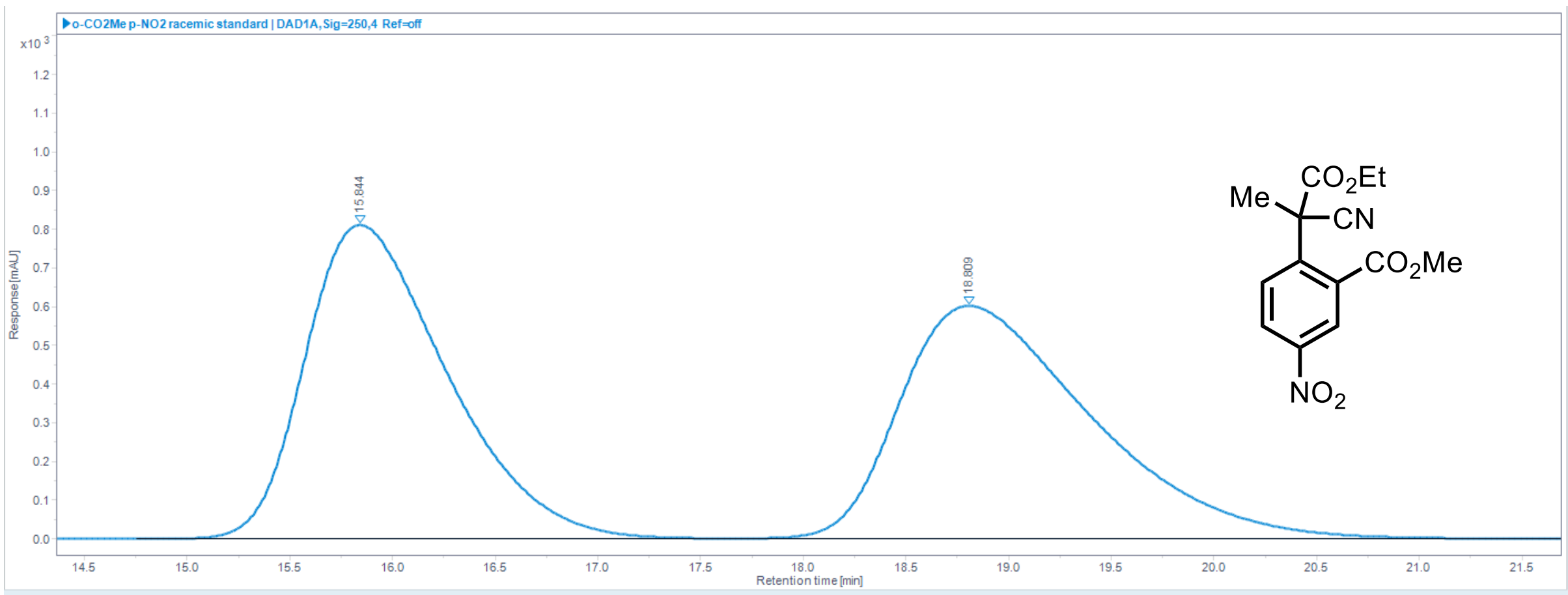

Injection Results

Peaks | Summary

#	Name	Signal description	RT (min)	Area (mAU·s)	Area%	Height (mAU)	Height%	Amount	Concentration	Start time (min)	End time (min)
1		DAD1A,Sig=250,4 Ref=off	15.844	39089.348	49.839	811.511	57.39			14.757	17.677
2		DAD1A,Sig=250,4 Ref=off	18.809	39341.380	50.161	602.434	42.61			17.677	23.810

Chiral HPLC chromatogram of rac-**13**

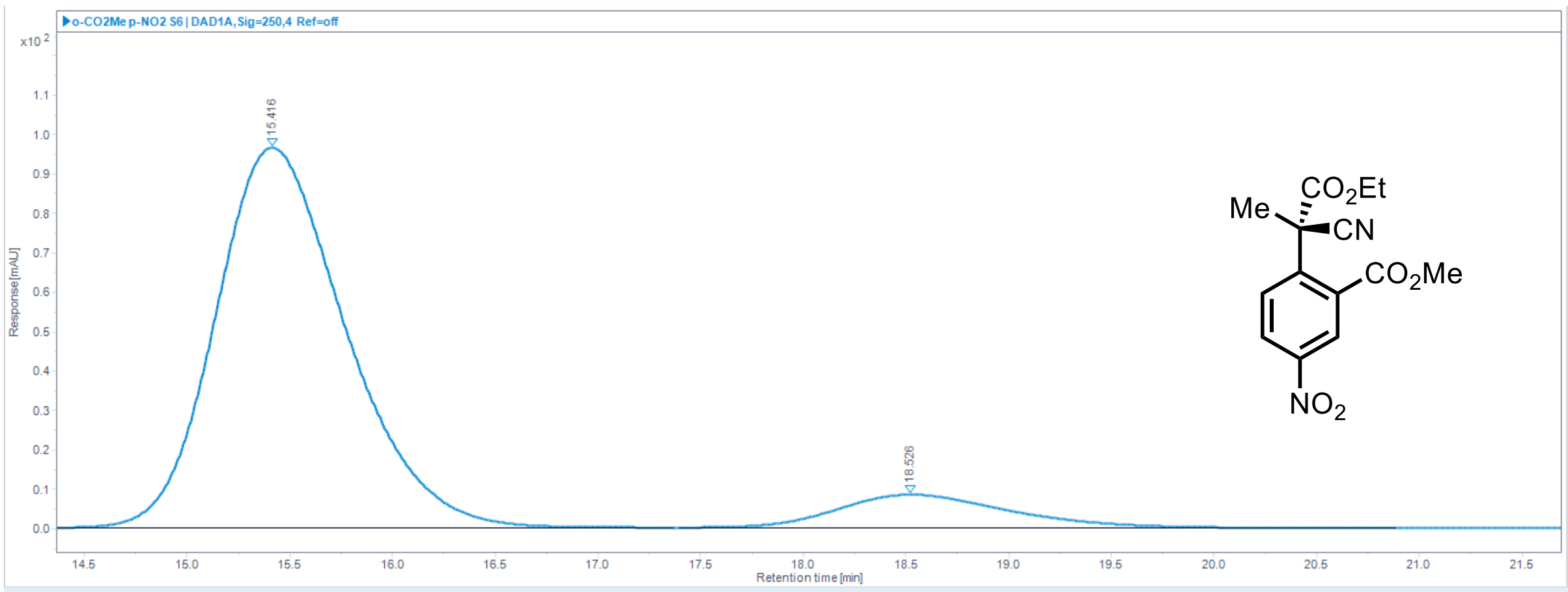

Injection Results

Peaks | Summary

#	Name	Signal description	RT (min)	Area (mAU·s)	Area%	Height (mAU)	Height%	Amount	Concentration	Start time (min)	End time (min)
1		DAD1A,Sig=250,4 Ref=off	15.416	4244.487	89.771	96.430	92.02			14.153	17.369
2		DAD1A,Sig=250,4 Ref=off	18.526	483.625	10.229	8.359	7.98			17.399	20.886

Chiral HPLC chromatogram of **(S)-13** from an analytical scale biotransformation using eS$_N$Ar1.2(R65V D66E)

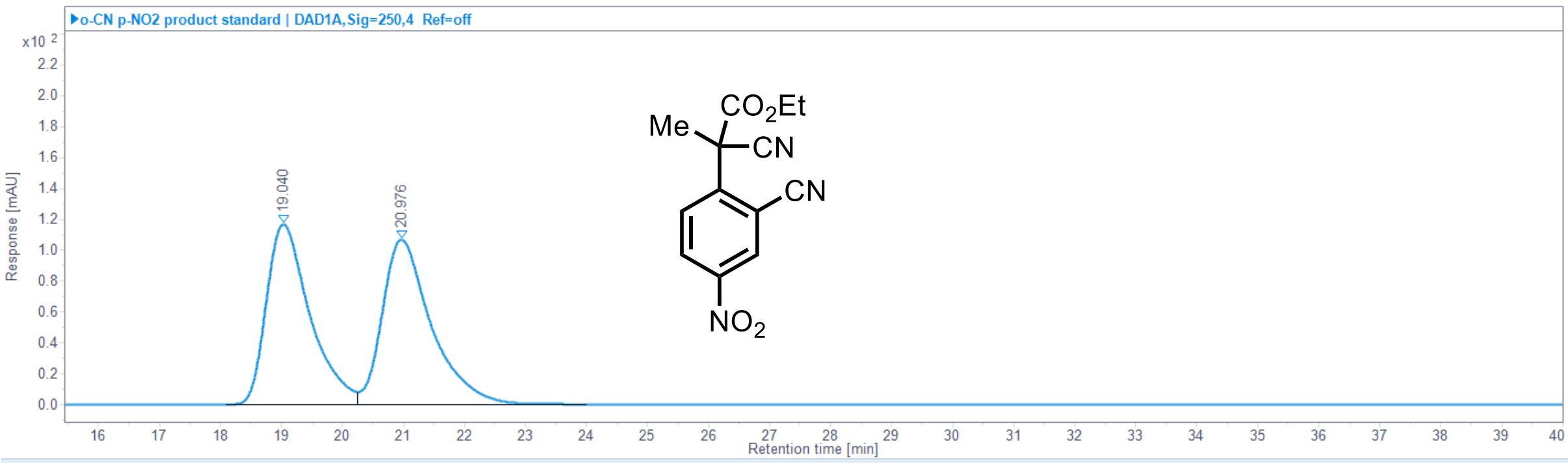

Injection Results

Peaks Summary

#	Name	Signal description	RT (min)	Area (mAU...	Area%	Height (m...	Height%	Amount	Concentration	Start time (min)	End time (min)
1		DAD1A,Sig=250,4 Ref=off	19.040	5758.627	49.228	116.577	52.23			18.098	20.260
2		DAD1A,Sig=250,4 Ref=off	20.976	5939.126	50.772	106.616	47.77			20.260	24.005

Chiral HPLC chromatogram of rac-**14a**

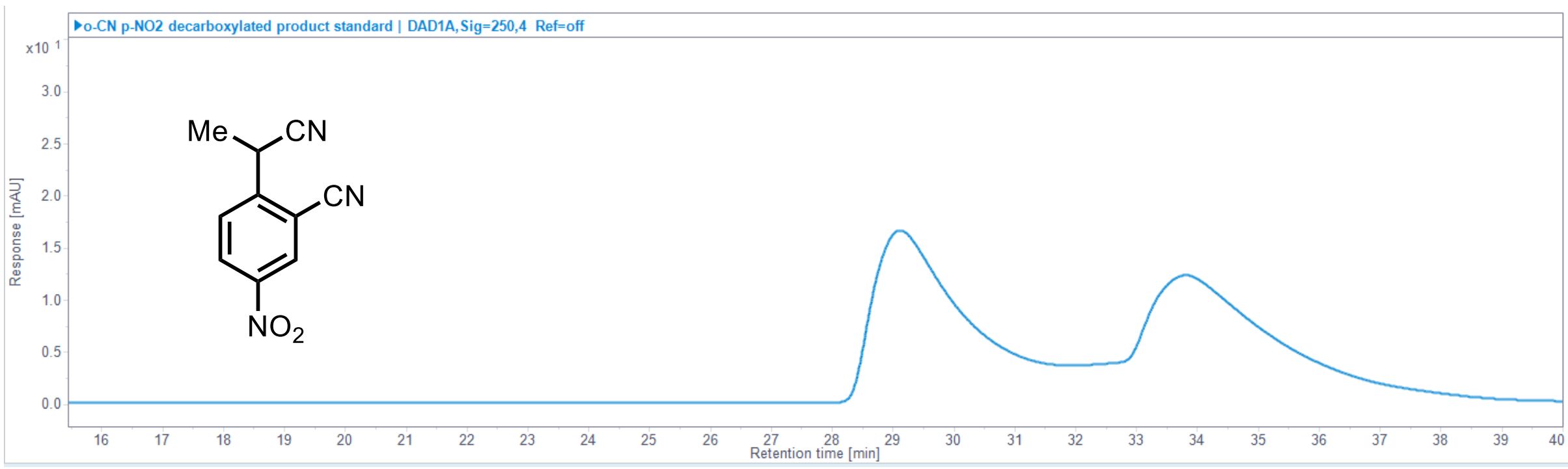

Chiral HPLC chromatogram of rac-**14b**

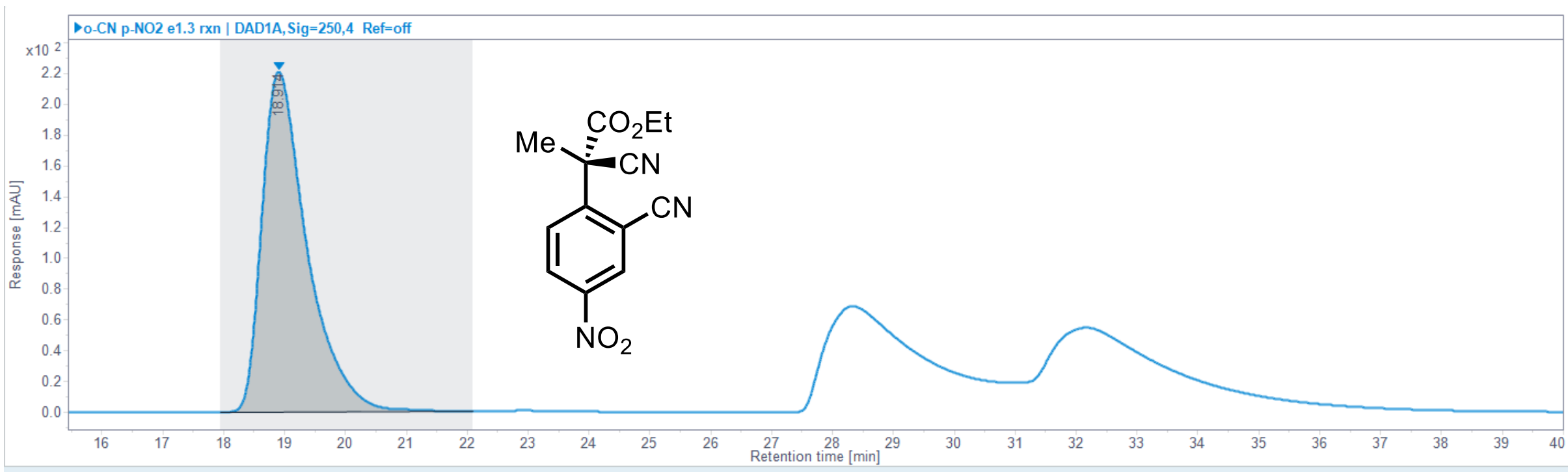

Injection Results

Peaks Summary

#	Name	Signal description	RT (min)	Area (mAU...	Area%	Height (m...	Height%	Amount	Concentration	Start time (min)	End time (min)
1		DAD1A,Sig=250,4 Ref=off	18.914	11378.318	100.000	220.580	100.00			17.955	22.109

Chiral HPLC chromatogram of **(S)-11** from an analytical scale biotransformation using eSNAr1.3

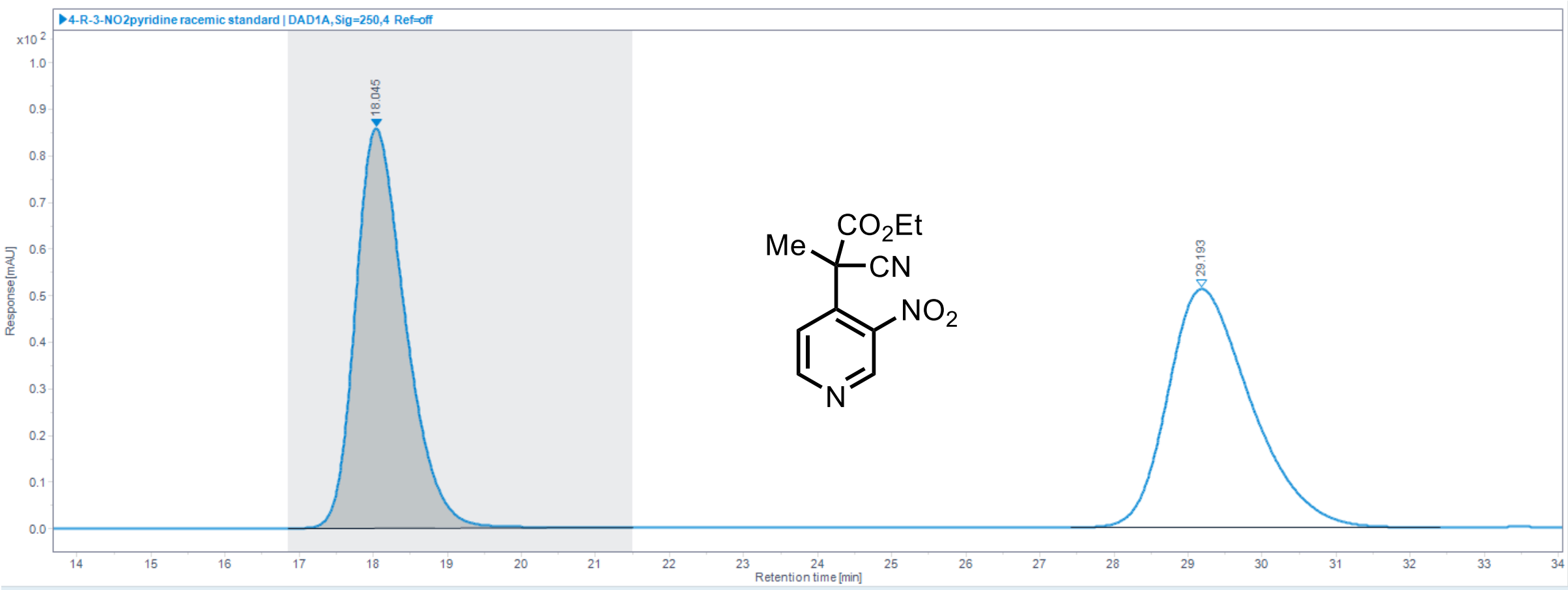

Injection Results

Peaks | Summary

#	Name	Signal description	RT (min)	Area (mAU·s)	Area%	Height (mAU)	Height%	Amount	Concentration	Start time (min)	End time (min)
1		DAD1A,Sig=250,4 Ref=off	18.045	4014.888	50.085	85.732	62.63			16.869	21.512
2		DAD1A,Sig=250,4 Ref=off	29.193	4001.249	49.915	51.155	37.37			27.422	32.405

Chiral HPLC chromatogram of rac-**15**

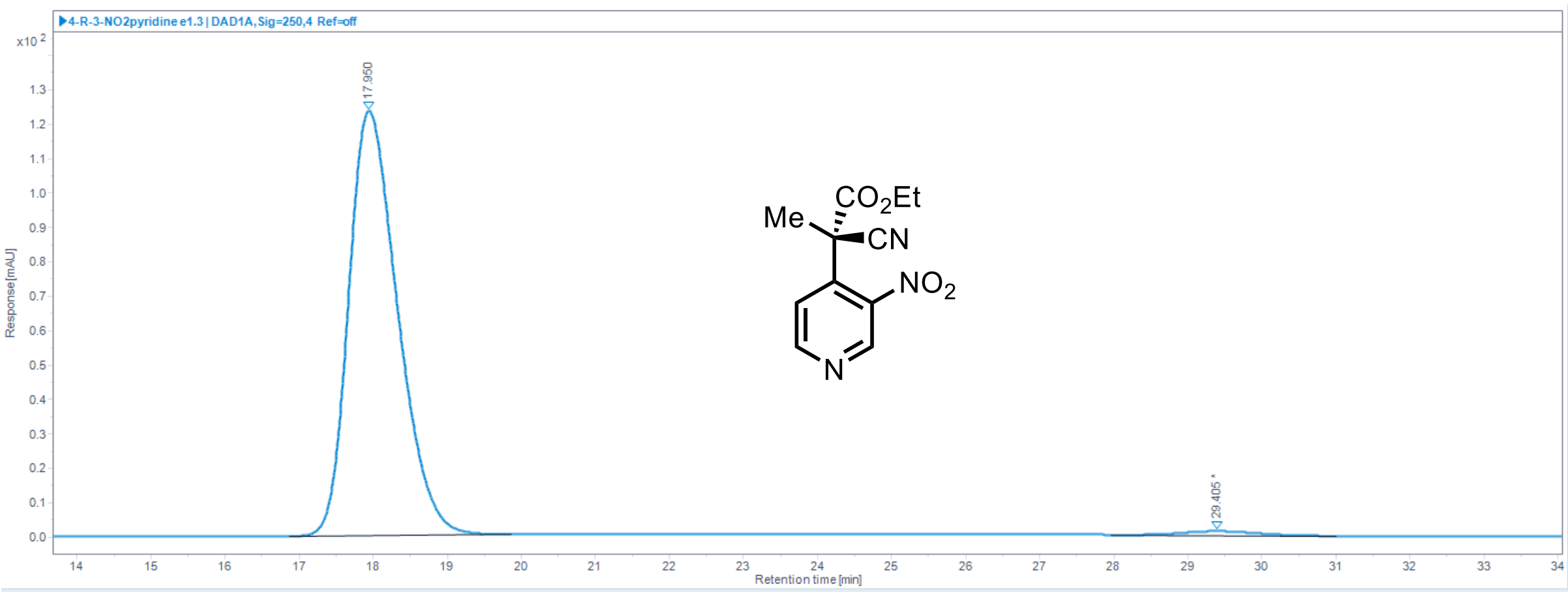

Injection Results

Peaks | Summary

#	Name	Signal description	RT (min)	Area (mAU·s)	Area%	Height (mAU)	Height%	Amount	Concentration	Start time (min)	End time (min)
1		DAD1A,Sig=250,4 Ref=off	17.950	5661.084	98.543	123.433	99.04			16.875	19.868
2		DAD1A,Sig=250,4 Ref=off	29.405	83.710	1.457	1.202	0.96			27.974	31.016

Chiral HPLC chromatogram of **(S)-15** from an analytical scale biotransformation using eS$_N$Ar1.3

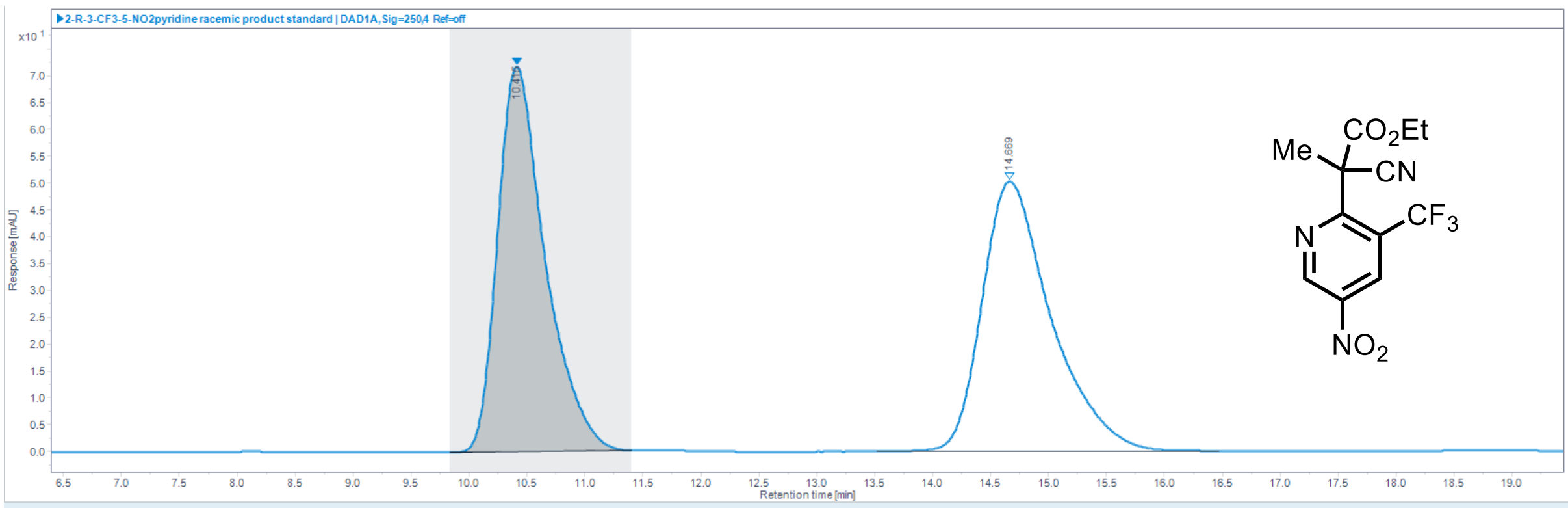

Injection Results

Peaks | Summary

#	Name	Signal description	RT (min)	Area (mAU·s)	Area%	Height (mAU)	Height%	Amount	Concentration	Start time (min)	End time (min)
1		DAD1A,Sig=250,4 Ref=off	10.415	2048.792	49.601	71.797	58.80			9.840	11.406
2		DAD1A,Sig=250,4 Ref=off	14.669	2081.744	50.399	50.303	41.20			13.520	16.473

Chiral HPLC chromatogram of rac-**16**

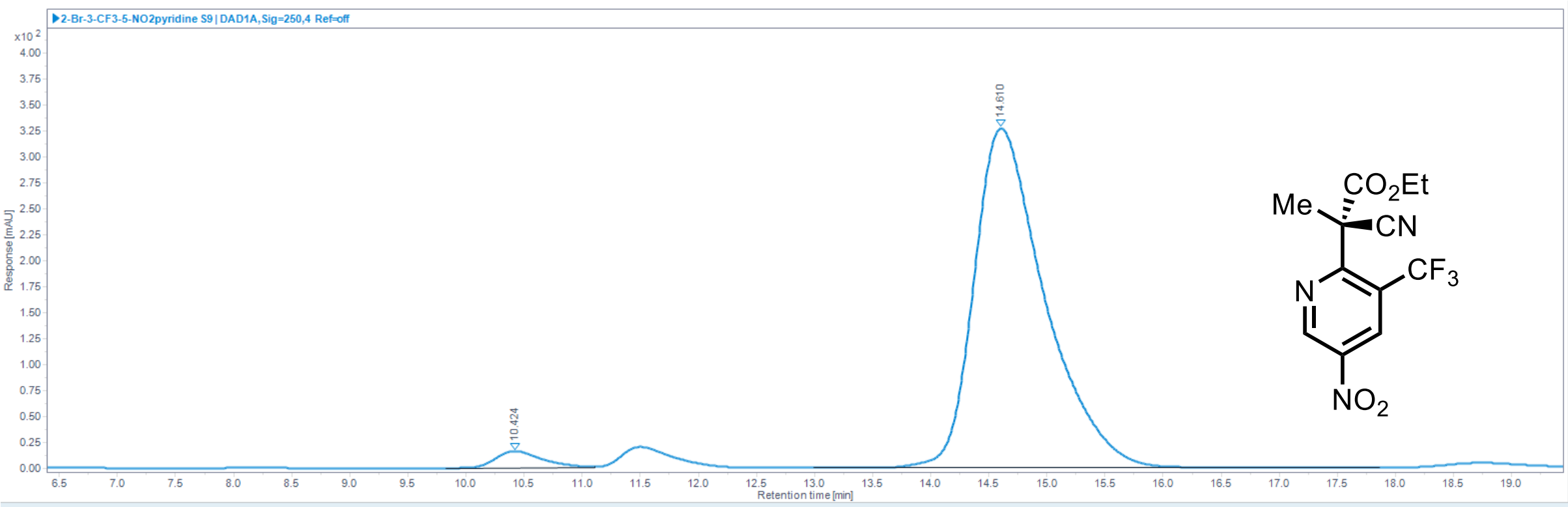

Injection Results

Peaks | Summary

#	Name	Signal description	RT (min)	Area (mAU·s)	Area%	Height (mAU)	Height%	Amount	Concentration	Start time (min)	End time (min)
1		DAD1A,Sig=250,4 Ref=off	10.424	462.789	3.227	16.223	4.73			9.835	11.110
2		DAD1A,Sig=250,4 Ref=off	14.610	13876.385	96.773	326.583	95.27			13.002	17.865

Chiral HPLC chromatogram of **(S)-16** from an analytical scale biotransformation using eS$_N$Ar1.2(L13F R65V D66E)

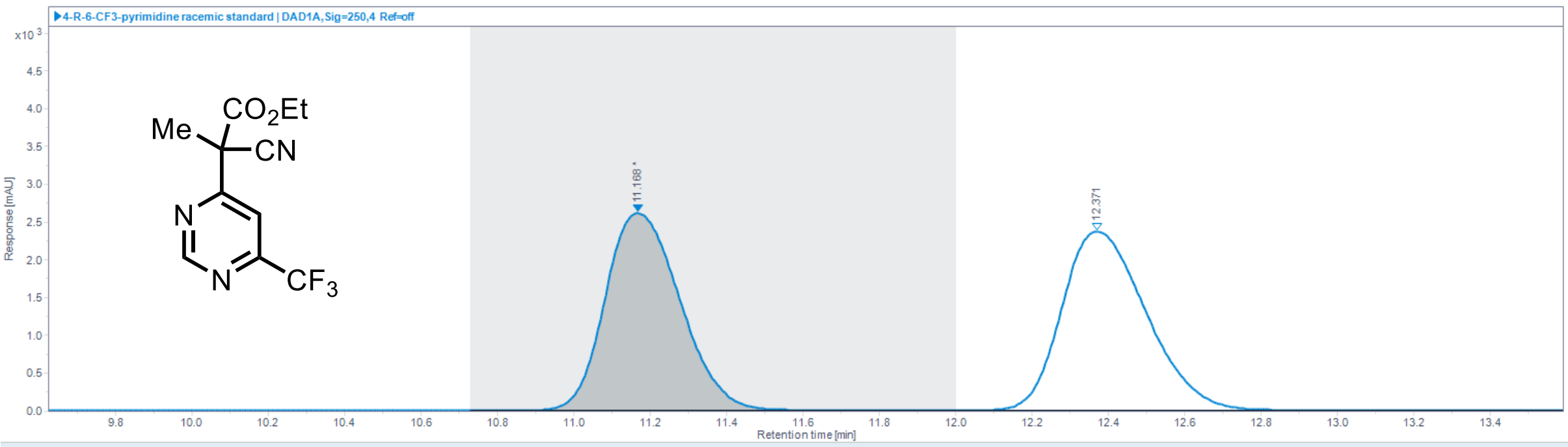

Injection Results

Peaks | Summary

#	Name	Signal description	RT (min)	Area (mAU·s)	Area%	Height (mAU)	Height%	Amount	Concentration	Start time (min)	End time (min)
1		DAD1A,Sig=250,4 Ref=off	11.168	35550.490	49.431	2610.969	52.42			10.731	12.003
2		DAD1A,Sig=250,4 Ref=off	12.371	36369.449	50.569	2369.812	47.58			12.003	13.930

Chiral HPLC chromatogram of rac-**17a**

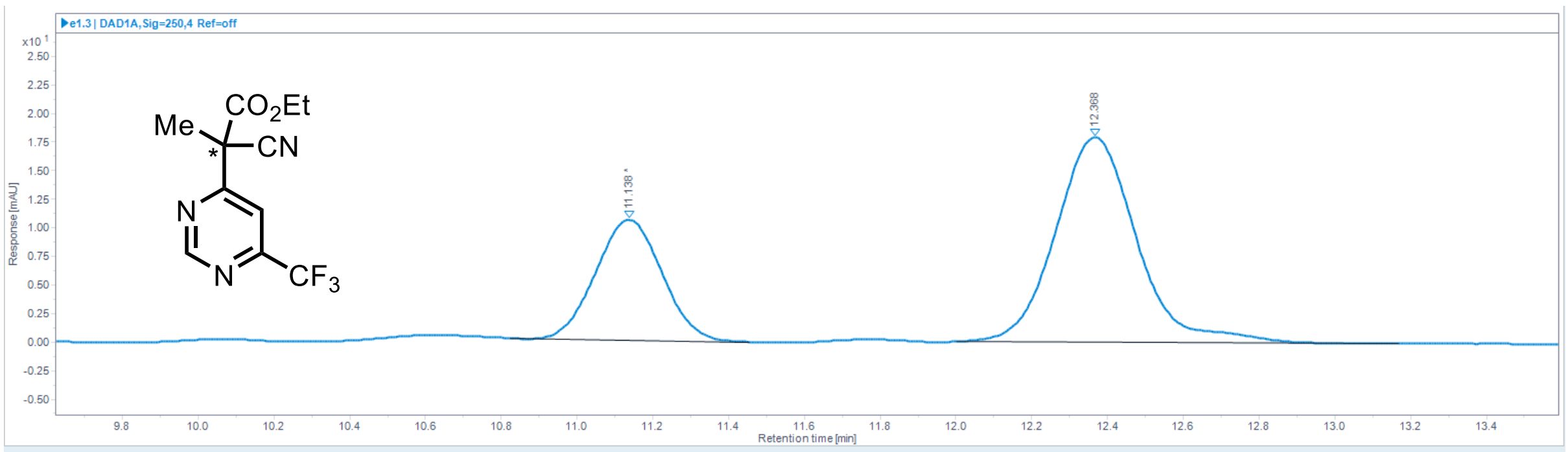

Injection Results

Peaks | Summary

#	Name	Signal description	RT (min)	Area (mAU·s)	Area%	Height (mAU)	Height%	Amount	Concentration	Start time (min)	End time (min)
1		DAD1A,Sig=250,4 Ref=off	11.138	128.063	32.388	10.518	36.97			10.825	11.457
2		DAD1A,Sig=250,4 Ref=off	12.368	267.340	67.612	17.933	63.03			12.002	13.169

Chiral HPLC chromatogram of enantioenriched **17a** from an analytical scale biotransformation using $eS_NAr1.3$

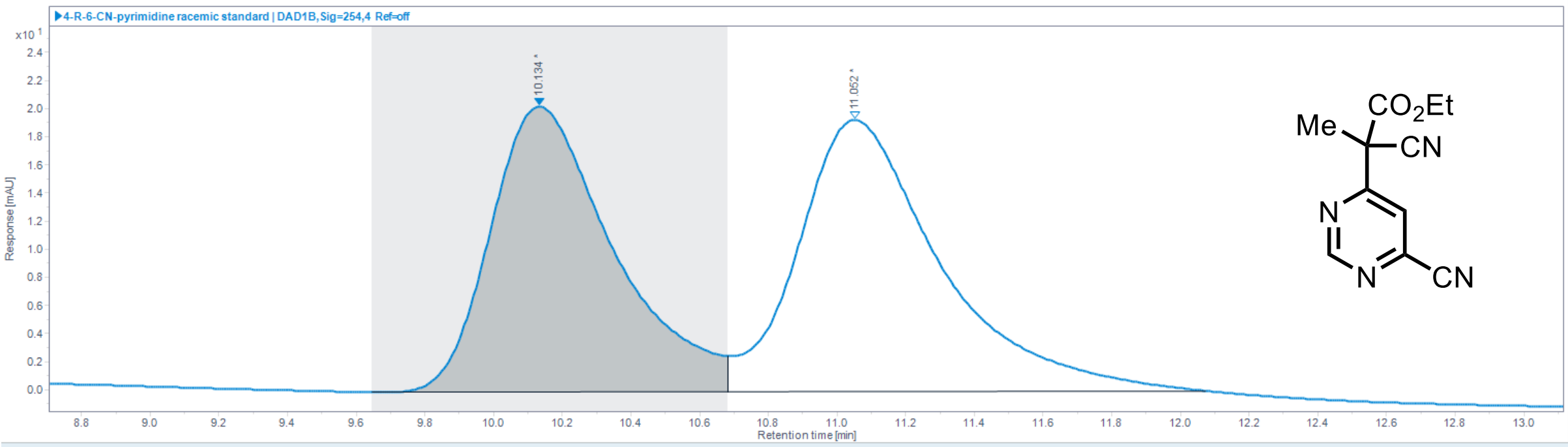

Injection Results

Peaks Summary

#	Name	Signal description	RT (min)	Area (mAU·s)	Area%	Height (mAU)	Height%	Amount	Concentration	Start time (min)	End time (min)
1		DAD1B,Sig=254,4 Ref=off	10.134	505.458	47.680	20.283	51.24			9.646	10.683
2		DAD1B,Sig=254,4 Ref=off	11.052	554.639	52.320	19.300	48.76			10.683	12.073

Chiral HPLC chromatogram of rac-**18a**

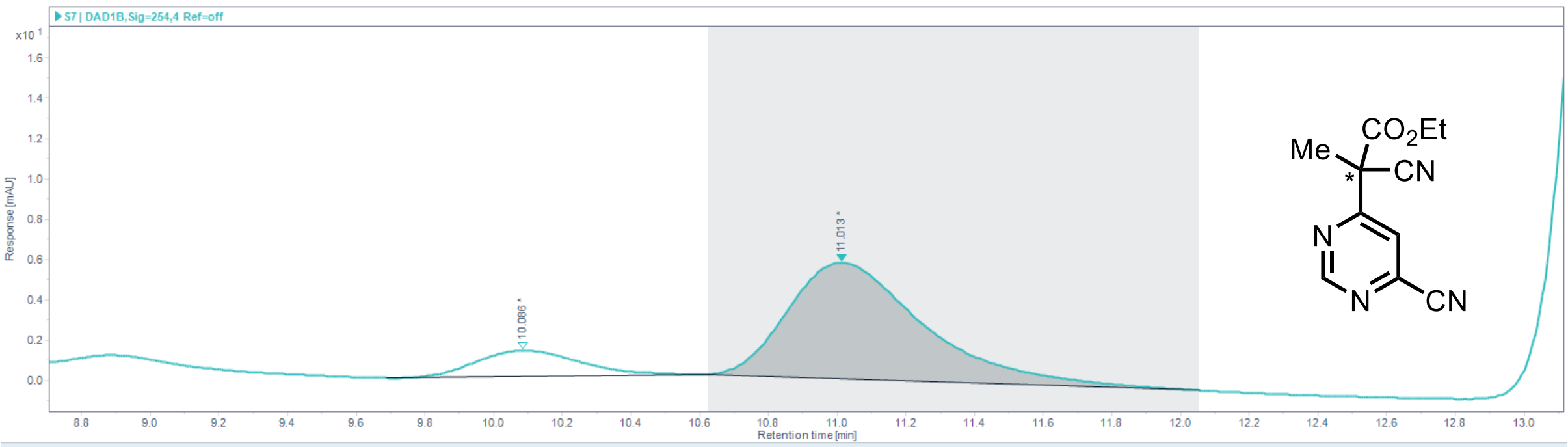

Injection Results

Peaks Summary

#	Name	Signal description	RT (min)	Area (mAU·s)	Area%	Height (mAU)	Height%	Amount	Concentration	Start time (min)	End time (min)
1		DAD1B,Sig=254,4 Ref=off	10.086	27.158	14.582	1.284	18.21			9.689	10.626
2		DAD1B,Sig=254,4 Ref=off	11.013	159.081	85.418	5.767	81.79			10.626	12.056

Chiral HPLC chromatogram of enantioenriched **18a** from an analytical scale biotransformation using eS_NAr1.2(E9D L13F R65V)

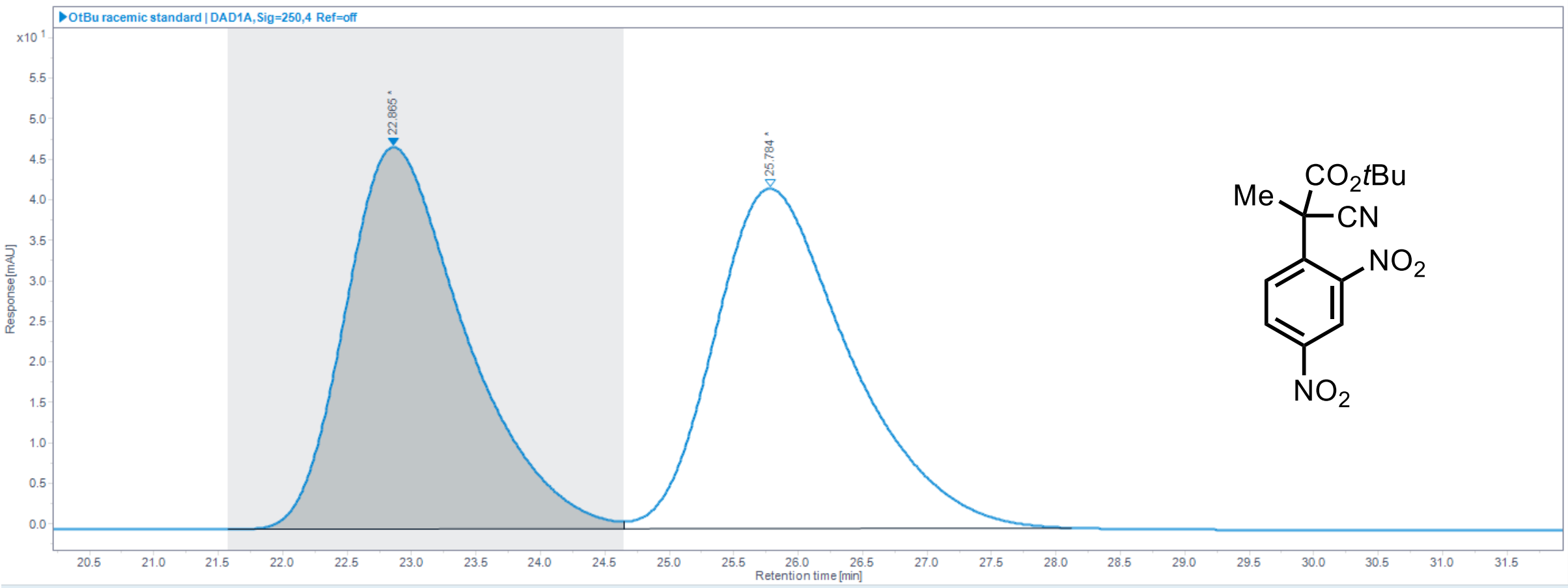

Injection Results

Peaks Summary

#	Name	Signal description	RT (min)	Area (mAU·s)	Area%	Height (mAU)	Height%	Amount	Concentration	Start time (min)	End time (min)
1		DAD1A,Sig=250,4 Ref=off	22.865	3063.017	50.028	47.149	52.92			21.585	24.650
2		DAD1A,Sig=250,4 Ref=off	25.784	3059.585	49.972	41.942	47.08			24.650	28.124

Chiral HPLC chromatogram of rac-**19**

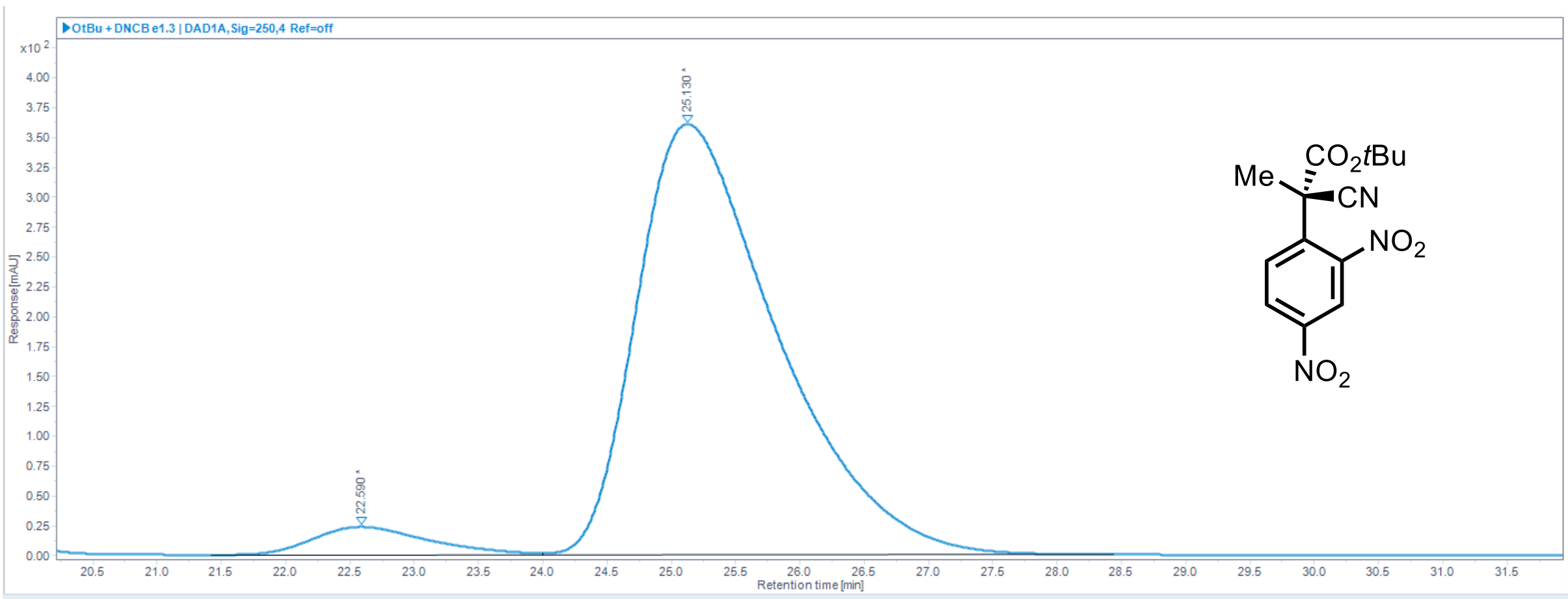

Injection Results

Peaks Summary

#	Name	Signal description	RT (min)	Area (mAU·s)	Area%	Height (mAU)	Height%	Amount	Concentration	Start time (min)	End time (min)
1		DAD1A,Sig=250,4 Ref=off	22.590	1464.423	5.076	23.392	6.10			21.422	24.001
2		DAD1A,Sig=250,4 Ref=off	25.130	27385.452	94.924	360.246	93.90			24.001	28.445

Chiral HPLC chromatogram of **(*S*)-19** from an analytical scale biotransformation using eS_NAr1.3

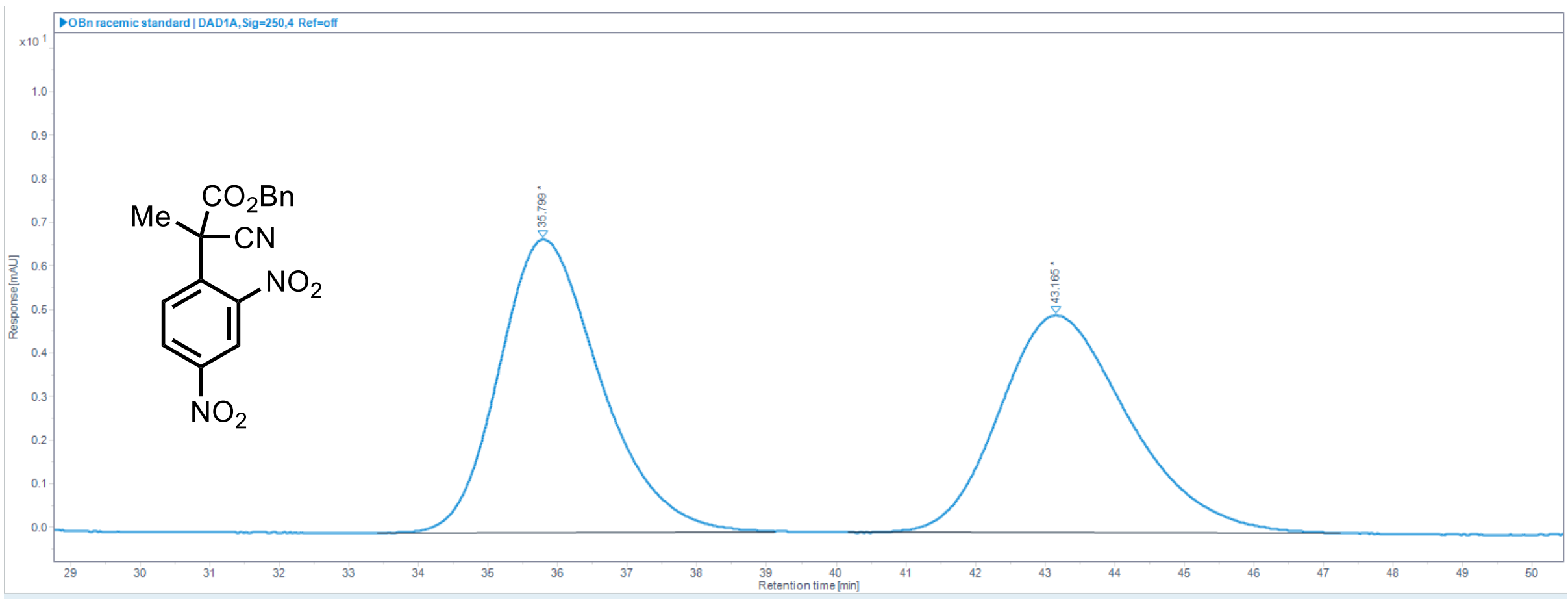

Injection Results

Peaks | Summary

Name	RT (min)	Area (mAU·s)	Area%	Height (mAU)	Height%
	35.799	682.066	51.298	6.727	57.40
	43.165	647.548	48.702	4.992	42.60

Chiral HPLC chromatogram of rac-**20**

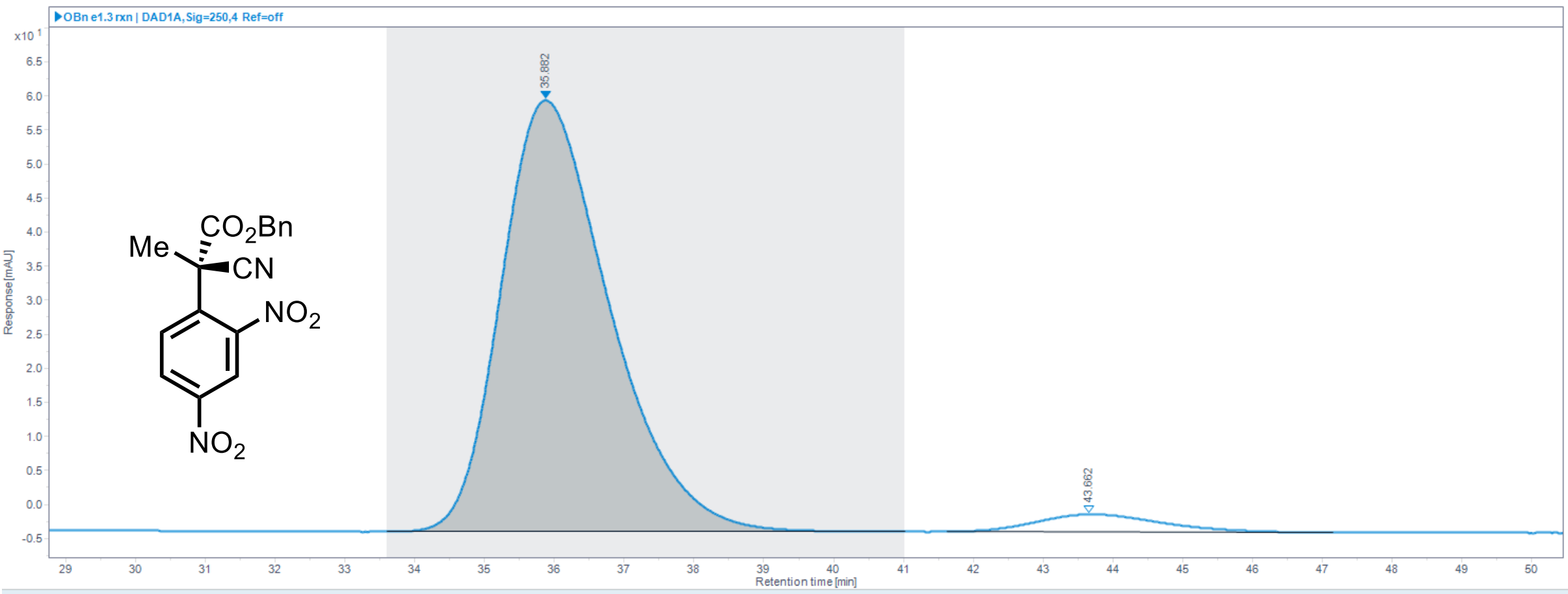

Injection Results

Peaks | Summary

Name	RT (min)	Area (mAU·s)	Area%	Height (mAU)	Height%
	35.882	6866.870	95.504	63.358	96.07
	43.662	323.247	4.496	2.590	3.93

Chiral HPLC chromatogram of **(*S*)-20** from an analytical scale biotransformation using $eS_NAr1.3$

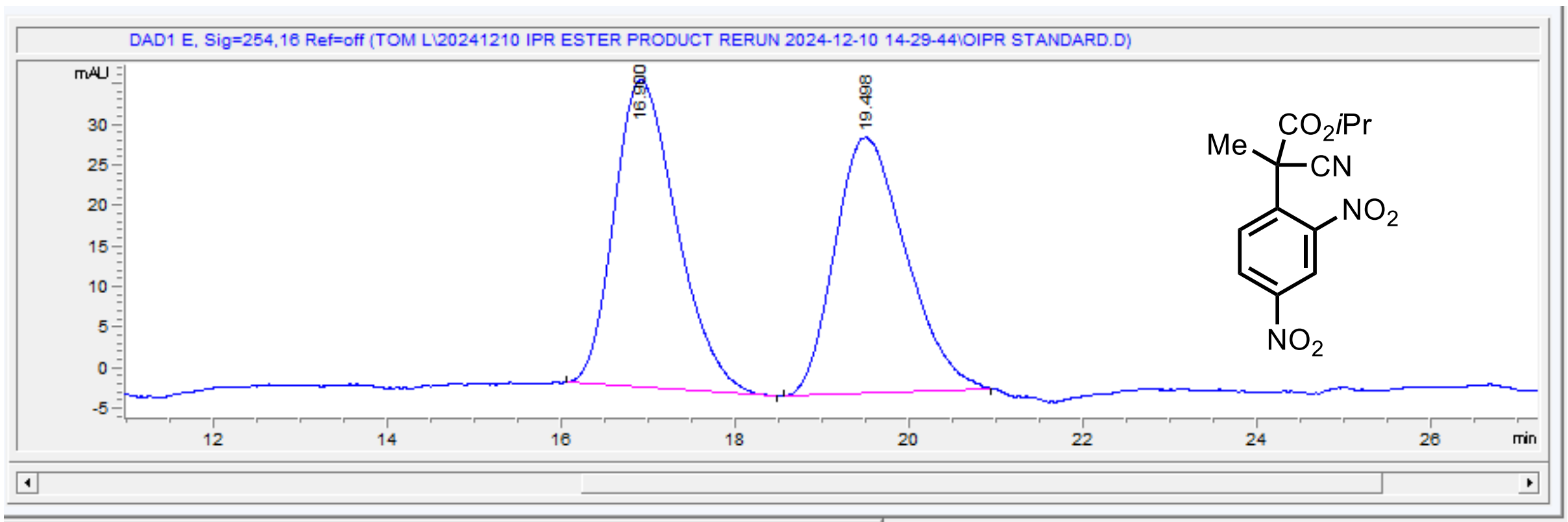

#	Time	Area	Height	Width	Area%	Symmetry
1	16.9	1895.6	37.8	0.5912	51.284	0.708
2	19.498	1800.7	31.5	0.6859	48.716	0.773

Chiral HPLC chromatogram of rac-**21**

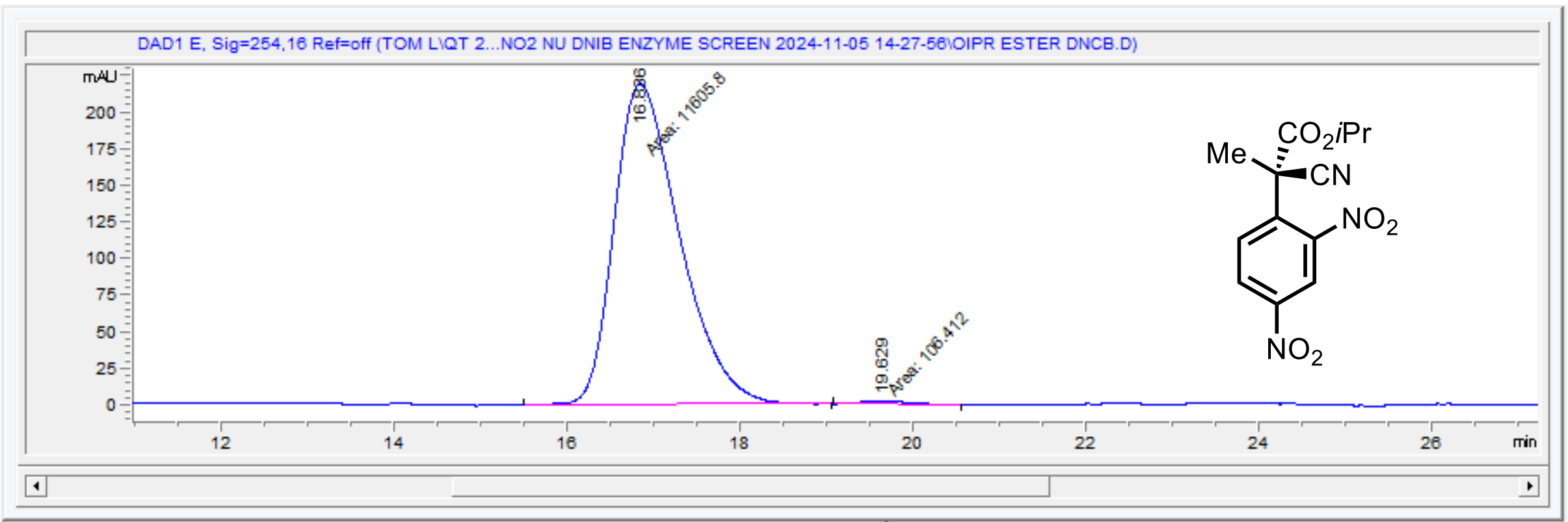

#	Time	Area	Height	Width	Area%	Symmetry
1	16.836	11605.8	219.3	0.882	99.091	0.637
2	19.629	106.4	2.4	0.7378	0.909	0.615

Chiral HPLC chromatogram of **(*S*)-21** from an analytical scale biotransformation using $eS_NAr1.3$

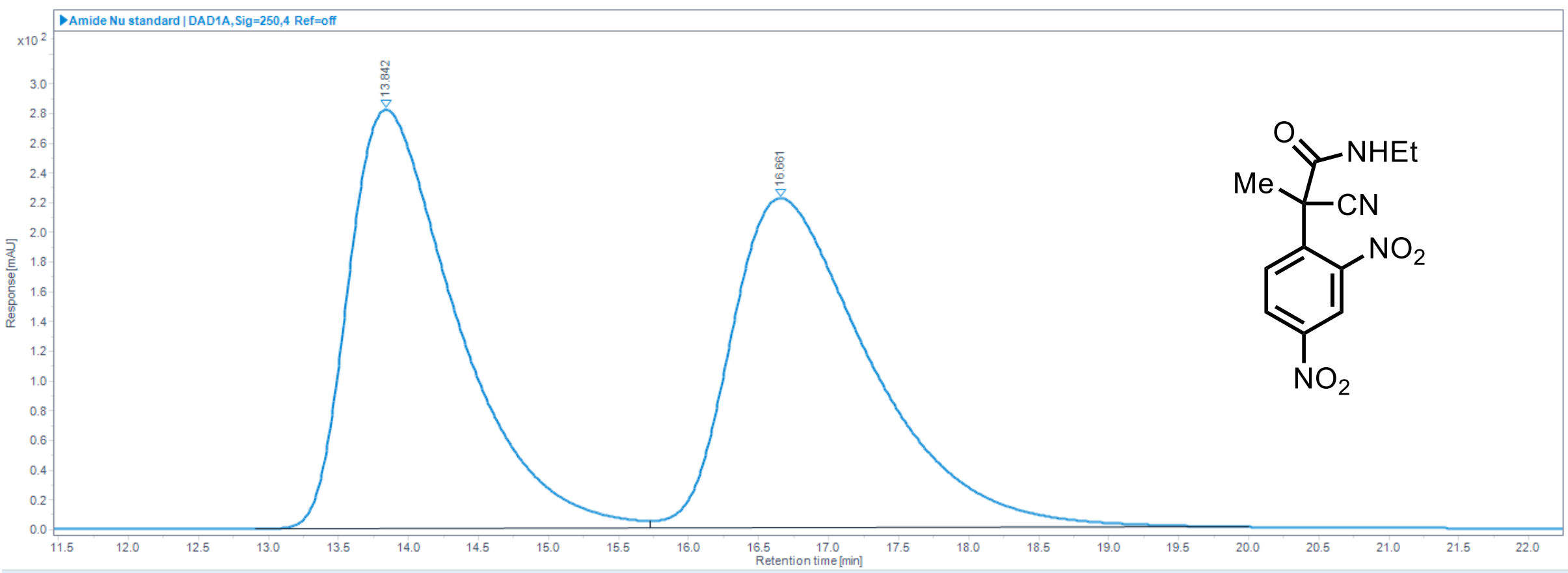

Injection Results

Peaks | Summary

#	Name	Signal description	RT (min)	Area (mAU·s)	Area%	Height (mAU)	Height%	Amount	Concentration	Start time (min)	End time (min)
1		DAD1A,Sig=250,4 Ref=off	13.842	15217.349	49.672	281.956	55.96			12.911	15.726
2		DAD1A,Sig=250,4 Ref=off	16.661	15418.099	50.328	221.910	44.04			15.726	20.004

Chiral HPLC chromatogram of rac-**22**

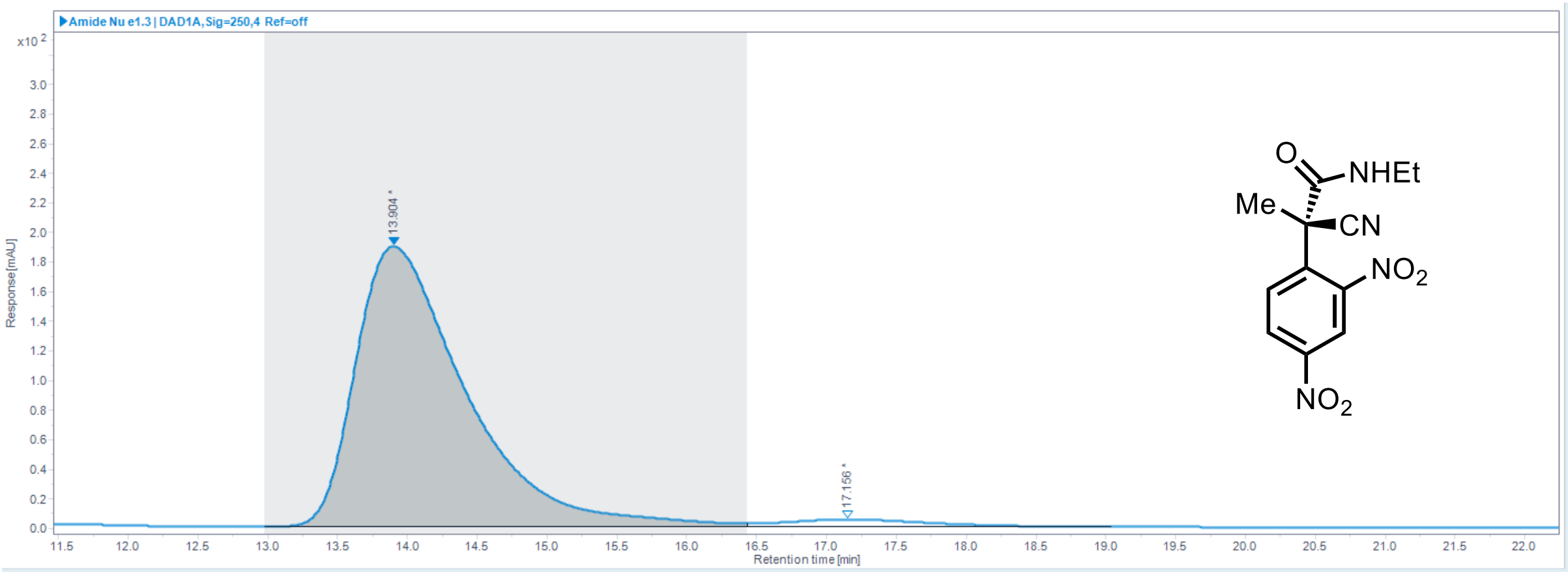

Injection Results

Peaks | Summary

#	Name	Signal description	RT (min)	Area (mAU·s)	Area%	Height (mAU)	Height%	Amount	Concentration	Start time (min)	End time (min)
1		DAD1A,Sig=250,4 Ref=off	13.904	10441.683	96.720	189.477	97.47			12.983	16.440
2		DAD1A,Sig=250,4 Ref=off	17.156	354.107	3.280	4.908	2.53			16.440	19.037

Chiral HPLC chromatogram of **(S)-22** from an analytical scale biotransformation using eS$_N$Ar1.3

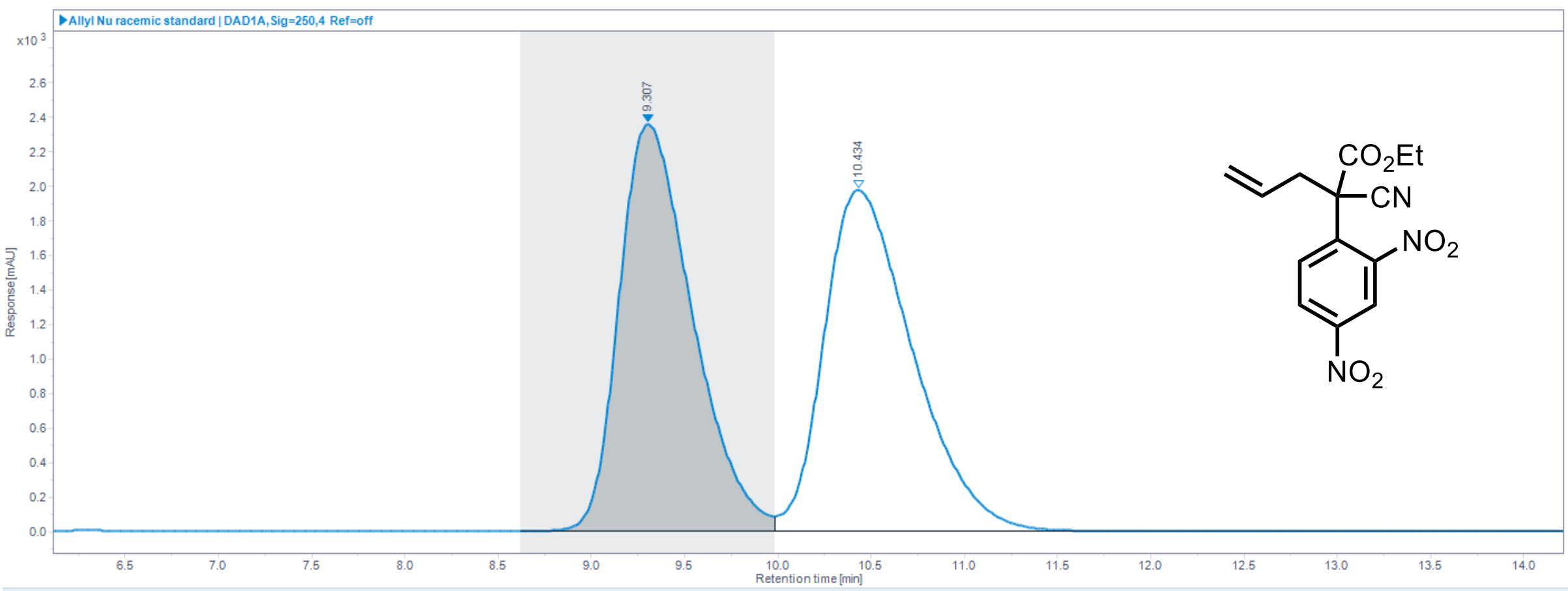

Injection Results

Peaks | Summary

#	Name	Signal description	RT (min)	Area (mAU·s)	Area%	Height (mAU)	Height%	Amount	Concentration	Start time (min)	End time (min)
1		DAD1A,Sig=250,4 Ref=off	9.307	64594.472	49.589	2356.543	54.40			8.622	9.985
2		DAD1A,Sig=250,4 Ref=off	10.434	65666.197	50.411	1975.219	45.60			9.985	15.727

Chiral HPLC chromatogram of rac-**23**

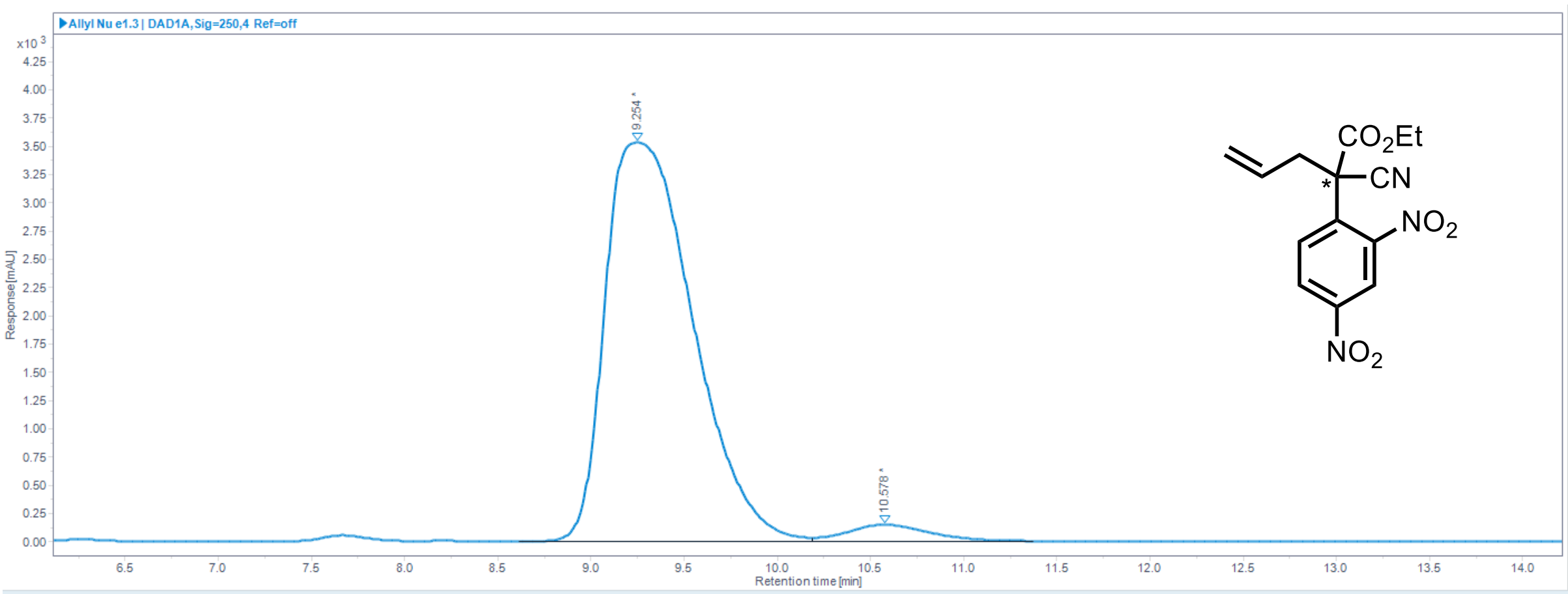

Injection Results

Peaks | Summary

#	Name	Signal description	RT (min)	Area (mAU·s)	Area%	Height (mAU)	Height%	Amount	Concentration	Start time (min)	End time (min)
1		DAD1A,Sig=250,4 Ref=off	9.254	115690.508	96.341	3528.533	96.07			8.620	10.191
2		DAD1A,Sig=250,4 Ref=off	10.578	4393.993	3.659	144.394	3.93			10.191	11.376

Chiral HPLC chromatogram of enantioenriched **(*S*)-23** from an analytical scale biotransformation using eS$_N$Ar1.3

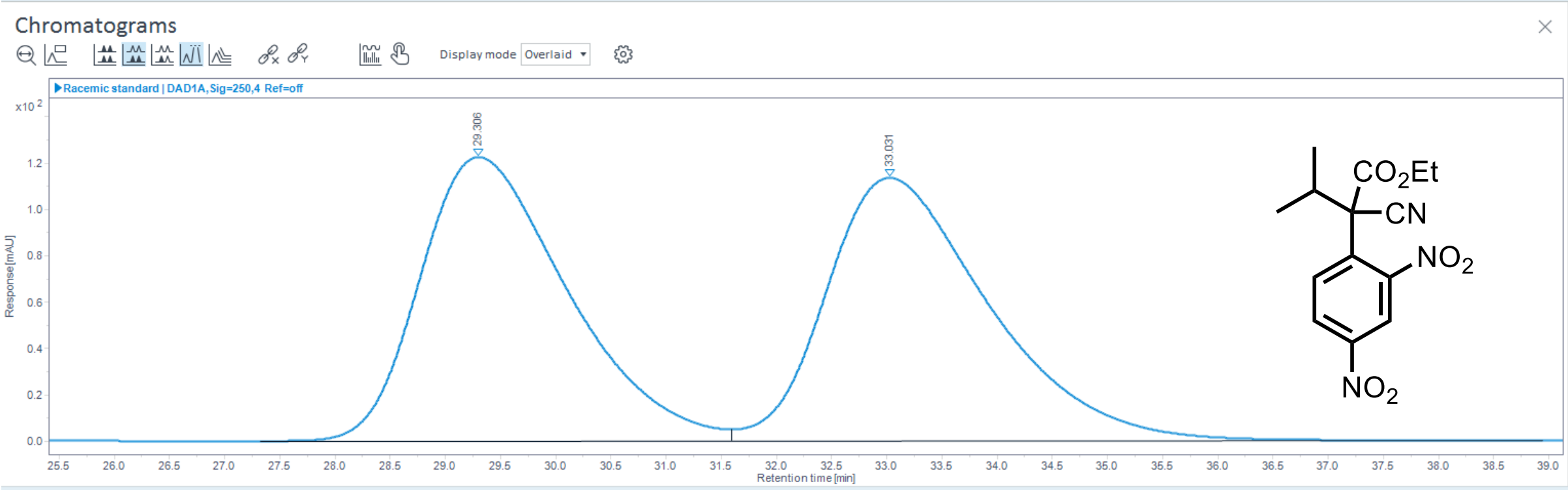

Injection Results

Peaks | Summary

#	Name	Signal description	RT (min)	Area (mAU·s)	Area%	Height (mAU)	Height%	Amount	Concentration	Start time (min)	End time (min)
1		DAD1A,Sig=250,4 Ref=off	29.306	11406.975	49.440	122.507	51.92			27.330	31.601
2		DAD1A,Sig=250,4 Ref=off	33.031	11665.376	50.560	113.468	48.08			31.601	38.947

Chiral HPLC chromatogram of rac-**24**

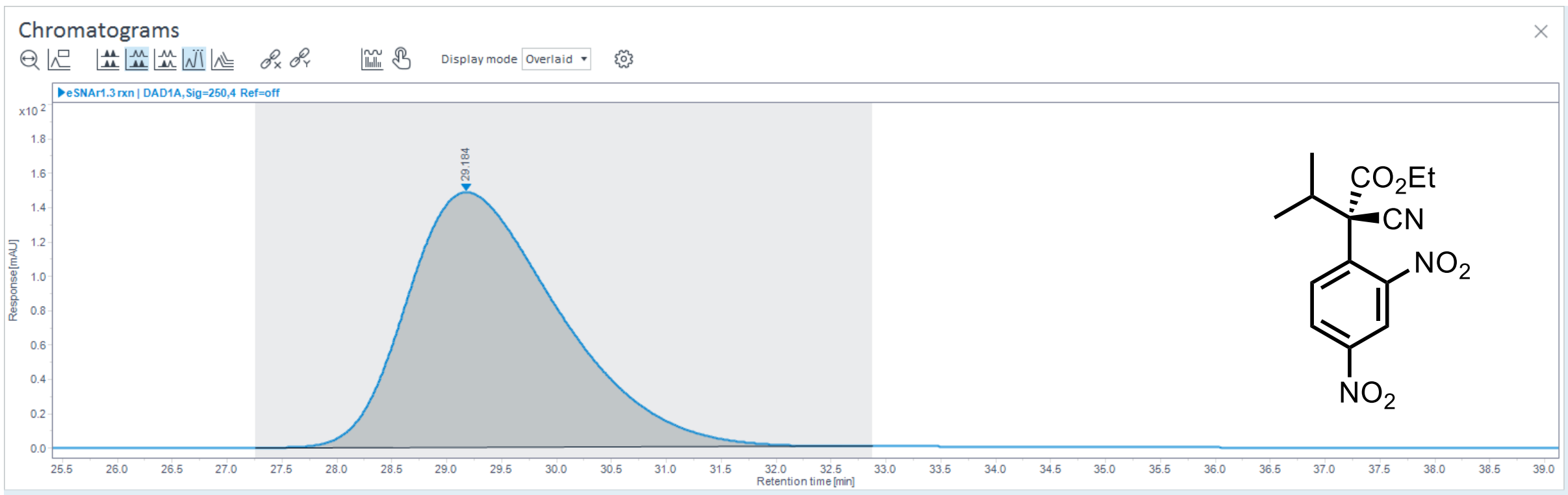

Injection Results

Peaks | Summary

#	Name	Signal description	RT (min)	Area (mAU·s)	Area%	Height (mAU)	Height%	Amount	Concentration	Start time (min)	End time (min)
1		DAD1A,Sig=250,4 Ref=off	29.184	14357.568	100.000	148.343	100.00			27.264	32.881

Chiral HPLC chromatogram of **(S)-24** from an analytical scale biotransformation using eS$_N$Ar1.3

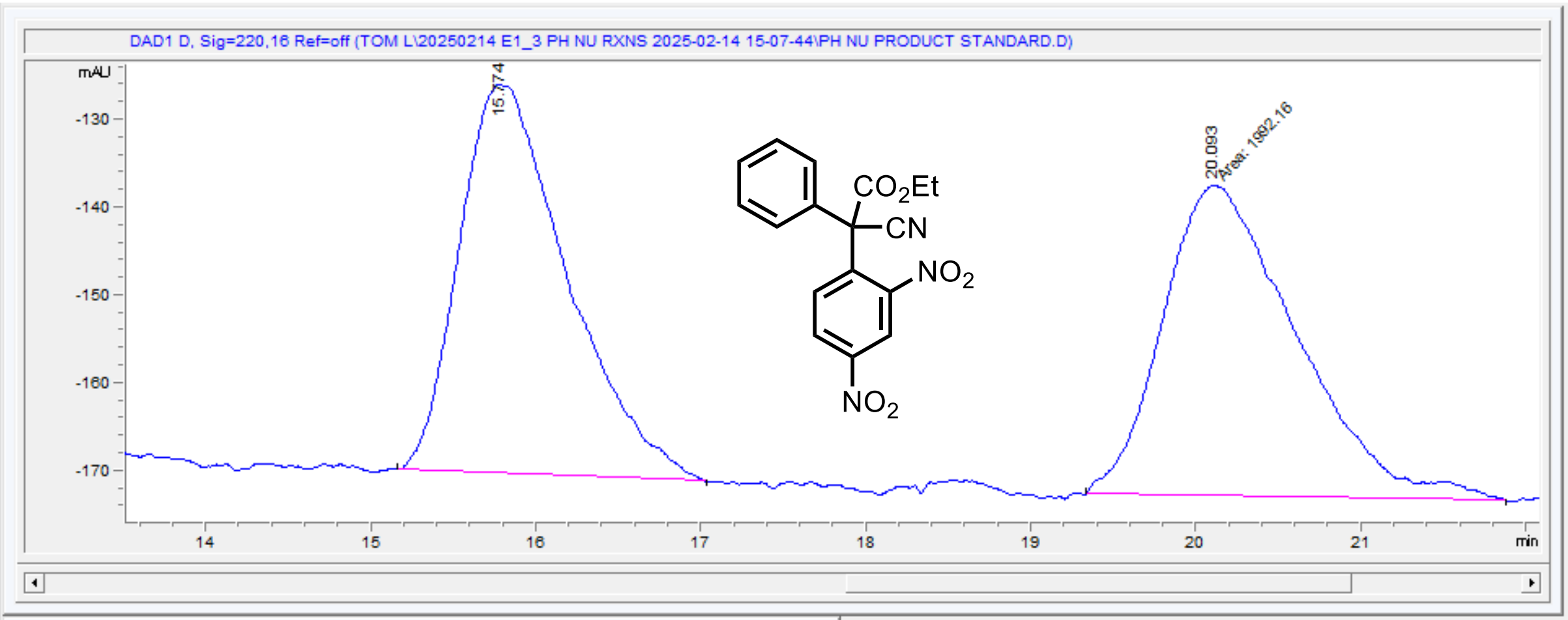

#	Time	Area	Height	Width	Area%	Symmetry
1	15.774	2013.9	44.3	0.5809	50.272	0.549
2	20.093	1992.2	35.3	0.9402	49.728	0.57

Chiral HPLC chromatogram of rac-**25**

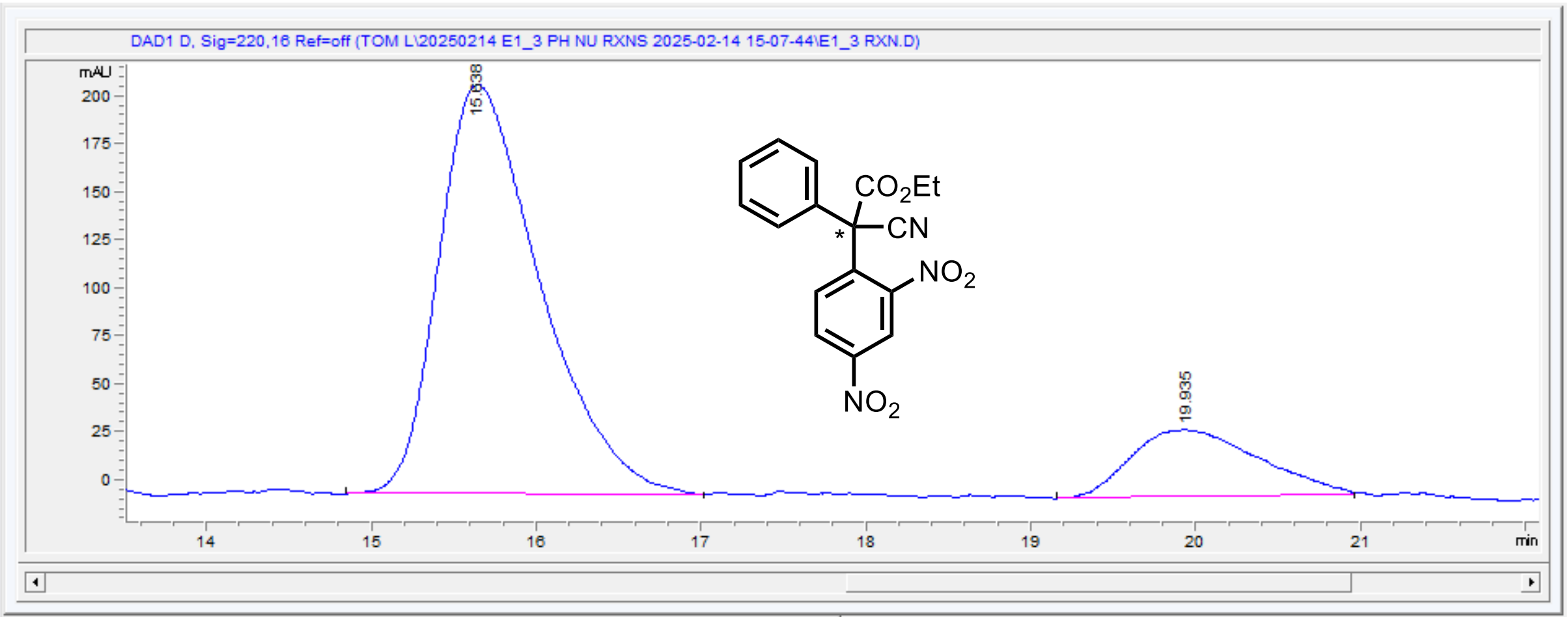

#	Time	Area	Height	Width	Area%	Symmetry
1	15.638	9163.6	213.7	0.6304	82.700	0.584
2	19.935	1916.9	35.1	0.642	17.300	0.717

Chiral HPLC chromatogram of **(*S*)-25** from an analytical scale biotransformation using eS_NAr1.3

Supplementary References

1. Lister, T. M. *et al.* Engineered enzymes for enantioselective nucleophilic aromatic substitutions. *Nature* **639**, 375–381 (2025).
