# Supplementary material for: Directed evolution of an enantiocomplementary SNArase reveals divergent catalytic features
Source: Nat Commun. 2026 Aug 21;17:10033. doi: 10.1038/s41467-026-76922-5 (PMC13594109; doi:10.1038/s41467-026-76922-5)
Supplement: Supplementary file 3 [file 41467_2026_76922_MOESM3_ESM.pdf]

nature portfolio

Peer Review File

Directed Evolution of an Enantiocomplementary S_NArase Reveals Divergent Catalytic Features

Corresponding Author: Professor Anthony Green

This file contains all reviewer reports in order by version, followed by all author rebuttals in order by version.

Version 0:

Reviewer comments:

Reviewer #1

(Remarks to the Author)
The manuscript by Green, Osuna and coworkers describes the directed evolution of an enantiocomplementary enzyme, eSNAr1.3, engineered to catalyze nucleophilic aromatic substitution reactions for the formation of all-carbon quaternary centers. Building upon a previously engineered ancestor that favored the (R)-enantiomer, the authors successfully redirected the evolutionary trajectory to generate a variant that selects for the (S)-enantiomer with high stereocontrol. Beyond achieving the targeted inversion of stereochemistry, the study reports significant enhancements in catalytic efficiency and a broadened substrate scope, effectively demonstrating the versatility of laboratory evolution in reprogramming enzyme mechanisms to access diverse synthetic outcomes. Although substrate scope reflects some limitations in terms of substitution patterns, reactions are consistently enantioselective, with few exceptions. The experimental work is robust and well-conducted, providing a thorough biochemical and structural characterization of the evolved biocatalyst. The kinetic analyses are rigorous, clearly quantifying the substantial rate improvements, while the X-ray crystallographic data offers valuable structural context. These experimental findings are strongly supported by molecular dynamics simulations, which rationalize the achieved enantiocomplementarity through steric blocking by the A19L mutation and identifying a novel nucleophile activation dyad involving His23 and Arg124. The manuscript is well-written and logically structured, presenting evolutionary and mechanistic data with clarity. Therefore I recommend publication with no further changes.

Reviewer #2

(Remarks to the Author)
Osuna, Green, and co-workers reported the development of a SNAr enzyme with complementary enantioselectivity to their previously engineered one, expanding the synthetic utility of SNAr biocatalysts. The conserved and divergent mechanistic features of two SNAr enzymes, SNAr1.3 and eSNAr1.3, are well illustrated. However, I am concerned about the novelty of the present work. For both SNAr1.3 and eSNAr1.3, the conserved Arg124 residue activates the nucleophile through H-bonding with its enolate anion. The His23 residue in eSNAr1.3 facilitates catalysis by positioning the nucleophile substrate close to the electrophile. The authors speculate that His23 is also involved in nucleophile activation by promoting the enolate formation, which lacks experimental or computational support. The claim of a distinct nucleophile activation mechanism is therefore questionable. Compared to SNAr1.3, the scope of eSNAr1.3 is not expanded in terms of substrate variety.

Other points:
(1) When discussing the effect of halide leaving group on eSNAr1.3 activity and selectivity (Figure 3), the fluoride leaving group is missing. Yet, fluoride electrophiles are heavily used in the scope study.
(2) What are the reaction results with alternative halide leaving groups in Figure 4? It is unclear how the halide leaving groups are chosen. Can the authors provide a guidance?
(3) The directed evolution of eSNAr was performed with cell lysates, while analytical reactions were conducted with purified proteins. Can cell lysates or whole-cells be used for reactions?
(4) A protocol for preparative scale reaction is missing.
(5) For some products that don’t undergo hydrolysis-decarboxylation, what is the reason for low yields? What’s the mass balance?
(6) It should be noted in the manuscript that the hydrolysis-decarboxylation process is non-enzymatic to avoid misunderstanding that this side reaction affects the product ee.

(7) Page 9, “We considered the possibility that His23 might serve as a catalytic nucleophile to accelerate SNAr chemistry, however this hypothesis was excluded as we were unable to accumulate any arylated protein intermediates within catalytically relevant timeframes (see Supplementary information, Methods)” Where is this Methods section in SI?
(8) Is the enzyme denatured after ~2000 turnovers? What are the possible factors for enzyme deactivation?
(9) Figure 4, product 25, a should be f ?
(10) It is difficult to read Figure 5b and 5e. Consider reorganizing Figure 5.
(11) The author names in reference 19 are incomplete.

Reviewer #3

(Remarks to the Author)
This manuscript reports the impressive engineering and comprehensive characterization of an enantiocomplementary biocatalyst, eSNAr1.3, for nucleophilic aromatic substitution (SNAr) reactions. The work builds logically upon the authors' prior development of SNAr1.3 and successfully addresses a significant challenge in the inversion of stereoselectivity while enhancing activity. The combination of directed evolution, detailed biochemical and structural analysis, and sophisticated computational modeling provides a compelling and multi-faceted understanding of how divergent evolutionary trajectories can lead to distinct, yet highly efficient, catalytic solutions. The study is well-executed, with the findings are of considerable interest to the fields of biocatalysis, enzyme engineering, and synthetic organic chemistry. The major comments are outlined below.
The mechanistic analysis primarily relies on ground-state binding poses (distance, dihedral angle, etc) and conformational sampling, a common and often insightful approach in enzymology. However, to fully validate the proposed catalytic mechanism, QM/MM calculations exploring the bond-forming/breaking transition state would provide a more definitive picture. A discussion acknowledging this perspective would strengthen the manuscript.
It is recommended to analyze the correlation between catalytic activity and thermal stability. The study comprehensively characterizes the dramatic improvements in activity and enantioselectivity achieved through directed evolution. However, it does not address whether these beneficial mutations, particularly those reshaping the active site, have concomitantly affected the enzyme's thermal stability. It is a common observation in protein engineering that mutations enhancing activity can sometimes destabilize the folded state. Providing data on the melting temperature (Tm) or residual activity after incubation at elevated temperatures for key variants would be highly valuable.
In Figure 2c and 2d, the color palette used to distinguish different variants along the evolutionary trajectory (e.g., grey, light green, green) may present challenges in visual discrimination. To improve accessibility and clarity, it is recommended to replace the current color scheme with a set of colors that offer higher contrast and are more easily distinguishable.

Reviewer #4

(Remarks to the Author)
Lister et al. determine an X-ray structure to study the binding mode of the electrophile substrate and they use MD simulation to investigate the conformational flexibility and the formation of catalytically competent states for SNAr1.3 and eSNAr1.3. The structural studies are well described and the discussion is sound.

Figure 1: Label Arg124. It is probably the arginine in front of the figure but there is a residue shown in the back, which might also be an arginine.

Conclusion page 12: The authors describe a "distinctive nucleophile activation mechanism" and in the next sentence again "distinctive mechanisms". For sure, the active site geometry differs including the identity of protein residues binding the two substrates, which results in different stereochemical outcome. This is well described and analyzed. But I am less convinced about the different mechanisms. What is the catalytic mechanism in detail and how does it differ? How is the nucleophile activated? A different geometry and identity of the active site alone does not indicate a different mechanism. How exacly does the arginine or histidine *activate* a nucleophile, in contrast to a role in positioning the reactants in a catalytically competent way?

Page 18: Free Energy Landscape (FEL) reconstruction. Is the free energy landscape shown or analyzed. I think the authors just show the conformational landscape. It is not obvious to me why this is a *re*construction, but this seems to be common terminology.

Caption of Supplementary figure 16: "... leading to Arg124 being significantly more ordered SNAr1.3(K39A) in ..." -> "... more ordered than in SNAr1.3(K39A) in ..." ?

Figure S22: It would help the interpretation of this figure if - similar to figure S21 - the catalytic distances were represented as arrows of nucleophilic attack.

Supplementary Table 10: Please set in the space group symbol P 31 2 1 the first "1" in subscript. Please show for all R-values (data reduction and refinement) also the values for the highest resolution shell. The I/sigma(I) and the CC1/2 values indicate that data to higher resolution could have been used. Was no higher resolution data collected?

Version 1:

Reviewer comments:

Reviewer #2

(Remarks to the Author)
All my conerns have been carefully addressed. The manuscript is now ready for publication.

Reviewer #3

(Remarks to the Author)
The authors have been revised their manuscript accordingly, which is acceptable now.

Reviewer #4

(Remarks to the Author)
The authors have satisfactorily addressed all points I raised in my review.
Crystal structure coordinates, structure factors or validation report were not provided for review.
I noticed further minor points:
Figure legend Fig. S24: Boltz-1 -> Boltz-2
Figure S30: "X-ray bound molecules" instead of "X-ray bonded molecules"?
Table S10: "Resolution range" add "(Å)"
Table S10: "Wilson B-factor" add "(Å**2)"
Table S10: "RMS(bonds)" add "(Å)"
Table S10: "RMS(angles)" add "(°)"
Table S10: "Clashscore" -> "Molprobity clashscore" (exactly 2.0?, specify one digit after comma)
Table S10: " Average B-factor " add "(Å**2)"

Open Access This Peer Review File is licensed under a Creative Commons Attribution 4.0 International License, which permits use, sharing, adaptation, distribution and reproduction in any medium or format, as long as you give appropriate credit to the original author(s) and the source, provide a link to the Creative Commons license, and indicate if changes were made.
In cases where reviewers are anonymous, credit should be given to 'Anonymous Referee' and the source.
The images or other third party material in this Peer Review File are included in the article's Creative Commons license, unless indicated otherwise in a credit line to the material. If material is not included in the article's Creative Commons license and your intended use is not permitted by statutory regulation or exceeds the permitted use, you will need to obtain permission directly from the copyright holder.
To view a copy of this license, visit https://creativecommons.org/licenses/by/4.0/

Response to reviewers' comments

We would like to thank the reviewers for their constructive feedback on our previous submission. We have now uploaded a revised version of the paper to the Nature Communications website that addresses the issues raised by the reviewers in their critique. The specific changes made are outlined in the reviewer response below and are also highlighted in red in the marked-up versions of the manuscript and supporting information.

Referee #1

Comment:

The manuscript by Green, Osuna and coworkers describes the directed evolution of an enantiocomplementary enzyme, eSNAr1.3, engineered to catalyze nucleophilic aromatic substitution reactions for the formation of all-carbon quaternary centers. Building upon a previously engineered ancestor that favored the (R)-enantiomer, the authors successfully redirected the evolutionary trajectory to generate a variant that selects for the (S)-enantiomer with high stereocontrol. Beyond achieving the targeted inversion of stereochemistry, the study reports significant enhancements in catalytic efficiency and a broadened substrate scope, effectively demonstrating the versatility of laboratory evolution in reprogramming enzyme mechanisms to access diverse synthetic outcomes. Although substrate scope reflects some limitations in terms of substitution patterns, reactions are consistently enantioselective, with few exceptions. The experimental work is robust and well-conducted, providing a thorough biochemical and structural characterization of the evolved biocatalyst. The kinetic analyses are rigorous, clearly quantifying the substantial rate improvements, while the X-ray crystallographic data offers valuable structural context. These experimental findings are strongly supported by molecular dynamics simulations, which rationalize the achieved enantiocomplementarity through steric blocking by the A19L mutation and identifying a novel nucleophile activation dyad involving His23 and Arg124. The manuscript is well-written and logically structured, presenting evolutionary and mechanistic data with clarity. Therefore I recommend publication with no further changes.

Response:

We thank the reviewer for their positive assessment of our work.

Referee #2

Comment:

Osuna, Green, and co-workers reported the development of a SNAr enzyme with complementary enantioselectivity to their previously engineered one, expanding the synthetic utility of SNAr biocatalysts. The conserved and divergent mechanistic features of two SNAr enzymes, SNAr1.3 and eSNAr1.3, are well illustrated. However, I am concerned about the novelty of the present work. For both SNAr1.3 and eSNAr1.3, the conserved Arg124 residue activates the nucleophile through H-bonding with its enolate anion. The His23 residue in eSNAr1.3 facilitates catalysis by positioning the nucleophile substrate close to the electrophile. The authors speculate that His23 is also involved in nucleophile activation by promoting the enolate formation, which lacks experimental or computational support. The claim of a distinct nucleophile activation mechanism is therefore questionable. Compared to SNAr1.3, the scope of eSNAr1.3 is not expanded in terms of substrate variety.

Response:

We thank the reviewer for their assessment of our work. To clarify the role of His23 and Arg124, we performed additional MD simulations and mechanistic QM/MM calculations. MD simulations of the H23A and R124A single mutants showed a dramatic decrease in the number of catalytically productive frames, consistent with the experimentally observed loss of activity. This reduction was observed in both mutants, suggesting that His23 and Arg124 act synergistically and are both required to properly orient and retain the nucleophile in the active site pocket for S_NAr. In the MD simulations of $eS_NAr1.3$, His23 predominantly adopts a single conformation, whereas Arg124 remains slightly more flexible and samples two major conformations, depending on whether it interacts directly with Asp9 or indirectly through a water molecule. The water-mediated interaction between Arg124 and Asp9 positions Arg124 close to the halide. QM/MM calculations indicated that the latter Arg124 conformation facilitates halide release during S_NAr. The overlay of the optimized transition state and reactant complex with the X-ray crystal structure of $eS_NAr1.3$, the parent MBH scaffold and several liganded $S_NAr1.3$ structures shows that the catalytically productive Arg124 conformation predicted by QM/MM is also observed crystallographically depending on the ligand present in the active site (Supplementary Fig. 30). This suggests that the proposed role of Arg124 in facilitating halide release during nucleophilic aromatic substitution might be shared by $eS_NAr1.3$ and $S_NAr1.3$.

Altogether the new calculations have revealed that $S_NAr1.3$ and $eS_NAr1.3$ share several active site features and mechanistic elements, but also some evolutionary divergencies:

(1) The well-defined halide-binding pocket of $S_NAr1.3$ has been abandoned in $eS_NAr1.3$, suggesting that this feature is not a prerequisite for efficient enzymatic S_NAr catalysis;
(2) His23 has emerged as a key catalytic residue in $eS_NAr1.3$, whereas this residue was abandoned in $S_NAr1.3$. MD simulations reveal His23 works in synergy with Arg124 to properly orient and retain the nucleophile in the active site pocket for S_NAr.
(3) Arg124 has been repositioned as a result of the Asp9 residue installed during evolution, which alters its flexibility and allows the adoption of a catalytically relevant pose important for promoting halide release during S_NAr catalysis. Mutation of Asp9 results in a substantial 24-fold decrease in rate with minimal impact on selectivity, highlighting its importance to $eS_NAr1.3$ catalysis.
(4) Interestingly, $S_NAr1.3$ and $eS_NAr1.3$ also display opposite preferences for the halide leaving group of the electrophilic coupling partner.

Taken together, this analysis provides crucial insights to guide future enzyme design campaigns to develop more proficient S_NArase biocatalysts. As a result of the reviewer's comments, we have revised the manuscript title to better reflect the divergent catalytic features of $S_NAr1.3$ versus $eS_NAr1.3$ rather than focusing solely on the altered nucleophile activation mechanism.

Comment:

(1) When discussing the effect of halide leaving group on eSNAr1.3 activity and selectivity (Figure 3), the fluoride leaving group is missing. Yet, fluoride electrophiles are heavily used in the scope study.

Response:

We thank the reviewer for raising this point. The high level of background reactivity with 2,4-dinitrofluorobenzene complicates accurate determination of initial rates with S_NAr variants. To address the reviewer's comment, we have now provided a complete analysis of the halide leaving group effect on $eS_NAr1.3$ activity, using the 4-halo-3-nitrobenzonitrile series of electrophiles. The activity follows the trend F > Cl ~ Br > I. Appropriate discussion is now included in the main text with an accompanying figure presented in the Supporting Information.

Comment:

(2) What are the reaction results with alternative halide leaving groups in Figure 4? It is unclear how the halide leaving groups are chosen. Can the authors provide a guidance?

Response:

We have now provided additional text in the legend of Figure 4 to justify the choice of halide leaving group.

Comment:

(3) The directed evolution of eSNAr was performed with cell lysates, while analytical reactions were conducted with purified proteins. Can cell lysates or whole-cells be used for reactions?

Response:

Purified protein was used for analytical reactions as allows for a more accurate and quantitative assessment/comparison of engineered enzyme variants. However, our enzymes are also able to operate efficiently in cell-free lysate, as evidenced by our analysis of over 6000 clones under these conditions during directed evolution. Furthermore, activity improvements identified in cell-free lysate reliably translated to purified protein.

Comment:

(4) A protocol for preparative scale reaction is missing.

Response:

We kindly direct the reviewer to pages 52 and 53 of the Supporting Information (‘Preparative Scale Biotransformations’) that describes the procedures and characterisation data.

Comment:

(5) For some products that don't undergo hydrolysis-decarboxylation, what is the reason for low yields? What's the mass balance?

Response:

With the exception of 14, 17 and 18 undergoing hydrolysis-decarboxylation, in all other cases a single reaction product was observed with remaining starting material making up the mass balance (>95% mass recovery was observed in all biotransformations). Additional text has been included in the manuscript.

Comment:

(6) It should be noted in the manuscript that the hydrolysis-decarboxylation process is non-enzymatic to avoid misunderstanding that this side reaction affects the product ee.

Response:

We thank the reviewer for highlighting this oversight. The text has now adjusted as requested.

Comment:

(7) Page 9, "We considered the possibility that His23 might serve as a catalytic nucleophile to accelerate SNAr chemistry, however this hypothesis was excluded as we were unable to accumulate any arylated protein intermediates within catalytically relevant timeframes (see Supplementary information, Methods)" Where is this Methods section in SI?

Response:

We thank the reviewer for noticing this omission, we have now included the protocol in the Methods section.

Comment:

(8) Is the enzyme denatured after ~2000 turnovers? What are the possible factors for enzyme deactivation?

Response:

We investigated why the $eS_NAr1.3$ is limited to 2000 turnovers with the 2,4-dinitrochlorobenzene electrophile. Interestingly, while the addition of fresh enzyme had no effect on turnover number, catalytic activity could be rescued by the addition of fresh nucleophile, suggesting that nucleophile degradation limits the total turnovers rather than enzyme stability. NMR analysis shows the nucleophile undergoes slow non-enzymatic ester hydrolysis under the assay conditions. We have added a brief discussion of these points to the main text and a figure into the Supporting Information.

Comment:

(9) Figure 4, product 25, a should be f ?

Response:

We thank the reviewer for noticing this error, the figure has now been corrected.

Comment:

(10) It is difficult to read Figure 5b and 5e. Consider reorganizing Figure 5.

Response:

We thank the reviewer for this suggestion. We have completely reorganized Figure 5 and also added the new computational insights obtained from the mechanistic QM/MM calculations.

Comment:

(11) The author names in reference 19 are incomplete.

Response:

We thank the reviewer for noticing this error, the reference has now been corrected.

Referee #3

Comment:

This manuscript reports the impressive engineering and comprehensive characterization of an enantiocomplementary biocatalyst, eSNAr1.3, for nucleophilic aromatic substitution (SNAr) reactions. The work builds logically upon the authors' prior development of SNAr1.3 and successfully addresses a significant challenge in the inversion of stereoselectivity while enhancing activity. The combination of directed evolution, detailed biochemical and structural analysis, and sophisticated computational modeling provides a compelling and multi-faceted understanding of how divergent evolutionary trajectories can lead to distinct, yet highly efficient, catalytic solutions. The study is well-executed, with the findings are of considerable interest to the fields of biocatalysis, enzyme engineering, and synthetic organic chemistry.

Response:

We thank the reviewer for their positive assessment of our work.

Comment:

The mechanistic analysis primarily relies on ground-state binding poses (distance, dihedral angle, etc) and conformational sampling, a common and often insightful approach in enzymology. However, to fully validate the proposed catalytic mechanism, QM/MM calculations exploring the bond-forming/breaking transition state would provide a more definitive picture. A discussion acknowledging this perspective would strengthen the manuscript.

Response:

We agree with the reviewer that QM/MM calculations could provide additional insights to those provided by conformational sampling and geometrical ground-state binding pose analysis. As described in referee 1 response, we have performed additional MD and QM/MM calculations to elucidate the role of His23, Arg124 and Asp9.

MD simulations of the H23A and R124 single mutants have shown that His23 and Arg124 are both essential for catalysis. Mutating either residue strongly reduces the number of catalytically productive conformations, matching the experimentally observed loss of activity. QM/MM calculations have revealed that Arg124 facilitates halide release during the S_NAr reaction when positioned near the halide through a water-mediated interaction with Asp9. This catalytically productive Arg124 conformation is also observed in X-ray structures, further supporting its mechanistic relevance in $eS_NAr1.3$.

Comment:

It is recommended to analyze the correlation between catalytic activity and thermal stability. The study comprehensively characterizes the dramatic improvements in activity and enantioselectivity achieved through directed evolution. However, it does not address whether these beneficial mutations, particularly those reshaping the active site, have concomitantly affected the enzyme's thermal stability. It is a common observation in protein engineering that mutations enhancing activity can sometimes destabilize the folded state. Providing data on the melting temperature (Tm) or residual activity after incubation at elevated temperatures for key variants would be highly valuable.

Response:

We thank the reviewer for their suggestion. Interestingly, in this instance the Tm was essentially unchanged across the evolutionary trajectory (T_m of variants ranges from 77.5 to 78.5 °C). These data have now been included in the revised manuscript with an accompanying Supplementary Figure.

Comment:

In Figure 2c and 2d, the color palette used to distinguish different variants along the evolutionary trajectory (e.g., grey, light green, green) may present challenges in visual discrimination. To improve accessibility and clarity, it is recommended to replace the current color scheme with a set of colors that offer higher contrast and are more easily distinguishable.

Response:

We thank the reviewer for their suggestion. We have now adjusted the colour scheme as suggested.

Referee #4

Comment:

Lister et al. determine an X-ray structure to study the binding mode of the electrophile substrate and they use MD simulation to investigate the conformational flexibility and the formation of catalytically competent states for SNAr1.3 and eSNAr1.3. The structural studies are well described and the discussion is sound.

Response:

We thank the reviewer for their positive assessment of our work.

Comment:

Figure 1: Label Arg124. It is probably the arginine in front of the figure but there is a residue shown in the back, which might also be an arginine.

Response:

We thank the reviewer for spotting this potential ambiguity. The figure has been amended accordingly.

Comment:

Conclusion page 12: The authors describe a "distinctive nucleophile activation mechanism" and in the next sentence again "distinctive mechanisms". For sure, the active site geometry differs including the identity of protein residues binding the two substrates, which results in different stereochemical outcome. This is well described and analyzed. But I am less convinced about the different mechanisms. What is the catalytic mechanism in detail and how does it differ? How is the nucleophile activated? A different geometry and identity of the active site alone does not indicate a different mechanism. How exactly does the arginine or histidine *activate* a nucleophile, in contrast to a role in positioning the reactants in a catalytically competent way?

Response:

A similar point was also raised by Reviewer 1. We expect that H-bonding interactions involving Arg124, His23 and the substrate acidify the α-C–H of the substrate and therefore promote formation of a reactive enolate state. We have included a statement to this effect in revised manuscript.

We have also performed additional MD simulations and QM/MM calculations to elucidate the catalytic mechanism and the role of residues His23, Arg124 and Asp9:
(1) MD simulations of the H23A and R124 single mutants have shown that His23 and Arg124 are both essential for catalysis. Mutating either residue strongly reduces the number of catalytically productive conformations, matching the experimentally observed loss of activity. His23 and Arg124 act synergistically and properly position the nucleophile for the reaction.

(2) QM/MM calculations have revealed that Arg124 facilitates halide release during the S_NAr reaction when positioned near the halide through a water-mediated interaction with Asp9. This catalytically productive Arg124 conformation is also observed in X-ray structures, supporting its mechanistic relevance in both eS_NAr1.3 and S_NAr1.3. However, S_NAr1.3 lacks an equivalent Asp9, making Arg124 more flexible and less preorganized, which likely contributes to its lower catalytic activity.

Altogether, the new calculations show that S_NAr1.3 and eS_NAr1.3 share key active site and mechanistic features but have diverged in important ways during evolution: (1) the well-defined halide-binding pocket present in S_NAr1.3 is no longer retained in eS_NAr1.3, indicating that this pocket is not required for efficient enzymatic S_NAr catalysis. (2) His23 has emerged as a key catalytic residue in eS_NAr1.3, whereas this residue was abandoned in S_NAr1.3. (3) The evolved Asp9 residue repositions Arg124, reducing its flexibility and promoting a catalytically relevant conformation that facilitates halide release. The strong 24-fold rate decrease observed upon Asp9 mutation, with little effect on selectivity, further supports its central role in eS_NAr1.3 catalysis. (4) S_NAr1.3 and eS_NAr1.3 show opposite preferences for the halide leaving group of the electrophilic coupling partner.

As a result of the reviewer's comments, we have revised the manuscript title to better reflect the divergent catalytic features of S_NAr1.3 versus eS_NAr1.3 rather than focusing solely on the altered nucleophile activation mechanism.

Comment:

Page 18: Free Energy Landscape (FEL) reconstruction. Is the free energy landscape shown or analyzed. I think the authors just show the conformational landscape. It is not obvious to me why this is a *re*construction, but this seems to be common terminology.

Response:

We thank the reviewer for the comment. The conformational landscape is shown and computed based on a selected catalytically relevant degrees of freedom (DOF): y axis is the dihedral angle between the nitrogen atom of the *ortho*-nitro substituent (electrophile), the halocarbon (electrophile), the halogen atom (electrophile) and the chiral carbon in the nucleophile, and the x axis corresponds to the catalytic distance between the halocarbon in the electrophile and the chiral carbon atom in the nucleophile. By using this set of catalytically relevant DOFs, the conformational landscape could be estimated (often termed reconstructed).

Comment:

Caption of Supplementary figure 16: "... leading to Arg124 being significantly more ordered SNAr1.3(K39A) in ..." -> "... more ordered than in SNAr1.3(K39A) in ..." ?

Response:

This has now been corrected.

Comment:

Figure S22: It would help the interpretation of this figure if - similar to figure S21 - the catalytic distances were represented as arrows of nucleophilic attack.

Response:

We thank the reviewer for this suggestion. A new revised Figure S22 (now S31) has been included.

Comment:

Supplementary Table 10: Please set in the space group symbol P 31 2 1 the first "1" in subscript. Please show for all R-values (data reduction and refinement) also the values for the highest resolution shell. The I/sigma(I) and the CC1/2 values indicate that data to higher resolution could have been used. Was no higher resolution data collected?

Response:

We have amended the space group notation and displayed additional R-values in accordance with the reviewers suggestion. The resolution cutoffs were chosen to be closer to the values given by a paired refinement performed on the PDB redo server (please see results attached), his lower resolution cutoff facilitated clearer interpretation of the electron density maps.

**Crystallographic data**							
**From original header**							
Spacegroup	P 31 2 1	*a:* 70.396 Å	*b:* 70.396 Å	*c:* 117.124 Å	*α:* 90.00°	*β:* 90.00°	*γ:* 120.00°
Resolution	2.10 Å	*Reflections*	18105	*Test set*	980 (5.4%)		
R	0.1828	*R-free*	0.2281				
**According to PDB-REDO**							
Resolution	2.18 Å	*Reflections*	18105	*Test set*	878 (4.8%)		
Twin	false	*Radiation damage*	N/A				
**PDB-REDO files**							
Re-refined and rebuilt structure<br>PDB | mmCIF | MTZ		Re-refined (only) structure<br>PDB | MTZ		All files<br>(compressed)			
**REFMAC5 command files**							

Response to reviewers' comments

We would like to thank the reviewers for their constructive feedback on our previous submission. We have now uploaded a revised version of the Supporting Information to the *Nature Communications* website that addresses the minor issues raised by reviewer #4.

Referee #2

Comment:

All my concerns have been carefully addressed. The manuscript is now ready for publication.

Response:

We thank the reviewer again for their constructive feedback.

Referee #3

Comment:

The authors have been revised their manuscript accordingly, which is acceptable now.

Response:

We thank the reviewer again for their constructive feedback.

Referee #4

Comment:

The authors have satisfactorily addressed all points I raised in my review.

Response:

We thank the reviewer again for their constructive feedback.

Comment:

Crystal structure coordinates, structure factors or validation report were not provided for review.

Response:

We have now included these in our resubmission.

Comment:

I noticed further minor points:

Figure legend Fig. S24: Boltz-1 -> Boltz-2

Figure S30: "X-ray bound molecules" instead of "X-ray bonded molecules"?

Table S10: "Resolution range" add "(Å)"

Table S10: "Wilson B-factor" add "(Å**2)"

Table S10: "RMS(bonds)" add "(Å)"

Table S10: "RMS(angles)" add "(°)"

Table S10: "Clashscore" -> "Molprobity clashscore" (exactly 2.0?, specify one digit after comma)

Table S10: " Average B-factor " add "(Å**2)"

Response:

We thank the reviewer for raising these minor points, we have now changed the text as requested.
